# Supplementary material for: Molecular dynamics simulations provide insights into ULK-101 potency and selectivity toward autophagic kinases ULK1/2
Source: bioRxiv. 2023 Dec 3:2023.12.01.569261. Preprint. [Version 1] doi: 10.1101/2023.12.01.569261 (PMC10705459; doi:10.1101/2023.12.01.569261)
Supplement: Supplement 1 [file media-1.pdf]

Supplemental information - *Molecular dynamics simulations provide insights into ULK-101 potency and selectivity toward autophagic kinases ULK1/2*

- Example code for how these simulations were run on a cluster
- Required input files to fun fABMACS. https://github.com/BradleyDickson/fABMACS
- proc.pdb – coordinate file for refined ULK2 and ULK-101 for simulation

Code for equilibration with GROMACS and running fABMACS.

#build system and relax/equilibrate with GROMACS

```
pdb2gmx_mpi -f FILE -o proc.pdb -water TIP3P -ignh -ter
#where FILE is just your clean protein
#add ligand pdb ATOM after TER in proc.pdb followed by ENDMDL
#add to topol.top  #include "ligand.itp" after charm in forcefield parameters and add ligand.itp
to working dir and add ligand to [ molecules ] section

editconf_mpi -f proc.pdb -o boxed.gro -c -d 1 -bt triclinic

gmx_mpi solvate -cp boxed.gro -cs spc216.gro -o solv.gro -p topol.top

grompp_mpi -f ~/GROrep/minim.mdp -c solv.gro -p topol.top -o ions.tpr

genion_mpi -s ions.tpr -o solv_ions.gro -p topol.top -pname NA -nname CL -neutral -conc 0.15

grompp_mpi -f ~/GROrep/minim.mdp -c solv_ions.gro -p topol.top -o em.tpr

mpirun -np 120 /opt/software/GROMACS/5.0.5-intel-2015a-hybrid/bin/mdrun_mpi -deffnm em

grompp_mpi -f ~/GROrep/nvt.mdp -c em.gro -p topol.top -o canon.tpr

mpirun -np 120 /opt/software/GROMACS/5.0.5-intel-2015a-hybrid/bin/mdrun_mpi -deffnm canon

grompp_mpi -f ~/GROrep/npt.mdp -c canon.gro -p topol.top -o isob.tpr

mpirun -np 120 /opt/software/GROMACS/5.0.5-intel-2015a-hybrid/bin/mdrun_mpi -deffnm isob
```

#build fABMACS

```
#git clone https://github.com/BradleyDickson/fABMACS.git
awk '{print $2/10,$3/10,$4/10}' listIndex > Reference
#the +0 or +1 is for if you use the atom # after selecting
awk '{print $1+0}' listIndex > fABMACS/list
tail -1 isob.gro |awk '{print $1/2"\n"$2/2"\n"$3/2}' > sphpoints

module load ifort/2015.1.133-GCC-4.9.2  impi/5.0.2.044
module load GROMACS/5.0.5-hybrid
module load cmake

cd fABMACS
./PATCHscript.sh 480 6  3  3 6 5.5 OVERFILL
cd ..
mkdir fBuild
mkdir fBin
cd fBuild
cmake ../fABMACS -DGMX_BUILD_OWN_FFTW=ON -DGMX_SIMD=AVX2_256 -DGMX_OPENMP=OFF -DGMX_MPI=ON -
DCMAKE_INSTALL_PREFIX=../fBin
make -j24
make install
cd ..
```

#run fABMACS

```
grompp_mpi -f ~/GROrep/md.mdp -c isob.gro -p topol.top -o run1.tpr
```

```
mpirun -np 120 PATHtoFAB/mdrun_mpi -deffnm run1
```

#params

```
300.0
0.9
0.05
10.0
7.17029   9.22826   7.87935
4.6
20.0
0
70.0
```

#sphpoints

```
3.58514
4.61413
3.93967
```

#listIndex (atoms from ULK-101 for collective variables)

```
4432 27.857 38.636 37.254
4433 27.977 39.566 36.254
4434 28.807 39.156 35.224
4447 27.847 47.316 34.714
4448 27.237 46.086 34.904
4450 27.937 48.206 36.184
```

#Reference

```
2.7857 3.8636 3.7254
2.7977 3.9566 3.6254
2.8807 3.9156 3.5224
2.7847 4.7316 3.4714
2.7237 4.6086 3.4904
2.7937 4.8206 3.6184
```

#backs.itp non-mobile atoms to restrain protein in space

```
; position restraints for C-alpha of Protein in water

[ position_restraints ]
;  i funct       fcx        fcy        fcz
1686 1 1000 1000 1000
1687 1 1000 1000 1000
1688 1 1000 1000 1000
1689 1 1000 1000 1000
1690 1 1000 1000 1000
1691 1 1000 1000 1000
1692 1 1000 1000 1000
1693 1 1000 1000 1000
1694 1 1000 1000 1000
1695 1 1000 1000 1000
```

1696	1	1000	1000	1000
1697	1	1000	1000	1000
1698	1	1000	1000	1000
1699	1	1000	1000	1000
1700	1	1000	1000	1000
1701	1	1000	1000	1000
1702	1	1000	1000	1000
1703	1	1000	1000	1000
1704	1	1000	1000	1000
1705	1	1000	1000	1000
1706	1	1000	1000	1000
1707	1	1000	1000	1000
1708	1	1000	1000	1000
1709	1	1000	1000	1000
1710	1	1000	1000	1000
1711	1	1000	1000	1000
1712	1	1000	1000	1000
1713	1	1000	1000	1000
1714	1	1000	1000	1000
1715	1	1000	1000	1000
1716	1	1000	1000	1000
1717	1	1000	1000	1000
1718	1	1000	1000	1000
1719	1	1000	1000	1000
1720	1	1000	1000	1000
1721	1	1000	1000	1000
1722	1	1000	1000	1000
1723	1	1000	1000	1000
1724	1	1000	1000	1000
1725	1	1000	1000	1000
1726	1	1000	1000	1000
1727	1	1000	1000	1000
1728	1	1000	1000	1000
1729	1	1000	1000	1000
1730	1	1000	1000	1000
1731	1	1000	1000	1000
1732	1	1000	1000	1000
1733	1	1000	1000	1000
1734	1	1000	1000	1000
1735	1	1000	1000	1000
1736	1	1000	1000	1000
1737	1	1000	1000	1000
1738	1	1000	1000	1000
1739	1	1000	1000	1000
1740	1	1000	1000	1000
1741	1	1000	1000	1000
1742	1	1000	1000	1000
1743	1	1000	1000	1000
1744	1	1000	1000	1000
1745	1	1000	1000	1000
1746	1	1000	1000	1000
1747	1	1000	1000	1000
1748	1	1000	1000	1000
1749	1	1000	1000	1000
1750	1	1000	1000	1000
1751	1	1000	1000	1000
1752	1	1000	1000	1000
1753	1	1000	1000	1000
1754	1	1000	1000	1000
1755	1	1000	1000	1000
1756	1	1000	1000	1000
1757	1	1000	1000	1000
1758	1	1000	1000	1000
1759	1	1000	1000	1000
1760	1	1000	1000	1000
1761	1	1000	1000	1000
1762	1	1000	1000	1000
1763	1	1000	1000	1000
1764	1	1000	1000	1000
1765	1	1000	1000	1000
1766	1	1000	1000	1000

1767	1	1000	1000	1000
1768	1	1000	1000	1000
1769	1	1000	1000	1000
1770	1	1000	1000	1000
1771	1	1000	1000	1000
1772	1	1000	1000	1000
1773	1	1000	1000	1000
1774	1	1000	1000	1000
1775	1	1000	1000	1000
1776	1	1000	1000	1000
1777	1	1000	1000	1000
1778	1	1000	1000	1000
1779	1	1000	1000	1000
1780	1	1000	1000	1000
1781	1	1000	1000	1000
1782	1	1000	1000	1000
1783	1	1000	1000	1000
1784	1	1000	1000	1000
1785	1	1000	1000	1000
1786	1	1000	1000	1000
1787	1	1000	1000	1000
1788	1	1000	1000	1000
1789	1	1000	1000	1000
1790	1	1000	1000	1000
1791	1	1000	1000	1000
1792	1	1000	1000	1000
1793	1	1000	1000	1000
1794	1	1000	1000	1000
1795	1	1000	1000	1000
1796	1	1000	1000	1000
1797	1	1000	1000	1000
1798	1	1000	1000	1000
1799	1	1000	1000	1000
1800	1	1000	1000	1000
1801	1	1000	1000	1000
1802	1	1000	1000	1000
1803	1	1000	1000	1000
1804	1	1000	1000	1000
1805	1	1000	1000	1000
1806	1	1000	1000	1000
1807	1	1000	1000	1000
1808	1	1000	1000	1000
1809	1	1000	1000	1000
1810	1	1000	1000	1000
1811	1	1000	1000	1000
1812	1	1000	1000	1000
1813	1	1000	1000	1000
1814	1	1000	1000	1000
1815	1	1000	1000	1000
1816	1	1000	1000	1000
1817	1	1000	1000	1000
1818	1	1000	1000	1000
1819	1	1000	1000	1000
1820	1	1000	1000	1000
1821	1	1000	1000	1000
1822	1	1000	1000	1000
1823	1	1000	1000	1000
1824	1	1000	1000	1000
1825	1	1000	1000	1000
1826	1	1000	1000	1000
1827	1	1000	1000	1000
1828	1	1000	1000	1000
1829	1	1000	1000	1000
1830	1	1000	1000	1000
1831	1	1000	1000	1000
1832	1	1000	1000	1000
1833	1	1000	1000	1000
1834	1	1000	1000	1000
1835	1	1000	1000	1000
1836	1	1000	1000	1000
1837	1	1000	1000	1000

1838	1	1000	1000	1000
1839	1	1000	1000	1000
1840	1	1000	1000	1000
1841	1	1000	1000	1000
1842	1	1000	1000	1000
1843	1	1000	1000	1000
1844	1	1000	1000	1000
1845	1	1000	1000	1000
1846	1	1000	1000	1000
1847	1	1000	1000	1000
1848	1	1000	1000	1000
1849	1	1000	1000	1000
1850	1	1000	1000	1000
1851	1	1000	1000	1000
1852	1	1000	1000	1000
1853	1	1000	1000	1000
1854	1	1000	1000	1000
1855	1	1000	1000	1000
1856	1	1000	1000	1000
1857	1	1000	1000	1000
1858	1	1000	1000	1000
1859	1	1000	1000	1000
1860	1	1000	1000	1000
1861	1	1000	1000	1000
1862	1	1000	1000	1000
1863	1	1000	1000	1000
1864	1	1000	1000	1000
1865	1	1000	1000	1000
1866	1	1000	1000	1000
1867	1	1000	1000	1000
1868	1	1000	1000	1000
1869	1	1000	1000	1000
1870	1	1000	1000	1000
1871	1	1000	1000	1000
1872	1	1000	1000	1000
1873	1	1000	1000	1000
1874	1	1000	1000	1000
1875	1	1000	1000	1000
1876	1	1000	1000	1000
1877	1	1000	1000	1000
1878	1	1000	1000	1000
1879	1	1000	1000	1000
1880	1	1000	1000	1000
1881	1	1000	1000	1000
1882	1	1000	1000	1000
1883	1	1000	1000	1000
1884	1	1000	1000	1000
1885	1	1000	1000	1000
1886	1	1000	1000	1000
1887	1	1000	1000	1000
1888	1	1000	1000	1000
1889	1	1000	1000	1000
1890	1	1000	1000	1000
1891	1	1000	1000	1000
1892	1	1000	1000	1000
1893	1	1000	1000	1000
1894	1	1000	1000	1000
1895	1	1000	1000	1000
1896	1	1000	1000	1000
1897	1	1000	1000	1000
1898	1	1000	1000	1000
1899	1	1000	1000	1000
1900	1	1000	1000	1000
1901	1	1000	1000	1000
1902	1	1000	1000	1000
1903	1	1000	1000	1000
1904	1	1000	1000	1000
1905	1	1000	1000	1000
1906	1	1000	1000	1000
1907	1	1000	1000	1000
1908	1	1000	1000	1000

1909 1 1000 1000 1000
1910 1 1000 1000 1000
1911 1 1000 1000 1000
1912 1 1000 1000 1000
1913 1 1000 1000 1000
1914 1 1000 1000 1000
1915 1 1000 1000 1000
1916 1 1000 1000 1000
1917 1 1000 1000 1000
1918 1 1000 1000 1000
1919 1 1000 1000 1000
1920 1 1000 1000 1000
1921 1 1000 1000 1000
1922 1 1000 1000 1000
1923 1 1000 1000 1000
1924 1 1000 1000 1000
4000 1 1000 1000 1000
4001 1 1000 1000 1000
4002 1 1000 1000 1000
4003 1 1000 1000 1000
4004 1 1000 1000 1000
4005 1 1000 1000 1000
4006 1 1000 1000 1000
4007 1 1000 1000 1000
4008 1 1000 1000 1000
4009 1 1000 1000 1000
4010 1 1000 1000 1000
4011 1 1000 1000 1000
4012 1 1000 1000 1000
4013 1 1000 1000 1000
4014 1 1000 1000 1000
4015 1 1000 1000 1000
4016 1 1000 1000 1000
4017 1 1000 1000 1000
4018 1 1000 1000 1000
4019 1 1000 1000 1000
4020 1 1000 1000 1000
4021 1 1000 1000 1000
4022 1 1000 1000 1000
4023 1 1000 1000 1000
4024 1 1000 1000 1000
4025 1 1000 1000 1000
4026 1 1000 1000 1000
4027 1 1000 1000 1000
4028 1 1000 1000 1000
4029 1 1000 1000 1000
4030 1 1000 1000 1000
4031 1 1000 1000 1000
4032 1 1000 1000 1000
4033 1 1000 1000 1000
4034 1 1000 1000 1000
4035 1 1000 1000 1000
4036 1 1000 1000 1000
4037 1 1000 1000 1000
4038 1 1000 1000 1000
4039 1 1000 1000 1000
4040 1 1000 1000 1000
4041 1 1000 1000 1000
4042 1 1000 1000 1000
4043 1 1000 1000 1000
4044 1 1000 1000 1000
4045 1 1000 1000 1000
4046 1 1000 1000 1000
4047 1 1000 1000 1000
4048 1 1000 1000 1000
4049 1 1000 1000 1000
4050 1 1000 1000 1000
4051 1 1000 1000 1000
4052 1 1000 1000 1000
4053 1 1000 1000 1000
4054 1 1000 1000 1000

4055 1 1000 1000 1000
4056 1 1000 1000 1000
4057 1 1000 1000 1000
4058 1 1000 1000 1000
4059 1 1000 1000 1000
4060 1 1000 1000 1000
4061 1 1000 1000 1000
4062 1 1000 1000 1000
4063 1 1000 1000 1000
4064 1 1000 1000 1000
4065 1 1000 1000 1000
4066 1 1000 1000 1000
4067 1 1000 1000 1000
4068 1 1000 1000 1000
4069 1 1000 1000 1000
4070 1 1000 1000 1000
4071 1 1000 1000 1000
4072 1 1000 1000 1000
4073 1 1000 1000 1000
4074 1 1000 1000 1000
4075 1 1000 1000 1000
4076 1 1000 1000 1000
4077 1 1000 1000 1000
4078 1 1000 1000 1000
4079 1 1000 1000 1000
4080 1 1000 1000 1000
4081 1 1000 1000 1000
4082 1 1000 1000 1000
4083 1 1000 1000 1000
4084 1 1000 1000 1000
4085 1 1000 1000 1000
4086 1 1000 1000 1000
4087 1 1000 1000 1000
4088 1 1000 1000 1000
4089 1 1000 1000 1000
4090 1 1000 1000 1000
4091 1 1000 1000 1000
4092 1 1000 1000 1000
4093 1 1000 1000 1000
4094 1 1000 1000 1000
4095 1 1000 1000 1000
4096 1 1000 1000 1000
4097 1 1000 1000 1000
4098 1 1000 1000 1000
4099 1 1000 1000 1000
4100 1 1000 1000 1000
4101 1 1000 1000 1000
4102 1 1000 1000 1000
4103 1 1000 1000 1000
4104 1 1000 1000 1000
4105 1 1000 1000 1000
4106 1 1000 1000 1000
4107 1 1000 1000 1000
4108 1 1000 1000 1000
4109 1 1000 1000 1000
4110 1 1000 1000 1000
4111 1 1000 1000 1000
4112 1 1000 1000 1000
4113 1 1000 1000 1000
4114 1 1000 1000 1000
4115 1 1000 1000 1000
4116 1 1000 1000 1000
4117 1 1000 1000 1000
4118 1 1000 1000 1000

#proc.pdb

```
TITLE     Gromacs Runs On Most of All Computer Systems
MODEL        1
ATOM      1  N   SER A   1     -20.284 -17.510   3.277  1.00  0.00           N
ATOM      2  H1  SER A   1     -20.993 -17.913   2.699  1.00  0.00
ATOM      3  H2  SER A   1     -19.385 -17.815   2.963  1.00  0.00
ATOM      4  CA  SER A   1     -20.362 -16.005   3.194  1.00  0.00           C
ATOM      5  HA  SER A   1     -20.213 -15.746   2.240  1.00  0.00
ATOM      6  CB  SER A   1     -21.733 -15.502   3.574  1.00  0.00           C
ATOM      7  HB1 SER A   1     -22.418 -15.884   2.953  1.00  0.00
ATOM      8  HB2 SER A   1     -21.945 -15.774   4.513  1.00  0.00
ATOM      9  OG  SER A   1     -21.832 -14.073   3.503  1.00  0.00           O
ATOM     10  HG1 SER A   1     -22.755 -13.790   3.763  1.00  0.00
ATOM     11  C   SER A   1     -19.279 -15.369   4.077  1.00  0.00           C
ATOM     12  O   SER A   1     -19.298 -15.510   5.318  1.00  0.00           O
ATOM     13  N   MET A   2     -18.330 -14.694   3.425  1.00  0.00           N
ATOM     14  HN  MET A   2     -18.340 -14.708   2.425  1.00  0.00
ATOM     15  CA  MET A   2     -17.276 -13.935   4.099  1.00  0.00           C
ATOM     16  HA  MET A   2     -17.342 -14.150   5.073  1.00  0.00
ATOM     17  CB  MET A   2     -15.885 -14.334   3.597  1.00  0.00           C
ATOM     18  HB1 MET A   2     -15.851 -14.202   2.606  1.00  0.00
ATOM     19  HB2 MET A   2     -15.205 -13.746   4.035  1.00  0.00
ATOM     20  CG  MET A   2     -15.519 -15.765   3.888  1.00  0.00           C
ATOM     21  HG1 MET A   2     -16.395 -16.236   3.995  1.00  0.00
ATOM     22  HG2 MET A   2     -15.067 -16.087   3.056  1.00  0.00
ATOM     23  SD  MET A   2     -14.473 -15.924   5.341  1.00  0.00           S
ATOM     24  CE  MET A   2     -12.900 -16.397   4.617  1.00  0.00           C
ATOM     25  HE1 MET A   2     -12.222 -16.517   5.342  1.00  0.00
ATOM     26  HE2 MET A   2     -13.008 -17.256   4.116  1.00  0.00
ATOM     27  HE3 MET A   2     -12.593 -15.681   3.989  1.00  0.00
ATOM     28  C   MET A   2     -17.514 -12.452   3.804  1.00  0.00           C
ATOM     29  O   MET A   2     -17.953 -12.094   2.718  1.00  0.00           O
ATOM     30  N   GLU A   3     -17.255 -11.606   4.804  1.00  0.00           N
ATOM     31  HN  GLU A   3     -17.027 -11.990   5.699  1.00  0.00
ATOM     32  CA  GLU A   3     -17.285 -10.153   4.667  1.00  0.00           C
ATOM     33  HA  GLU A   3     -18.009  -9.937   4.012  1.00  0.00
ATOM     34  CB  GLU A   3     -17.616  -9.482   6.003  1.00  0.00           C
ATOM     35  HB1 GLU A   3     -17.334 -10.095   6.741  1.00  0.00
ATOM     36  HB2 GLU A   3     -17.099  -8.628   6.063  1.00  0.00
ATOM     37  CG  GLU A   3     -19.092  -9.157   6.194  1.00  0.00           C
ATOM     38  HG1 GLU A   3     -19.619  -9.980   5.979  1.00  0.00
ATOM     39  HG2 GLU A   3     -19.232  -8.913   7.154  1.00  0.00
ATOM     40  CD  GLU A   3     -19.625  -8.013   5.331  1.00  0.00           C
ATOM     41  OE1 GLU A   3     -18.821  -7.265   4.691  1.00  0.00           O
ATOM     42  OE2 GLU A   3     -20.848  -7.841   5.327  1.00  0.00           O
ATOM     43  C   GLU A   3     -15.915  -9.690   4.160  1.00  0.00           C
ATOM     44  O   GLU A   3     -14.887 -10.183   4.575  1.00  0.00           O
ATOM     45  N   VAL A   4     -15.934  -8.747   3.235  1.00  0.00           N
ATOM     46  HN  VAL A   4     -16.809  -8.321   3.004  1.00  0.00
ATOM     47  CA  VAL A   4     -14.767  -8.313   2.555  1.00  0.00           C
ATOM     48  HA  VAL A   4     -14.056  -8.984   2.764  1.00  0.00
ATOM     49  CB  VAL A   4     -15.011  -8.300   1.039  1.00  0.00           C
ATOM     50  HB  VAL A   4     -15.890  -7.856   0.868  1.00  0.00
ATOM     51  CG1 VAL A   4     -13.969  -7.489   0.296  1.00  0.00           C
ATOM     52 1HG1 VAL A   4     -14.168  -7.509  -0.684  1.00  0.00
ATOM     53 2HG1 VAL A   4     -13.989  -6.543   0.621  1.00  0.00
ATOM     54 3HG1 VAL A   4     -13.063  -7.879   0.461  1.00  0.00
ATOM     55  CG2 VAL A   4     -15.075  -9.715   0.504  1.00  0.00           C
ATOM     56 1HG2 VAL A   4     -15.234  -9.691  -0.483  1.00  0.00
ATOM     57 2HG2 VAL A   4     -14.211 -10.182   0.691  1.00  0.00
ATOM     58 3HG2 VAL A   4     -15.823 -10.207   0.950  1.00  0.00
ATOM     59  C   VAL A   4     -14.364  -6.940   3.095  1.00  0.00           C
ATOM     60  O   VAL A   4     -15.195  -6.071   3.270  1.00  0.00           O
ATOM     61  N   VAL A   5     -13.069  -6.790   3.357  1.00  0.00           N
ATOM     62  HN  VAL A   5     -12.472  -7.583   3.237  1.00  0.00
ATOM     63  CA  VAL A   5     -12.471  -5.554   3.804  1.00  0.00           C
ATOM     64  HA  VAL A   5     -13.141  -4.833   3.625  1.00  0.00
ATOM     65  CB  VAL A   5     -12.171  -5.608   5.309  1.00  0.00           C
ATOM     66  HB  VAL A   5     -11.519  -6.350   5.465  1.00  0.00
```

ATOM	67	CG1	VAL	A	5	-11.521	-4.319	5.797	1.00	0.00	C
ATOM	68	1HG1	VAL	A	5	-11.340	-4.388	6.778	1.00	0.00	
ATOM	69	2HG1	VAL	A	5	-10.661	-4.174	5.308	1.00	0.00	
ATOM	70	3HG1	VAL	A	5	-12.136	-3.550	5.625	1.00	0.00	
ATOM	71	CG2	VAL	A	5	-13.412	-5.936	6.112	1.00	0.00	C
ATOM	72	1HG2	VAL	A	5	-13.182	-5.963	7.085	1.00	0.00	
ATOM	73	2HG2	VAL	A	5	-14.107	-5.235	5.953	1.00	0.00	
ATOM	74	3HG2	VAL	A	5	-13.767	-6.827	5.828	1.00	0.00	
ATOM	75	C	VAL	A	5	-11.184	-5.329	3.010	1.00	0.00	C
ATOM	76	O	VAL	A	5	-10.147	-5.922	3.329	1.00	0.00	O
ATOM	77	N	GLY	A	6	-11.257	-4.468	1.985	1.00	0.00	N
ATOM	78	HN	GLY	A	6	-12.072	-3.896	1.891	1.00	0.00	
ATOM	79	CA	GLY	A	6	-10.195	-4.339	1.009	1.00	0.00	C
ATOM	80	HA1	GLY	A	6	-10.542	-3.803	0.239	1.00	0.00	
ATOM	81	HA2	GLY	A	6	-9.434	-3.852	1.438	1.00	0.00	
ATOM	82	C	GLY	A	6	-9.739	-5.709	0.532	1.00	0.00	C
ATOM	83	O	GLY	A	6	-10.550	-6.512	0.085	1.00	0.00	O
ATOM	84	N	ASP	A	7	-8.433	-5.989	0.679	1.00	0.00	N
ATOM	85	HN	ASP	A	7	-7.850	-5.324	1.145	1.00	0.00	
ATOM	86	CA	ASP	A	7	-7.827	-7.231	0.184	1.00	0.00	C
ATOM	87	HA	ASP	A	7	-8.347	-7.450	-0.642	1.00	0.00	
ATOM	88	CB	ASP	A	7	-6.362	-7.026	-0.171	1.00	0.00	C
ATOM	89	HB1	ASP	A	7	-5.887	-6.684	0.640	1.00	0.00	
ATOM	90	HB2	ASP	A	7	-5.976	-7.910	-0.436	1.00	0.00	
ATOM	91	CG	ASP	A	7	-6.130	-6.044	-1.309	1.00	0.00	C
ATOM	92	OD1	ASP	A	7	-6.959	-5.992	-2.235	1.00	0.00	O
ATOM	93	OD2	ASP	A	7	-5.103	-5.362	-1.276	1.00	0.00	O
ATOM	94	C	ASP	A	7	-7.952	-8.369	1.211	1.00	0.00	C
ATOM	95	O	ASP	A	7	-7.379	-9.446	1.002	1.00	0.00	O
ATOM	96	N	PHE	A	8	-8.701	-8.135	2.301	1.00	0.00	N
ATOM	97	HN	PHE	A	8	-9.175	-7.257	2.371	1.00	0.00	
ATOM	98	CA	PHE	A	8	-8.858	-9.094	3.387	1.00	0.00	C
ATOM	99	HA	PHE	A	8	-8.290	-9.884	3.158	1.00	0.00	
ATOM	100	CB	PHE	A	8	-8.374	-8.495	4.704	1.00	0.00	C
ATOM	101	HB1	PHE	A	8	-8.949	-7.708	4.928	1.00	0.00	
ATOM	102	HB2	PHE	A	8	-8.462	-9.186	5.421	1.00	0.00	
ATOM	103	CG	PHE	A	8	-6.939	-8.034	4.669	1.00	0.00	C
ATOM	104	CD1	PHE	A	8	-6.614	-6.761	4.209	1.00	0.00	C
ATOM	105	HD1	PHE	A	8	-7.340	-6.142	3.910	1.00	0.00	
ATOM	106	CE1	PHE	A	8	-5.294	-6.345	4.162	1.00	0.00	C
ATOM	107	HE1	PHE	A	8	-5.074	-5.425	3.836	1.00	0.00	
ATOM	108	CZ	PHE	A	8	-4.277	-7.194	4.563	1.00	0.00	C
ATOM	109	HZ	PHE	A	8	-3.325	-6.891	4.524	1.00	0.00	
ATOM	110	CD2	PHE	A	8	-5.908	-8.877	5.074	1.00	0.00	C
ATOM	111	HD2	PHE	A	8	-6.122	-9.794	5.410	1.00	0.00	
ATOM	112	CE2	PHE	A	8	-4.583	-8.456	5.016	1.00	0.00	C
ATOM	113	HE2	PHE	A	8	-3.851	-9.073	5.305	1.00	0.00	
ATOM	114	C	PHE	A	8	-10.324	-9.504	3.489	1.00	0.00	C
ATOM	115	O	PHE	A	8	-11.188	-8.849	2.922	1.00	0.00	O
ATOM	116	N	GLU	A	9	-10.585	-10.589	4.220	1.00	0.00	N
ATOM	117	HN	GLU	A	9	-9.821	-11.082	4.636	1.00	0.00	
ATOM	118	CA	GLU	A	9	-11.932	-11.083	4.437	1.00	0.00	C
ATOM	119	HA	GLU	A	9	-12.543	-10.292	4.398	1.00	0.00	
ATOM	120	CB	GLU	A	9	-12.322	-12.056	3.329	1.00	0.00	C
ATOM	121	HB1	GLU	A	9	-13.274	-12.331	3.466	1.00	0.00	
ATOM	122	HB2	GLU	A	9	-12.233	-11.590	2.449	1.00	0.00	
ATOM	123	CG	GLU	A	9	-11.470	-13.305	3.291	1.00	0.00	C
ATOM	124	HG1	GLU	A	9	-10.516	-13.017	3.214	1.00	0.00	
ATOM	125	HG2	GLU	A	9	-11.604	-13.788	4.157	1.00	0.00	
ATOM	126	CD	GLU	A	9	-11.763	-14.283	2.153	1.00	0.00	C
ATOM	127	OE1	GLU	A	9	-12.826	-14.149	1.491	1.00	0.00	O
ATOM	128	OE2	GLU	A	9	-10.913	-15.193	1.922	1.00	0.00	O
ATOM	129	C	GLU	A	9	-12.011	-11.740	5.818	1.00	0.00	C
ATOM	130	O	GLU	A	9	-10.995	-12.099	6.390	1.00	0.00	O
ATOM	131	N	TYR	A	10	-13.231	-11.876	6.340	1.00	0.00	N
ATOM	132	HN	TYR	A	10	-14.013	-11.515	5.832	1.00	0.00	
ATOM	133	CA	TYR	A	10	-13.470	-12.524	7.611	1.00	0.00	C
ATOM	134	HA	TYR	A	10	-12.805	-13.270	7.641	1.00	0.00	
ATOM	135	CB	TYR	A	10	-13.243	-11.560	8.782	1.00	0.00	C
ATOM	136	HB1	TYR	A	10	-13.213	-12.092	9.628	1.00	0.00	
ATOM	137	HB2	TYR	A	10	-12.366	-11.100	8.647	1.00	0.00	

ATOM	138	CG	TYR	A	10	-14.300	-10.494	8.947	1.00	0.00	C
ATOM	139	CD1	TYR	A	10	-14.243	-9.353	8.189	1.00	0.00	C
ATOM	140	HD1	TYR	A	10	-13.500	-9.245	7.529	1.00	0.00	
ATOM	141	CE1	TYR	A	10	-15.182	-8.348	8.311	1.00	0.00	C
ATOM	142	HE1	TYR	A	10	-15.114	-7.530	7.739	1.00	0.00	
ATOM	143	CZ	TYR	A	10	-16.206	-8.467	9.212	1.00	0.00	C
ATOM	144	OH	TYR	A	10	-17.091	-7.427	9.282	1.00	0.00	O
ATOM	145	HH	TYR	A	10	-17.788	-7.628	9.970	1.00	0.00	
ATOM	146	CD2	TYR	A	10	-15.338	-10.613	9.856	1.00	0.00	C
ATOM	147	HD2	TYR	A	10	-15.405	-11.435	10.422	1.00	0.00	
ATOM	148	CE2	TYR	A	10	-16.287	-9.609	9.993	1.00	0.00	C
ATOM	149	HE2	TYR	A	10	-17.029	-9.710	10.655	1.00	0.00	
ATOM	150	C	TYR	A	10	-14.904	-13.051	7.667	1.00	0.00	C
ATOM	151	O	TYR	A	10	-15.774	-12.608	6.950	1.00	0.00	O
ATOM	152	N	SER	A	11	-15.102	-14.016	8.557	1.00	0.00	N
ATOM	153	HN	SER	A	11	-14.296	-14.388	9.018	1.00	0.00	
ATOM	154	CA	SER	A	11	-16.375	-14.568	8.911	1.00	0.00	C
ATOM	155	HA	SER	A	11	-17.031	-14.298	8.206	1.00	0.00	
ATOM	156	CB	SER	A	11	-16.273	-16.067	8.925	1.00	0.00	C
ATOM	157	HB1	SER	A	11	-16.304	-16.427	7.992	1.00	0.00	
ATOM	158	HB2	SER	A	11	-15.422	-16.354	9.365	1.00	0.00	
ATOM	159	OG	SER	A	11	-17.347	-16.639	9.645	1.00	0.00	O
ATOM	160	HG1	SER	A	11	-17.258	-17.635	9.641	1.00	0.00	
ATOM	161	C	SER	A	11	-16.790	-14.017	10.283	1.00	0.00	C
ATOM	162	O	SER	A	11	-15.965	-13.919	11.191	1.00	0.00	O
ATOM	163	N	LYS	A	12	-18.076	-13.681	10.422	1.00	0.00	N
ATOM	164	HN	LYS	A	12	-18.689	-13.821	9.644	1.00	0.00	
ATOM	165	CA	LYS	A	12	-18.636	-13.118	11.654	1.00	0.00	C
ATOM	166	HA	LYS	A	12	-18.038	-12.364	11.926	1.00	0.00	
ATOM	167	CB	LYS	A	12	-20.031	-12.566	11.385	1.00	0.00	C
ATOM	168	HB1	LYS	A	12	-20.582	-13.284	10.959	1.00	0.00	
ATOM	169	HB2	LYS	A	12	-20.444	-12.302	12.257	1.00	0.00	
ATOM	170	CG	LYS	A	12	-20.025	-11.354	10.467	1.00	0.00	C
ATOM	171	HG1	LYS	A	12	-19.354	-10.699	10.815	1.00	0.00	
ATOM	172	HG2	LYS	A	12	-19.754	-11.654	9.552	1.00	0.00	
ATOM	173	CD	LYS	A	12	-21.354	-10.645	10.352	1.00	0.00	C
ATOM	174	HD1	LYS	A	12	-21.942	-11.168	9.735	1.00	0.00	
ATOM	175	HD2	LYS	A	12	-21.774	-10.605	11.259	1.00	0.00	
ATOM	176	CE	LYS	A	12	-21.214	-9.233	9.815	1.00	0.00	C
ATOM	177	HE1	LYS	A	12	-20.979	-8.616	10.566	1.00	0.00	
ATOM	178	HE2	LYS	A	12	-20.489	-9.212	9.127	1.00	0.00	
ATOM	179	NZ	LYS	A	12	-22.472	-8.751	9.187	1.00	0.00	N
ATOM	180	HZ1	LYS	A	12	-22.339	-7.820	8.847	1.00	0.00	
ATOM	181	HZ2	LYS	A	12	-22.716	-9.352	8.426	1.00	0.00	
ATOM	182	HZ3	LYS	A	12	-23.207	-8.756	9.865	1.00	0.00	
ATOM	183	C	LYS	A	12	-18.639	-14.164	12.773	1.00	0.00	C
ATOM	184	O	LYS	A	12	-18.708	-13.800	13.937	1.00	0.00	O
ATOM	185	N	ARG	A	13	-18.506	-15.447	12.408	1.00	0.00	N
ATOM	186	HN	ARG	A	13	-18.493	-15.670	11.433	1.00	0.00	
ATOM	187	CA	ARG	A	13	-18.379	-16.530	13.374	1.00	0.00	C
ATOM	188	HA	ARG	A	13	-19.189	-16.433	13.953	1.00	0.00	
ATOM	189	CB	ARG	A	13	-18.348	-17.903	12.685	1.00	0.00	C
ATOM	190	HB1	ARG	A	13	-17.696	-17.856	11.929	1.00	0.00	
ATOM	191	HB2	ARG	A	13	-18.035	-18.578	13.353	1.00	0.00	
ATOM	192	CG	ARG	A	13	-19.672	-18.395	12.120	1.00	0.00	C
ATOM	193	HG1	ARG	A	13	-20.387	-18.270	12.808	1.00	0.00	
ATOM	194	HG2	ARG	A	13	-19.897	-17.862	11.304	1.00	0.00	
ATOM	195	CD	ARG	A	13	-19.584	-19.875	11.747	1.00	0.00	C
ATOM	196	HD1	ARG	A	13	-20.384	-20.126	11.201	1.00	0.00	
ATOM	197	HD2	ARG	A	13	-18.753	-20.032	11.213	1.00	0.00	
ATOM	198	NE	ARG	A	13	-19.540	-20.778	12.902	1.00	0.00	N
ATOM	199	HE	ARG	A	13	-20.130	-20.557	13.679	1.00	0.00	
ATOM	200	CZ	ARG	A	13	-18.777	-21.874	13.005	1.00	0.00	C
ATOM	201	NH1	ARG	A	13	-17.872	-22.156	12.081	1.00	0.00	N
ATOM	202	1HH1	ARG	A	13	-17.753	-21.548	11.296	1.00	0.00	
ATOM	203	2HH1	ARG	A	13	-17.308	-22.977	12.168	1.00	0.00	
ATOM	204	NH2	ARG	A	13	-18.909	-22.670	14.048	1.00	0.00	N
ATOM	205	1HH2	ARG	A	13	-19.578	-22.455	14.760	1.00	0.00	
ATOM	206	2HH2	ARG	A	13	-18.340	-23.489	14.127	1.00	0.00	
ATOM	207	C	ARG	A	13	-17.075	-16.384	14.166	1.00	0.00	C
ATOM	208	O	ARG	A	13	-17.006	-16.795	15.319	1.00	0.00	O

ATOM	209	N	ASP	A	14	-16.037	-15.843	13.522	1.00	0.00	N
ATOM	210	HN	ASP	A	14	-16.198	-15.397	12.641	1.00	0.00	
ATOM	211	CA	ASP	A	14	-14.679	-15.876	14.051	1.00	0.00	C
ATOM	212	HA	ASP	A	14	-14.626	-16.683	14.639	1.00	0.00	
ATOM	213	CB	ASP	A	14	-13.651	-16.043	12.930	1.00	0.00	C
ATOM	214	HB1	ASP	A	14	-13.810	-15.332	12.245	1.00	0.00	
ATOM	215	HB2	ASP	A	14	-12.736	-15.932	13.319	1.00	0.00	
ATOM	216	CG	ASP	A	14	-13.705	-17.386	12.234	1.00	0.00	C
ATOM	217	OD1	ASP	A	14	-13.731	-18.405	12.952	1.00	0.00	O
ATOM	218	OD2	ASP	A	14	-13.739	-17.392	10.961	1.00	0.00	O
ATOM	219	C	ASP	A	14	-14.423	-14.611	14.876	1.00	0.00	C
ATOM	220	O	ASP	A	14	-13.489	-13.866	14.617	1.00	0.00	O
ATOM	221	N	LEU	A	15	-15.262	-14.414	15.897	1.00	0.00	N
ATOM	222	HN	LEU	A	15	-15.983	-15.091	16.046	1.00	0.00	
ATOM	223	CA	LEU	A	15	-15.197	-13.283	16.804	1.00	0.00	C
ATOM	224	HA	LEU	A	15	-15.033	-12.453	16.272	1.00	0.00	
ATOM	225	CB	LEU	A	15	-16.541	-13.202	17.532	1.00	0.00	C
ATOM	226	HB1	LEU	A	15	-17.261	-13.144	16.841	1.00	0.00	
ATOM	227	HB2	LEU	A	15	-16.654	-14.043	18.062	1.00	0.00	
ATOM	228	CG	LEU	A	15	-16.722	-12.028	18.486	1.00	0.00	C
ATOM	229	HG	LEU	A	15	-15.982	-12.055	19.158	1.00	0.00	
ATOM	230	CD1	LEU	A	15	-16.645	-10.709	17.738	1.00	0.00	C
ATOM	231	1HD1	LEU	A	15	-16.766	-9.953	18.382	1.00	0.00	
ATOM	232	2HD1	LEU	A	15	-15.752	-10.629	17.294	1.00	0.00	
ATOM	233	3HD1	LEU	A	15	-17.366	-10.676	17.046	1.00	0.00	
ATOM	234	CD2	LEU	A	15	-18.059	-12.133	19.209	1.00	0.00	C
ATOM	235	1HD2	LEU	A	15	-18.164	-11.357	19.831	1.00	0.00	
ATOM	236	2HD2	LEU	A	15	-18.802	-12.126	18.539	1.00	0.00	
ATOM	237	3HD2	LEU	A	15	-18.089	-12.985	19.732	1.00	0.00	
ATOM	238	C	LEU	A	15	-14.035	-13.484	17.785	1.00	0.00	C
ATOM	239	O	LEU	A	15	-13.827	-14.577	18.267	1.00	0.00	O
ATOM	240	N	VAL	A	16	-13.252	-12.427	18.020	1.00	0.00	N
ATOM	241	HN	VAL	A	16	-13.438	-11.573	17.534	1.00	0.00	
ATOM	242	CA	VAL	A	16	-12.132	-12.471	18.962	1.00	0.00	C
ATOM	243	HA	VAL	A	16	-11.916	-13.438	19.094	1.00	0.00	
ATOM	244	CB	VAL	A	16	-10.873	-11.786	18.400	1.00	0.00	C
ATOM	245	HB	VAL	A	16	-11.060	-10.804	18.359	1.00	0.00	
ATOM	246	CG1	VAL	A	16	-9.669	-12.017	19.295	1.00	0.00	C
ATOM	247	1HG1	VAL	A	16	-8.870	-11.561	18.903	1.00	0.00	
ATOM	248	2HG1	VAL	A	16	-9.853	-11.644	20.204	1.00	0.00	
ATOM	249	3HG1	VAL	A	16	-9.490	-12.998	19.367	1.00	0.00	
ATOM	250	CG2	VAL	A	16	-10.556	-12.246	17.000	1.00	0.00	C
ATOM	251	1HG2	VAL	A	16	-9.734	-11.779	16.674	1.00	0.00	
ATOM	252	2HG2	VAL	A	16	-10.400	-13.234	17.001	1.00	0.00	
ATOM	253	3HG2	VAL	A	16	-11.324	-12.031	16.397	1.00	0.00	
ATOM	254	C	VAL	A	16	-12.578	-11.826	20.278	1.00	0.00	C
ATOM	255	O	VAL	A	16	-12.377	-12.394	21.344	1.00	0.00	O
ATOM	256	N	GLY	A	17	-13.193	-10.643	20.184	1.00	0.00	N
ATOM	257	HN	GLY	A	17	-13.283	-10.220	19.283	1.00	0.00	
ATOM	258	CA	GLY	A	17	-13.737	-9.946	21.340	1.00	0.00	C
ATOM	259	HA1	GLY	A	17	-14.499	-10.486	21.697	1.00	0.00	
ATOM	260	HA2	GLY	A	17	-13.018	-9.886	22.032	1.00	0.00	
ATOM	261	C	GLY	A	17	-14.218	-8.553	20.973	1.00	0.00	C
ATOM	262	O	GLY	A	17	-14.445	-8.258	19.780	1.00	0.00	O
ATOM	263	N	HIS	A	18	-14.385	-7.704	21.991	1.00	0.00	N
ATOM	264	HN	HIS	A	18	-14.113	-7.996	22.908	1.00	0.00	
ATOM	265	CA	HIS	A	18	-14.947	-6.370	21.821	1.00	0.00	C
ATOM	266	HA	HIS	A	18	-15.003	-6.217	20.834	1.00	0.00	
ATOM	267	CB	HIS	A	18	-16.362	-6.289	22.423	1.00	0.00	C
ATOM	268	HB1	HIS	A	18	-16.291	-6.324	23.420	1.00	0.00	
ATOM	269	HB2	HIS	A	18	-16.780	-5.423	22.149	1.00	0.00	
ATOM	270	ND1	HIS	A	18	-17.449	-8.548	22.751	1.00	0.00	N
ATOM	271	CG	HIS	A	18	-17.287	-7.381	22.005	1.00	0.00	C
ATOM	272	CE1	HIS	A	18	-18.330	-9.327	22.155	1.00	0.00	C
ATOM	273	HE1	HIS	A	18	-18.628	-10.227	22.473	1.00	0.00	
ATOM	274	NE2	HIS	A	18	-18.757	-8.701	21.046	1.00	0.00	N
ATOM	275	HE2	HIS	A	18	-19.431	-9.058	20.399	1.00	0.00	
ATOM	276	CD2	HIS	A	18	-18.116	-7.489	20.945	1.00	0.00	C
ATOM	277	HD2	HIS	A	18	-18.241	-6.812	20.220	1.00	0.00	
ATOM	278	C	HIS	A	18	-14.014	-5.337	22.467	1.00	0.00	C
ATOM	279	O	HIS	A	18	-13.277	-5.645	23.398	1.00	0.00	O

ATOM	280	N	GLY	A	19	-14.052	-4.106	21.942	1.00	0.00	N
ATOM	281	HN	GLY	A	19	-14.322	-4.006	20.984	1.00	0.00	
ATOM	282	CA	GLY	A	19	-13.721	-2.907	22.694	1.00	0.00	C
ATOM	283	HA1	GLY	A	19	-13.163	-3.171	23.480	1.00	0.00	
ATOM	284	HA2	GLY	A	19	-13.195	-2.297	22.101	1.00	0.00	
ATOM	285	C	GLY	A	19	-14.992	-2.218	23.160	1.00	0.00	C
ATOM	286	O	GLY	A	19	-16.086	-2.781	23.080	1.00	0.00	O
ATOM	287	N	ALA	A	20	-14.863	-0.974	23.602	1.00	0.00	N
ATOM	288	HN	ALA	A	20	-13.949	-0.581	23.700	1.00	0.00	
ATOM	289	CA	ALA	A	20	-16.024	-0.173	23.946	1.00	0.00	C
ATOM	290	HA	ALA	A	20	-16.594	-0.726	24.554	1.00	0.00	
ATOM	291	CB	ALA	A	20	-15.602	1.060	24.712	1.00	0.00	C
ATOM	292	HB1	ALA	A	20	-16.410	1.602	24.942	1.00	0.00	
ATOM	293	HB2	ALA	A	20	-15.133	0.786	25.552	1.00	0.00	
ATOM	294	HB3	ALA	A	20	-14.983	1.607	24.148	1.00	0.00	
ATOM	295	C	ALA	A	20	-16.797	0.181	22.660	1.00	0.00	C
ATOM	296	O	ALA	A	20	-18.023	0.232	22.660	1.00	0.00	O
ATOM	297	N	PHE	A	21	-16.060	0.406	21.562	1.00	0.00	N
ATOM	298	HN	PHE	A	21	-15.082	0.200	21.593	1.00	0.00	
ATOM	299	CA	PHE	A	21	-16.627	0.942	20.314	1.00	0.00	C
ATOM	300	HA	PHE	A	21	-17.615	1.003	20.453	1.00	0.00	
ATOM	301	CB	PHE	A	21	-16.045	2.327	20.056	1.00	0.00	C
ATOM	302	HB1	PHE	A	21	-15.047	2.275	20.102	1.00	0.00	
ATOM	303	HB2	PHE	A	21	-16.322	2.632	19.145	1.00	0.00	
ATOM	304	CG	PHE	A	21	-16.514	3.344	21.060	1.00	0.00	C
ATOM	305	CD1	PHE	A	21	-17.762	3.924	20.936	1.00	0.00	C
ATOM	306	HD1	PHE	A	21	-18.350	3.671	20.168	1.00	0.00	
ATOM	307	CE1	PHE	A	21	-18.209	4.854	21.865	1.00	0.00	C
ATOM	308	HE1	PHE	A	21	-19.101	5.290	21.745	1.00	0.00	
ATOM	309	CZ	PHE	A	21	-17.426	5.177	22.953	1.00	0.00	C
ATOM	310	HZ	PHE	A	21	-17.739	5.860	23.613	1.00	0.00	
ATOM	311	CD2	PHE	A	21	-15.753	3.651	22.176	1.00	0.00	C
ATOM	312	HD2	PHE	A	21	-14.861	3.215	22.299	1.00	0.00	
ATOM	313	CE2	PHE	A	21	-16.210	4.554	23.125	1.00	0.00	C
ATOM	314	HE2	PHE	A	21	-15.656	4.752	23.934	1.00	0.00	
ATOM	315	C	PHE	A	21	-16.370	0.006	19.126	1.00	0.00	C
ATOM	316	O	PHE	A	21	-16.924	0.214	18.059	1.00	0.00	O
ATOM	317	N	ALA	A	22	-15.539	-1.023	19.324	1.00	0.00	N
ATOM	318	HN	ALA	A	22	-15.265	-1.227	20.264	1.00	0.00	
ATOM	319	CA	ALA	A	22	-15.011	-1.859	18.273	1.00	0.00	C
ATOM	320	HA	ALA	A	22	-15.412	-1.526	17.420	1.00	0.00	
ATOM	321	CB	ALA	A	22	-13.510	-1.707	18.221	1.00	0.00	C
ATOM	322	HB1	ALA	A	22	-13.141	-2.285	17.493	1.00	0.00	
ATOM	323	HB2	ALA	A	22	-13.278	-0.752	18.037	1.00	0.00	
ATOM	324	HB3	ALA	A	22	-13.116	-1.983	19.098	1.00	0.00	
ATOM	325	C	ALA	A	22	-15.409	-3.325	18.498	1.00	0.00	C
ATOM	326	O	ALA	A	22	-15.451	-3.797	19.628	1.00	0.00	O
ATOM	327	N	VAL	A	23	-15.639	-4.041	17.392	1.00	0.00	N
ATOM	328	HN	VAL	A	23	-15.757	-3.557	16.525	1.00	0.00	
ATOM	329	CA	VAL	A	23	-15.725	-5.500	17.395	1.00	0.00	C
ATOM	330	HA	VAL	A	23	-15.694	-5.801	18.348	1.00	0.00	
ATOM	331	CB	VAL	A	23	-17.053	-5.984	16.777	1.00	0.00	C
ATOM	332	HB	VAL	A	23	-16.991	-5.851	15.788	1.00	0.00	
ATOM	333	CG1	VAL	A	23	-17.281	-7.461	17.026	1.00	0.00	C
ATOM	334	1HG1	VAL	A	23	-18.148	-7.739	16.613	1.00	0.00	
ATOM	335	2HG1	VAL	A	23	-16.534	-7.987	16.619	1.00	0.00	
ATOM	336	3HG1	VAL	A	23	-17.311	-7.632	18.011	1.00	0.00	
ATOM	337	CG2	VAL	A	23	-18.238	-5.175	17.275	1.00	0.00	C
ATOM	338	1HG2	VAL	A	23	-19.077	-5.518	16.853	1.00	0.00	
ATOM	339	2HG2	VAL	A	23	-18.306	-5.261	18.269	1.00	0.00	
ATOM	340	3HG2	VAL	A	23	-18.111	-4.214	17.031	1.00	0.00	
ATOM	341	C	VAL	A	23	-14.528	-6.032	16.613	1.00	0.00	C
ATOM	342	O	VAL	A	23	-14.186	-5.453	15.590	1.00	0.00	O
ATOM	343	N	VAL	A	24	-13.920	-7.129	17.078	1.00	0.00	N
ATOM	344	HN	VAL	A	24	-14.316	-7.609	17.861	1.00	0.00	
ATOM	345	CA	VAL	A	24	-12.702	-7.643	16.480	1.00	0.00	C
ATOM	346	HA	VAL	A	24	-12.544	-7.049	15.692	1.00	0.00	
ATOM	347	CB	VAL	A	24	-11.507	-7.553	17.443	1.00	0.00	C
ATOM	348	HB	VAL	A	24	-11.655	-8.218	18.175	1.00	0.00	
ATOM	349	CG1	VAL	A	24	-10.193	-7.917	16.749	1.00	0.00	C
ATOM	350	1HG1	VAL	A	24	-9.440	-7.848	17.403	1.00	0.00	

```
ATOM    351 2HG1 VAL A  24     -10.248  -8.853  16.402  1.00  0.00
ATOM    352 3HG1 VAL A  24     -10.032  -7.288  15.988  1.00  0.00
ATOM    353  CG2 VAL A  24     -11.424  -6.191  18.101  1.00  0.00           C
ATOM    354 1HG2 VAL A  24     -10.638  -6.167  18.719  1.00  0.00
ATOM    355 2HG2 VAL A  24     -11.317  -5.487  17.398  1.00  0.00
ATOM    356 3HG2 VAL A  24     -12.262  -6.020  18.620  1.00  0.00
ATOM    357  C   VAL A  24     -12.903  -9.089  16.036  1.00  0.00           C
ATOM    358  O   VAL A  24     -13.262  -9.946  16.858  1.00  0.00           O
ATOM    359  N   PHE A  25     -12.549  -9.357  14.766  1.00  0.00           N
ATOM    360  HN  PHE A  25     -12.181  -8.609  14.214  1.00  0.00
ATOM    361  CA  PHE A  25     -12.668 -10.665  14.147  1.00  0.00           C
ATOM    362  HA  PHE A  25     -13.027 -11.269  14.859  1.00  0.00
ATOM    363  CB  PHE A  25     -13.622 -10.591  12.965  1.00  0.00           C
ATOM    364  HB1 PHE A  25     -13.222  -9.982  12.280  1.00  0.00
ATOM    365  HB2 PHE A  25     -13.713 -11.510  12.581  1.00  0.00
ATOM    366  CG  PHE A  25     -15.008 -10.084  13.275  1.00  0.00           C
ATOM    367  CD1 PHE A  25     -16.010 -10.958  13.686  1.00  0.00           C
ATOM    368  HD1 PHE A  25     -15.796 -11.926  13.821  1.00  0.00
ATOM    369  CE1 PHE A  25     -17.301 -10.505  13.911  1.00  0.00           C
ATOM    370  HE1 PHE A  25     -18.013 -11.147  14.197  1.00  0.00
ATOM    371  CZ  PHE A  25     -17.605  -9.170  13.741  1.00  0.00           C
ATOM    372  HZ  PHE A  25     -18.529  -8.837  13.930  1.00  0.00
ATOM    373  CD2 PHE A  25     -15.334  -8.751  13.092  1.00  0.00           C
ATOM    374  HD2 PHE A  25     -14.629  -8.105  12.799  1.00  0.00
ATOM    375  CE2 PHE A  25     -16.632  -8.301  13.311  1.00  0.00           C
ATOM    376  HE2 PHE A  25     -16.858  -7.340  13.155  1.00  0.00
ATOM    377  C   PHE A  25     -11.303 -11.156  13.660  1.00  0.00           C
ATOM    378  O   PHE A  25     -10.438 -10.367  13.353  1.00  0.00           O
ATOM    379  N   ARG A  26     -11.134 -12.480  13.642  1.00  0.00           N
ATOM    380  HN  ARG A  26     -11.828 -13.037  14.098  1.00  0.00
ATOM    381  CA  ARG A  26     -10.018 -13.184  13.011  1.00  0.00           C
ATOM    382  HA  ARG A  26      -9.162 -12.686  13.153  1.00  0.00
ATOM    383  CB  ARG A  26      -9.874 -14.570  13.658  1.00  0.00           C
ATOM    384  HB1 ARG A  26      -9.907 -14.444  14.649  1.00  0.00
ATOM    385  HB2 ARG A  26     -10.656 -15.121  13.366  1.00  0.00
ATOM    386  CG  ARG A  26      -8.616 -15.376  13.349  1.00  0.00           C
ATOM    387  HG1 ARG A  26      -8.592 -15.563  12.367  1.00  0.00
ATOM    388  HG2 ARG A  26      -7.818 -14.829  13.604  1.00  0.00
ATOM    389  CD  ARG A  26      -8.562 -16.710  14.106  1.00  0.00           C
ATOM    390  HD1 ARG A  26      -8.019 -17.358  13.572  1.00  0.00
ATOM    391  HD2 ARG A  26      -8.123 -16.557  14.991  1.00  0.00
ATOM    392  NE  ARG A  26      -9.859 -17.364  14.380  1.00  0.00           N
ATOM    393  HE  ARG A  26     -10.271 -17.886  13.633  1.00  0.00
ATOM    394  CZ  ARG A  26     -10.541 -17.321  15.552  1.00  0.00           C
ATOM    395  NH1 ARG A  26     -10.052 -16.680  16.597  1.00  0.00           N
ATOM    396 1HH1 ARG A  26      -9.168 -16.218  16.531  1.00  0.00
ATOM    397 2HH1 ARG A  26     -10.566 -16.657  17.455  1.00  0.00
ATOM    398  NH2 ARG A  26     -11.723 -17.916  15.663  1.00  0.00           N
ATOM    399 1HH2 ARG A  26     -12.115 -18.399  14.880  1.00  0.00
ATOM    400 2HH2 ARG A  26     -12.221 -17.881  16.529  1.00  0.00
ATOM    401  C   ARG A  26     -10.337 -13.253  11.519  1.00  0.00           C
ATOM    402  O   ARG A  26     -11.440 -13.573  11.153  1.00  0.00           O
ATOM    403  N   GLY A  27      -9.395 -12.837  10.684  1.00  0.00           N
ATOM    404  HN  GLY A  27      -8.519 -12.536  11.060  1.00  0.00
ATOM    405  CA  GLY A  27      -9.585 -12.802   9.250  1.00  0.00           C
ATOM    406  HA1 GLY A  27     -10.365 -13.385   9.023  1.00  0.00
ATOM    407  HA2 GLY A  27      -9.785 -11.859   8.984  1.00  0.00
ATOM    408  C   GLY A  27      -8.346 -13.292   8.535  1.00  0.00           C
ATOM    409  O   GLY A  27      -7.427 -13.802   9.156  1.00  0.00           O
ATOM    410  N   ARG A  28      -8.323 -13.125   7.211  1.00  0.00           N
ATOM    411  HN  ARG A  28      -9.128 -12.743   6.756  1.00  0.00
ATOM    412  CA  ARG A  28      -7.178 -13.477   6.421  1.00  0.00           C
ATOM    413  HA  ARG A  28      -6.383 -13.301   7.001  1.00  0.00
ATOM    414  CB  ARG A  28      -7.234 -14.950   6.041  1.00  0.00           C
ATOM    415  HB1 ARG A  28      -6.432 -15.172   5.486  1.00  0.00
ATOM    416  HB2 ARG A  28      -7.224 -15.500   6.876  1.00  0.00
ATOM    417  CG  ARG A  28      -8.481 -15.291   5.250  1.00  0.00           C
ATOM    418  HG1 ARG A  28      -9.284 -15.097   5.814  1.00  0.00
ATOM    419  HG2 ARG A  28      -8.507 -14.723   4.427  1.00  0.00
ATOM    420  CD  ARG A  28      -8.527 -16.736   4.834  1.00  0.00           C
ATOM    421  HD1 ARG A  28      -7.644 -17.014   4.456  1.00  0.00
```

ATOM	422	HD2	ARG	A	28	-8.750	-17.313	5.619	1.00	0.00	
ATOM	423	NE	ARG	A	28	-9.549	-16.917	3.816	1.00	0.00	N
ATOM	424	HE	ARG	A	28	-9.891	-16.105	3.343	1.00	0.00	
ATOM	425	CZ	ARG	A	28	-10.048	-18.083	3.482	1.00	0.00	C
ATOM	426	NH1	ARG	A	28	-9.525	-19.183	3.994	1.00	0.00	N
ATOM	427	1HH1	ARG	A	28	-8.756	-19.122	4.630	1.00	0.00	
ATOM	428	2HH1	ARG	A	28	-9.898	-20.077	3.746	1.00	0.00	
ATOM	429	NH2	ARG	A	28	-11.029	-18.146	2.604	1.00	0.00	N
ATOM	430	1HH2	ARG	A	28	-11.389	-17.307	2.196	1.00	0.00	
ATOM	431	2HH2	ARG	A	28	-11.413	-19.033	2.346	1.00	0.00	
ATOM	432	C	ARG	A	28	-7.136	-12.613	5.165	1.00	0.00	C
ATOM	433	O	ARG	A	28	-8.130	-12.019	4.800	1.00	0.00	O
ATOM	434	N	HIS	A	29	-5.955	-12.562	4.543	1.00	0.00	N
ATOM	435	HN	HIS	A	29	-5.148	-12.898	5.029	1.00	0.00	
ATOM	436	CA	HIS	A	29	-5.781	-12.042	3.192	1.00	0.00	C
ATOM	437	HA	HIS	A	29	-6.184	-11.127	3.176	1.00	0.00	
ATOM	438	CB	HIS	A	29	-4.283	-11.930	2.836	1.00	0.00	C
ATOM	439	HB1	HIS	A	29	-3.791	-11.528	3.608	1.00	0.00	
ATOM	440	HB2	HIS	A	29	-3.921	-12.843	2.649	1.00	0.00	
ATOM	441	ND1	HIS	A	29	-3.949	-11.610	0.371	1.00	0.00	N
ATOM	442	CG	HIS	A	29	-4.009	-11.085	1.640	1.00	0.00	C
ATOM	443	CE1	HIS	A	29	-3.704	-10.640	-0.493	1.00	0.00	C
ATOM	444	HE1	HIS	A	29	-3.616	-10.746	-1.483	1.00	0.00	
ATOM	445	NE2	HIS	A	29	-3.595	-9.495	0.183	1.00	0.00	N
ATOM	446	HE2	HIS	A	29	-3.413	-8.596	-0.215	1.00	0.00	
ATOM	447	CD2	HIS	A	29	-3.775	-9.759	1.516	1.00	0.00	C
ATOM	448	HD2	HIS	A	29	-3.740	-9.093	2.261	1.00	0.00	
ATOM	449	C	HIS	A	29	-6.521	-12.959	2.220	1.00	0.00	C
ATOM	450	O	HIS	A	29	-6.614	-14.161	2.430	1.00	0.00	O
ATOM	451	N	ARG	A	30	-7.041	-12.368	1.151	1.00	0.00	N
ATOM	452	HN	ARG	A	30	-6.828	-11.406	0.978	1.00	0.00	
ATOM	453	CA	ARG	A	30	-7.908	-13.073	0.225	1.00	0.00	C
ATOM	454	HA	ARG	A	30	-8.484	-13.688	0.763	1.00	0.00	
ATOM	455	CB	ARG	A	30	-8.829	-12.077	-0.466	1.00	0.00	C
ATOM	456	HB1	ARG	A	30	-8.271	-11.343	-0.852	1.00	0.00	
ATOM	457	HB2	ARG	A	30	-9.313	-12.550	-1.202	1.00	0.00	
ATOM	458	CG	ARG	A	30	-9.858	-11.463	0.482	1.00	0.00	C
ATOM	459	HG1	ARG	A	30	-10.511	-12.166	0.763	1.00	0.00	
ATOM	460	HG2	ARG	A	30	-9.391	-11.100	1.288	1.00	0.00	
ATOM	461	CD	ARG	A	30	-10.606	-10.336	-0.211	1.00	0.00	C
ATOM	462	HD1	ARG	A	30	-11.170	-9.832	0.443	1.00	0.00	
ATOM	463	HD2	ARG	A	30	-9.966	-9.707	-0.653	1.00	0.00	
ATOM	464	NE	ARG	A	30	-11.470	-10.931	-1.227	1.00	0.00	N
ATOM	465	HE	ARG	A	30	-12.112	-11.638	-0.931	1.00	0.00	
ATOM	466	CZ	ARG	A	30	-11.468	-10.605	-2.513	1.00	0.00	C
ATOM	467	NH1	ARG	A	30	-10.989	-9.424	-2.885	1.00	0.00	N
ATOM	468	1HH1	ARG	A	30	-10.634	-8.790	-2.198	1.00	0.00	
ATOM	469	2HH1	ARG	A	30	-10.983	-9.169	-3.852	1.00	0.00	
ATOM	470	NH2	ARG	A	30	-11.922	-11.475	-3.404	1.00	0.00	N
ATOM	471	1HH2	ARG	A	30	-12.261	-12.367	-3.104	1.00	0.00	
ATOM	472	2HH2	ARG	A	30	-11.925	-11.240	-4.376	1.00	0.00	
ATOM	473	C	ARG	A	30	-7.089	-13.957	-0.724	1.00	0.00	C
ATOM	474	O	ARG	A	30	-7.539	-15.003	-1.063	1.00	0.00	O
ATOM	475	N	GLN	A	31	-5.879	-13.558	-1.084	1.00	0.00	N
ATOM	476	HN	GLN	A	31	-5.563	-12.652	-0.803	1.00	0.00	
ATOM	477	CA	GLN	A	31	-4.974	-14.419	-1.895	1.00	0.00	C
ATOM	478	HA	GLN	A	31	-5.572	-15.022	-2.424	1.00	0.00	
ATOM	479	CB	GLN	A	31	-4.131	-13.587	-2.856	1.00	0.00	C
ATOM	480	HB1	GLN	A	31	-3.573	-12.951	-2.323	1.00	0.00	
ATOM	481	HB2	GLN	A	31	-3.534	-14.202	-3.371	1.00	0.00	
ATOM	482	CG	GLN	A	31	-4.939	-12.785	-3.843	1.00	0.00	C
ATOM	483	HG1	GLN	A	31	-5.435	-13.411	-4.445	1.00	0.00	
ATOM	484	HG2	GLN	A	31	-5.592	-12.214	-3.344	1.00	0.00	
ATOM	485	CD	GLN	A	31	-4.042	-11.901	-4.677	1.00	0.00	C
ATOM	486	OE1	GLN	A	31	-3.409	-12.347	-5.649	1.00	0.00	O
ATOM	487	NE2	GLN	A	31	-3.984	-10.625	-4.294	1.00	0.00	N
ATOM	488	1HE2	GLN	A	31	-4.513	-10.316	-3.504	1.00	0.00	
ATOM	489	2HE2	GLN	A	31	-3.411	-9.978	-4.797	1.00	0.00	
ATOM	490	C	GLN	A	31	-4.049	-15.242	-0.982	1.00	0.00	C
ATOM	491	O	GLN	A	31	-3.988	-16.446	-1.087	1.00	0.00	O
ATOM	492	N	LYS	A	32	-3.333	-14.558	-0.095	1.00	0.00	N

ATOM	493	HN	LYS	A	32	-3.444	-13.565	-0.063	1.00	0.00	
ATOM	494	CA	LYS	A	32	-2.399	-15.173	0.829	1.00	0.00	C
ATOM	495	HA	LYS	A	32	-1.996	-15.977	0.391	1.00	0.00	
ATOM	496	CB	LYS	A	32	-1.292	-14.160	1.151	1.00	0.00	C
ATOM	497	HB1	LYS	A	32	-0.977	-13.760	0.290	1.00	0.00	
ATOM	498	HB2	LYS	A	32	-1.684	-13.444	1.728	1.00	0.00	
ATOM	499	CG	LYS	A	32	-0.077	-14.707	1.869	1.00	0.00	C
ATOM	500	HG1	LYS	A	32	-0.354	-15.012	2.780	1.00	0.00	
ATOM	501	HG2	LYS	A	32	0.278	-15.485	1.350	1.00	0.00	
ATOM	502	CD	LYS	A	32	1.026	-13.700	2.024	1.00	0.00	C
ATOM	503	HD1	LYS	A	32	1.421	-13.516	1.124	1.00	0.00	
ATOM	504	HD2	LYS	A	32	0.639	-12.856	2.397	1.00	0.00	
ATOM	505	CE	LYS	A	32	2.117	-14.191	2.948	1.00	0.00	C
ATOM	506	HE1	LYS	A	32	2.852	-13.514	2.985	1.00	0.00	
ATOM	507	HE2	LYS	A	32	1.742	-14.325	3.865	1.00	0.00	
ATOM	508	NZ	LYS	A	32	2.680	-15.477	2.474	1.00	0.00	N
ATOM	509	HZ1	LYS	A	32	3.398	-15.778	3.102	1.00	0.00	
ATOM	510	HZ2	LYS	A	32	1.956	-16.166	2.440	1.00	0.00	
ATOM	511	HZ3	LYS	A	32	3.067	-15.355	1.560	1.00	0.00	
ATOM	512	C	LYS	A	32	-3.172	-15.605	2.083	1.00	0.00	C
ATOM	513	O	LYS	A	32	-3.078	-14.985	3.127	1.00	0.00	O
ATOM	514	N	THR	A	33	-3.926	-16.693	1.965	1.00	0.00	N
ATOM	515	HN	THR	A	33	-3.797	-17.284	1.169	1.00	0.00	
ATOM	516	CA	THR	A	33	-4.942	-17.059	2.960	1.00	0.00	C
ATOM	517	HA	THR	A	33	-5.420	-16.207	3.175	1.00	0.00	
ATOM	518	CB	THR	A	33	-5.939	-18.068	2.379	1.00	0.00	C
ATOM	519	HB	THR	A	33	-6.571	-18.384	3.086	1.00	0.00	
ATOM	520	OG1	THR	A	33	-5.191	-19.178	1.898	1.00	0.00	O
ATOM	521	HG1	THR	A	33	-5.814	-19.857	1.511	1.00	0.00	
ATOM	522	CG2	THR	A	33	-6.753	-17.502	1.238	1.00	0.00	C
ATOM	523	1HG2	THR	A	33	-7.383	-18.201	0.900	1.00	0.00	
ATOM	524	2HG2	THR	A	33	-7.273	-16.711	1.560	1.00	0.00	
ATOM	525	3HG2	THR	A	33	-6.141	-17.220	0.500	1.00	0.00	
ATOM	526	C	THR	A	33	-4.294	-17.605	4.236	1.00	0.00	C
ATOM	527	O	THR	A	33	-4.983	-17.749	5.232	1.00	0.00	O
ATOM	528	N	ASP	A	34	-2.984	-17.886	4.196	1.00	0.00	N
ATOM	529	HN	ASP	A	34	-2.495	-17.786	3.329	1.00	0.00	
ATOM	530	CA	ASP	A	34	-2.237	-18.338	5.387	1.00	0.00	C
ATOM	531	HA	ASP	A	34	-2.834	-18.988	5.858	1.00	0.00	
ATOM	532	CB	ASP	A	34	-0.956	-19.096	5.003	1.00	0.00	C
ATOM	533	HB1	ASP	A	34	-0.402	-19.211	5.827	1.00	0.00	
ATOM	534	HB2	ASP	A	34	-1.218	-19.994	4.650	1.00	0.00	
ATOM	535	CG	ASP	A	34	-0.092	-18.407	3.946	1.00	0.00	C
ATOM	536	OD1	ASP	A	34	-0.630	-18.149	2.834	1.00	0.00	O
ATOM	537	OD2	ASP	A	34	1.115	-18.143	4.228	1.00	0.00	O
ATOM	538	C	ASP	A	34	-1.951	-17.139	6.315	1.00	0.00	C
ATOM	539	O	ASP	A	34	-1.646	-17.315	7.487	1.00	0.00	O
ATOM	540	N	TRP	A	35	-2.062	-15.924	5.774	1.00	0.00	N
ATOM	541	HN	TRP	A	35	-2.341	-15.860	4.816	1.00	0.00	
ATOM	542	CA	TRP	A	35	-1.801	-14.685	6.494	1.00	0.00	C
ATOM	543	HA	TRP	A	35	-1.038	-14.860	7.116	1.00	0.00	
ATOM	544	CB	TRP	A	35	-1.408	-13.596	5.496	1.00	0.00	C
ATOM	545	HB1	TRP	A	35	-0.548	-13.862	5.060	1.00	0.00	
ATOM	546	HB2	TRP	A	35	-2.126	-13.528	4.803	1.00	0.00	
ATOM	547	CG	TRP	A	35	-1.213	-12.231	6.071	1.00	0.00	C
ATOM	548	CD1	TRP	A	35	-1.077	-11.901	7.382	1.00	0.00	C
ATOM	549	HD1	TRP	A	35	-1.097	-12.550	8.142	1.00	0.00	
ATOM	550	NE1	TRP	A	35	-0.910	-10.555	7.508	1.00	0.00	N
ATOM	551	HE1	TRP	A	35	-0.808	-10.063	8.373	1.00	0.00	
ATOM	552	CE2	TRP	A	35	-0.903	-9.982	6.267	1.00	0.00	C
ATOM	553	CD2	TRP	A	35	-1.058	-11.011	5.326	1.00	0.00	C
ATOM	554	CE3	TRP	A	35	-1.045	-10.684	3.961	1.00	0.00	C
ATOM	555	HE3	TRP	A	35	-1.151	-11.397	3.267	1.00	0.00	
ATOM	556	CZ3	TRP	A	35	-0.884	-9.367	3.591	1.00	0.00	C
ATOM	557	HZ3	TRP	A	35	-0.888	-9.125	2.621	1.00	0.00	
ATOM	558	CZ2	TRP	A	35	-0.674	-8.664	5.881	1.00	0.00	C
ATOM	559	HZ2	TRP	A	35	-0.485	-7.954	6.559	1.00	0.00	
ATOM	560	CH2	TRP	A	35	-0.716	-8.369	4.545	1.00	0.00	C
ATOM	561	HH2	TRP	A	35	-0.624	-7.418	4.249	1.00	0.00	
ATOM	562	C	TRP	A	35	-3.034	-14.282	7.315	1.00	0.00	C
ATOM	563	O	TRP	A	35	-4.009	-13.761	6.771	1.00	0.00	O

ATOM	564	N	GLU	A	36	-2.975	-14.529	8.631	1.00	0.00	N
ATOM	565	HN	GLU	A	36	-2.142	-14.930	9.012	1.00	0.00	
ATOM	566	CA	GLU	A	36	-4.078	-14.234	9.516	1.00	0.00	C
ATOM	567	HA	GLU	A	36	-4.913	-14.383	8.986	1.00	0.00	
ATOM	568	CB	GLU	A	36	-4.083	-15.178	10.711	1.00	0.00	C
ATOM	569	HB1	GLU	A	36	-3.152	-15.247	11.069	1.00	0.00	
ATOM	570	HB2	GLU	A	36	-4.684	-14.800	11.415	1.00	0.00	
ATOM	571	CG	GLU	A	36	-4.570	-16.573	10.357	1.00	0.00	C
ATOM	572	HG1	GLU	A	36	-5.406	-16.487	9.816	1.00	0.00	
ATOM	573	HG2	GLU	A	36	-3.864	-17.024	9.811	1.00	0.00	
ATOM	574	CD	GLU	A	36	-4.870	-17.445	11.561	1.00	0.00	C
ATOM	575	OE1	GLU	A	36	-4.070	-17.394	12.507	1.00	0.00	O
ATOM	576	OE2	GLU	A	36	-5.919	-18.141	11.565	1.00	0.00	O
ATOM	577	C	GLU	A	36	-3.969	-12.779	9.972	1.00	0.00	C
ATOM	578	O	GLU	A	36	-2.860	-12.257	10.169	1.00	0.00	O
ATOM	579	N	VAL	A	37	-5.127	-12.134	10.125	1.00	0.00	N
ATOM	580	HN	VAL	A	37	-5.978	-12.616	9.919	1.00	0.00	
ATOM	581	CA	VAL	A	37	-5.193	-10.789	10.570	1.00	0.00	C
ATOM	582	HA	VAL	A	37	-4.311	-10.638	11.017	1.00	0.00	
ATOM	583	CB	VAL	A	37	-5.358	-9.815	9.394	1.00	0.00	C
ATOM	584	HB	VAL	A	37	-5.405	-8.893	9.777	1.00	0.00	
ATOM	585	CG1	VAL	A	37	-4.173	-9.866	8.444	1.00	0.00	C
ATOM	586	1HG1	VAL	A	37	-4.318	-9.220	7.695	1.00	0.00	
ATOM	587	2HG1	VAL	A	37	-3.339	-9.621	8.938	1.00	0.00	
ATOM	588	3HG1	VAL	A	37	-4.082	-10.791	8.074	1.00	0.00	
ATOM	589	CG2	VAL	A	37	-6.643	-10.054	8.643	1.00	0.00	C
ATOM	590	1HG2	VAL	A	37	-6.714	-9.403	7.888	1.00	0.00	
ATOM	591	2HG2	VAL	A	37	-6.649	-10.986	8.281	1.00	0.00	
ATOM	592	3HG2	VAL	A	37	-7.419	-9.932	9.262	1.00	0.00	
ATOM	593	C	VAL	A	37	-6.345	-10.632	11.556	1.00	0.00	C
ATOM	594	O	VAL	A	37	-7.295	-11.399	11.547	1.00	0.00	O
ATOM	595	N	ALA	A	38	-6.230	-9.613	12.404	1.00	0.00	N
ATOM	596	HN	ALA	A	38	-5.342	-9.159	12.480	1.00	0.00	
ATOM	597	CA	ALA	A	38	-7.320	-9.126	13.223	1.00	0.00	C
ATOM	598	HA	ALA	A	38	-7.967	-9.877	13.353	1.00	0.00	
ATOM	599	CB	ALA	A	38	-6.804	-8.701	14.564	1.00	0.00	C
ATOM	600	HB1	ALA	A	38	-7.563	-8.366	15.122	1.00	0.00	
ATOM	601	HB2	ALA	A	38	-6.374	-9.481	15.018	1.00	0.00	
ATOM	602	HB3	ALA	A	38	-6.130	-7.972	14.446	1.00	0.00	
ATOM	603	C	ALA	A	38	-7.968	-7.952	12.508	1.00	0.00	C
ATOM	604	O	ALA	A	38	-7.276	-7.049	12.072	1.00	0.00	O
ATOM	605	N	ILE	A	39	-9.292	-7.976	12.408	1.00	0.00	N
ATOM	606	HN	ILE	A	39	-9.793	-8.758	12.779	1.00	0.00	
ATOM	607	CA	ILE	A	39	-10.024	-6.911	11.782	1.00	0.00	C
ATOM	608	HA	ILE	A	39	-9.367	-6.220	11.481	1.00	0.00	
ATOM	609	CB	ILE	A	39	-10.748	-7.469	10.549	1.00	0.00	C
ATOM	610	HB	ILE	A	39	-11.339	-8.222	10.838	1.00	0.00	
ATOM	611	CG2	ILE	A	39	-11.661	-6.432	9.913	1.00	0.00	C
ATOM	612	1HG2	ILE	A	39	-12.114	-6.831	9.116	1.00	0.00	
ATOM	613	2HG2	ILE	A	39	-12.350	-6.142	10.577	1.00	0.00	
ATOM	614	3HG2	ILE	A	39	-11.119	-5.641	9.628	1.00	0.00	
ATOM	615	CG1	ILE	A	39	-9.735	-8.014	9.549	1.00	0.00	C
ATOM	616	1HG1	ILE	A	39	-9.151	-7.266	9.235	1.00	0.00	
ATOM	617	2HG1	ILE	A	39	-9.174	-8.709	9.998	1.00	0.00	
ATOM	618	CD	ILE	A	39	-10.376	-8.635	8.355	1.00	0.00	C
ATOM	619	HD1	ILE	A	39	-9.668	-8.974	7.735	1.00	0.00	
ATOM	620	HD2	ILE	A	39	-10.956	-9.396	8.646	1.00	0.00	
ATOM	621	HD3	ILE	A	39	-10.933	-7.952	7.883	1.00	0.00	
ATOM	622	C	ILE	A	39	-10.962	-6.266	12.815	1.00	0.00	C
ATOM	623	O	ILE	A	39	-11.895	-6.919	13.300	1.00	0.00	O
ATOM	624	N	LYS	A	40	-10.676	-5.005	13.175	1.00	0.00	N
ATOM	625	HN	LYS	A	40	-9.849	-4.579	12.810	1.00	0.00	
ATOM	626	CA	LYS	A	40	-11.526	-4.226	14.082	1.00	0.00	C
ATOM	627	HA	LYS	A	40	-11.925	-4.910	14.693	1.00	0.00	
ATOM	628	CB	LYS	A	40	-10.737	-3.182	14.868	1.00	0.00	C
ATOM	629	HB1	LYS	A	40	-10.120	-2.722	14.230	1.00	0.00	
ATOM	630	HB2	LYS	A	40	-11.390	-2.520	15.236	1.00	0.00	
ATOM	631	CG	LYS	A	40	-9.899	-3.681	16.026	1.00	0.00	C
ATOM	632	HG1	LYS	A	40	-10.464	-4.270	16.604	1.00	0.00	
ATOM	633	HG2	LYS	A	40	-9.130	-4.208	15.665	1.00	0.00	
ATOM	634	CD	LYS	A	40	-9.363	-2.531	16.864	1.00	0.00	C

ATOM	635	HD1	LYS	A	40	-8.806	-1.942	16.278	1.00	0.00	
ATOM	636	HD2	LYS	A	40	-10.137	-2.009	17.222	1.00	0.00	
ATOM	637	CE	LYS	A	40	-8.512	-2.989	18.031	1.00	0.00	C
ATOM	638	HE1	LYS	A	40	-9.100	-3.381	18.739	1.00	0.00	
ATOM	639	HE2	LYS	A	40	-7.861	-3.680	17.718	1.00	0.00	
ATOM	640	NZ	LYS	A	40	-7.754	-1.862	18.621	1.00	0.00	N
ATOM	641	HZ1	LYS	A	40	-7.204	-2.195	19.387	1.00	0.00	
ATOM	642	HZ2	LYS	A	40	-7.154	-1.464	17.927	1.00	0.00	
ATOM	643	HZ3	LYS	A	40	-8.393	-1.165	18.948	1.00	0.00	
ATOM	644	C	LYS	A	40	-12.557	-3.448	13.271	1.00	0.00	C
ATOM	645	O	LYS	A	40	-12.190	-2.681	12.406	1.00	0.00	O
ATOM	646	N	SER	A	41	-13.838	-3.656	13.584	1.00	0.00	N
ATOM	647	HN	SER	A	41	-14.059	-4.406	14.208	1.00	0.00	
ATOM	648	CA	SER	A	41	-14.923	-2.850	13.065	1.00	0.00	C
ATOM	649	HA	SER	A	41	-14.588	-2.404	12.235	1.00	0.00	
ATOM	650	CB	SER	A	41	-16.075	-3.711	12.687	1.00	0.00	C
ATOM	651	HB1	SER	A	41	-15.834	-4.292	11.910	1.00	0.00	
ATOM	652	HB2	SER	A	41	-16.348	-4.284	13.460	1.00	0.00	
ATOM	653	OG	SER	A	41	-17.182	-2.911	12.316	1.00	0.00	O
ATOM	654	HG1	SER	A	41	-17.950	-3.500	12.064	1.00	0.00	
ATOM	655	C	SER	A	41	-15.335	-1.821	14.113	1.00	0.00	C
ATOM	656	O	SER	A	41	-15.595	-2.188	15.252	1.00	0.00	O
ATOM	657	N	ILE	A	42	-15.385	-0.552	13.707	1.00	0.00	N
ATOM	658	HN	ILE	A	42	-15.227	-0.363	12.738	1.00	0.00	
ATOM	659	CA	ILE	A	42	-15.654	0.571	14.584	1.00	0.00	C
ATOM	660	HA	ILE	A	42	-15.933	0.184	15.463	1.00	0.00	
ATOM	661	CB	ILE	A	42	-14.388	1.420	14.773	1.00	0.00	C
ATOM	662	HB	ILE	A	42	-14.124	1.762	13.871	1.00	0.00	
ATOM	663	CG2	ILE	A	42	-14.670	2.626	15.675	1.00	0.00	C
ATOM	664	1HG2	ILE	A	42	-13.834	3.163	15.784	1.00	0.00	
ATOM	665	2HG2	ILE	A	42	-15.380	3.195	15.259	1.00	0.00	
ATOM	666	3HG2	ILE	A	42	-14.980	2.307	16.571	1.00	0.00	
ATOM	667	CG1	ILE	A	42	-13.196	0.602	15.277	1.00	0.00	C
ATOM	668	1HG1	ILE	A	42	-13.370	0.340	16.226	1.00	0.00	
ATOM	669	2HG1	ILE	A	42	-13.115	-0.220	14.714	1.00	0.00	
ATOM	670	CD	ILE	A	42	-11.868	1.364	15.219	1.00	0.00	C
ATOM	671	HD1	ILE	A	42	-11.133	0.778	15.560	1.00	0.00	
ATOM	672	HD2	ILE	A	42	-11.675	1.625	14.273	1.00	0.00	
ATOM	673	HD3	ILE	A	42	-11.930	2.185	15.786	1.00	0.00	
ATOM	674	C	ILE	A	42	-16.775	1.419	13.967	1.00	0.00	C
ATOM	675	O	ILE	A	42	-16.597	2.022	12.915	1.00	0.00	O
ATOM	676	N	ASN	A	43	-17.927	1.493	14.636	1.00	0.00	N
ATOM	677	HN	ASN	A	43	-18.034	0.963	15.477	1.00	0.00	
ATOM	678	CA	ASN	A	43	-19.037	2.325	14.176	1.00	0.00	C
ATOM	679	HA	ASN	A	43	-19.220	2.067	13.227	1.00	0.00	
ATOM	680	CB	ASN	A	43	-20.294	2.040	14.995	1.00	0.00	C
ATOM	681	HB1	ASN	A	43	-20.567	1.089	14.848	1.00	0.00	
ATOM	682	HB2	ASN	A	43	-20.088	2.182	15.963	1.00	0.00	
ATOM	683	CG	ASN	A	43	-21.454	2.932	14.619	1.00	0.00	C
ATOM	684	OD1	ASN	A	43	-21.708	3.932	15.280	1.00	0.00	O
ATOM	685	ND2	ASN	A	43	-22.152	2.581	13.559	1.00	0.00	N
ATOM	686	1HD2	ASN	A	43	-21.904	1.759	13.047	1.00	0.00	
ATOM	687	2HD2	ASN	A	43	-22.930	3.137	13.266	1.00	0.00	
ATOM	688	C	ASN	A	43	-18.630	3.809	14.246	1.00	0.00	C
ATOM	689	O	ASN	A	43	-18.177	4.275	15.278	1.00	0.00	O
ATOM	690	N	LYS	A	44	-18.828	4.547	13.148	1.00	0.00	N
ATOM	691	HN	LYS	A	44	-19.317	4.136	12.379	1.00	0.00	
ATOM	692	CA	LYS	A	44	-18.356	5.947	13.018	1.00	0.00	C
ATOM	693	HA	LYS	A	44	-17.412	5.934	13.347	1.00	0.00	
ATOM	694	CB	LYS	A	44	-18.388	6.415	11.558	1.00	0.00	C
ATOM	695	HB1	LYS	A	44	-19.224	6.067	11.133	1.00	0.00	
ATOM	696	HB2	LYS	A	44	-18.400	7.415	11.547	1.00	0.00	
ATOM	697	CG	LYS	A	44	-17.209	5.951	10.720	1.00	0.00	C
ATOM	698	HG1	LYS	A	44	-16.401	6.467	11.004	1.00	0.00	
ATOM	699	HG2	LYS	A	44	-17.062	4.978	10.898	1.00	0.00	
ATOM	700	CD	LYS	A	44	-17.378	6.132	9.230	1.00	0.00	C
ATOM	701	HD1	LYS	A	44	-16.991	5.333	8.769	1.00	0.00	
ATOM	702	HD2	LYS	A	44	-18.356	6.192	9.028	1.00	0.00	
ATOM	703	CE	LYS	A	44	-16.695	7.378	8.695	1.00	0.00	C
ATOM	704	HE1	LYS	A	44	-17.121	8.189	9.096	1.00	0.00	
ATOM	705	HE2	LYS	A	44	-15.725	7.356	8.939	1.00	0.00	

ATOM	706	NZ	LYS	A	44	-16.807	7.469	7.217	1.00	0.00	N
ATOM	707	HZ1	LYS	A	44	-16.348	8.298	6.898	1.00	0.00	
ATOM	708	HZ2	LYS	A	44	-16.376	6.669	6.800	1.00	0.00	
ATOM	709	HZ3	LYS	A	44	-17.772	7.501	6.957	1.00	0.00	
ATOM	710	C	LYS	A	44	-19.193	6.897	13.881	1.00	0.00	C
ATOM	711	O	LYS	A	44	-18.660	7.866	14.425	1.00	0.00	O
ATOM	712	N	LYS	A	45	-20.498	6.625	14.005	1.00	0.00	N
ATOM	713	HN	LYS	A	45	-20.868	5.805	13.569	1.00	0.00	
ATOM	714	CA	LYS	A	45	-21.400	7.501	14.765	1.00	0.00	C
ATOM	715	HA	LYS	A	45	-21.174	8.436	14.491	1.00	0.00	
ATOM	716	CB	LYS	A	45	-22.865	7.245	14.397	1.00	0.00	C
ATOM	717	HB1	LYS	A	45	-22.954	6.295	14.097	1.00	0.00	
ATOM	718	HB2	LYS	A	45	-23.426	7.392	15.212	1.00	0.00	
ATOM	719	CG	LYS	A	45	-23.398	8.141	13.290	1.00	0.00	C
ATOM	720	HG1	LYS	A	45	-23.705	8.999	13.703	1.00	0.00	
ATOM	721	HG2	LYS	A	45	-22.651	8.327	12.652	1.00	0.00	
ATOM	722	CD	LYS	A	45	-24.557	7.554	12.503	1.00	0.00	C
ATOM	723	HD1	LYS	A	45	-24.414	6.569	12.407	1.00	0.00	
ATOM	724	HD2	LYS	A	45	-25.405	7.721	13.006	1.00	0.00	
ATOM	725	CE	LYS	A	45	-24.687	8.161	11.121	1.00	0.00	C
ATOM	726	HE1	LYS	A	45	-25.258	8.981	11.169	1.00	0.00	
ATOM	727	HE2	LYS	A	45	-23.780	8.406	10.778	1.00	0.00	
ATOM	728	NZ	LYS	A	45	-25.308	7.211	10.170	1.00	0.00	N
ATOM	729	HZ1	LYS	A	45	-25.380	7.640	9.269	1.00	0.00	
ATOM	730	HZ2	LYS	A	45	-24.743	6.389	10.104	1.00	0.00	
ATOM	731	HZ3	LYS	A	45	-26.221	6.963	10.495	1.00	0.00	
ATOM	732	C	LYS	A	45	-21.151	7.335	16.273	1.00	0.00	C
ATOM	733	O	LYS	A	45	-21.156	8.310	17.003	1.00	0.00	O
ATOM	734	N	ASN	A	46	-20.940	6.100	16.738	1.00	0.00	N
ATOM	735	HN	ASN	A	46	-20.994	5.325	16.109	1.00	0.00	
ATOM	736	CA	ASN	A	46	-20.634	5.859	18.137	1.00	0.00	C
ATOM	737	HA	ASN	A	46	-21.423	6.223	18.632	1.00	0.00	
ATOM	738	CB	ASN	A	46	-20.491	4.377	18.474	1.00	0.00	C
ATOM	739	HB1	ASN	A	46	-19.900	3.943	17.794	1.00	0.00	
ATOM	740	HB2	ASN	A	46	-20.078	4.286	19.380	1.00	0.00	
ATOM	741	CG	ASN	A	46	-21.806	3.658	18.482	1.00	0.00	C
ATOM	742	OD1	ASN	A	46	-22.839	4.278	18.680	1.00	0.00	O
ATOM	743	ND2	ASN	A	46	-21.770	2.357	18.246	1.00	0.00	N
ATOM	744	1HD2	ASN	A	46	-20.895	1.904	18.074	1.00	0.00	
ATOM	745	2HD2	ASN	A	46	-22.618	1.826	18.239	1.00	0.00	
ATOM	746	C	ASN	A	46	-19.339	6.578	18.489	1.00	0.00	C
ATOM	747	O	ASN	A	46	-19.231	7.201	19.550	1.00	0.00	O
ATOM	748	N	LEU	A	47	-18.365	6.474	17.584	1.00	0.00	N
ATOM	749	HN	LEU	A	47	-18.561	6.009	16.720	1.00	0.00	
ATOM	750	CA	LEU	A	47	-17.043	7.003	17.799	1.00	0.00	C
ATOM	751	HA	LEU	A	47	-16.719	6.627	18.667	1.00	0.00	
ATOM	752	CB	LEU	A	47	-16.153	6.550	16.650	1.00	0.00	C
ATOM	753	HB1	LEU	A	47	-16.147	5.550	16.637	1.00	0.00	
ATOM	754	HB2	LEU	A	47	-16.549	6.892	15.798	1.00	0.00	
ATOM	755	CG	LEU	A	47	-14.716	7.020	16.714	1.00	0.00	C
ATOM	756	HG	LEU	A	47	-14.688	8.016	16.793	1.00	0.00	
ATOM	757	CD1	LEU	A	47	-14.026	6.448	17.935	1.00	0.00	C
ATOM	758	1HD1	LEU	A	47	-13.079	6.767	17.963	1.00	0.00	
ATOM	759	2HD1	LEU	A	47	-14.504	6.750	18.760	1.00	0.00	
ATOM	760	3HD1	LEU	A	47	-14.040	5.449	17.888	1.00	0.00	
ATOM	761	CD2	LEU	A	47	-14.010	6.618	15.442	1.00	0.00	C
ATOM	762	1HD2	LEU	A	47	-13.059	6.925	15.477	1.00	0.00	
ATOM	763	2HD2	LEU	A	47	-14.035	5.623	15.347	1.00	0.00	
ATOM	764	3HD2	LEU	A	47	-14.468	7.039	14.659	1.00	0.00	
ATOM	765	C	LEU	A	47	-17.085	8.534	17.895	1.00	0.00	C
ATOM	766	O	LEU	A	47	-16.308	9.124	18.644	1.00	0.00	O
ATOM	767	N	SER	A	48	-17.986	9.160	17.131	1.00	0.00	N
ATOM	768	HN	SER	A	48	-18.602	8.610	16.566	1.00	0.00	
ATOM	769	CA	SER	A	48	-18.107	10.611	17.088	1.00	0.00	C
ATOM	770	HA	SER	A	48	-17.167	10.949	17.043	1.00	0.00	
ATOM	771	CB	SER	A	48	-18.808	11.068	15.846	1.00	0.00	C
ATOM	772	HB1	SER	A	48	-18.481	11.972	15.571	1.00	0.00	
ATOM	773	HB2	SER	A	48	-18.660	10.419	15.100	1.00	0.00	
ATOM	774	OG	SER	A	48	-20.194	11.158	16.076	1.00	0.00	O
ATOM	775	HG1	SER	A	48	-20.651	11.464	15.241	1.00	0.00	
ATOM	776	C	SER	A	48	-18.813	11.143	18.348	1.00	0.00	C

ATOM	777	O	SER	A	48	-18.821	12.330	18.570	1.00	0.00	O
ATOM	778	N	LYS	A	49	-19.393	10.254	19.159	1.00	0.00	N
ATOM	779	HN	LYS	A	49	-19.409	9.293	18.882	1.00	0.00	
ATOM	780	CA	LYS	A	49	-20.017	10.630	20.458	1.00	0.00	C
ATOM	781	HA	LYS	A	49	-20.206	11.610	20.390	1.00	0.00	
ATOM	782	CB	LYS	A	49	-21.325	9.864	20.700	1.00	0.00	C
ATOM	783	HB1	LYS	A	49	-21.130	8.886	20.623	1.00	0.00	
ATOM	784	HB2	LYS	A	49	-21.637	10.069	21.628	1.00	0.00	
ATOM	785	CG	LYS	A	49	-22.469	10.181	19.750	1.00	0.00	C
ATOM	786	HG1	LYS	A	49	-22.727	11.139	19.872	1.00	0.00	
ATOM	787	HG2	LYS	A	49	-22.153	10.038	18.812	1.00	0.00	
ATOM	788	CD	LYS	A	49	-23.697	9.322	19.977	1.00	0.00	C
ATOM	789	HD1	LYS	A	49	-23.414	8.364	20.030	1.00	0.00	
ATOM	790	HD2	LYS	A	49	-24.123	9.592	20.841	1.00	0.00	
ATOM	791	CE	LYS	A	49	-24.715	9.471	18.862	1.00	0.00	C
ATOM	792	HE1	LYS	A	49	-24.964	10.435	18.767	1.00	0.00	
ATOM	793	HE2	LYS	A	49	-24.318	9.143	18.005	1.00	0.00	
ATOM	794	NZ	LYS	A	49	-25.948	8.696	19.132	1.00	0.00	N
ATOM	795	HZ1	LYS	A	49	-26.592	8.820	18.377	1.00	0.00	
ATOM	796	HZ2	LYS	A	49	-25.720	7.726	19.220	1.00	0.00	
ATOM	797	HZ3	LYS	A	49	-26.365	9.019	19.982	1.00	0.00	
ATOM	798	C	LYS	A	49	-19.056	10.337	21.614	1.00	0.00	C
ATOM	799	O	LYS	A	49	-19.407	10.553	22.757	1.00	0.00	O
ATOM	800	N	SER	A	50	-17.842	9.872	21.294	1.00	0.00	N
ATOM	801	HN	SER	A	50	-17.624	9.742	20.327	1.00	0.00	
ATOM	802	CA	SER	A	50	-16.818	9.545	22.285	1.00	0.00	C
ATOM	803	HA	SER	A	50	-17.268	9.550	23.178	1.00	0.00	
ATOM	804	CB	SER	A	50	-16.266	8.170	22.041	1.00	0.00	C
ATOM	805	HB1	SER	A	50	-15.813	7.830	22.865	1.00	0.00	
ATOM	806	HB2	SER	A	50	-17.002	7.545	21.783	1.00	0.00	
ATOM	807	OG	SER	A	50	-15.305	8.186	20.981	1.00	0.00	O
ATOM	808	HG1	SER	A	50	-14.952	7.261	20.837	1.00	0.00	
ATOM	809	C	SER	A	50	-15.697	10.586	22.233	1.00	0.00	C
ATOM	810	O	SER	A	50	-15.669	11.429	21.349	1.00	0.00	O
ATOM	811	N	GLN	A	51	-14.725	10.441	23.121	1.00	0.00	N
ATOM	812	HN	GLN	A	51	-14.806	9.731	23.821	1.00	0.00	
ATOM	813	CA	GLN	A	51	-13.573	11.266	23.105	1.00	0.00	C
ATOM	814	HA	GLN	A	51	-13.781	12.036	22.502	1.00	0.00	
ATOM	815	CB	GLN	A	51	-13.320	11.769	24.518	1.00	0.00	C
ATOM	816	HB1	GLN	A	51	-13.129	10.985	25.109	1.00	0.00	
ATOM	817	HB2	GLN	A	51	-12.527	12.378	24.505	1.00	0.00	
ATOM	818	CG	GLN	A	51	-14.510	12.529	25.083	1.00	0.00	C
ATOM	819	HG1	GLN	A	51	-14.741	13.284	24.469	1.00	0.00	
ATOM	820	HG2	GLN	A	51	-15.291	11.909	25.158	1.00	0.00	
ATOM	821	CD	GLN	A	51	-14.195	13.088	26.450	1.00	0.00	C
ATOM	822	OE1	GLN	A	51	-13.404	12.517	27.212	1.00	0.00	O
ATOM	823	NE2	GLN	A	51	-14.796	14.234	26.759	1.00	0.00	N
ATOM	824	1HE2	GLN	A	51	-15.411	14.669	26.102	1.00	0.00	
ATOM	825	2HE2	GLN	A	51	-14.632	14.660	27.649	1.00	0.00	
ATOM	826	C	GLN	A	51	-12.380	10.498	22.529	1.00	0.00	C
ATOM	827	O	GLN	A	51	-11.247	10.969	22.658	1.00	0.00	O
ATOM	828	N	ILE	A	52	-12.652	9.399	21.812	1.00	0.00	N
ATOM	829	HN	ILE	A	52	-13.607	9.134	21.676	1.00	0.00	
ATOM	830	CA	ILE	A	52	-11.607	8.580	21.226	1.00	0.00	C
ATOM	831	HA	ILE	A	52	-10.903	8.507	21.933	1.00	0.00	
ATOM	832	CB	ILE	A	52	-12.107	7.157	20.886	1.00	0.00	C
ATOM	833	HB	ILE	A	52	-12.858	7.251	20.233	1.00	0.00	
ATOM	834	CG2	ILE	A	52	-11.001	6.341	20.230	1.00	0.00	C
ATOM	835	1HG2	ILE	A	52	-11.343	5.426	20.018	1.00	0.00	
ATOM	836	2HG2	ILE	A	52	-10.710	6.792	19.386	1.00	0.00	
ATOM	837	3HG2	ILE	A	52	-10.224	6.270	20.855	1.00	0.00	
ATOM	838	CG1	ILE	A	52	-12.692	6.422	22.090	1.00	0.00	C
ATOM	839	1HG1	ILE	A	52	-13.504	6.915	22.402	1.00	0.00	
ATOM	840	2HG1	ILE	A	52	-12.951	5.499	21.807	1.00	0.00	
ATOM	841	CD	ILE	A	52	-11.750	6.301	23.253	1.00	0.00	C
ATOM	842	HD1	ILE	A	52	-12.202	5.811	23.998	1.00	0.00	
ATOM	843	HD2	ILE	A	52	-10.934	5.798	22.970	1.00	0.00	
ATOM	844	HD3	ILE	A	52	-11.487	7.214	23.565	1.00	0.00	
ATOM	845	C	ILE	A	52	-11.065	9.288	19.973	1.00	0.00	C
ATOM	846	O	ILE	A	52	-11.844	9.715	19.099	1.00	0.00	O
ATOM	847	N	LEU	A	53	-9.726	9.353	19.881	1.00	0.00	N

ATOM	848	HN	LEU	A	53	-9.191	9.008	20.652	1.00	0.00	
ATOM	849	CA	LEU	A	53	-9.013	9.877	18.765	1.00	0.00	C
ATOM	850	HA	LEU	A	53	-9.676	10.365	18.197	1.00	0.00	
ATOM	851	CB	LEU	A	53	-7.937	10.827	19.291	1.00	0.00	C
ATOM	852	HB1	LEU	A	53	-7.242	10.282	19.760	1.00	0.00	
ATOM	853	HB2	LEU	A	53	-7.523	11.293	18.509	1.00	0.00	
ATOM	854	CG	LEU	A	53	-8.438	11.889	20.262	1.00	0.00	C
ATOM	855	HG	LEU	A	53	-8.825	11.425	21.059	1.00	0.00	
ATOM	856	CD1	LEU	A	53	-7.295	12.770	20.723	1.00	0.00	C
ATOM	857	1HD1	LEU	A	53	-7.641	13.460	21.359	1.00	0.00	
ATOM	858	2HD1	LEU	A	53	-6.606	12.209	21.183	1.00	0.00	
ATOM	859	3HD1	LEU	A	53	-6.883	13.222	19.932	1.00	0.00	
ATOM	860	CD2	LEU	A	53	-9.525	12.738	19.623	1.00	0.00	C
ATOM	861	1HD2	LEU	A	53	-9.838	13.427	20.277	1.00	0.00	
ATOM	862	2HD2	LEU	A	53	-9.159	13.192	18.811	1.00	0.00	
ATOM	863	3HD2	LEU	A	53	-10.293	12.154	19.361	1.00	0.00	
ATOM	864	C	LEU	A	53	-8.396	8.730	17.963	1.00	0.00	C
ATOM	865	O	LEU	A	53	-7.221	8.430	18.088	1.00	0.00	O
ATOM	866	N	LEU	A	54	-9.192	8.156	17.067	1.00	0.00	N
ATOM	867	HN	LEU	A	54	-10.123	8.504	16.958	1.00	0.00	
ATOM	868	CA	LEU	A	54	-8.766	7.052	16.247	1.00	0.00	C
ATOM	869	HA	LEU	A	54	-8.397	6.362	16.870	1.00	0.00	
ATOM	870	CB	LEU	A	54	-9.976	6.495	15.481	1.00	0.00	C
ATOM	871	HB1	LEU	A	54	-10.678	6.240	16.146	1.00	0.00	
ATOM	872	HB2	LEU	A	54	-10.328	7.215	14.883	1.00	0.00	
ATOM	873	CG	LEU	A	54	-9.673	5.270	14.619	1.00	0.00	C
ATOM	874	HG	LEU	A	54	-8.890	5.476	14.032	1.00	0.00	
ATOM	875	CD1	LEU	A	54	-9.301	4.094	15.504	1.00	0.00	C
ATOM	876	1HD1	LEU	A	54	-9.104	3.297	14.934	1.00	0.00	
ATOM	877	2HD1	LEU	A	54	-8.492	4.326	16.044	1.00	0.00	
ATOM	878	3HD1	LEU	A	54	-10.062	3.885	16.118	1.00	0.00	
ATOM	879	CD2	LEU	A	54	-10.839	4.932	13.695	1.00	0.00	C
ATOM	880	1HD2	LEU	A	54	-10.608	4.128	13.147	1.00	0.00	
ATOM	881	2HD2	LEU	A	54	-11.653	4.739	14.243	1.00	0.00	
ATOM	882	3HD2	LEU	A	54	-11.020	5.707	13.090	1.00	0.00	
ATOM	883	C	LEU	A	54	-7.673	7.519	15.278	1.00	0.00	C
ATOM	884	O	LEU	A	54	-6.742	6.769	14.971	1.00	0.00	O
ATOM	885	N	GLY	A	55	-7.828	8.742	14.764	1.00	0.00	N
ATOM	886	HN	GLY	A	55	-8.669	9.243	14.970	1.00	0.00	
ATOM	887	CA	GLY	A	55	-6.840	9.372	13.927	1.00	0.00	C
ATOM	888	HA1	GLY	A	55	-6.840	8.898	13.047	1.00	0.00	
ATOM	889	HA2	GLY	A	55	-7.117	10.324	13.793	1.00	0.00	
ATOM	890	C	GLY	A	55	-5.454	9.316	14.548	1.00	0.00	C
ATOM	891	O	GLY	A	55	-4.475	9.040	13.872	1.00	0.00	O
ATOM	892	N	LYS	A	56	-5.384	9.558	15.856	1.00	0.00	N
ATOM	893	HN	LYS	A	56	-6.225	9.772	16.352	1.00	0.00	
ATOM	894	CA	LYS	A	56	-4.134	9.523	16.585	1.00	0.00	C
ATOM	895	HA	LYS	A	56	-3.514	10.103	16.057	1.00	0.00	
ATOM	896	CB	LYS	A	56	-4.321	10.089	17.987	1.00	0.00	C
ATOM	897	HB1	LYS	A	56	-4.707	11.008	17.907	1.00	0.00	
ATOM	898	HB2	LYS	A	56	-4.961	9.501	18.481	1.00	0.00	
ATOM	899	CG	LYS	A	56	-3.033	10.179	18.794	1.00	0.00	C
ATOM	900	HG1	LYS	A	56	-2.498	9.347	18.647	1.00	0.00	
ATOM	901	HG2	LYS	A	56	-2.509	10.972	18.483	1.00	0.00	
ATOM	902	CD	LYS	A	56	-3.318	10.327	20.280	1.00	0.00	C
ATOM	903	HD1	LYS	A	56	-2.460	10.537	20.749	1.00	0.00	
ATOM	904	HD2	LYS	A	56	-3.960	11.083	20.407	1.00	0.00	
ATOM	905	CE	LYS	A	56	-3.915	9.078	20.904	1.00	0.00	C
ATOM	906	HE1	LYS	A	56	-4.757	8.834	20.422	1.00	0.00	
ATOM	907	HE2	LYS	A	56	-3.261	8.325	20.835	1.00	0.00	
ATOM	908	NZ	LYS	A	56	-4.239	9.283	22.331	1.00	0.00	N
ATOM	909	HZ1	LYS	A	56	-4.629	8.443	22.708	1.00	0.00	
ATOM	910	HZ2	LYS	A	56	-3.406	9.518	22.832	1.00	0.00	
ATOM	911	HZ3	LYS	A	56	-4.902	10.027	22.419	1.00	0.00	
ATOM	912	C	LYS	A	56	-3.583	8.094	16.666	1.00	0.00	C
ATOM	913	O	LYS	A	56	-2.380	7.888	16.434	1.00	0.00	O
ATOM	914	N	GLU	A	57	-4.442	7.127	17.030	1.00	0.00	N
ATOM	915	HN	GLU	A	57	-5.391	7.368	17.231	1.00	0.00	
ATOM	916	CA	GLU	A	57	-4.022	5.730	17.139	1.00	0.00	C
ATOM	917	HA	GLU	A	57	-3.325	5.702	17.855	1.00	0.00	
ATOM	918	CB	GLU	A	57	-5.192	4.832	17.561	1.00	0.00	C

ATOM	919	HB1	GLU	A	57	-5.950	4.977	16.925	1.00	0.00	
ATOM	920	HB2	GLU	A	57	-4.895	3.878	17.513	1.00	0.00	
ATOM	921	CG	GLU	A	57	-5.697	5.100	18.970	1.00	0.00	C
ATOM	922	HG1	GLU	A	57	-5.044	4.704	19.615	1.00	0.00	
ATOM	923	HG2	GLU	A	57	-5.738	6.090	19.103	1.00	0.00	
ATOM	924	CD	GLU	A	57	-7.071	4.525	19.275	1.00	0.00	C
ATOM	925	OE1	GLU	A	57	-7.334	3.403	18.822	1.00	0.00	O
ATOM	926	OE2	GLU	A	57	-7.902	5.231	19.911	1.00	0.00	O
ATOM	927	C	GLU	A	57	-3.437	5.272	15.793	1.00	0.00	C
ATOM	928	O	GLU	A	57	-2.404	4.607	15.756	1.00	0.00	O
ATOM	929	N	ILE	A	58	-4.103	5.659	14.697	1.00	0.00	N
ATOM	930	HN	ILE	A	58	-4.909	6.241	14.805	1.00	0.00	
ATOM	931	CA	ILE	A	58	-3.699	5.265	13.356	1.00	0.00	C
ATOM	932	HA	ILE	A	58	-3.597	4.271	13.400	1.00	0.00	
ATOM	933	CB	ILE	A	58	-4.773	5.602	12.311	1.00	0.00	C
ATOM	934	HB	ILE	A	58	-5.022	6.566	12.402	1.00	0.00	
ATOM	935	CG2	ILE	A	58	-4.208	5.379	10.910	1.00	0.00	C
ATOM	936	1HG2	ILE	A	58	-4.907	5.598	10.230	1.00	0.00	
ATOM	937	2HG2	ILE	A	58	-3.413	5.970	10.773	1.00	0.00	
ATOM	938	3HG2	ILE	A	58	-3.934	4.423	10.810	1.00	0.00	
ATOM	939	CG1	ILE	A	58	-6.058	4.791	12.537	1.00	0.00	C
ATOM	940	1HG1	ILE	A	58	-5.875	3.832	12.323	1.00	0.00	
ATOM	941	2HG1	ILE	A	58	-6.326	4.873	13.497	1.00	0.00	
ATOM	942	CD	ILE	A	58	-7.222	5.230	11.701	1.00	0.00	C
ATOM	943	HD1	ILE	A	58	-8.015	4.656	11.906	1.00	0.00	
ATOM	944	HD2	ILE	A	58	-7.439	6.184	11.907	1.00	0.00	
ATOM	945	HD3	ILE	A	58	-6.989	5.143	10.733	1.00	0.00	
ATOM	946	C	ILE	A	58	-2.362	5.913	12.999	1.00	0.00	C
ATOM	947	O	ILE	A	58	-1.497	5.266	12.422	1.00	0.00	O
ATOM	948	N	LYS	A	59	-2.217	7.194	13.320	1.00	0.00	N
ATOM	949	HN	LYS	A	59	-2.964	7.662	13.792	1.00	0.00	
ATOM	950	CA	LYS	A	59	-1.003	7.940	13.007	1.00	0.00	C
ATOM	951	HA	LYS	A	59	-0.859	7.873	12.020	1.00	0.00	
ATOM	952	CB	LYS	A	59	-1.178	9.412	13.373	1.00	0.00	C
ATOM	953	HB1	LYS	A	59	-1.857	9.804	12.753	1.00	0.00	
ATOM	954	HB2	LYS	A	59	-1.519	9.457	14.312	1.00	0.00	
ATOM	955	CG	LYS	A	59	0.077	10.271	13.292	1.00	0.00	C
ATOM	956	HG1	LYS	A	59	0.770	9.891	13.905	1.00	0.00	
ATOM	957	HG2	LYS	A	59	0.419	10.257	12.352	1.00	0.00	
ATOM	958	CD	LYS	A	59	-0.206	11.715	13.697	1.00	0.00	C
ATOM	959	HD1	LYS	A	59	-0.795	12.133	13.005	1.00	0.00	
ATOM	960	HD2	LYS	A	59	-0.676	11.715	14.580	1.00	0.00	
ATOM	961	CE	LYS	A	59	1.042	12.556	13.826	1.00	0.00	C
ATOM	962	HE1	LYS	A	59	0.886	13.285	14.493	1.00	0.00	
ATOM	963	HE2	LYS	A	59	1.801	11.982	14.131	1.00	0.00	
ATOM	964	NZ	LYS	A	59	1.409	13.172	12.535	1.00	0.00	N
ATOM	965	HZ1	LYS	A	59	2.237	13.721	12.651	1.00	0.00	
ATOM	966	HZ2	LYS	A	59	1.577	12.454	11.859	1.00	0.00	
ATOM	967	HZ3	LYS	A	59	0.662	13.757	12.221	1.00	0.00	
ATOM	968	C	LYS	A	59	0.180	7.310	13.743	1.00	0.00	C
ATOM	969	O	LYS	A	59	1.254	7.170	13.191	1.00	0.00	O
ATOM	970	N	ILE	A	60	-0.044	6.910	14.993	1.00	0.00	N
ATOM	971	HN	ILE	A	60	-0.966	6.991	15.372	1.00	0.00	
ATOM	972	CA	ILE	A	60	1.016	6.358	15.823	1.00	0.00	C
ATOM	973	HA	ILE	A	60	1.818	6.942	15.699	1.00	0.00	
ATOM	974	CB	ILE	A	60	0.604	6.417	17.296	1.00	0.00	C
ATOM	975	HB	ILE	A	60	-0.350	6.129	17.385	1.00	0.00	
ATOM	976	CG2	ILE	A	60	1.417	5.446	18.116	1.00	0.00	C
ATOM	977	1HG2	ILE	A	60	1.134	5.499	19.074	1.00	0.00	
ATOM	978	2HG2	ILE	A	60	1.269	4.517	17.776	1.00	0.00	
ATOM	979	3HG2	ILE	A	60	2.387	5.678	18.042	1.00	0.00	
ATOM	980	CG1	ILE	A	60	0.710	7.849	17.825	1.00	0.00	C
ATOM	981	1HG1	ILE	A	60	1.677	8.078	17.933	1.00	0.00	
ATOM	982	2HG1	ILE	A	60	0.294	8.467	17.157	1.00	0.00	
ATOM	983	CD	ILE	A	60	0.026	8.069	19.155	1.00	0.00	C
ATOM	984	HD1	ILE	A	60	0.138	9.023	19.434	1.00	0.00	
ATOM	985	HD2	ILE	A	60	-0.948	7.859	19.068	1.00	0.00	
ATOM	986	HD3	ILE	A	60	0.435	7.470	19.843	1.00	0.00	
ATOM	987	C	ILE	A	60	1.372	4.934	15.353	1.00	0.00	C
ATOM	988	O	ILE	A	60	2.534	4.576	15.291	1.00	0.00	O
ATOM	989	N	LEU	A	61	0.364	4.132	15.018	1.00	0.00	N

```
ATOM    990  HN  LEU A  61      -0.574   4.471  15.087  1.00  0.00
ATOM    991  CA  LEU A  61       0.597   2.766  14.551  1.00  0.00           C
ATOM    992  HA  LEU A  61       1.156   2.310  15.244  1.00  0.00
ATOM    993  CB  LEU A  61      -0.747   2.048  14.421  1.00  0.00           C
ATOM    994  HB1 LEU A  61      -1.396   2.681  13.999  1.00  0.00
ATOM    995  HB2 LEU A  61      -0.618   1.256  13.824  1.00  0.00
ATOM    996  CG  LEU A  61      -1.356   1.554  15.732  1.00  0.00           C
ATOM    997  HG  LEU A  61      -1.397   2.338  16.352  1.00  0.00
ATOM    998  CD1 LEU A  61      -2.770   1.054  15.521  1.00  0.00           C
ATOM    999 1HD1 LEU A  61      -3.145   0.737  16.392  1.00  0.00
ATOM   1000 2HD1 LEU A  61      -3.337   1.797  15.165  1.00  0.00
ATOM   1001 3HD1 LEU A  61      -2.762   0.298  14.867  1.00  0.00
ATOM   1002  CD2 LEU A  61      -0.494   0.477  16.397  1.00  0.00           C
ATOM   1003 1HD2 LEU A  61      -0.927   0.182  17.249  1.00  0.00
ATOM   1004 2HD2 LEU A  61      -0.404  -0.305  15.781  1.00  0.00
ATOM   1005 3HD2 LEU A  61       0.412   0.851  16.596  1.00  0.00
ATOM   1006  C   LEU A  61       1.367   2.776  13.213  1.00  0.00           C
ATOM   1007  O   LEU A  61       2.213   1.936  12.993  1.00  0.00           O
ATOM   1008  N   LYS A  62       1.055   3.724  12.326  1.00  0.00           N
ATOM   1009  HN  LYS A  62       0.329   4.373  12.555  1.00  0.00
ATOM   1010  CA  LYS A  62       1.734   3.857  11.022  1.00  0.00           C
ATOM   1011  HA  LYS A  62       1.642   2.971  10.568  1.00  0.00
ATOM   1012  CB  LYS A  62       1.076   4.940  10.165  1.00  0.00           C
ATOM   1013  HB1 LYS A  62       0.861   5.716  10.758  1.00  0.00
ATOM   1014  HB2 LYS A  62       1.734   5.225   9.468  1.00  0.00
ATOM   1015  CG  LYS A  62      -0.204   4.534   9.453  1.00  0.00           C
ATOM   1016  HG1 LYS A  62      -0.019   3.708   8.920  1.00  0.00
ATOM   1017  HG2 LYS A  62      -0.899   4.336  10.144  1.00  0.00
ATOM   1018  CD  LYS A  62      -0.753   5.594   8.514  1.00  0.00           C
ATOM   1019  HD1 LYS A  62      -1.547   5.216   8.037  1.00  0.00
ATOM   1020  HD2 LYS A  62      -1.036   6.384   9.057  1.00  0.00
ATOM   1021  CE  LYS A  62       0.256   6.057   7.479  1.00  0.00           C
ATOM   1022  HE1 LYS A  62       0.832   6.774   7.872  1.00  0.00
ATOM   1023  HE2 LYS A  62       0.827   5.285   7.201  1.00  0.00
ATOM   1024  NZ  LYS A  62      -0.411   6.601   6.276  1.00  0.00           N
ATOM   1025  HZ1 LYS A  62       0.280   6.896   5.616  1.00  0.00
ATOM   1026  HZ2 LYS A  62      -0.984   5.893   5.864  1.00  0.00
ATOM   1027  HZ3 LYS A  62      -0.980   7.382   6.535  1.00  0.00
ATOM   1028  C   LYS A  62       3.207   4.217  11.246  1.00  0.00           C
ATOM   1029  O   LYS A  62       4.079   3.744  10.560  1.00  0.00           O
ATOM   1030  N   GLU A  63       3.455   5.079  12.228  1.00  0.00           N
ATOM   1031  HN  GLU A  63       2.692   5.385  12.797  1.00  0.00
ATOM   1032  CA  GLU A  63       4.778   5.596  12.513  1.00  0.00           C
ATOM   1033  HA  GLU A  63       5.185   5.928  11.662  1.00  0.00
ATOM   1034  CB  GLU A  63       4.638   6.773  13.483  1.00  0.00           C
ATOM   1035  HB1 GLU A  63       3.928   7.387  13.138  1.00  0.00
ATOM   1036  HB2 GLU A  63       4.366   6.418  14.378  1.00  0.00
ATOM   1037  CG  GLU A  63       5.896   7.581  13.670  1.00  0.00           C
ATOM   1038  HG1 GLU A  63       6.519   7.048  14.242  1.00  0.00
ATOM   1039  HG2 GLU A  63       6.307   7.719  12.769  1.00  0.00
ATOM   1040  CD  GLU A  63       5.693   8.947  14.322  1.00  0.00           C
ATOM   1041  OE1 GLU A  63       4.549   9.426  14.368  1.00  0.00           O
ATOM   1042  OE2 GLU A  63       6.694   9.539  14.773  1.00  0.00           O
ATOM   1043  C   GLU A  63       5.652   4.455  13.059  1.00  0.00           C
ATOM   1044  O   GLU A  63       6.831   4.434  12.857  1.00  0.00           O
ATOM   1045  N   LEU A  64       5.035   3.525  13.788  1.00  0.00           N
ATOM   1046  HN  LEU A  64       4.050   3.608  13.936  1.00  0.00
ATOM   1047  CA  LEU A  64       5.734   2.397  14.375  1.00  0.00           C
ATOM   1048  HA  LEU A  64       6.678   2.677  14.549  1.00  0.00
ATOM   1049  CB  LEU A  64       5.058   2.032  15.695  1.00  0.00           C
ATOM   1050  HB1 LEU A  64       4.121   1.743  15.497  1.00  0.00
ATOM   1051  HB2 LEU A  64       5.562   1.272  16.105  1.00  0.00
ATOM   1052  CG  LEU A  64       5.003   3.173  16.711  1.00  0.00           C
ATOM   1053  HG  LEU A  64       4.646   3.973  16.228  1.00  0.00
ATOM   1054  CD1 LEU A  64       4.053   2.873  17.857  1.00  0.00           C
ATOM   1055 1HD1 LEU A  64       4.048   3.642  18.496  1.00  0.00
ATOM   1056 2HD1 LEU A  64       3.131   2.732  17.497  1.00  0.00
ATOM   1057 3HD1 LEU A  64       4.355   2.047  18.333  1.00  0.00
ATOM   1058  CD2 LEU A  64       6.387   3.517  17.243  1.00  0.00           C
ATOM   1059 1HD2 LEU A  64       6.314   4.265  17.903  1.00  0.00
ATOM   1060 2HD2 LEU A  64       6.781   2.714  17.691  1.00  0.00
```

```
ATOM   1061 3HD2 LEU A  64       6.975   3.798  16.485  1.00  0.00
ATOM   1062  C   LEU A  64       5.724   1.223  13.387  1.00  0.00           C
ATOM   1063  O   LEU A  64       4.734   0.968  12.735  1.00  0.00           O
ATOM   1064  N   GLN A  65       6.873   0.570  13.237  1.00  0.00           N
ATOM   1065  HN  GLN A  65       7.692   0.914  13.697  1.00  0.00
ATOM   1066  CA  GLN A  65       6.970  -0.607  12.439  1.00  0.00           C
ATOM   1067  HA  GLN A  65       6.070  -1.042  12.472  1.00  0.00
ATOM   1068  CB  GLN A  65       7.261  -0.283  10.984  1.00  0.00           C
ATOM   1069  HB1 GLN A  65       6.612   0.410  10.672  1.00  0.00
ATOM   1070  HB2 GLN A  65       8.192   0.076  10.913  1.00  0.00
ATOM   1071  CG  GLN A  65       7.135  -1.518  10.097  1.00  0.00           C
ATOM   1072  HG1 GLN A  65       7.799  -2.197  10.409  1.00  0.00
ATOM   1073  HG2 GLN A  65       6.210  -1.884  10.199  1.00  0.00
ATOM   1074  CD  GLN A  65       7.375  -1.287   8.629  1.00  0.00           C
ATOM   1075  OE1 GLN A  65       8.129  -2.031   8.004  1.00  0.00           O
ATOM   1076  NE2 GLN A  65       6.708  -0.282   8.063  1.00  0.00           N
ATOM   1077 1HE2 GLN A  65       6.084   0.276   8.611  1.00  0.00
ATOM   1078 2HE2 GLN A  65       6.831  -0.085   7.090  1.00  0.00
ATOM   1079  C   GLN A  65       8.043  -1.495  13.049  1.00  0.00           C
ATOM   1080  O   GLN A  65       9.202  -1.329  12.785  1.00  0.00           O
ATOM   1081  N   HIS A  66       7.607  -2.397  13.920  1.00  0.00           N
ATOM   1082  HN  HIS A  66       6.622  -2.556  13.992  1.00  0.00
ATOM   1083  CA  HIS A  66       8.475  -3.142  14.750  1.00  0.00           C
ATOM   1084  HA  HIS A  66       9.299  -3.313  14.209  1.00  0.00
ATOM   1085  CB  HIS A  66       8.845  -2.325  15.993  1.00  0.00           C
ATOM   1086  HB1 HIS A  66       9.101  -1.398  15.719  1.00  0.00
ATOM   1087  HB2 HIS A  66       8.061  -2.285  16.612  1.00  0.00
ATOM   1088  ND1 HIS A  66       9.816  -3.863  17.740  1.00  0.00           N
ATOM   1089  CG  HIS A  66       9.988  -2.921  16.732  1.00  0.00           C
ATOM   1090  CE1 HIS A  66      10.998  -4.238  18.168  1.00  0.00           C
ATOM   1091  HE1 HIS A  66      11.175  -4.905  18.892  1.00  0.00
ATOM   1092  NE2 HIS A  66      11.931  -3.570  17.465  1.00  0.00           N
ATOM   1093  HE2 HIS A  66      12.920  -3.659  17.583  1.00  0.00
ATOM   1094  CD2 HIS A  66      11.316  -2.757  16.572  1.00  0.00           C
ATOM   1095  HD2 HIS A  66      11.764  -2.149  15.917  1.00  0.00
ATOM   1096  C   HIS A  66       7.797  -4.460  15.109  1.00  0.00           C
ATOM   1097  O   HIS A  66       6.609  -4.516  15.256  1.00  0.00           O
ATOM   1098  N   GLU A  67       8.603  -5.499  15.283  1.00  0.00           N
ATOM   1099  HN  GLU A  67       9.590  -5.339  15.271  1.00  0.00
ATOM   1100  CA  GLU A  67       8.125  -6.867  15.491  1.00  0.00           C
ATOM   1101  HA  GLU A  67       7.435  -7.020  14.783  1.00  0.00
ATOM   1102  CB  GLU A  67       9.272  -7.871  15.320  1.00  0.00           C
ATOM   1103  HB1 GLU A  67       8.913  -8.794  15.461  1.00  0.00
ATOM   1104  HB2 GLU A  67       9.630  -7.792  14.390  1.00  0.00
ATOM   1105  CG  GLU A  67      10.420  -7.651  16.294  1.00  0.00           C
ATOM   1106  HG1 GLU A  67      10.541  -6.666  16.416  1.00  0.00
ATOM   1107  HG2 GLU A  67      10.170  -8.067  17.169  1.00  0.00
ATOM   1108  CD  GLU A  67      11.750  -8.235  15.862  1.00  0.00           C
ATOM   1109  OE1 GLU A  67      11.750  -9.429  15.517  1.00  0.00           O
ATOM   1110  OE2 GLU A  67      12.774  -7.481  15.860  1.00  0.00           O
ATOM   1111  C   GLU A  67       7.476  -7.014  16.872  1.00  0.00           C
ATOM   1112  O   GLU A  67       6.787  -7.986  17.107  1.00  0.00           O
ATOM   1113  N   ASN A  68       7.709  -6.055  17.775  1.00  0.00           N
ATOM   1114  HN  ASN A  68       8.257  -5.265  17.502  1.00  0.00
ATOM   1115  CA  ASN A  68       7.194  -6.112  19.152  1.00  0.00           C
ATOM   1116  HA  ASN A  68       6.782  -7.016  19.267  1.00  0.00
ATOM   1117  CB  ASN A  68       8.328  -5.975  20.160  1.00  0.00           C
ATOM   1118  HB1 ASN A  68       8.855  -5.153  19.944  1.00  0.00
ATOM   1119  HB2 ASN A  68       7.937  -5.889  21.076  1.00  0.00
ATOM   1120  CG  ASN A  68       9.263  -7.167  20.143  1.00  0.00           C
ATOM   1121  OD1 ASN A  68      10.465  -7.005  19.943  1.00  0.00           O
ATOM   1122  ND2 ASN A  68       8.721  -8.364  20.356  1.00  0.00           N
ATOM   1123 1HD2 ASN A  68       7.738  -8.446  20.518  1.00  0.00
ATOM   1124 2HD2 ASN A  68       9.297  -9.181  20.354  1.00  0.00
ATOM   1125  C   ASN A  68       6.126  -5.032  19.359  1.00  0.00           C
ATOM   1126  O   ASN A  68       5.755  -4.699  20.503  1.00  0.00           O
ATOM   1127  N   ILE A  69       5.621  -4.492  18.238  1.00  0.00           N
ATOM   1128  HN  ILE A  69       6.018  -4.758  17.359  1.00  0.00
ATOM   1129  CA  ILE A  69       4.522  -3.537  18.244  1.00  0.00           C
ATOM   1130  HA  ILE A  69       4.120  -3.542  19.159  1.00  0.00
ATOM   1131  CB  ILE A  69       5.048  -2.123  17.942  1.00  0.00           C
```

ATOM	1132	HB	ILE	A	69	5.513	-2.129	17.057	1.00	0.00	
ATOM	1133	CG2	ILE	A	69	3.884	-1.146	17.836	1.00	0.00	C
ATOM	1134	1HG2	ILE	A	69	4.234	-0.230	17.640	1.00	0.00	
ATOM	1135	2HG2	ILE	A	69	3.273	-1.434	17.099	1.00	0.00	
ATOM	1136	3HG2	ILE	A	69	3.381	-1.132	18.700	1.00	0.00	
ATOM	1137	CG1	ILE	A	69	6.096	-1.678	18.967	1.00	0.00	C
ATOM	1138	1HG1	ILE	A	69	5.703	-1.759	19.883	1.00	0.00	
ATOM	1139	2HG1	ILE	A	69	6.892	-2.278	18.892	1.00	0.00	
ATOM	1140	CD	ILE	A	69	6.562	-0.250	18.778	1.00	0.00	C
ATOM	1141	HD1	ILE	A	69	7.242	-0.026	19.477	1.00	0.00	
ATOM	1142	HD2	ILE	A	69	6.971	-0.150	17.871	1.00	0.00	
ATOM	1143	HD3	ILE	A	69	5.781	0.369	18.862	1.00	0.00	
ATOM	1144	C	ILE	A	69	3.485	-3.988	17.214	1.00	0.00	C
ATOM	1145	O	ILE	A	69	3.796	-4.189	16.035	1.00	0.00	O
ATOM	1146	N	VAL	A	70	2.241	-4.100	17.652	1.00	0.00	N
ATOM	1147	HN	VAL	A	70	2.021	-3.775	18.572	1.00	0.00	
ATOM	1148	CA	VAL	A	70	1.198	-4.672	16.852	1.00	0.00	C
ATOM	1149	HA	VAL	A	70	1.465	-5.621	16.685	1.00	0.00	
ATOM	1150	CB	VAL	A	70	-0.150	-4.702	17.595	1.00	0.00	C
ATOM	1151	HB	VAL	A	70	0.007	-5.090	18.503	1.00	0.00	
ATOM	1152	CG1	VAL	A	70	-0.717	-3.302	17.786	1.00	0.00	C
ATOM	1153	1HG1	VAL	A	70	-1.590	-3.359	18.270	1.00	0.00	
ATOM	1154	2HG1	VAL	A	70	-0.075	-2.752	18.320	1.00	0.00	
ATOM	1155	3HG1	VAL	A	70	-0.859	-2.875	16.893	1.00	0.00	
ATOM	1156	CG2	VAL	A	70	-1.149	-5.594	16.888	1.00	0.00	C
ATOM	1157	1HG2	VAL	A	70	-2.012	-5.593	17.393	1.00	0.00	
ATOM	1158	2HG2	VAL	A	70	-1.304	-5.252	15.961	1.00	0.00	
ATOM	1159	3HG2	VAL	A	70	-0.790	-6.526	16.843	1.00	0.00	
ATOM	1160	C	VAL	A	70	1.131	-3.894	15.541	1.00	0.00	C
ATOM	1161	O	VAL	A	70	1.115	-2.671	15.548	1.00	0.00	O
ATOM	1162	N	ALA	A	71	1.164	-4.643	14.416	1.00	0.00	N
ATOM	1163	HN	ALA	A	71	1.176	-5.638	14.511	1.00	0.00	
ATOM	1164	CA	ALA	A	71	1.183	-4.089	13.097	1.00	0.00	C
ATOM	1165	HA	ALA	A	71	1.801	-3.303	13.117	1.00	0.00	
ATOM	1166	CB	ALA	A	71	1.716	-5.134	12.149	1.00	0.00	C
ATOM	1167	HB1	ALA	A	71	1.736	-4.763	11.221	1.00	0.00	
ATOM	1168	HB2	ALA	A	71	2.642	-5.393	12.425	1.00	0.00	
ATOM	1169	HB3	ALA	A	71	1.124	-5.939	12.173	1.00	0.00	
ATOM	1170	C	ALA	A	71	-0.218	-3.619	12.697	1.00	0.00	C
ATOM	1171	O	ALA	A	71	-1.179	-4.278	12.970	1.00	0.00	O
ATOM	1172	N	LEU	A	72	-0.296	-2.463	12.037	1.00	0.00	N
ATOM	1173	HN	LEU	A	72	0.498	-1.856	12.060	1.00	0.00	
ATOM	1174	CA	LEU	A	72	-1.463	-2.031	11.287	1.00	0.00	C
ATOM	1175	HA	LEU	A	72	-2.234	-2.612	11.547	1.00	0.00	
ATOM	1176	CB	LEU	A	72	-1.785	-0.575	11.631	1.00	0.00	C
ATOM	1177	HB1	LEU	A	72	-2.004	-0.529	12.606	1.00	0.00	
ATOM	1178	HB2	LEU	A	72	-0.970	-0.026	11.446	1.00	0.00	
ATOM	1179	CG	LEU	A	72	-2.944	0.046	10.867	1.00	0.00	C
ATOM	1180	HG	LEU	A	72	-2.762	-0.057	9.889	1.00	0.00	
ATOM	1181	CD1	LEU	A	72	-4.220	-0.669	11.180	1.00	0.00	C
ATOM	1182	1HD1	LEU	A	72	-4.971	-0.249	10.670	1.00	0.00	
ATOM	1183	2HD1	LEU	A	72	-4.136	-1.630	10.918	1.00	0.00	
ATOM	1184	3HD1	LEU	A	72	-4.406	-0.605	12.161	1.00	0.00	
ATOM	1185	CD2	LEU	A	72	-3.085	1.528	11.179	1.00	0.00	C
ATOM	1186	1HD2	LEU	A	72	-3.854	1.906	10.663	1.00	0.00	
ATOM	1187	2HD2	LEU	A	72	-3.250	1.648	12.158	1.00	0.00	
ATOM	1188	3HD2	LEU	A	72	-2.244	2.004	10.922	1.00	0.00	
ATOM	1189	C	LEU	A	72	-1.132	-2.169	9.796	1.00	0.00	C
ATOM	1190	O	LEU	A	72	-0.185	-1.542	9.319	1.00	0.00	O
ATOM	1191	N	TYR	A	73	-1.946	-2.943	9.068	1.00	0.00	N
ATOM	1192	HN	TYR	A	73	-2.742	-3.358	9.509	1.00	0.00	
ATOM	1193	CA	TYR	A	73	-1.710	-3.201	7.651	1.00	0.00	C
ATOM	1194	HA	TYR	A	73	-0.737	-3.012	7.516	1.00	0.00	
ATOM	1195	CB	TYR	A	73	-2.024	-4.655	7.313	1.00	0.00	C
ATOM	1196	HB1	TYR	A	73	-2.984	-4.835	7.529	1.00	0.00	
ATOM	1197	HB2	TYR	A	73	-1.870	-4.798	6.335	1.00	0.00	
ATOM	1198	CG	TYR	A	73	-1.187	-5.647	8.063	1.00	0.00	C
ATOM	1199	CD1	TYR	A	73	0.193	-5.600	7.990	1.00	0.00	C
ATOM	1200	HD1	TYR	A	73	0.631	-4.897	7.430	1.00	0.00	
ATOM	1201	CE1	TYR	A	73	0.980	-6.508	8.677	1.00	0.00	C
ATOM	1202	HE1	TYR	A	73	1.976	-6.474	8.594	1.00	0.00	

ATOM	1203	CZ	TYR	A	73	0.380	-7.465	9.480	1.00	0.00	C
ATOM	1204	OH	TYR	A	73	1.146	-8.348	10.188	1.00	0.00	O
ATOM	1205	HH	TYR	A	73	0.552	-8.962	10.708	1.00	0.00	
ATOM	1206	CD2	TYR	A	73	-1.767	-6.619	8.853	1.00	0.00	C
ATOM	1207	HD2	TYR	A	73	-2.764	-6.667	8.917	1.00	0.00	
ATOM	1208	CE2	TYR	A	73	-0.999	-7.527	9.557	1.00	0.00	C
ATOM	1209	HE2	TYR	A	73	-1.439	-8.226	10.120	1.00	0.00	
ATOM	1210	C	TYR	A	73	-2.561	-2.277	6.767	1.00	0.00	C
ATOM	1211	O	TYR	A	73	-2.141	-1.926	5.692	1.00	0.00	O
ATOM	1212	N	ASP	A	74	-3.770	-1.923	7.220	1.00	0.00	N
ATOM	1213	HN	ASP	A	74	-4.027	-2.125	8.165	1.00	0.00	
ATOM	1214	CA	ASP	A	74	-4.699	-1.254	6.354	1.00	0.00	C
ATOM	1215	HA	ASP	A	74	-4.154	-0.550	5.898	1.00	0.00	
ATOM	1216	CB	ASP	A	74	-5.282	-2.224	5.329	1.00	0.00	C
ATOM	1217	HB1	ASP	A	74	-4.542	-2.802	4.984	1.00	0.00	
ATOM	1218	HB2	ASP	A	74	-5.965	-2.794	5.786	1.00	0.00	
ATOM	1219	CG	ASP	A	74	-5.946	-1.551	4.144	1.00	0.00	C
ATOM	1220	OD1	ASP	A	74	-5.585	-0.408	3.854	1.00	0.00	O
ATOM	1221	OD2	ASP	A	74	-6.781	-2.205	3.486	1.00	0.00	O
ATOM	1222	C	ASP	A	74	-5.824	-0.622	7.165	1.00	0.00	C
ATOM	1223	O	ASP	A	74	-6.193	-1.104	8.250	1.00	0.00	O
ATOM	1224	N	VAL	A	75	-6.356	0.459	6.591	1.00	0.00	N
ATOM	1225	HN	VAL	A	75	-5.924	0.808	5.760	1.00	0.00	
ATOM	1226	CA	VAL	A	75	-7.523	1.161	7.097	1.00	0.00	C
ATOM	1227	HA	VAL	A	75	-7.899	0.616	7.846	1.00	0.00	
ATOM	1228	CB	VAL	A	75	-7.137	2.535	7.656	1.00	0.00	C
ATOM	1229	HB	VAL	A	75	-6.838	3.102	6.889	1.00	0.00	
ATOM	1230	CG1	VAL	A	75	-8.318	3.231	8.299	1.00	0.00	C
ATOM	1231	1HG1	VAL	A	75	-8.029	4.121	8.651	1.00	0.00	
ATOM	1232	2HG1	VAL	A	75	-9.040	3.360	7.619	1.00	0.00	
ATOM	1233	3HG1	VAL	A	75	-8.665	2.672	9.052	1.00	0.00	
ATOM	1234	CG2	VAL	A	75	-5.988	2.420	8.645	1.00	0.00	C
ATOM	1235	1HG2	VAL	A	75	-5.756	3.328	8.994	1.00	0.00	
ATOM	1236	2HG2	VAL	A	75	-6.260	1.830	9.405	1.00	0.00	
ATOM	1237	3HG2	VAL	A	75	-5.192	2.026	8.186	1.00	0.00	
ATOM	1238	C	VAL	A	75	-8.525	1.289	5.945	1.00	0.00	C
ATOM	1239	O	VAL	A	75	-8.167	1.777	4.892	1.00	0.00	O
ATOM	1240	N	GLN	A	76	-9.746	0.778	6.150	1.00	0.00	N
ATOM	1241	HN	GLN	A	76	-9.927	0.288	7.003	1.00	0.00	
ATOM	1242	CA	GLN	A	76	-10.814	0.910	5.180	1.00	0.00	C
ATOM	1243	HA	GLN	A	76	-10.416	1.406	4.408	1.00	0.00	
ATOM	1244	CB	GLN	A	76	-11.316	-0.447	4.677	1.00	0.00	C
ATOM	1245	HB1	GLN	A	76	-11.623	-0.982	5.464	1.00	0.00	
ATOM	1246	HB2	GLN	A	76	-12.087	-0.289	4.060	1.00	0.00	
ATOM	1247	CG	GLN	A	76	-10.277	-1.265	3.930	1.00	0.00	C
ATOM	1248	HG1	GLN	A	76	-9.462	-1.348	4.503	1.00	0.00	
ATOM	1249	HG2	GLN	A	76	-10.653	-2.174	3.751	1.00	0.00	
ATOM	1250	CD	GLN	A	76	-9.873	-0.648	2.612	1.00	0.00	C
ATOM	1251	OE1	GLN	A	76	-10.705	-0.308	1.790	1.00	0.00	O
ATOM	1252	NE2	GLN	A	76	-8.577	-0.488	2.393	1.00	0.00	N
ATOM	1253	1HE2	GLN	A	76	-7.914	-0.771	3.086	1.00	0.00	
ATOM	1254	2HE2	GLN	A	76	-8.261	-0.084	1.535	1.00	0.00	
ATOM	1255	C	GLN	A	76	-11.961	1.675	5.839	1.00	0.00	C
ATOM	1256	O	GLN	A	76	-12.435	1.308	6.894	1.00	0.00	O
ATOM	1257	N	GLU	A	77	-12.395	2.731	5.155	1.00	0.00	N
ATOM	1258	HN	GLU	A	77	-11.945	2.947	4.289	1.00	0.00	
ATOM	1259	CA	GLU	A	77	-13.464	3.573	5.583	1.00	0.00	C
ATOM	1260	HA	GLU	A	77	-13.656	3.370	6.543	1.00	0.00	
ATOM	1261	CB	GLU	A	77	-13.014	5.021	5.450	1.00	0.00	C
ATOM	1262	HB1	GLU	A	77	-12.136	5.122	5.918	1.00	0.00	
ATOM	1263	HB2	GLU	A	77	-12.899	5.227	4.478	1.00	0.00	
ATOM	1264	CG	GLU	A	77	-13.982	6.031	6.036	1.00	0.00	C
ATOM	1265	HG1	GLU	A	77	-14.646	6.273	5.328	1.00	0.00	
ATOM	1266	HG2	GLU	A	77	-14.456	5.599	6.804	1.00	0.00	
ATOM	1267	CD	GLU	A	77	-13.331	7.312	6.537	1.00	0.00	C
ATOM	1268	OE1	GLU	A	77	-12.129	7.269	6.870	1.00	0.00	O
ATOM	1269	OE2	GLU	A	77	-14.023	8.350	6.597	1.00	0.00	O
ATOM	1270	C	GLU	A	77	-14.699	3.268	4.722	1.00	0.00	C
ATOM	1271	O	GLU	A	77	-14.666	3.423	3.514	1.00	0.00	O
ATOM	1272	N	LEU	A	78	-15.770	2.792	5.366	1.00	0.00	N
ATOM	1273	HN	LEU	A	78	-15.650	2.445	6.296	1.00	0.00	

ATOM	1274	CA	LEU	A	78	-17.113	2.753	4.776	1.00	0.00	C
ATOM	1275	HA	LEU	A	78	-17.043	2.854	3.784	1.00	0.00	
ATOM	1276	CB	LEU	A	78	-17.759	1.393	5.068	1.00	0.00	C
ATOM	1277	HB1	LEU	A	78	-17.448	1.091	5.969	1.00	0.00	
ATOM	1278	HB2	LEU	A	78	-18.751	1.519	5.082	1.00	0.00	
ATOM	1279	CG	LEU	A	78	-17.437	0.282	4.059	1.00	0.00	C
ATOM	1280	HG	LEU	A	78	-17.885	0.527	3.199	1.00	0.00	
ATOM	1281	CD1	LEU	A	78	-15.937	0.162	3.820	1.00	0.00	C
ATOM	1282	1HD1	LEU	A	78	-15.762	-0.568	3.160	1.00	0.00	
ATOM	1283	2HD1	LEU	A	78	-15.586	1.027	3.461	1.00	0.00	
ATOM	1284	3HD1	LEU	A	78	-15.478	-0.051	4.682	1.00	0.00	
ATOM	1285	CD2	LEU	A	78	-17.975	-1.059	4.518	1.00	0.00	C
ATOM	1286	1HD2	LEU	A	78	-17.749	-1.759	3.840	1.00	0.00	
ATOM	1287	2HD2	LEU	A	78	-17.563	-1.300	5.397	1.00	0.00	
ATOM	1288	3HD2	LEU	A	78	-18.968	-1.002	4.620	1.00	0.00	
ATOM	1289	C	LEU	A	78	-17.913	3.926	5.346	1.00	0.00	C
ATOM	1290	O	LEU	A	78	-17.424	4.642	6.224	1.00	0.00	O
ATOM	1291	N	PRO	A	79	-19.135	4.225	4.832	1.00	0.00	N
ATOM	1292	CD	PRO	A	79	-19.820	3.506	3.745	1.00	0.00	C
ATOM	1293	HD1	PRO	A	79	-20.359	2.744	4.105	1.00	0.00	
ATOM	1294	HD2	PRO	A	79	-19.162	3.162	3.075	1.00	0.00	
ATOM	1295	CA	PRO	A	79	-19.903	5.378	5.323	1.00	0.00	C
ATOM	1296	HA	PRO	A	79	-19.323	6.192	5.324	1.00	0.00	
ATOM	1297	CB	PRO	A	79	-21.097	5.466	4.356	1.00	0.00	C
ATOM	1298	HB1	PRO	A	79	-21.928	5.114	4.787	1.00	0.00	
ATOM	1299	HB2	PRO	A	79	-21.247	6.411	4.064	1.00	0.00	
ATOM	1300	CG	PRO	A	79	-20.699	4.593	3.164	1.00	0.00	C
ATOM	1301	HG1	PRO	A	79	-21.508	4.193	2.733	1.00	0.00	
ATOM	1302	HG2	PRO	A	79	-20.191	5.127	2.488	1.00	0.00	
ATOM	1303	C	PRO	A	79	-20.365	5.197	6.780	1.00	0.00	C
ATOM	1304	O	PRO	A	79	-20.465	6.169	7.503	1.00	0.00	O
ATOM	1305	N	ASN	A	80	-20.604	3.936	7.177	1.00	0.00	N
ATOM	1306	HN	ASN	A	80	-20.461	3.208	6.506	1.00	0.00	
ATOM	1307	CA	ASN	A	80	-21.062	3.536	8.529	1.00	0.00	C
ATOM	1308	HA	ASN	A	80	-21.639	4.302	8.813	1.00	0.00	
ATOM	1309	CB	ASN	A	80	-21.850	2.223	8.496	1.00	0.00	C
ATOM	1310	HB1	ASN	A	80	-21.267	1.505	8.116	1.00	0.00	
ATOM	1311	HB2	ASN	A	80	-22.113	1.980	9.430	1.00	0.00	
ATOM	1312	CG	ASN	A	80	-23.106	2.313	7.654	1.00	0.00	C
ATOM	1313	OD1	ASN	A	80	-23.861	3.280	7.764	1.00	0.00	O
ATOM	1314	ND2	ASN	A	80	-23.331	1.317	6.808	1.00	0.00	N
ATOM	1315	1HD2	ASN	A	80	-22.686	0.555	6.749	1.00	0.00	
ATOM	1316	2HD2	ASN	A	80	-24.146	1.327	6.229	1.00	0.00	
ATOM	1317	C	ASN	A	80	-19.890	3.331	9.499	1.00	0.00	C
ATOM	1318	O	ASN	A	80	-19.996	3.689	10.669	1.00	0.00	O
ATOM	1319	N	SER	A	81	-18.806	2.703	9.015	1.00	0.00	N
ATOM	1320	HN	SER	A	81	-18.665	2.701	8.025	1.00	0.00	
ATOM	1321	CA	SER	A	81	-17.805	2.012	9.866	1.00	0.00	C
ATOM	1322	HA	SER	A	81	-17.932	2.347	10.800	1.00	0.00	
ATOM	1323	CB	SER	A	81	-18.052	0.531	9.849	1.00	0.00	C
ATOM	1324	HB1	SER	A	81	-17.839	0.149	8.950	1.00	0.00	
ATOM	1325	HB2	SER	A	81	-17.499	0.074	10.545	1.00	0.00	
ATOM	1326	OG	SER	A	81	-19.418	0.275	10.128	1.00	0.00	O
ATOM	1327	HG1	SER	A	81	-19.581	-0.711	10.116	1.00	0.00	
ATOM	1328	C	SER	A	81	-16.375	2.323	9.400	1.00	0.00	C
ATOM	1329	O	SER	A	81	-16.149	2.740	8.261	1.00	0.00	O
ATOM	1330	N	VAL	A	82	-15.416	2.129	10.309	1.00	0.00	N
ATOM	1331	HN	VAL	A	82	-15.677	1.997	11.265	1.00	0.00	
ATOM	1332	CA	VAL	A	82	-14.016	2.103	9.960	1.00	0.00	C
ATOM	1333	HA	VAL	A	82	-13.981	2.277	8.976	1.00	0.00	
ATOM	1334	CB	VAL	A	82	-13.208	3.208	10.658	1.00	0.00	C
ATOM	1335	HB	VAL	A	82	-13.384	3.128	11.639	1.00	0.00	
ATOM	1336	CG1	VAL	A	82	-11.727	3.054	10.409	1.00	0.00	C
ATOM	1337	1HG1	VAL	A	82	-11.232	3.787	10.876	1.00	0.00	
ATOM	1338	2HG1	VAL	A	82	-11.420	2.169	10.760	1.00	0.00	
ATOM	1339	3HG1	VAL	A	82	-11.547	3.104	9.427	1.00	0.00	
ATOM	1340	CG2	VAL	A	82	-13.631	4.581	10.215	1.00	0.00	C
ATOM	1341	1HG2	VAL	A	82	-13.083	5.269	10.691	1.00	0.00	
ATOM	1342	2HG2	VAL	A	82	-13.494	4.670	9.228	1.00	0.00	
ATOM	1343	3HG2	VAL	A	82	-14.598	4.718	10.431	1.00	0.00	
ATOM	1344	C	VAL	A	82	-13.474	0.722	10.326	1.00	0.00	C

ATOM	1345	O	VAL	A	82	-13.777	0.185	11.369	1.00	0.00	O
ATOM	1346	N	PHE	A	83	-12.635	0.181	9.450	1.00	0.00	N
ATOM	1347	HN	PHE	A	83	-12.405	0.696	8.624	1.00	0.00	
ATOM	1348	CA	PHE	A	83	-12.049	-1.104	9.640	1.00	0.00	C
ATOM	1349	HA	PHE	A	83	-12.434	-1.465	10.489	1.00	0.00	
ATOM	1350	CB	PHE	A	83	-12.411	-2.025	8.473	1.00	0.00	C
ATOM	1351	HB1	PHE	A	83	-12.247	-1.534	7.618	1.00	0.00	
ATOM	1352	HB2	PHE	A	83	-11.823	-2.833	8.512	1.00	0.00	
ATOM	1353	CG	PHE	A	83	-13.834	-2.499	8.458	1.00	0.00	C
ATOM	1354	CD1	PHE	A	83	-14.211	-3.625	9.159	1.00	0.00	C
ATOM	1355	HD1	PHE	A	83	-13.531	-4.118	9.702	1.00	0.00	
ATOM	1356	CE1	PHE	A	83	-15.524	-4.086	9.123	1.00	0.00	C
ATOM	1357	HE1	PHE	A	83	-15.776	-4.915	9.623	1.00	0.00	
ATOM	1358	CZ	PHE	A	83	-16.476	-3.403	8.401	1.00	0.00	C
ATOM	1359	HZ	PHE	A	83	-17.420	-3.733	8.372	1.00	0.00	
ATOM	1360	CD2	PHE	A	83	-14.797	-1.819	7.740	1.00	0.00	C
ATOM	1361	HD2	PHE	A	83	-14.543	-0.998	7.228	1.00	0.00	
ATOM	1362	CE2	PHE	A	83	-16.117	-2.261	7.719	1.00	0.00	C
ATOM	1363	HE2	PHE	A	83	-16.806	-1.748	7.207	1.00	0.00	
ATOM	1364	C	PHE	A	83	-10.537	-0.939	9.752	1.00	0.00	C
ATOM	1365	O	PHE	A	83	-9.899	-0.327	8.875	1.00	0.00	O
ATOM	1366	N	LEU	A	84	-9.978	-1.505	10.821	1.00	0.00	N
ATOM	1367	HN	LEU	A	84	-10.574	-1.866	11.538	1.00	0.00	
ATOM	1368	CA	LEU	A	84	-8.539	-1.621	10.990	1.00	0.00	C
ATOM	1369	HA	LEU	A	84	-8.090	-1.036	10.315	1.00	0.00	
ATOM	1370	CB	LEU	A	84	-8.182	-1.158	12.401	1.00	0.00	C
ATOM	1371	HB1	LEU	A	84	-8.960	-1.356	12.997	1.00	0.00	
ATOM	1372	HB2	LEU	A	84	-7.387	-1.682	12.706	1.00	0.00	
ATOM	1373	CG	LEU	A	84	-7.849	0.321	12.545	1.00	0.00	C
ATOM	1374	HG	LEU	A	84	-6.934	0.468	12.168	1.00	0.00	
ATOM	1375	CD1	LEU	A	84	-8.791	1.190	11.750	1.00	0.00	C
ATOM	1376	1HD1	LEU	A	84	-8.540	2.151	11.870	1.00	0.00	
ATOM	1377	2HD1	LEU	A	84	-8.730	0.948	10.782	1.00	0.00	
ATOM	1378	3HD1	LEU	A	84	-9.727	1.047	12.071	1.00	0.00	
ATOM	1379	CD2	LEU	A	84	-7.847	0.727	14.008	1.00	0.00	C
ATOM	1380	1HD2	LEU	A	84	-7.627	1.700	14.084	1.00	0.00	
ATOM	1381	2HD2	LEU	A	84	-8.750	0.558	14.402	1.00	0.00	
ATOM	1382	3HD2	LEU	A	84	-7.162	0.191	14.502	1.00	0.00	
ATOM	1383	C	LEU	A	84	-8.135	-3.082	10.767	1.00	0.00	C
ATOM	1384	O	LEU	A	84	-8.675	-3.992	11.420	1.00	0.00	O
ATOM	1385	N	VAL	A	85	-7.230	-3.304	9.818	1.00	0.00	N
ATOM	1386	HN	VAL	A	85	-6.931	-2.546	9.238	1.00	0.00	
ATOM	1387	CA	VAL	A	85	-6.669	-4.622	9.609	1.00	0.00	C
ATOM	1388	HA	VAL	A	85	-7.308	-5.273	10.019	1.00	0.00	
ATOM	1389	CB	VAL	A	85	-6.536	-4.960	8.122	1.00	0.00	C
ATOM	1390	HB	VAL	A	85	-5.852	-4.342	7.734	1.00	0.00	
ATOM	1391	CG1	VAL	A	85	-6.038	-6.379	7.917	1.00	0.00	C
ATOM	1392	1HG1	VAL	A	85	-5.962	-6.568	6.938	1.00	0.00	
ATOM	1393	2HG1	VAL	A	85	-5.141	-6.483	8.347	1.00	0.00	
ATOM	1394	3HG1	VAL	A	85	-6.683	-7.022	8.331	1.00	0.00	
ATOM	1395	CG2	VAL	A	85	-7.831	-4.719	7.367	1.00	0.00	C
ATOM	1396	1HG2	VAL	A	85	-7.703	-4.951	6.403	1.00	0.00	
ATOM	1397	2HG2	VAL	A	85	-8.554	-5.291	7.754	1.00	0.00	
ATOM	1398	3HG2	VAL	A	85	-8.089	-3.756	7.447	1.00	0.00	
ATOM	1399	C	VAL	A	85	-5.313	-4.657	10.292	1.00	0.00	C
ATOM	1400	O	VAL	A	85	-4.415	-3.932	9.907	1.00	0.00	O
ATOM	1401	N	MET	A	86	-5.180	-5.525	11.291	1.00	0.00	N
ATOM	1402	HN	MET	A	86	-5.919	-6.175	11.469	1.00	0.00	
ATOM	1403	CA	MET	A	86	-4.009	-5.559	12.122	1.00	0.00	C
ATOM	1404	HA	MET	A	86	-3.349	-4.965	11.661	1.00	0.00	
ATOM	1405	CB	MET	A	86	-4.342	-5.042	13.520	1.00	0.00	C
ATOM	1406	HB1	MET	A	86	-4.964	-5.682	13.971	1.00	0.00	
ATOM	1407	HB2	MET	A	86	-3.500	-4.963	14.054	1.00	0.00	
ATOM	1408	CG	MET	A	86	-4.994	-3.711	13.486	1.00	0.00	C
ATOM	1409	HG1	MET	A	86	-4.349	-3.117	13.006	1.00	0.00	
ATOM	1410	HG2	MET	A	86	-5.812	-3.836	12.925	1.00	0.00	
ATOM	1411	SD	MET	A	86	-5.367	-3.136	15.131	1.00	0.00	S
ATOM	1412	CE	MET	A	86	-3.741	-2.554	15.603	1.00	0.00	C
ATOM	1413	HE1	MET	A	86	-3.775	-2.187	16.532	1.00	0.00	
ATOM	1414	HE2	MET	A	86	-3.092	-3.314	15.569	1.00	0.00	
ATOM	1415	HE3	MET	A	86	-3.448	-1.836	14.972	1.00	0.00	

```
ATOM   1416  C   MET A  86      -3.468  -6.976  12.227  1.00  0.00           C
ATOM   1417  O   MET A  86      -4.104  -7.911  11.885  1.00  0.00           O
ATOM   1418  N   GLU A  87      -2.249  -7.063  12.713  1.00  0.00           N
ATOM   1419  HN  GLU A  87      -1.744  -6.214  12.870  1.00  0.00
ATOM   1420  CA  GLU A  87      -1.597  -8.307  13.033  1.00  0.00           C
ATOM   1421  HA  GLU A  87      -1.489  -8.837  12.192  1.00  0.00
ATOM   1422  CB  GLU A  87      -0.236  -7.977  13.633  1.00  0.00           C
ATOM   1423  HB1 GLU A  87       0.299  -7.469  12.958  1.00  0.00
ATOM   1424  HB2 GLU A  87      -0.371  -7.410  14.446  1.00  0.00
ATOM   1425  CG  GLU A  87       0.558  -9.168  14.038  1.00  0.00           C
ATOM   1426  HG1 GLU A  87       0.069  -9.640  14.771  1.00  0.00
ATOM   1427  HG2 GLU A  87       0.642  -9.776  13.248  1.00  0.00
ATOM   1428  CD  GLU A  87       1.936  -8.803  14.524  1.00  0.00           C
ATOM   1429  OE1 GLU A  87       2.094  -7.692  15.088  1.00  0.00           O
ATOM   1430  OE2 GLU A  87       2.864  -9.625  14.291  1.00  0.00           O
ATOM   1431  C   GLU A  87      -2.468  -9.090  14.009  1.00  0.00           C
ATOM   1432  O   GLU A  87      -2.978  -8.524  14.980  1.00  0.00           O
ATOM   1433  N   TYR A  88      -2.680 -10.366  13.705  1.00  0.00           N
ATOM   1434  HN  TYR A  88      -2.351 -10.717  12.828  1.00  0.00
ATOM   1435  CA  TYR A  88      -3.381 -11.273  14.615  1.00  0.00           C
ATOM   1436  HA  TYR A  88      -4.041 -10.704  15.105  1.00  0.00
ATOM   1437  CB  TYR A  88      -4.125 -12.375  13.865  1.00  0.00           C
ATOM   1438  HB1 TYR A  88      -4.814 -11.951  13.278  1.00  0.00
ATOM   1439  HB2 TYR A  88      -3.468 -12.875  13.301  1.00  0.00
ATOM   1440  CG  TYR A  88      -4.823 -13.369  14.757  1.00  0.00           C
ATOM   1441  CD1 TYR A  88      -5.885 -12.962  15.547  1.00  0.00           C
ATOM   1442  HD1 TYR A  88      -6.190 -12.011  15.504  1.00  0.00
ATOM   1443  CE1 TYR A  88      -6.534 -13.842  16.395  1.00  0.00           C
ATOM   1444  HE1 TYR A  88      -7.291 -13.522  16.965  1.00  0.00
ATOM   1445  CZ  TYR A  88      -6.136 -15.162  16.453  1.00  0.00           C
ATOM   1446  OH  TYR A  88      -6.799 -15.979  17.320  1.00  0.00           O
ATOM   1447  HH  TYR A  88      -6.418 -16.902  17.266  1.00  0.00
ATOM   1448  CD2 TYR A  88      -4.431 -14.703  14.825  1.00  0.00           C
ATOM   1449  HD2 TYR A  88      -3.673 -15.022  14.256  1.00  0.00
ATOM   1450  CE2 TYR A  88      -5.073 -15.598  15.669  1.00  0.00           C
ATOM   1451  HE2 TYR A  88      -4.773 -16.551  15.713  1.00  0.00
ATOM   1452  C   TYR A  88      -2.365 -11.917  15.553  1.00  0.00           C
ATOM   1453  O   TYR A  88      -1.407 -12.515  15.092  1.00  0.00           O
ATOM   1454  N   CYS A  89      -2.590 -11.771  16.858  1.00  0.00           N
ATOM   1455  HN  CYS A  89      -3.361 -11.207  17.154  1.00  0.00
ATOM   1456  CA  CYS A  89      -1.763 -12.397  17.859  1.00  0.00           C
ATOM   1457  HA  CYS A  89      -0.921 -12.684  17.401  1.00  0.00
ATOM   1458  CB  CYS A  89      -1.416 -11.408  18.957  1.00  0.00           C
ATOM   1459  HB1 CYS A  89      -2.289 -11.205  19.400  1.00  0.00
ATOM   1460  HB2 CYS A  89      -0.834 -11.922  19.587  1.00  0.00
ATOM   1461  SG  CYS A  89      -0.603  -9.921  18.335  1.00  0.00           S
ATOM   1462  HG1 CYS A  89      -0.399  -9.309  19.099  1.00  0.00
ATOM   1463  C   CYS A  89      -2.522 -13.590  18.436  1.00  0.00           C
ATOM   1464  O   CYS A  89      -3.540 -13.405  19.122  1.00  0.00           O
ATOM   1465  N   ASN A  90      -2.032 -14.798  18.155  1.00  0.00           N
ATOM   1466  HN  ASN A  90      -1.112 -14.876  17.771  1.00  0.00
ATOM   1467  CA  ASN A  90      -2.819 -16.021  18.399  1.00  0.00           C
ATOM   1468  HA  ASN A  90      -3.760 -15.758  18.184  1.00  0.00
ATOM   1469  CB  ASN A  90      -2.383 -17.156  17.473  1.00  0.00           C
ATOM   1470  HB1 ASN A  90      -2.997 -17.934  17.606  1.00  0.00
ATOM   1471  HB2 ASN A  90      -2.444 -16.841  16.526  1.00  0.00
ATOM   1472  CG  ASN A  90      -0.958 -17.612  17.740  1.00  0.00           C
ATOM   1473  OD1 ASN A  90      -0.260 -17.042  18.579  1.00  0.00           O
ATOM   1474  ND2 ASN A  90      -0.501 -18.621  17.022  1.00  0.00           N
ATOM   1475 1HD2 ASN A  90      -1.087 -19.052  16.336  1.00  0.00
ATOM   1476 2HD2 ASN A  90       0.431 -18.954  17.164  1.00  0.00
ATOM   1477  C   ASN A  90      -2.723 -16.478  19.870  1.00  0.00           C
ATOM   1478  O   ASN A  90      -3.271 -17.498  20.208  1.00  0.00           O
ATOM   1479  N   GLY A  91      -2.058 -15.698  20.732  1.00  0.00           N
ATOM   1480  HN  GLY A  91      -1.696 -14.826  20.404  1.00  0.00
ATOM   1481  CA  GLY A  91      -1.833 -16.067  22.150  1.00  0.00           C
ATOM   1482  HA1 GLY A  91      -2.028 -17.044  22.240  1.00  0.00
ATOM   1483  HA2 GLY A  91      -0.868 -15.900  22.354  1.00  0.00
ATOM   1484  C   GLY A  91      -2.690 -15.298  23.154  1.00  0.00           C
ATOM   1485  O   GLY A  91      -2.618 -15.565  24.349  1.00  0.00           O
ATOM   1486  N   GLY A  92      -3.502 -14.350  22.684  1.00  0.00           N
```

```
ATOM   1487  HN  GLY A  92      -3.563 -14.210  21.696  1.00  0.00
ATOM   1488  CA  GLY A  92      -4.312 -13.502  23.576  1.00  0.00           C
ATOM   1489  HA1 GLY A  92      -4.990 -13.022  23.019  1.00  0.00
ATOM   1490  HA2 GLY A  92      -4.778 -14.096  24.232  1.00  0.00
ATOM   1491  C   GLY A  92      -3.446 -12.487  24.323  1.00  0.00           C
ATOM   1492  O   GLY A  92      -2.341 -12.132  23.871  1.00  0.00           O
ATOM   1493  N   ASP A  93      -3.947 -12.015  25.469  1.00  0.00           N
ATOM   1494  HN  ASP A  93      -4.802 -12.397  25.819  1.00  0.00
ATOM   1495  CA  ASP A  93      -3.282 -10.961  26.221  1.00  0.00           C
ATOM   1496  HA  ASP A  93      -2.646 -10.515  25.592  1.00  0.00
ATOM   1497  CB  ASP A  93      -4.275  -9.875  26.667  1.00  0.00           C
ATOM   1498  HB1 ASP A  93      -3.751  -9.131  27.082  1.00  0.00
ATOM   1499  HB2 ASP A  93      -4.746  -9.537  25.852  1.00  0.00
ATOM   1500  CG  ASP A  93      -5.342 -10.302  27.672  1.00  0.00           C
ATOM   1501  OD1 ASP A  93      -5.092 -11.254  28.430  1.00  0.00           O
ATOM   1502  OD2 ASP A  93      -6.403  -9.635  27.710  1.00  0.00           O
ATOM   1503  C   ASP A  93      -2.475 -11.590  27.368  1.00  0.00           C
ATOM   1504  O   ASP A  93      -2.715 -12.735  27.794  1.00  0.00           O
ATOM   1505  N   LEU A  94      -1.491 -10.833  27.839  1.00  0.00           N
ATOM   1506  HN  LEU A  94      -1.344  -9.932  27.430  1.00  0.00
ATOM   1507  CA  LEU A  94      -0.619 -11.253  28.920  1.00  0.00           C
ATOM   1508  HA  LEU A  94      -0.202 -12.119  28.644  1.00  0.00
ATOM   1509  CB  LEU A  94       0.456 -10.185  29.130  1.00  0.00           C
ATOM   1510  HB1 LEU A  94       1.008 -10.136  28.297  1.00  0.00
ATOM   1511  HB2 LEU A  94      -0.005  -9.310  29.277  1.00  0.00
ATOM   1512  CG  LEU A  94       1.403 -10.417  30.304  1.00  0.00           C
ATOM   1513  HG  LEU A  94       0.854 -10.438  31.139  1.00  0.00
ATOM   1514  CD1 LEU A  94       2.112 -11.747  30.184  1.00  0.00           C
ATOM   1515 1HD1 LEU A  94       2.724 -11.870  30.965  1.00  0.00
ATOM   1516 2HD1 LEU A  94       1.437 -12.485  30.170  1.00  0.00
ATOM   1517 3HD1 LEU A  94       2.644 -11.766  29.338  1.00  0.00
ATOM   1518  CD2 LEU A  94       2.430  -9.289  30.398  1.00  0.00           C
ATOM   1519 1HD2 LEU A  94       3.041  -9.458  31.172  1.00  0.00
ATOM   1520 2HD2 LEU A  94       2.964  -9.253  29.553  1.00  0.00
ATOM   1521 3HD2 LEU A  94       1.957  -8.418  30.531  1.00  0.00
ATOM   1522  C   LEU A  94      -1.434 -11.471  30.196  1.00  0.00           C
ATOM   1523  O   LEU A  94      -1.082 -12.323  30.999  1.00  0.00           O
ATOM   1524  N   ALA A  95      -2.521 -10.700  30.355  1.00  0.00           N
ATOM   1525  HN  ALA A  95      -2.740 -10.034  29.642  1.00  0.00
ATOM   1526  CA  ALA A  95      -3.410 -10.788  31.534  1.00  0.00           C
ATOM   1527  HA  ALA A  95      -2.871 -10.519  32.332  1.00  0.00
ATOM   1528  CB  ALA A  95      -4.559  -9.821  31.408  1.00  0.00           C
ATOM   1529  HB1 ALA A  95      -5.147  -9.896  32.213  1.00  0.00
ATOM   1530  HB2 ALA A  95      -4.204  -8.889  31.340  1.00  0.00
ATOM   1531  HB3 ALA A  95      -5.088 -10.037  30.587  1.00  0.00
ATOM   1532  C   ALA A  95      -3.920 -12.227  31.699  1.00  0.00           C
ATOM   1533  O   ALA A  95      -3.856 -12.784  32.788  1.00  0.00           O
ATOM   1534  N   ASP A  96      -4.399 -12.808  30.592  1.00  0.00           N
ATOM   1535  HN  ASP A  96      -4.365 -12.289  29.738  1.00  0.00
ATOM   1536  CA  ASP A  96      -4.966 -14.147  30.555  1.00  0.00           C
ATOM   1537  HA  ASP A  96      -5.564 -14.222  31.353  1.00  0.00
ATOM   1538  CB  ASP A  96      -5.834 -14.340  29.310  1.00  0.00           C
ATOM   1539  HB1 ASP A  96      -5.313 -14.040  28.511  1.00  0.00
ATOM   1540  HB2 ASP A  96      -6.050 -15.312  29.221  1.00  0.00
ATOM   1541  CG  ASP A  96      -7.148 -13.548  29.366  1.00  0.00           C
ATOM   1542  OD1 ASP A  96      -7.778 -13.515  30.435  1.00  0.00           O
ATOM   1543  OD2 ASP A  96      -7.529 -12.951  28.352  1.00  0.00           O
ATOM   1544  C   ASP A  96      -3.846 -15.190  30.670  1.00  0.00           C
ATOM   1545  O   ASP A  96      -4.049 -16.248  31.245  1.00  0.00           O
ATOM   1546  N   TYR A  97      -2.665 -14.881  30.133  1.00  0.00           N
ATOM   1547  HN  TYR A  97      -2.568 -14.018  29.637  1.00  0.00
ATOM   1548  CA  TYR A  97      -1.508 -15.766  30.247  1.00  0.00           C
ATOM   1549  HA  TYR A  97      -1.796 -16.647  29.872  1.00  0.00
ATOM   1550  CB  TYR A  97      -0.333 -15.223  29.436  1.00  0.00           C
ATOM   1551  HB1 TYR A  97      -0.651 -15.033  28.507  1.00  0.00
ATOM   1552  HB2 TYR A  97      -0.021 -14.374  29.862  1.00  0.00
ATOM   1553  CG  TYR A  97       0.850 -16.149  29.336  1.00  0.00           C
ATOM   1554  CD1 TYR A  97       0.803 -17.277  28.535  1.00  0.00           C
ATOM   1555  HD1 TYR A  97      -0.033 -17.480  28.025  1.00  0.00
ATOM   1556  CE1 TYR A  97       1.888 -18.131  28.424  1.00  0.00           C
ATOM   1557  HE1 TYR A  97       1.829 -18.946  27.847  1.00  0.00
```

```
ATOM   1558  CZ  TYR A  97       3.059 -17.851  29.118  1.00  0.00           C
ATOM   1559  OH  TYR A  97       4.134 -18.690  28.989  1.00  0.00           O
ATOM   1560  HH  TYR A  97       4.891 -18.351  29.548  1.00  0.00
ATOM   1561  CD2 TYR A  97       2.027 -15.880  30.017  1.00  0.00           C
ATOM   1562  HD2 TYR A  97       2.082 -15.066  30.596  1.00  0.00
ATOM   1563  CE2 TYR A  97       3.129 -16.713  29.914  1.00  0.00           C
ATOM   1564  HE2 TYR A  97       3.970 -16.497  30.409  1.00  0.00
ATOM   1565  C   TYR A  97      -1.109 -15.916  31.715  1.00  0.00           C
ATOM   1566  O   TYR A  97      -0.782 -17.013  32.149  1.00  0.00           O
ATOM   1567  N   LEU A  98      -1.119 -14.795  32.459  1.00  0.00           N
ATOM   1568  HN  LEU A  98      -1.401 -13.937  32.029  1.00  0.00
ATOM   1569  CA  LEU A  98      -0.729 -14.778  33.893  1.00  0.00           C
ATOM   1570  HA  LEU A  98       0.147 -15.254  33.975  1.00  0.00
ATOM   1571  CB  LEU A  98      -0.570 -13.331  34.369  1.00  0.00           C
ATOM   1572  HB1 LEU A  98      -1.387 -12.825  34.092  1.00  0.00
ATOM   1573  HB2 LEU A  98      -0.506 -13.341  35.367  1.00  0.00
ATOM   1574  CG  LEU A  98       0.651 -12.585  33.827  1.00  0.00           C
ATOM   1575  HG  LEU A  98       0.530 -12.497  32.838  1.00  0.00
ATOM   1576  CD1 LEU A  98       0.763 -11.200  34.428  1.00  0.00           C
ATOM   1577 1HD1 LEU A  98       1.568 -10.739  34.055  1.00  0.00
ATOM   1578 2HD1 LEU A  98      -0.057 -10.672  34.204  1.00  0.00
ATOM   1579 3HD1 LEU A  98       0.851 -11.274  35.421  1.00  0.00
ATOM   1580  CD2 LEU A  98       1.931 -13.348  34.077  1.00  0.00           C
ATOM   1581 1HD2 LEU A  98       2.704 -12.831  33.710  1.00  0.00
ATOM   1582 2HD2 LEU A  98       2.054 -13.479  35.061  1.00  0.00
ATOM   1583 3HD2 LEU A  98       1.882 -14.239  33.626  1.00  0.00
ATOM   1584  C   LEU A  98      -1.784 -15.509  34.731  1.00  0.00           C
ATOM   1585  O   LEU A  98      -1.445 -16.170  35.697  1.00  0.00           O
ATOM   1586  N   GLN A  99      -3.058 -15.329  34.356  1.00  0.00           N
ATOM   1587  HN  GLN A  99      -3.244 -14.673  33.624  1.00  0.00
ATOM   1588  CA  GLN A  99      -4.187 -16.029  34.948  1.00  0.00           C
ATOM   1589  HA  GLN A  99      -4.221 -15.755  35.909  1.00  0.00
ATOM   1590  CB  GLN A  99      -5.480 -15.603  34.244  1.00  0.00           C
ATOM   1591  HB1 GLN A  99      -5.523 -14.604  34.233  1.00  0.00
ATOM   1592  HB2 GLN A  99      -5.460 -15.947  33.305  1.00  0.00
ATOM   1593  CG  GLN A  99      -6.740 -16.126  34.916  1.00  0.00           C
ATOM   1594  HG1 GLN A  99      -7.513 -16.004  34.294  1.00  0.00
ATOM   1595  HG2 GLN A  99      -6.622 -17.099  35.114  1.00  0.00
ATOM   1596  CD  GLN A  99      -7.039 -15.400  36.211  1.00  0.00           C
ATOM   1597  OE1 GLN A  99      -6.673 -14.233  36.376  1.00  0.00           O
ATOM   1598  NE2 GLN A  99      -7.680 -16.094  37.150  1.00  0.00           N
ATOM   1599 1HE2 GLN A  99      -7.938 -17.045  36.980  1.00  0.00
ATOM   1600 2HE2 GLN A  99      -7.903 -15.664  38.025  1.00  0.00
ATOM   1601  C   GLN A  99      -3.974 -17.551  34.853  1.00  0.00           C
ATOM   1602  O   GLN A  99      -4.300 -18.291  35.765  1.00  0.00           O
ATOM   1603  N   ALA A 100      -3.406 -17.993  33.734  1.00  0.00           N
ATOM   1604  HN  ALA A 100      -3.081 -17.320  33.069  1.00  0.00
ATOM   1605  CA  ALA A 100      -3.232 -19.414  33.425  1.00  0.00           C
ATOM   1606  HA  ALA A 100      -4.058 -19.867  33.760  1.00  0.00
ATOM   1607  CB  ALA A 100      -3.128 -19.615  31.930  1.00  0.00           C
ATOM   1608  HB1 ALA A 100      -3.010 -20.588  31.732  1.00  0.00
ATOM   1609  HB2 ALA A 100      -3.963 -19.285  31.490  1.00  0.00
ATOM   1610  HB3 ALA A 100      -2.342 -19.105  31.579  1.00  0.00
ATOM   1611  C   ALA A 100      -1.996 -19.994  34.138  1.00  0.00           C
ATOM   1612  O   ALA A 100      -2.019 -21.155  34.526  1.00  0.00           O
ATOM   1613  N   LYS A 101      -0.922 -19.199  34.260  1.00  0.00           N
ATOM   1614  HN  LYS A 101      -1.024 -18.226  34.053  1.00  0.00
ATOM   1615  CA  LYS A 101       0.402 -19.693  34.685  1.00  0.00           C
ATOM   1616  HA  LYS A 101       0.342 -20.689  34.622  1.00  0.00
ATOM   1617  CB  LYS A 101       1.489 -19.151  33.751  1.00  0.00           C
ATOM   1618  HB1 LYS A 101       1.456 -18.152  33.784  1.00  0.00
ATOM   1619  HB2 LYS A 101       2.375 -19.467  34.091  1.00  0.00
ATOM   1620  CG  LYS A 101       1.377 -19.569  32.293  1.00  0.00           C
ATOM   1621  HG1 LYS A 101       0.424 -19.476  32.004  1.00  0.00
ATOM   1622  HG2 LYS A 101       1.953 -18.967  31.741  1.00  0.00
ATOM   1623  CD  LYS A 101       1.808 -20.990  32.049  1.00  0.00           C
ATOM   1624  HD1 LYS A 101       2.490 -21.235  32.738  1.00  0.00
ATOM   1625  HD2 LYS A 101       1.008 -21.582  32.146  1.00  0.00
ATOM   1626  CE  LYS A 101       2.408 -21.210  30.676  1.00  0.00           C
ATOM   1627  HE1 LYS A 101       2.023 -20.547  30.034  1.00  0.00
ATOM   1628  HE2 LYS A 101       3.400 -21.091  30.723  1.00  0.00
```

```
ATOM   1629  NZ  LYS A 101       2.121 -22.573  30.173  1.00  0.00           N
ATOM   1630  HZ1 LYS A 101       2.529 -22.687  29.267  1.00  0.00
ATOM   1631  HZ2 LYS A 101       2.508 -23.248  30.801  1.00  0.00
ATOM   1632  HZ3 LYS A 101       1.132 -22.704  30.112  1.00  0.00
ATOM   1633  C   LYS A 101       0.727 -19.275  36.136  1.00  0.00           C
ATOM   1634  O   LYS A 101       1.587 -19.885  36.768  1.00  0.00           O
ATOM   1635  N   GLY A 102       0.076 -18.213  36.632  1.00  0.00           N
ATOM   1636  HN  GLY A 102      -0.575 -17.740  36.038  1.00  0.00
ATOM   1637  CA  GLY A 102       0.263 -17.708  37.989  1.00  0.00           C
ATOM   1638  HA1 GLY A 102      -0.591 -17.276  38.278  1.00  0.00
ATOM   1639  HA2 GLY A 102       0.460 -18.487  38.584  1.00  0.00
ATOM   1640  C   GLY A 102       1.396 -16.703  38.073  1.00  0.00           C
ATOM   1641  O   GLY A 102       1.162 -15.496  38.030  1.00  0.00           O
ATOM   1642  N   THR A 103       2.621 -17.212  38.219  1.00  0.00           N
ATOM   1643  HN  THR A 103       2.709 -18.203  38.319  1.00  0.00
ATOM   1644  CA  THR A 103       3.831 -16.416  38.241  1.00  0.00           C
ATOM   1645  HA  THR A 103       3.548 -15.482  38.021  1.00  0.00
ATOM   1646  CB  THR A 103       4.496 -16.409  39.627  1.00  0.00           C
ATOM   1647  HB  THR A 103       5.398 -15.981  39.567  1.00  0.00
ATOM   1648  OG1 THR A 103       4.671 -17.758  40.053  1.00  0.00           O
ATOM   1649  HG1 THR A 103       5.103 -17.769  40.955  1.00  0.00
ATOM   1650  CG2 THR A 103       3.704 -15.662  40.673  1.00  0.00           C
ATOM   1651 1HG2 THR A 103       4.190 -15.696  41.546  1.00  0.00
ATOM   1652 2HG2 THR A 103       3.593 -14.709  40.390  1.00  0.00
ATOM   1653 3HG2 THR A 103       2.804 -16.086  40.776  1.00  0.00
ATOM   1654  C   THR A 103       4.786 -16.980  37.196  1.00  0.00           C
ATOM   1655  O   THR A 103       4.608 -18.104  36.754  1.00  0.00           O
ATOM   1656  N   LEU A 104       5.811 -16.197  36.841  1.00  0.00           N
ATOM   1657  HN  LEU A 104       5.921 -15.319  37.306  1.00  0.00
ATOM   1658  CA  LEU A 104       6.774 -16.559  35.812  1.00  0.00           C
ATOM   1659  HA  LEU A 104       6.550 -17.485  35.508  1.00  0.00
ATOM   1660  CB  LEU A 104       6.654 -15.568  34.652  1.00  0.00           C
ATOM   1661  HB1 LEU A 104       6.994 -14.682  34.968  1.00  0.00
ATOM   1662  HB2 LEU A 104       7.230 -15.899  33.904  1.00  0.00
ATOM   1663  CG  LEU A 104       5.246 -15.358  34.100  1.00  0.00           C
ATOM   1664  HG  LEU A 104       4.672 -15.003  34.838  1.00  0.00
ATOM   1665  CD1 LEU A 104       5.269 -14.341  32.967  1.00  0.00           C
ATOM   1666 1HD1 LEU A 104       4.342 -14.213  32.615  1.00  0.00
ATOM   1667 2HD1 LEU A 104       5.619 -13.469  33.309  1.00  0.00
ATOM   1668 3HD1 LEU A 104       5.861 -14.673  32.233  1.00  0.00
ATOM   1669  CD2 LEU A 104       4.632 -16.670  33.632  1.00  0.00           C
ATOM   1670 1HD2 LEU A 104       3.713 -16.500  33.277  1.00  0.00
ATOM   1671 2HD2 LEU A 104       5.200 -17.065  32.910  1.00  0.00
ATOM   1672 3HD2 LEU A 104       4.580 -17.307  34.401  1.00  0.00
ATOM   1673  C   LEU A 104       8.190 -16.534  36.395  1.00  0.00           C
ATOM   1674  O   LEU A 104       8.519 -15.669  37.193  1.00  0.00           O
ATOM   1675  N   SER A 105       9.021 -17.481  35.948  1.00  0.00           N
ATOM   1676  HN  SER A 105       8.646 -18.217  35.384  1.00  0.00
ATOM   1677  CA  SER A 105      10.443 -17.491  36.244  1.00  0.00           C
ATOM   1678  HA  SER A 105      10.521 -17.592  37.236  1.00  0.00
ATOM   1679  CB  SER A 105      11.120 -18.666  35.598  1.00  0.00           C
ATOM   1680  HB1 SER A 105      12.086 -18.692  35.855  1.00  0.00
ATOM   1681  HB2 SER A 105      10.677 -19.517  35.879  1.00  0.00
ATOM   1682  OG  SER A 105      11.040 -18.571  34.174  1.00  0.00           O
ATOM   1683  HG1 SER A 105      11.495 -19.360  33.761  1.00  0.00
ATOM   1684  C   SER A 105      11.077 -16.181  35.767  1.00  0.00           C
ATOM   1685  O   SER A 105      10.603 -15.551  34.828  1.00  0.00           O
ATOM   1686  N   GLU A 106      12.184 -15.811  36.406  1.00  0.00           N
ATOM   1687  HN  GLU A 106      12.484 -16.349  37.194  1.00  0.00
ATOM   1688  CA  GLU A 106      12.969 -14.671  36.014  1.00  0.00           C
ATOM   1689  HA  GLU A 106      12.357 -13.881  36.049  1.00  0.00
ATOM   1690  CB  GLU A 106      14.112 -14.440  37.000  1.00  0.00           C
ATOM   1691  HB1 GLU A 106      14.671 -15.268  37.039  1.00  0.00
ATOM   1692  HB2 GLU A 106      14.668 -13.678  36.669  1.00  0.00
ATOM   1693  CG  GLU A 106      13.646 -14.114  38.407  1.00  0.00           C
ATOM   1694  HG1 GLU A 106      12.900 -13.451  38.340  1.00  0.00
ATOM   1695  HG2 GLU A 106      13.306 -14.957  38.823  1.00  0.00
ATOM   1696  CD  GLU A 106      14.713 -13.533  39.322  1.00  0.00           C
ATOM   1697  OE1 GLU A 106      15.688 -12.916  38.794  1.00  0.00           O
ATOM   1698  OE2 GLU A 106      14.561 -13.676  40.564  1.00  0.00           O
ATOM   1699  C   GLU A 106      13.477 -14.879  34.586  1.00  0.00           C
```

```
ATOM   1700  O   GLU A 106      13.559 -13.924  33.835  1.00  0.00           O
ATOM   1701  N   ASP A 107      13.775 -16.131  34.208  1.00  0.00           N
ATOM   1702  HN  ASP A 107      13.705 -16.869  34.879  1.00  0.00
ATOM   1703  CA  ASP A 107      14.205 -16.456  32.828  1.00  0.00           C
ATOM   1704  HA  ASP A 107      15.055 -15.954  32.668  1.00  0.00
ATOM   1705  CB  ASP A 107      14.525 -17.946  32.652  1.00  0.00           C
ATOM   1706  HB1 ASP A 107      13.816 -18.478  33.115  1.00  0.00
ATOM   1707  HB2 ASP A 107      14.516 -18.157  31.675  1.00  0.00
ATOM   1708  CG  ASP A 107      15.875 -18.371  33.214  1.00  0.00           C
ATOM   1709  OD1 ASP A 107      16.701 -17.483  33.523  1.00  0.00           O
ATOM   1710  OD2 ASP A 107      16.112 -19.596  33.309  1.00  0.00           O
ATOM   1711  C   ASP A 107      13.121 -15.997  31.849  1.00  0.00           C
ATOM   1712  O   ASP A 107      13.408 -15.274  30.921  1.00  0.00           O
ATOM   1713  N   THR A 108      11.876 -16.411  32.104  1.00  0.00           N
ATOM   1714  HN  THR A 108      11.719 -16.982  32.910  1.00  0.00
ATOM   1715  CA  THR A 108      10.732 -16.068  31.259  1.00  0.00           C
ATOM   1716  HA  THR A 108      10.965 -16.422  30.353  1.00  0.00
ATOM   1717  CB  THR A 108       9.447 -16.744  31.757  1.00  0.00           C
ATOM   1718  HB  THR A 108       9.251 -16.464  32.697  1.00  0.00
ATOM   1719  OG1 THR A 108       9.645 -18.159  31.768  1.00  0.00           O
ATOM   1720  HG1 THR A 108       8.813 -18.610  32.091  1.00  0.00
ATOM   1721  CG2 THR A 108       8.241 -16.401  30.913  1.00  0.00           C
ATOM   1722 1HG2 THR A 108       7.435 -16.865  31.279  1.00  0.00
ATOM   1723 2HG2 THR A 108       8.093 -15.412  30.930  1.00  0.00
ATOM   1724 3HG2 THR A 108       8.398 -16.698  29.971  1.00  0.00
ATOM   1725  C   THR A 108      10.538 -14.540  31.217  1.00  0.00           C
ATOM   1726  O   THR A 108      10.254 -13.982  30.161  1.00  0.00           O
ATOM   1727  N   ILE A 109      10.653 -13.882  32.385  1.00  0.00           N
ATOM   1728  HN  ILE A 109      10.931 -14.393  33.198  1.00  0.00
ATOM   1729  CA  ILE A 109      10.389 -12.452  32.513  1.00  0.00           C
ATOM   1730  HA  ILE A 109       9.483 -12.284  32.125  1.00  0.00
ATOM   1731  CB  ILE A 109      10.336 -12.028  33.995  1.00  0.00           C
ATOM   1732  HB  ILE A 109      11.106 -12.460  34.465  1.00  0.00
ATOM   1733  CG2 ILE A 109      10.507 -10.520  34.159  1.00  0.00           C
ATOM   1734 1HG2 ILE A 109      10.467 -10.284  35.130  1.00  0.00
ATOM   1735 2HG2 ILE A 109      11.391 -10.242  33.784  1.00  0.00
ATOM   1736 3HG2 ILE A 109       9.774 -10.046  33.670  1.00  0.00
ATOM   1737  CG1 ILE A 109       9.040 -12.511  34.658  1.00  0.00           C
ATOM   1738 1HG1 ILE A 109       8.285 -11.941  34.334  1.00  0.00
ATOM   1739 2HG1 ILE A 109       8.880 -13.461  34.390  1.00  0.00
ATOM   1740  CD  ILE A 109       9.068 -12.444  36.178  1.00  0.00           C
ATOM   1741  HD1 ILE A 109       8.196 -12.772  36.542  1.00  0.00
ATOM   1742  HD2 ILE A 109       9.811 -13.018  36.523  1.00  0.00
ATOM   1743  HD3 ILE A 109       9.216 -11.498  36.467  1.00  0.00
ATOM   1744  C   ILE A 109      11.431 -11.679  31.696  1.00  0.00           C
ATOM   1745  O   ILE A 109      11.102 -10.675  31.072  1.00  0.00           O
ATOM   1746  N   ARG A 110      12.673 -12.170  31.683  1.00  0.00           N
ATOM   1747  HN  ARG A 110      12.858 -13.005  32.202  1.00  0.00
ATOM   1748  CA  ARG A 110      13.787 -11.545  30.943  1.00  0.00           C
ATOM   1749  HA  ARG A 110      13.860 -10.603  31.270  1.00  0.00
ATOM   1750  CB  ARG A 110      15.101 -12.279  31.232  1.00  0.00           C
ATOM   1751  HB1 ARG A 110      15.194 -12.382  32.222  1.00  0.00
ATOM   1752  HB2 ARG A 110      15.059 -13.182  30.805  1.00  0.00
ATOM   1753  CG  ARG A 110      16.342 -11.567  30.712  1.00  0.00           C
ATOM   1754  HG1 ARG A 110      16.238 -11.412  29.730  1.00  0.00
ATOM   1755  HG2 ARG A 110      16.431 -10.688  31.181  1.00  0.00
ATOM   1756  CD  ARG A 110      17.605 -12.384  30.951  1.00  0.00           C
ATOM   1757  HD1 ARG A 110      17.453 -13.327  30.655  1.00  0.00
ATOM   1758  HD2 ARG A 110      18.362 -11.989  30.430  1.00  0.00
ATOM   1759  NE  ARG A 110      18.003 -12.418  32.358  1.00  0.00           N
ATOM   1760  HE  ARG A 110      18.434 -11.598  32.734  1.00  0.00
ATOM   1761  CZ  ARG A 110      17.836 -13.459  33.182  1.00  0.00           C
ATOM   1762  NH1 ARG A 110      17.267 -14.573  32.751  1.00  0.00           N
ATOM   1763 1HH1 ARG A 110      16.958 -14.642  31.803  1.00  0.00
ATOM   1764 2HH1 ARG A 110      17.146 -15.346  33.374  1.00  0.00
ATOM   1765  NH2 ARG A 110      18.209 -13.362  34.447  1.00  0.00           N
ATOM   1766 1HH2 ARG A 110      18.614 -12.513  34.785  1.00  0.00
ATOM   1767 2HH2 ARG A 110      18.086 -14.138  35.066  1.00  0.00
ATOM   1768  C   ARG A 110      13.481 -11.555  29.439  1.00  0.00           C
ATOM   1769  O   ARG A 110      13.745 -10.580  28.731  1.00  0.00           O
ATOM   1770  N   VAL A 111      12.918 -12.669  28.963  1.00  0.00           N
```

```
ATOM   1771  HN  VAL A 111      12.718 -13.410  29.603  1.00  0.00
ATOM   1772  CA  VAL A 111      12.582 -12.858  27.563  1.00  0.00           C
ATOM   1773  HA  VAL A 111      13.417 -12.673  27.045  1.00  0.00
ATOM   1774  CB  VAL A 111      12.151 -14.313  27.293  1.00  0.00           C
ATOM   1775  HB  VAL A 111      11.512 -14.576  28.015  1.00  0.00
ATOM   1776  CG1 VAL A 111      11.403 -14.462  25.976  1.00  0.00           C
ATOM   1777 1HG1 VAL A 111      11.145 -15.419  25.846  1.00  0.00
ATOM   1778 2HG1 VAL A 111      10.580 -13.895  25.994  1.00  0.00
ATOM   1779 3HG1 VAL A 111      11.993 -14.170  25.223  1.00  0.00
ATOM   1780  CG2 VAL A 111      13.338 -15.261  27.357  1.00  0.00           C
ATOM   1781 1HG2 VAL A 111      13.029 -16.195  27.178  1.00  0.00
ATOM   1782 2HG2 VAL A 111      14.014 -14.996  26.669  1.00  0.00
ATOM   1783 3HG2 VAL A 111      13.752 -15.215  28.266  1.00  0.00
ATOM   1784  C   VAL A 111      11.488 -11.856  27.170  1.00  0.00           C
ATOM   1785  O   VAL A 111      11.602 -11.201  26.145  1.00  0.00           O
ATOM   1786  N   PHE A 112      10.435 -11.766  27.990  1.00  0.00           N
ATOM   1787  HN  PHE A 112      10.413 -12.351  28.801  1.00  0.00
ATOM   1788  CA  PHE A 112       9.319 -10.860  27.761  1.00  0.00           C
ATOM   1789  HA  PHE A 112       8.970 -11.074  26.849  1.00  0.00
ATOM   1790  CB  PHE A 112       8.209 -11.104  28.787  1.00  0.00           C
ATOM   1791  HB1 PHE A 112       8.628 -11.203  29.690  1.00  0.00
ATOM   1792  HB2 PHE A 112       7.599 -10.312  28.788  1.00  0.00
ATOM   1793  CG  PHE A 112       7.369 -12.328  28.536  1.00  0.00           C
ATOM   1794  CD1 PHE A 112       7.756 -13.297  27.615  1.00  0.00           C
ATOM   1795  HD1 PHE A 112       8.609 -13.180  27.107  1.00  0.00
ATOM   1796  CE1 PHE A 112       6.977 -14.422  27.391  1.00  0.00           C
ATOM   1797  HE1 PHE A 112       7.262 -15.100  26.714  1.00  0.00
ATOM   1798  CZ  PHE A 112       5.812 -14.606  28.100  1.00  0.00           C
ATOM   1799  HZ  PHE A 112       5.252 -15.420  27.944  1.00  0.00
ATOM   1800  CD2 PHE A 112       6.201 -12.538  29.248  1.00  0.00           C
ATOM   1801  HD2 PHE A 112       5.914 -11.869  29.933  1.00  0.00
ATOM   1802  CE2 PHE A 112       5.421 -13.666  29.024  1.00  0.00           C
ATOM   1803  HE2 PHE A 112       4.572 -13.795  29.537  1.00  0.00
ATOM   1804  C   PHE A 112       9.809  -9.406  27.803  1.00  0.00           C
ATOM   1805  O   PHE A 112       9.396  -8.567  26.999  1.00  0.00           O
ATOM   1806  N   LEU A 113      10.691  -9.122  28.754  1.00  0.00           N
ATOM   1807  HN  LEU A 113      11.090  -9.870  29.284  1.00  0.00
ATOM   1808  CA  LEU A 113      11.090  -7.767  29.045  1.00  0.00           C
ATOM   1809  HA  LEU A 113      10.269  -7.197  29.010  1.00  0.00
ATOM   1810  CB  LEU A 113      11.701  -7.741  30.442  1.00  0.00           C
ATOM   1811  HB1 LEU A 113      11.052  -8.167  31.072  1.00  0.00
ATOM   1812  HB2 LEU A 113      12.545  -8.277  30.421  1.00  0.00
ATOM   1813  CG  LEU A 113      12.039  -6.367  30.985  1.00  0.00           C
ATOM   1814  HG  LEU A 113      12.664  -5.910  30.353  1.00  0.00
ATOM   1815  CD1 LEU A 113      10.782  -5.514  31.097  1.00  0.00           C
ATOM   1816 1HD1 LEU A 113      11.022  -4.612  31.456  1.00  0.00
ATOM   1817 2HD1 LEU A 113      10.366  -5.413  30.193  1.00  0.00
ATOM   1818 3HD1 LEU A 113      10.133  -5.957  31.715  1.00  0.00
ATOM   1819  CD2 LEU A 113      12.729  -6.508  32.331  1.00  0.00           C
ATOM   1820 1HD2 LEU A 113      12.951  -5.601  32.688  1.00  0.00
ATOM   1821 2HD2 LEU A 113      12.120  -6.978  32.970  1.00  0.00
ATOM   1822 3HD2 LEU A 113      13.570  -7.038  32.221  1.00  0.00
ATOM   1823  C   LEU A 113      12.085  -7.277  27.991  1.00  0.00           C
ATOM   1824  O   LEU A 113      12.113  -6.108  27.653  1.00  0.00           O
ATOM   1825  N   HIS A 114      12.912  -8.188  27.488  1.00  0.00           N
ATOM   1826  HN  HIS A 114      12.915  -9.111  27.873  1.00  0.00
ATOM   1827  CA  HIS A 114      13.806  -7.875  26.398  1.00  0.00           C
ATOM   1828  HA  HIS A 114      14.413  -7.150  26.723  1.00  0.00
ATOM   1829  CB  HIS A 114      14.672  -9.089  26.048  1.00  0.00           C
ATOM   1830  HB1 HIS A 114      15.152  -9.396  26.870  1.00  0.00
ATOM   1831  HB2 HIS A 114      14.084  -9.824  25.711  1.00  0.00
ATOM   1832  ND1 HIS A 114      15.491  -9.128  23.664  1.00  0.00           N
ATOM   1833  CG  HIS A 114      15.693  -8.805  25.001  1.00  0.00           C
ATOM   1834  CE1 HIS A 114      16.552  -8.768  22.965  1.00  0.00           C
ATOM   1835  HE1 HIS A 114      16.684  -8.908  21.984  1.00  0.00
ATOM   1836  NE2 HIS A 114      17.425  -8.180  23.805  1.00  0.00           N
ATOM   1837  HE2 HIS A 114      18.313  -7.798  23.548  1.00  0.00
ATOM   1838  CD2 HIS A 114      16.896  -8.191  25.070  1.00  0.00           C
ATOM   1839  HD2 HIS A 114      17.322  -7.814  25.893  1.00  0.00
ATOM   1840  C   HIS A 114      12.984  -7.364  25.202  1.00  0.00           C
ATOM   1841  O   HIS A 114      13.364  -6.408  24.531  1.00  0.00           O
```

```
ATOM   1842  N   GLN A 115      11.836  -7.994  24.951  1.00  0.00           N
ATOM   1843  HN  GLN A 115      11.541  -8.727  25.564  1.00  0.00
ATOM   1844  CA  GLN A 115      11.003  -7.648  23.816  1.00  0.00           C
ATOM   1845  HA  GLN A 115      11.612  -7.531  23.031  1.00  0.00
ATOM   1846  CB  GLN A 115      10.045  -8.794  23.511  1.00  0.00           C
ATOM   1847  HB1 GLN A 115       9.530  -9.009  24.341  1.00  0.00
ATOM   1848  HB2 GLN A 115       9.414  -8.500  22.794  1.00  0.00
ATOM   1849  CG  GLN A 115      10.760 -10.056  23.042  1.00  0.00           C
ATOM   1850  HG1 GLN A 115      11.267  -9.855  22.204  1.00  0.00
ATOM   1851  HG2 GLN A 115      11.396 -10.357  23.752  1.00  0.00
ATOM   1852  CD  GLN A 115       9.788 -11.170  22.761  1.00  0.00           C
ATOM   1853  OE1 GLN A 115       9.003 -11.102  21.842  1.00  0.00           O
ATOM   1854  NE2 GLN A 115       9.819 -12.200  23.568  1.00  0.00           N
ATOM   1855 1HE2 GLN A 115      10.468 -12.220  24.328  1.00  0.00
ATOM   1856 2HE2 GLN A 115       9.193 -12.967  23.424  1.00  0.00
ATOM   1857  C   GLN A 115      10.276  -6.320  24.084  1.00  0.00           C
ATOM   1858  O   GLN A 115      10.226  -5.461  23.227  1.00  0.00           O
ATOM   1859  N   ILE A 116       9.726  -6.172  25.287  1.00  0.00           N
ATOM   1860  HN  ILE A 116       9.773  -6.934  25.933  1.00  0.00
ATOM   1861  CA  ILE A 116       9.059  -4.947  25.700  1.00  0.00           C
ATOM   1862  HA  ILE A 116       8.276  -4.833  25.089  1.00  0.00
ATOM   1863  CB  ILE A 116       8.541  -5.062  27.143  1.00  0.00           C
ATOM   1864  HB  ILE A 116       9.269  -5.435  27.718  1.00  0.00
ATOM   1865  CG2 ILE A 116       8.179  -3.692  27.679  1.00  0.00           C
ATOM   1866 1HG2 ILE A 116       7.844  -3.779  28.617  1.00  0.00
ATOM   1867 2HG2 ILE A 116       8.988  -3.105  27.667  1.00  0.00
ATOM   1868 3HG2 ILE A 116       7.466  -3.288  27.106  1.00  0.00
ATOM   1869  CG1 ILE A 116       7.359  -6.021  27.239  1.00  0.00           C
ATOM   1870 1HG1 ILE A 116       6.543  -5.565  26.882  1.00  0.00
ATOM   1871 2HG1 ILE A 116       7.555  -6.832  26.688  1.00  0.00
ATOM   1872  CD  ILE A 116       7.065  -6.470  28.636  1.00  0.00           C
ATOM   1873  HD1 ILE A 116       6.283  -7.093  28.628  1.00  0.00
ATOM   1874  HD2 ILE A 116       7.864  -6.942  29.010  1.00  0.00
ATOM   1875  HD3 ILE A 116       6.852  -5.675  29.204  1.00  0.00
ATOM   1876  C   ILE A 116      10.025  -3.769  25.540  1.00  0.00           C
ATOM   1877  O   ILE A 116       9.658  -2.734  25.009  1.00  0.00           O
ATOM   1878  N   ALA A 117      11.259  -3.960  25.997  1.00  0.00           N
ATOM   1879  HN  ALA A 117      11.477  -4.846  26.407  1.00  0.00
ATOM   1880  CA  ALA A 117      12.307  -2.961  25.937  1.00  0.00           C
ATOM   1881  HA  ALA A 117      11.960  -2.164  26.432  1.00  0.00
ATOM   1882  CB  ALA A 117      13.550  -3.493  26.609  1.00  0.00           C
ATOM   1883  HB1 ALA A 117      14.273  -2.803  26.567  1.00  0.00
ATOM   1884  HB2 ALA A 117      13.347  -3.706  27.565  1.00  0.00
ATOM   1885  HB3 ALA A 117      13.853  -4.322  26.140  1.00  0.00
ATOM   1886  C   ALA A 117      12.609  -2.572  24.495  1.00  0.00           C
ATOM   1887  O   ALA A 117      12.846  -1.398  24.187  1.00  0.00           O
ATOM   1888  N   ALA A 118      12.636  -3.567  23.606  1.00  0.00           N
ATOM   1889  HN  ALA A 118      12.482  -4.504  23.919  1.00  0.00
ATOM   1890  CA  ALA A 118      12.884  -3.324  22.191  1.00  0.00           C
ATOM   1891  HA  ALA A 118      13.771  -2.870  22.108  1.00  0.00
ATOM   1892  CB  ALA A 118      12.977  -4.631  21.465  1.00  0.00           C
ATOM   1893  HB1 ALA A 118      13.147  -4.462  20.494  1.00  0.00
ATOM   1894  HB2 ALA A 118      13.728  -5.171  21.846  1.00  0.00
ATOM   1895  HB3 ALA A 118      12.118  -5.133  21.570  1.00  0.00
ATOM   1896  C   ALA A 118      11.782  -2.418  21.619  1.00  0.00           C
ATOM   1897  O   ALA A 118      12.074  -1.507  20.832  1.00  0.00           O
ATOM   1898  N   ALA A 119      10.526  -2.642  22.052  1.00  0.00           N
ATOM   1899  HN  ALA A 119      10.371  -3.406  22.678  1.00  0.00
ATOM   1900  CA  ALA A 119       9.375  -1.827  21.656  1.00  0.00           C
ATOM   1901  HA  ALA A 119       9.358  -1.810  20.656  1.00  0.00
ATOM   1902  CB  ALA A 119       8.094  -2.457  22.152  1.00  0.00           C
ATOM   1903  HB1 ALA A 119       7.316  -1.892  21.876  1.00  0.00
ATOM   1904  HB2 ALA A 119       7.998  -3.371  21.759  1.00  0.00
ATOM   1905  HB3 ALA A 119       8.120  -2.524  23.149  1.00  0.00
ATOM   1906  C   ALA A 119       9.530  -0.407  22.198  1.00  0.00           C
ATOM   1907  O   ALA A 119       9.251   0.561  21.509  1.00  0.00           O
ATOM   1908  N   MET A 120       9.961  -0.305  23.458  1.00  0.00           N
ATOM   1909  HN  MET A 120      10.202  -1.139  23.955  1.00  0.00
ATOM   1910  CA  MET A 120      10.091   0.966  24.126  1.00  0.00           C
ATOM   1911  HA  MET A 120       9.194   1.403  24.057  1.00  0.00
ATOM   1912  CB  MET A 120      10.444   0.763  25.600  1.00  0.00           C
```

```
ATOM   1913  HB1 MET A 120      11.248   0.171  25.652  1.00  0.00
ATOM   1914  HB2 MET A 120      10.661   1.655  25.997  1.00  0.00
ATOM   1915  CG  MET A 120       9.331   0.130  26.428  1.00  0.00           C
ATOM   1916  HG1 MET A 120       9.444  -0.854  26.289  1.00  0.00
ATOM   1917  HG2 MET A 120       9.560   0.361  27.374  1.00  0.00
ATOM   1918  SD  MET A 120       7.675   0.694  25.974  1.00  0.00           S
ATOM   1919  CE  MET A 120       7.833   2.424  26.419  1.00  0.00           C
ATOM   1920  HE1 MET A 120       6.974   2.898  26.224  1.00  0.00
ATOM   1921  HE2 MET A 120       8.042   2.500  27.394  1.00  0.00
ATOM   1922  HE3 MET A 120       8.571   2.839  25.887  1.00  0.00
ATOM   1923  C   MET A 120      11.166   1.808  23.420  1.00  0.00           C
ATOM   1924  O   MET A 120      11.025   3.012  23.286  1.00  0.00           O
ATOM   1925  N   ARG A 121      12.241   1.162  22.966  1.00  0.00           N
ATOM   1926  HN  ARG A 121      12.303   0.173  23.098  1.00  0.00
ATOM   1927  CA  ARG A 121      13.345   1.861  22.273  1.00  0.00           C
ATOM   1928  HA  ARG A 121      13.756   2.511  22.912  1.00  0.00
ATOM   1929  CB  ARG A 121      14.452   0.876  21.872  1.00  0.00           C
ATOM   1930  HB1 ARG A 121      14.464   0.127  22.534  1.00  0.00
ATOM   1931  HB2 ARG A 121      14.240   0.515  20.964  1.00  0.00
ATOM   1932  CG  ARG A 121      15.848   1.496  21.827  1.00  0.00           C
ATOM   1933  HG1 ARG A 121      15.877   2.139  21.061  1.00  0.00
ATOM   1934  HG2 ARG A 121      15.995   1.991  22.683  1.00  0.00
ATOM   1935  CD  ARG A 121      16.995   0.499  21.653  1.00  0.00           C
ATOM   1936  HD1 ARG A 121      17.044   0.229  20.691  1.00  0.00
ATOM   1937  HD2 ARG A 121      17.851   0.943  21.918  1.00  0.00
ATOM   1938  NE  ARG A 121      16.884  -0.740  22.448  1.00  0.00           N
ATOM   1939  HE  ARG A 121      16.074  -1.307  22.299  1.00  0.00
ATOM   1940  CZ  ARG A 121      17.778  -1.173  23.355  1.00  0.00           C
ATOM   1941  NH1 ARG A 121      18.885  -0.479  23.578  1.00  0.00           N
ATOM   1942 1HH1 ARG A 121      19.056   0.366  23.072  1.00  0.00
ATOM   1943 2HH1 ARG A 121      19.550  -0.801  24.252  1.00  0.00
ATOM   1944  NH2 ARG A 121      17.553  -2.293  24.040  1.00  0.00           N
ATOM   1945 1HH2 ARG A 121      16.716  -2.817  23.881  1.00  0.00
ATOM   1946 2HH2 ARG A 121      18.221  -2.610  24.713  1.00  0.00
ATOM   1947  C   ARG A 121      12.766   2.635  21.086  1.00  0.00           C
ATOM   1948  O   ARG A 121      13.099   3.797  20.876  1.00  0.00           O
ATOM   1949  N   ILE A 122      11.833   2.004  20.365  1.00  0.00           N
ATOM   1950  HN  ILE A 122      11.562   1.078  20.627  1.00  0.00
ATOM   1951  CA  ILE A 122      11.197   2.627  19.205  1.00  0.00           C
ATOM   1952  HA  ILE A 122      11.941   2.986  18.642  1.00  0.00
ATOM   1953  CB  ILE A 122      10.407   1.598  18.358  1.00  0.00           C
ATOM   1954  HB  ILE A 122       9.625   1.293  18.901  1.00  0.00
ATOM   1955  CG2 ILE A 122       9.876   2.243  17.091  1.00  0.00           C
ATOM   1956 1HG2 ILE A 122       9.370   1.565  16.558  1.00  0.00
ATOM   1957 2HG2 ILE A 122       9.268   2.999  17.332  1.00  0.00
ATOM   1958 3HG2 ILE A 122      10.641   2.590  16.548  1.00  0.00
ATOM   1959  CG1 ILE A 122      11.220   0.342  18.034  1.00  0.00           C
ATOM   1960 1HG1 ILE A 122      11.459  -0.111  18.893  1.00  0.00
ATOM   1961 2HG1 ILE A 122      10.652  -0.269  17.482  1.00  0.00
ATOM   1962  CD  ILE A 122      12.503   0.609  17.270  1.00  0.00           C
ATOM   1963  HD1 ILE A 122      12.974  -0.256  17.097  1.00  0.00
ATOM   1964  HD2 ILE A 122      12.287   1.050  16.399  1.00  0.00
ATOM   1965  HD3 ILE A 122      13.094   1.209  17.810  1.00  0.00
ATOM   1966  C   ILE A 122      10.297   3.770  19.690  1.00  0.00           C
ATOM   1967  O   ILE A 122      10.352   4.852  19.161  1.00  0.00           O
ATOM   1968  N   LEU A 123       9.459   3.509  20.697  1.00  0.00           N
ATOM   1969  HN  LEU A 123       9.440   2.593  21.097  1.00  0.00
ATOM   1970  CA  LEU A 123       8.571   4.540  21.221  1.00  0.00           C
ATOM   1971  HA  LEU A 123       7.931   4.763  20.486  1.00  0.00
ATOM   1972  CB  LEU A 123       7.798   4.014  22.435  1.00  0.00           C
ATOM   1973  HB1 LEU A 123       8.467   3.693  23.105  1.00  0.00
ATOM   1974  HB2 LEU A 123       7.281   4.778  22.820  1.00  0.00
ATOM   1975  CG  LEU A 123       6.815   2.874  22.186  1.00  0.00           C
ATOM   1976  HG  LEU A 123       7.307   2.011  22.303  1.00  0.00
ATOM   1977  CD1 LEU A 123       5.669   2.953  23.187  1.00  0.00           C
ATOM   1978 1HD1 LEU A 123       5.028   2.204  23.020  1.00  0.00
ATOM   1979 2HD1 LEU A 123       6.032   2.880  24.116  1.00  0.00
ATOM   1980 3HD1 LEU A 123       5.195   3.827  23.083  1.00  0.00
ATOM   1981  CD2 LEU A 123       6.269   2.898  20.768  1.00  0.00           C
ATOM   1982 1HD2 LEU A 123       5.631   2.138  20.644  1.00  0.00
ATOM   1983 2HD2 LEU A 123       5.793   3.763  20.609  1.00  0.00
```

ATOM	1984	3HD2	LEU	A	123	7.024	2.808	20.119	1.00	0.00	
ATOM	1985	C	LEU	A	123	9.391	5.772	21.605	1.00	0.00	C
ATOM	1986	O	LEU	A	123	9.054	6.882	21.226	1.00	0.00	O
ATOM	1987	N	HIS	A	124	10.475	5.542	22.347	1.00	0.00	N
ATOM	1988	HN	HIS	A	124	10.736	4.591	22.515	1.00	0.00	
ATOM	1989	CA	HIS	A	124	11.312	6.603	22.935	1.00	0.00	C
ATOM	1990	HA	HIS	A	124	10.681	7.182	23.452	1.00	0.00	
ATOM	1991	CB	HIS	A	124	12.352	6.019	23.882	1.00	0.00	C
ATOM	1992	HB1	HIS	A	124	11.885	5.487	24.588	1.00	0.00	
ATOM	1993	HB2	HIS	A	124	12.963	5.421	23.364	1.00	0.00	
ATOM	1994	ND1	HIS	A	124	12.653	7.957	25.483	1.00	0.00	N
ATOM	1995	CG	HIS	A	124	13.185	7.054	24.560	1.00	0.00	C
ATOM	1996	CE1	HIS	A	124	13.634	8.716	25.950	1.00	0.00	C
ATOM	1997	HE1	HIS	A	124	13.538	9.449	26.624	1.00	0.00	
ATOM	1998	NE2	HIS	A	124	14.786	8.329	25.358	1.00	0.00	N
ATOM	1999	HE2	HIS	A	124	15.687	8.732	25.518	1.00	0.00	
ATOM	2000	CD2	HIS	A	124	14.515	7.295	24.503	1.00	0.00	C
ATOM	2001	HD2	HIS	A	124	15.180	6.806	23.938	1.00	0.00	
ATOM	2002	C	HIS	A	124	12.001	7.429	21.845	1.00	0.00	C
ATOM	2003	O	HIS	A	124	12.120	8.640	21.963	1.00	0.00	O
ATOM	2004	N	SER	A	125	12.454	6.763	20.786	1.00	0.00	N
ATOM	2005	HN	SER	A	125	12.303	5.776	20.733	1.00	0.00	
ATOM	2006	CA	SER	A	125	13.154	7.420	19.716	1.00	0.00	C
ATOM	2007	HA	SER	A	125	13.839	7.998	20.160	1.00	0.00	
ATOM	2008	CB	SER	A	125	13.880	6.425	18.876	1.00	0.00	C
ATOM	2009	HB1	SER	A	125	14.461	6.889	18.207	1.00	0.00	
ATOM	2010	HB2	SER	A	125	14.442	5.827	19.448	1.00	0.00	
ATOM	2011	OG	SER	A	125	12.956	5.623	18.176	1.00	0.00	O
ATOM	2012	HG1	SER	A	125	13.449	4.958	17.615	1.00	0.00	
ATOM	2013	C	SER	A	125	12.194	8.283	18.881	1.00	0.00	C
ATOM	2014	O	SER	A	125	12.623	9.252	18.275	1.00	0.00	O
ATOM	2015	N	LYS	A	126	10.902	7.941	18.872	1.00	0.00	N
ATOM	2016	HN	LYS	A	126	10.625	7.113	19.360	1.00	0.00	
ATOM	2017	CA	LYS	A	126	9.860	8.731	18.172	1.00	0.00	C
ATOM	2018	HA	LYS	A	126	10.358	9.208	17.448	1.00	0.00	
ATOM	2019	CB	LYS	A	126	8.765	7.828	17.598	1.00	0.00	C
ATOM	2020	HB1	LYS	A	126	8.241	7.457	18.365	1.00	0.00	
ATOM	2021	HB2	LYS	A	126	8.168	8.394	17.030	1.00	0.00	
ATOM	2022	CG	LYS	A	126	9.228	6.654	16.748	1.00	0.00	C
ATOM	2023	HG1	LYS	A	126	10.030	6.241	17.180	1.00	0.00	
ATOM	2024	HG2	LYS	A	126	8.490	5.980	16.702	1.00	0.00	
ATOM	2025	CD	LYS	A	126	9.600	7.024	15.348	1.00	0.00	C
ATOM	2026	HD1	LYS	A	126	8.849	7.546	14.944	1.00	0.00	
ATOM	2027	HD2	LYS	A	126	10.424	7.590	15.374	1.00	0.00	
ATOM	2028	CE	LYS	A	126	9.866	5.812	14.498	1.00	0.00	C
ATOM	2029	HE1	LYS	A	126	10.616	5.281	14.893	1.00	0.00	
ATOM	2030	HE2	LYS	A	126	9.044	5.245	14.452	1.00	0.00	
ATOM	2031	NZ	LYS	A	126	10.240	6.227	13.130	1.00	0.00	N
ATOM	2032	HZ1	LYS	A	126	10.415	5.416	12.572	1.00	0.00	
ATOM	2033	HZ2	LYS	A	126	9.493	6.755	12.726	1.00	0.00	
ATOM	2034	HZ3	LYS	A	126	11.065	6.791	13.167	1.00	0.00	
ATOM	2035	C	LYS	A	126	9.168	9.710	19.129	1.00	0.00	C
ATOM	2036	O	LYS	A	126	8.267	10.415	18.724	1.00	0.00	O
ATOM	2037	N	GLY	A	127	9.536	9.680	20.415	1.00	0.00	N
ATOM	2038	HN	GLY	A	127	10.235	9.022	20.696	1.00	0.00	
ATOM	2039	CA	GLY	A	127	8.963	10.566	21.431	1.00	0.00	C
ATOM	2040	HA1	GLY	A	127	9.490	10.443	22.272	1.00	0.00	
ATOM	2041	HA2	GLY	A	127	9.067	11.506	21.107	1.00	0.00	
ATOM	2042	C	GLY	A	127	7.488	10.282	21.707	1.00	0.00	C
ATOM	2043	O	GLY	A	127	6.728	11.205	21.964	1.00	0.00	O
ATOM	2044	N	ILE	A	128	7.115	8.993	21.732	1.00	0.00	N
ATOM	2045	HN	ILE	A	128	7.809	8.296	21.555	1.00	0.00	
ATOM	2046	CA	ILE	A	128	5.758	8.558	22.002	1.00	0.00	C
ATOM	2047	HA	ILE	A	128	5.200	9.387	21.994	1.00	0.00	
ATOM	2048	CB	ILE	A	128	5.262	7.595	20.914	1.00	0.00	C
ATOM	2049	HB	ILE	A	128	5.920	6.847	20.826	1.00	0.00	
ATOM	2050	CG2	ILE	A	128	3.913	6.986	21.311	1.00	0.00	C
ATOM	2051	1HG2	ILE	A	128	3.605	6.362	20.593	1.00	0.00	
ATOM	2052	2HG2	ILE	A	128	4.014	6.482	22.169	1.00	0.00	
ATOM	2053	3HG2	ILE	A	128	3.240	7.716	21.430	1.00	0.00	
ATOM	2054	CG1	ILE	A	128	5.202	8.280	19.551	1.00	0.00	C

ATOM	2055	1HG1	ILE	A	128	4.460	8.950	19.565	1.00	0.00	
ATOM	2056	2HG1	ILE	A	128	6.071	8.748	19.392	1.00	0.00	
ATOM	2057	CD	ILE	A	128	4.961	7.346	18.394	1.00	0.00	C
ATOM	2058	HD1	ILE	A	128	4.936	7.870	17.543	1.00	0.00	
ATOM	2059	HD2	ILE	A	128	5.699	6.673	18.349	1.00	0.00	
ATOM	2060	HD3	ILE	A	128	4.088	6.875	18.522	1.00	0.00	
ATOM	2061	C	ILE	A	128	5.709	7.880	23.372	1.00	0.00	C
ATOM	2062	O	ILE	A	128	6.517	7.017	23.678	1.00	0.00	O
ATOM	2063	N	ILE	A	129	4.724	8.282	24.167	1.00	0.00	N
ATOM	2064	HN	ILE	A	129	4.139	9.027	23.846	1.00	0.00	
ATOM	2065	CA	ILE	A	129	4.445	7.717	25.456	1.00	0.00	C
ATOM	2066	HA	ILE	A	129	5.204	7.103	25.671	1.00	0.00	
ATOM	2067	CB	ILE	A	129	4.362	8.809	26.543	1.00	0.00	C
ATOM	2068	HB	ILE	A	129	3.546	9.357	26.361	1.00	0.00	
ATOM	2069	CG2	ILE	A	129	4.209	8.204	27.920	1.00	0.00	C
ATOM	2070	1HG2	ILE	A	129	4.158	8.934	28.601	1.00	0.00	
ATOM	2071	2HG2	ILE	A	129	3.372	7.658	27.953	1.00	0.00	
ATOM	2072	3HG2	ILE	A	129	4.996	7.619	28.116	1.00	0.00	
ATOM	2073	CG1	ILE	A	129	5.557	9.750	26.504	1.00	0.00	C
ATOM	2074	1HG1	ILE	A	129	6.353	9.284	26.891	1.00	0.00	
ATOM	2075	2HG1	ILE	A	129	5.746	9.999	25.554	1.00	0.00	
ATOM	2076	CD	ILE	A	129	5.326	11.006	27.281	1.00	0.00	C
ATOM	2077	HD1	ILE	A	129	6.138	11.587	27.224	1.00	0.00	
ATOM	2078	HD2	ILE	A	129	4.540	11.493	26.901	1.00	0.00	
ATOM	2079	HD3	ILE	A	129	5.146	10.778	28.238	1.00	0.00	
ATOM	2080	C	ILE	A	129	3.124	6.970	25.340	1.00	0.00	C
ATOM	2081	O	ILE	A	129	2.142	7.529	24.877	1.00	0.00	O
ATOM	2082	N	HIS	A	130	3.102	5.726	25.814	1.00	0.00	N
ATOM	2083	HN	HIS	A	130	3.921	5.369	26.263	1.00	0.00	
ATOM	2084	CA	HIS	A	130	1.941	4.870	25.706	1.00	0.00	C
ATOM	2085	HA	HIS	A	130	1.535	5.029	24.806	1.00	0.00	
ATOM	2086	CB	HIS	A	130	2.395	3.411	25.803	1.00	0.00	C
ATOM	2087	HB1	HIS	A	130	3.108	3.244	25.122	1.00	0.00	
ATOM	2088	HB2	HIS	A	130	2.762	3.243	26.718	1.00	0.00	
ATOM	2089	ND1	HIS	A	130	0.248	2.267	26.444	1.00	0.00	N
ATOM	2090	CG	HIS	A	130	1.303	2.431	25.566	1.00	0.00	C
ATOM	2091	CE1	HIS	A	130	-0.550	1.336	25.979	1.00	0.00	C
ATOM	2092	HE1	HIS	A	130	-1.393	1.014	26.410	1.00	0.00	
ATOM	2093	NE2	HIS	A	130	-0.035	0.892	24.821	1.00	0.00	N
ATOM	2094	HE2	HIS	A	130	-0.432	0.178	24.244	1.00	0.00	
ATOM	2095	CD2	HIS	A	130	1.108	1.568	24.562	1.00	0.00	C
ATOM	2096	HD2	HIS	A	130	1.700	1.445	23.765	1.00	0.00	
ATOM	2097	C	HIS	A	130	0.895	5.250	26.764	1.00	0.00	C
ATOM	2098	O	HIS	A	130	-0.266	5.437	26.437	1.00	0.00	O
ATOM	2099	N	ARG	A	131	1.331	5.296	28.029	1.00	0.00	N
ATOM	2100	HN	ARG	A	131	2.251	4.947	28.207	1.00	0.00	
ATOM	2101	CA	ARG	A	131	0.574	5.816	29.185	1.00	0.00	C
ATOM	2102	HA	ARG	A	131	1.282	5.963	29.876	1.00	0.00	
ATOM	2103	CB	ARG	A	131	-0.120	7.142	28.864	1.00	0.00	C
ATOM	2104	HB1	ARG	A	131	-0.782	6.987	28.131	1.00	0.00	
ATOM	2105	HB2	ARG	A	131	-0.597	7.460	29.683	1.00	0.00	
ATOM	2106	CG	ARG	A	131	0.838	8.232	28.418	1.00	0.00	C
ATOM	2107	HG1	ARG	A	131	1.727	8.081	28.850	1.00	0.00	
ATOM	2108	HG2	ARG	A	131	0.939	8.190	27.424	1.00	0.00	
ATOM	2109	CD	ARG	A	131	0.346	9.605	28.795	1.00	0.00	C
ATOM	2110	HD1	ARG	A	131	0.335	9.694	29.791	1.00	0.00	
ATOM	2111	HD2	ARG	A	131	0.954	10.296	28.405	1.00	0.00	
ATOM	2112	NE	ARG	A	131	-0.995	9.870	28.311	1.00	0.00	N
ATOM	2113	HE	ARG	A	131	-1.336	9.310	27.556	1.00	0.00	
ATOM	2114	CZ	ARG	A	131	-1.797	10.799	28.798	1.00	0.00	C
ATOM	2115	NH1	ARG	A	131	-1.388	11.595	29.775	1.00	0.00	N
ATOM	2116	1HH1	ARG	A	131	-0.466	11.495	30.149	1.00	0.00	
ATOM	2117	2HH1	ARG	A	131	-2.001	12.296	30.139	1.00	0.00	
ATOM	2118	NH2	ARG	A	131	-3.013	10.917	28.302	1.00	0.00	N
ATOM	2119	1HH2	ARG	A	131	-3.315	10.308	27.568	1.00	0.00	
ATOM	2120	2HH2	ARG	A	131	-3.633	11.615	28.659	1.00	0.00	
ATOM	2121	C	ARG	A	131	-0.456	4.809	29.719	1.00	0.00	C
ATOM	2122	O	ARG	A	131	-1.005	5.023	30.787	1.00	0.00	O
ATOM	2123	N	ASP	A	132	-0.673	3.699	29.019	1.00	0.00	N
ATOM	2124	HN	ASP	A	132	-0.113	3.518	28.210	1.00	0.00	
ATOM	2125	CA	ASP	A	132	-1.701	2.739	29.395	1.00	0.00	C

```
ATOM   2126  HA  ASP A 132      -1.902   2.885  30.364  1.00  0.00
ATOM   2127  CB  ASP A 132      -2.964   3.000  28.585  1.00  0.00           C
ATOM   2128  HB1 ASP A 132      -3.132   3.986  28.591  1.00  0.00
ATOM   2129  HB2 ASP A 132      -2.793   2.697  27.647  1.00  0.00
ATOM   2130  CG  ASP A 132      -4.230   2.308  29.075  1.00  0.00           C
ATOM   2131  OD1 ASP A 132      -4.342   2.046  30.321  1.00  0.00           O
ATOM   2132  OD2 ASP A 132      -5.115   2.037  28.199  1.00  0.00           O
ATOM   2133  C   ASP A 132      -1.180   1.310  29.205  1.00  0.00           C
ATOM   2134  O   ASP A 132      -1.948   0.418  28.900  1.00  0.00           O
ATOM   2135  N   LEU A 133       0.125   1.101  29.401  1.00  0.00           N
ATOM   2136  HN  LEU A 133       0.725   1.880  29.582  1.00  0.00
ATOM   2137  CA  LEU A 133       0.691  -0.240  29.356  1.00  0.00           C
ATOM   2138  HA  LEU A 133       0.420  -0.630  28.476  1.00  0.00
ATOM   2139  CB  LEU A 133       2.213  -0.201  29.457  1.00  0.00           C
ATOM   2140  HB1 LEU A 133       2.456   0.476  30.152  1.00  0.00
ATOM   2141  HB2 LEU A 133       2.523  -1.106  29.749  1.00  0.00
ATOM   2142  CG  LEU A 133       2.970   0.153  28.194  1.00  0.00           C
ATOM   2143  HG  LEU A 133       2.732   1.088  27.930  1.00  0.00
ATOM   2144  CD1 LEU A 133       4.469   0.058  28.462  1.00  0.00           C
ATOM   2145 1HD1 LEU A 133       4.972   0.291  27.630  1.00  0.00
ATOM   2146 2HD1 LEU A 133       4.718   0.695  29.192  1.00  0.00
ATOM   2147 3HD1 LEU A 133       4.699  -0.874  28.740  1.00  0.00
ATOM   2148  CD2 LEU A 133       2.574  -0.759  27.047  1.00  0.00           C
ATOM   2149 1HD2 LEU A 133       3.087  -0.505  26.227  1.00  0.00
ATOM   2150 2HD2 LEU A 133       2.780  -1.707  27.289  1.00  0.00
ATOM   2151 3HD2 LEU A 133       1.594  -0.666  26.870  1.00  0.00
ATOM   2152  C   LEU A 133       0.144  -1.031  30.539  1.00  0.00           C
ATOM   2153  O   LEU A 133       0.164  -0.569  31.664  1.00  0.00           O
ATOM   2154  N   LYS A 134      -0.327  -2.233  30.253  1.00  0.00           N
ATOM   2155  HN  LYS A 134      -0.325  -2.527  29.297  1.00  0.00
ATOM   2156  CA  LYS A 134      -0.833  -3.125  31.226  1.00  0.00           C
ATOM   2157  HA  LYS A 134      -0.173  -3.155  31.977  1.00  0.00
ATOM   2158  CB  LYS A 134      -2.162  -2.599  31.775  1.00  0.00           C
ATOM   2159  HB1 LYS A 134      -2.476  -3.230  32.485  1.00  0.00
ATOM   2160  HB2 LYS A 134      -1.997  -1.699  32.178  1.00  0.00
ATOM   2161  CG  LYS A 134      -3.284  -2.449  30.772  1.00  0.00           C
ATOM   2162  HG1 LYS A 134      -2.984  -1.835  30.042  1.00  0.00
ATOM   2163  HG2 LYS A 134      -3.495  -3.348  30.387  1.00  0.00
ATOM   2164  CD  LYS A 134      -4.537  -1.883  31.397  1.00  0.00           C
ATOM   2165  HD1 LYS A 134      -4.766  -2.432  32.200  1.00  0.00
ATOM   2166  HD2 LYS A 134      -4.353  -0.941  31.679  1.00  0.00
ATOM   2167  CE  LYS A 134      -5.725  -1.882  30.459  1.00  0.00           C
ATOM   2168  HE1 LYS A 134      -5.399  -1.848  29.514  1.00  0.00
ATOM   2169  HE2 LYS A 134      -6.256  -2.718  30.598  1.00  0.00
ATOM   2170  NZ  LYS A 134      -6.615  -0.717  30.691  1.00  0.00           N
ATOM   2171  HZ1 LYS A 134      -7.385  -0.753  30.054  1.00  0.00
ATOM   2172  HZ2 LYS A 134      -6.958  -0.742  31.630  1.00  0.00
ATOM   2173  HZ3 LYS A 134      -6.102   0.129  30.546  1.00  0.00
ATOM   2174  C   LYS A 134      -0.945  -4.500  30.586  1.00  0.00           C
ATOM   2175  O   LYS A 134      -0.811  -4.625  29.383  1.00  0.00           O
ATOM   2176  N   PRO A 135      -1.133  -5.580  31.368  1.00  0.00           N
ATOM   2177  CD  PRO A 135      -1.237  -5.574  32.836  1.00  0.00           C
ATOM   2178  HD1 PRO A 135      -2.162  -5.326  33.123  1.00  0.00
ATOM   2179  HD2 PRO A 135      -0.581  -4.933  33.234  1.00  0.00
ATOM   2180  CA  PRO A 135      -1.170  -6.932  30.814  1.00  0.00           C
ATOM   2181  HA  PRO A 135      -0.302  -7.118  30.354  1.00  0.00
ATOM   2182  CB  PRO A 135      -1.451  -7.811  32.038  1.00  0.00           C
ATOM   2183  HB1 PRO A 135      -2.432  -7.967  32.152  1.00  0.00
ATOM   2184  HB2 PRO A 135      -0.980  -8.690  31.967  1.00  0.00
ATOM   2185  CG  PRO A 135      -0.905  -7.010  33.193  1.00  0.00           C
ATOM   2186  HG1 PRO A 135      -1.348  -7.276  34.049  1.00  0.00
ATOM   2187  HG2 PRO A 135       0.083  -7.138  33.276  1.00  0.00
ATOM   2188  C   PRO A 135      -2.244  -7.123  29.734  1.00  0.00           C
ATOM   2189  O   PRO A 135      -2.032  -7.896  28.806  1.00  0.00           O
ATOM   2190  N   GLN A 136      -3.359  -6.393  29.847  1.00  0.00           N
ATOM   2191  HN  GLN A 136      -3.442  -5.758  30.615  1.00  0.00
ATOM   2192  CA  GLN A 136      -4.475  -6.488  28.879  1.00  0.00           C
ATOM   2193  HA  GLN A 136      -4.691  -7.460  28.785  1.00  0.00
ATOM   2194  CB  GLN A 136      -5.721  -5.777  29.417  1.00  0.00           C
ATOM   2195  HB1 GLN A 136      -5.459  -4.849  29.681  1.00  0.00
ATOM   2196  HB2 GLN A 136      -6.397  -5.736  28.681  1.00  0.00
```

```
ATOM   2197  CG  GLN A 136      -6.377  -6.448  30.627  1.00  0.00           C
ATOM   2198  HG1 GLN A 136      -7.330  -6.148  30.663  1.00  0.00
ATOM   2199  HG2 GLN A 136      -6.344  -7.437  30.483  1.00  0.00
ATOM   2200  CD  GLN A 136      -5.748  -6.157  31.989  1.00  0.00           C
ATOM   2201  OE1 GLN A 136      -4.798  -5.374  32.150  1.00  0.00           O
ATOM   2202  NE2 GLN A 136      -6.274  -6.834  33.007  1.00  0.00           N
ATOM   2203 1HE2 GLN A 136      -7.024  -7.475  32.846  1.00  0.00
ATOM   2204 2HE2 GLN A 136      -5.919  -6.701  33.932  1.00  0.00
ATOM   2205  C   GLN A 136      -4.038  -5.913  27.507  1.00  0.00           C
ATOM   2206  O   GLN A 136      -4.591  -6.258  26.477  1.00  0.00           O
ATOM   2207  N   ASN A 137      -3.011  -5.056  27.510  1.00  0.00           N
ATOM   2208  HN  ASN A 137      -2.531  -4.904  28.374  1.00  0.00
ATOM   2209  CA  ASN A 137      -2.548  -4.330  26.335  1.00  0.00           C
ATOM   2210  HA  ASN A 137      -3.257  -4.455  25.641  1.00  0.00
ATOM   2211  CB  ASN A 137      -2.417  -2.838  26.642  1.00  0.00           C
ATOM   2212  HB1 ASN A 137      -1.986  -2.726  27.538  1.00  0.00
ATOM   2213  HB2 ASN A 137      -1.846  -2.410  25.942  1.00  0.00
ATOM   2214  CG  ASN A 137      -3.755  -2.137  26.663  1.00  0.00           C
ATOM   2215  OD1 ASN A 137      -4.759  -2.697  26.224  1.00  0.00           O
ATOM   2216  ND2 ASN A 137      -3.784  -0.911  27.165  1.00  0.00           N
ATOM   2217 1HD2 ASN A 137      -2.944  -0.491  27.508  1.00  0.00
ATOM   2218 2HD2 ASN A 137      -4.646  -0.406  27.201  1.00  0.00
ATOM   2219  C   ASN A 137      -1.224  -4.921  25.824  1.00  0.00           C
ATOM   2220  O   ASN A 137      -0.583  -4.346  24.974  1.00  0.00           O
ATOM   2221  N   ILE A 138      -0.835  -6.083  26.342  1.00  0.00           N
ATOM   2222  HN  ILE A 138      -1.375  -6.496  27.075  1.00  0.00
ATOM   2223  CA  ILE A 138       0.353  -6.765  25.869  1.00  0.00           C
ATOM   2224  HA  ILE A 138       0.762  -6.197  25.155  1.00  0.00
ATOM   2225  CB  ILE A 138       1.382  -6.911  26.994  1.00  0.00           C
ATOM   2226  HB  ILE A 138       0.950  -7.376  27.766  1.00  0.00
ATOM   2227  CG2 ILE A 138       2.537  -7.768  26.541  1.00  0.00           C
ATOM   2228 1HG2 ILE A 138       3.200  -7.854  27.285  1.00  0.00
ATOM   2229 2HG2 ILE A 138       2.200  -8.675  26.288  1.00  0.00
ATOM   2230 3HG2 ILE A 138       2.977  -7.343  25.750  1.00  0.00
ATOM   2231  CG1 ILE A 138       1.858  -5.552  27.497  1.00  0.00           C
ATOM   2232 1HG1 ILE A 138       2.465  -5.154  26.809  1.00  0.00
ATOM   2233 2HG1 ILE A 138       1.060  -4.962  27.620  1.00  0.00
ATOM   2234  CD  ILE A 138       2.607  -5.607  28.808  1.00  0.00           C
ATOM   2235  HD1 ILE A 138       2.885  -4.683  29.072  1.00  0.00
ATOM   2236  HD2 ILE A 138       2.013  -5.991  29.515  1.00  0.00
ATOM   2237  HD3 ILE A 138       3.418  -6.183  28.705  1.00  0.00
ATOM   2238  C   ILE A 138      -0.075  -8.117  25.297  1.00  0.00           C
ATOM   2239  O   ILE A 138      -0.567  -8.961  26.010  1.00  0.00           O
ATOM   2240  N   LEU A 139       0.095  -8.276  23.992  1.00  0.00           N
ATOM   2241  HN  LEU A 139       0.607  -7.582  23.486  1.00  0.00
ATOM   2242  CA  LEU A 139      -0.431  -9.412  23.274  1.00  0.00           C
ATOM   2243  HA  LEU A 139      -1.121  -9.833  23.862  1.00  0.00
ATOM   2244  CB  LEU A 139      -1.059  -8.931  21.963  1.00  0.00           C
ATOM   2245  HB1 LEU A 139      -0.368  -8.412  21.460  1.00  0.00
ATOM   2246  HB2 LEU A 139      -1.323  -9.736  21.432  1.00  0.00
ATOM   2247  CG  LEU A 139      -2.290  -8.046  22.113  1.00  0.00           C
ATOM   2248  HG  LEU A 139      -2.034  -7.243  22.652  1.00  0.00
ATOM   2249  CD1 LEU A 139      -2.770  -7.566  20.753  1.00  0.00           C
ATOM   2250 1HD1 LEU A 139      -3.577  -6.987  20.870  1.00  0.00
ATOM   2251 2HD1 LEU A 139      -2.044  -7.040  20.310  1.00  0.00
ATOM   2252 3HD1 LEU A 139      -3.005  -8.355  20.185  1.00  0.00
ATOM   2253  CD2 LEU A 139      -3.405  -8.782  22.857  1.00  0.00           C
ATOM   2254 1HD2 LEU A 139      -4.201  -8.182  22.943  1.00  0.00
ATOM   2255 2HD2 LEU A 139      -3.660  -9.603  22.347  1.00  0.00
ATOM   2256 3HD2 LEU A 139      -3.083  -9.042  23.768  1.00  0.00
ATOM   2257  C   LEU A 139       0.690 -10.407  22.999  1.00  0.00           C
ATOM   2258  O   LEU A 139       1.876 -10.043  22.994  1.00  0.00           O
ATOM   2259  N   LEU A 140       0.276 -11.651  22.745  1.00  0.00           N
ATOM   2260  HN  LEU A 140      -0.703 -11.803  22.609  1.00  0.00
ATOM   2261  CA  LEU A 140       1.166 -12.795  22.655  1.00  0.00           C
ATOM   2262  HA  LEU A 140       2.104 -12.458  22.733  1.00  0.00
ATOM   2263  CB  LEU A 140       0.819 -13.755  23.797  1.00  0.00           C
ATOM   2264  HB1 LEU A 140      -0.153 -13.649  24.007  1.00  0.00
ATOM   2265  HB2 LEU A 140       0.992 -14.688  23.482  1.00  0.00
ATOM   2266  CG  LEU A 140       1.596 -13.550  25.086  1.00  0.00           C
ATOM   2267  HG  LEU A 140       2.558 -13.764  24.915  1.00  0.00
```

```
ATOM   2268  CD1 LEU A 140       1.539 -12.109  25.542  1.00  0.00           C
ATOM   2269 1HD1 LEU A 140       2.058 -12.007  26.390  1.00  0.00
ATOM   2270 2HD1 LEU A 140       1.934 -11.520  24.837  1.00  0.00
ATOM   2271 3HD1 LEU A 140       0.587 -11.847  25.700  1.00  0.00
ATOM   2272  CD2 LEU A 140       1.068 -14.483  26.161  1.00  0.00           C
ATOM   2273 1HD2 LEU A 140       1.583 -14.343  27.006  1.00  0.00
ATOM   2274 2HD2 LEU A 140       0.101 -14.290  26.325  1.00  0.00
ATOM   2275 3HD2 LEU A 140       1.172 -15.431  25.860  1.00  0.00
ATOM   2276  C   LEU A 140       0.986 -13.488  21.305  1.00  0.00           C
ATOM   2277  O   LEU A 140      -0.133 -13.734  20.886  1.00  0.00           O
ATOM   2278  N   SER A 141       2.106 -13.795  20.653  1.00  0.00           N
ATOM   2279  HN  SER A 141       2.981 -13.512  21.046  1.00  0.00
ATOM   2280  CA  SER A 141       2.114 -14.524  19.394  1.00  0.00           C
ATOM   2281  HA  SER A 141       1.189 -14.875  19.248  1.00  0.00
ATOM   2282  CB  SER A 141       2.428 -13.597  18.247  1.00  0.00           C
ATOM   2283  HB1 SER A 141       1.698 -12.923  18.134  1.00  0.00
ATOM   2284  HB2 SER A 141       3.295 -13.126  18.407  1.00  0.00
ATOM   2285  OG  SER A 141       2.542 -14.332  17.032  1.00  0.00           O
ATOM   2286  HG1 SER A 141       2.750 -13.705  16.281  1.00  0.00
ATOM   2287  C   SER A 141       3.119 -15.683  19.489  1.00  0.00           C
ATOM   2288  O   SER A 141       4.265 -15.465  19.867  1.00  0.00           O
ATOM   2289  N   TYR A 142       2.666 -16.901  19.161  1.00  0.00           N
ATOM   2290  HN  TYR A 142       1.721 -16.983  18.845  1.00  0.00
ATOM   2291  CA  TYR A 142       3.489 -18.138  19.240  1.00  0.00           C
ATOM   2292  HA  TYR A 142       4.249 -17.922  19.853  1.00  0.00
ATOM   2293  CB  TYR A 142       2.663 -19.317  19.775  1.00  0.00           C
ATOM   2294  HB1 TYR A 142       1.852 -19.410  19.197  1.00  0.00
ATOM   2295  HB2 TYR A 142       3.223 -20.142  19.700  1.00  0.00
ATOM   2296  CG  TYR A 142       2.180 -19.240  21.204  1.00  0.00           C
ATOM   2297  CD1 TYR A 142       1.045 -18.522  21.554  1.00  0.00           C
ATOM   2298  HD1 TYR A 142       0.534 -18.039  20.843  1.00  0.00
ATOM   2299  CE1 TYR A 142       0.603 -18.459  22.867  1.00  0.00           C
ATOM   2300  HE1 TYR A 142      -0.191 -17.898  23.101  1.00  0.00
ATOM   2301  CZ  TYR A 142       1.262 -19.173  23.854  1.00  0.00           C
ATOM   2302  OH  TYR A 142       0.805 -19.127  25.137  1.00  0.00           O
ATOM   2303  HH  TYR A 142       1.389 -19.693  25.719  1.00  0.00
ATOM   2304  CD2 TYR A 142       2.822 -19.951  22.207  1.00  0.00           C
ATOM   2305  HD2 TYR A 142       3.636 -20.486  21.980  1.00  0.00
ATOM   2306  CE2 TYR A 142       2.365 -19.940  23.519  1.00  0.00           C
ATOM   2307  HE2 TYR A 142       2.830 -20.483  24.218  1.00  0.00
ATOM   2308  C   TYR A 142       3.960 -18.530  17.835  1.00  0.00           C
ATOM   2309  O   TYR A 142       3.173 -18.461  16.901  1.00  0.00           O
ATOM   2310  N   ALA A 143       5.194 -19.033  17.733  1.00  0.00           N
ATOM   2311  HN  ALA A 143       5.738 -19.122  18.567  1.00  0.00
ATOM   2312  CA  ALA A 143       5.802 -19.468  16.448  1.00  0.00           C
ATOM   2313  HA  ALA A 143       5.529 -18.796  15.759  1.00  0.00
ATOM   2314  CB  ALA A 143       7.316 -19.457  16.551  1.00  0.00           C
ATOM   2315  HB1 ALA A 143       7.711 -19.751  15.681  1.00  0.00
ATOM   2316  HB2 ALA A 143       7.629 -18.531  16.763  1.00  0.00
ATOM   2317  HB3 ALA A 143       7.605 -20.081  17.277  1.00  0.00
ATOM   2318  C   ALA A 143       5.278 -20.855  16.025  1.00  0.00           C
ATOM   2319  O   ALA A 143       4.593 -20.967  15.025  1.00  0.00           O
ATOM   2320  N   ASN A 144       5.445 -21.883  16.893  1.00  0.00           N
ATOM   2321  HN  ASN A 144       5.888 -21.714  17.774  1.00  0.00
ATOM   2322  CA  ASN A 144       4.998 -23.209  16.572  1.00  0.00           C
ATOM   2323  HA  ASN A 144       4.893 -23.178  15.578  1.00  0.00
ATOM   2324  CB  ASN A 144       5.987 -24.323  16.987  1.00  0.00           C
ATOM   2325  HB1 ASN A 144       5.610 -25.217  16.746  1.00  0.00
ATOM   2326  HB2 ASN A 144       6.860 -24.192  16.517  1.00  0.00
ATOM   2327  CG  ASN A 144       6.212 -24.261  18.497  1.00  0.00           C
ATOM   2328  OD1 ASN A 144       6.660 -23.239  19.016  1.00  0.00           O
ATOM   2329  ND2 ASN A 144       5.875 -25.367  19.216  1.00  0.00           N
ATOM   2330 1HD2 ASN A 144       5.499 -26.170  18.752  1.00  0.00
ATOM   2331 2HD2 ASN A 144       6.003 -25.377  20.208  1.00  0.00
ATOM   2332  C   ASN A 144       3.715 -23.466  17.290  1.00  0.00           C
ATOM   2333  O   ASN A 144       3.431 -22.907  18.345  1.00  0.00           O
ATOM   2334  N   ARG A 145       2.869 -24.317  16.704  1.00  0.00           N
ATOM   2335  HN  ARG A 145       3.068 -24.695  15.800  1.00  0.00
ATOM   2336  CA  ARG A 145       1.674 -24.675  17.398  1.00  0.00           C
ATOM   2337  HA  ARG A 145       1.751 -24.215  18.283  1.00  0.00
ATOM   2338  CB  ARG A 145       0.365 -24.218  16.725  1.00  0.00           C
```

ATOM	2339	HB1	ARG	A	145	0.465	-24.307	15.734	1.00	0.00	
ATOM	2340	HB2	ARG	A	145	-0.380	-24.806	17.039	1.00	0.00	
ATOM	2341	CG	ARG	A	145	0.004	-22.767	17.046	1.00	0.00	C
ATOM	2342	HG1	ARG	A	145	0.675	-22.164	16.615	1.00	0.00	
ATOM	2343	HG2	ARG	A	145	-0.905	-22.571	16.678	1.00	0.00	
ATOM	2344	CD	ARG	A	145	-0.004	-22.502	18.554	1.00	0.00	C
ATOM	2345	HD1	ARG	A	145	-0.487	-23.235	19.033	1.00	0.00	
ATOM	2346	HD2	ARG	A	145	0.932	-22.437	18.901	1.00	0.00	
ATOM	2347	NE	ARG	A	145	-0.706	-21.214	18.796	1.00	0.00	N
ATOM	2348	HE	ARG	A	145	-1.055	-20.673	18.031	1.00	0.00	
ATOM	2349	CZ	ARG	A	145	-0.865	-20.784	20.079	1.00	0.00	C
ATOM	2350	NH1	ARG	A	145	-0.344	-21.525	21.099	1.00	0.00	N
ATOM	2351	1HH1	ARG	A	145	0.148	-22.373	20.901	1.00	0.00	
ATOM	2352	2HH1	ARG	A	145	-0.454	-21.220	22.045	1.00	0.00	
ATOM	2353	NH2	ARG	A	145	-1.523	-19.622	20.355	1.00	0.00	N
ATOM	2354	1HH2	ARG	A	145	-1.898	-19.071	19.610	1.00	0.00	
ATOM	2355	2HH2	ARG	A	145	-1.631	-19.321	21.303	1.00	0.00	
ATOM	2356	C	ARG	A	145	1.675	-26.162	17.471	1.00	0.00	C
ATOM	2357	O	ARG	A	145	2.514	-26.822	16.860	1.00	0.00	O
ATOM	2358	N	ARG	A	146	0.763	-26.718	18.301	1.00	0.00	N
ATOM	2359	HN	ARG	A	146	0.218	-26.088	18.854	1.00	0.00	
ATOM	2360	CA	ARG	A	146	0.494	-28.131	18.472	1.00	0.00	C
ATOM	2361	HA	ARG	A	146	-0.190	-28.111	19.201	1.00	0.00	
ATOM	2362	CB	ARG	A	146	-0.050	-28.776	17.171	1.00	0.00	C
ATOM	2363	HB1	ARG	A	146	-0.793	-28.205	16.822	1.00	0.00	
ATOM	2364	HB2	ARG	A	146	0.690	-28.807	16.500	1.00	0.00	
ATOM	2365	CG	ARG	A	146	-0.590	-30.197	17.338	1.00	0.00	C
ATOM	2366	HG1	ARG	A	146	0.054	-30.710	17.906	1.00	0.00	
ATOM	2367	HG2	ARG	A	146	-1.474	-30.142	17.803	1.00	0.00	
ATOM	2368	CD	ARG	A	146	-0.787	-30.963	16.029	1.00	0.00	C
ATOM	2369	HD1	ARG	A	146	-1.613	-30.654	15.557	1.00	0.00	
ATOM	2370	HD2	ARG	A	146	0.004	-30.848	15.427	1.00	0.00	
ATOM	2371	NE	ARG	A	146	-0.929	-32.394	16.404	1.00	0.00	N
ATOM	2372	HE	ARG	A	146	-1.823	-32.778	16.634	1.00	0.00	
ATOM	2373	CZ	ARG	A	146	0.192	-33.169	16.426	1.00	0.00	C
ATOM	2374	NH1	ARG	A	146	1.367	-32.674	15.943	1.00	0.00	N
ATOM	2375	1HH1	ARG	A	146	1.404	-31.745	15.574	1.00	0.00	
ATOM	2376	2HH1	ARG	A	146	2.191	-33.240	15.958	1.00	0.00	
ATOM	2377	NH2	ARG	A	146	0.143	-34.445	16.911	1.00	0.00	N
ATOM	2378	1HH2	ARG	A	146	-0.720	-34.817	17.253	1.00	0.00	
ATOM	2379	2HH2	ARG	A	146	0.970	-35.007	16.924	1.00	0.00	
ATOM	2380	C	ARG	A	146	1.687	-28.926	18.966	1.00	0.00	C
ATOM	2381	O	ARG	A	146	1.715	-30.154	18.841	1.00	0.00	O
ATOM	2382	N	LYS	A	147	2.704	-28.258	19.551	1.00	0.00	N
ATOM	2383	HN	LYS	A	147	2.635	-27.263	19.617	1.00	0.00	
ATOM	2384	CA	LYS	A	147	3.881	-28.891	20.086	1.00	0.00	C
ATOM	2385	HA	LYS	A	147	3.565	-29.769	20.446	1.00	0.00	
ATOM	2386	CB	LYS	A	147	5.024	-29.120	19.065	1.00	0.00	C
ATOM	2387	HB1	LYS	A	147	5.214	-28.246	18.619	1.00	0.00	
ATOM	2388	HB2	LYS	A	147	5.832	-29.418	19.573	1.00	0.00	
ATOM	2389	CG	LYS	A	147	4.775	-30.153	17.958	1.00	0.00	C
ATOM	2390	HG1	LYS	A	147	3.944	-29.890	17.468	1.00	0.00	
ATOM	2391	HG2	LYS	A	147	5.553	-30.130	17.330	1.00	0.00	
ATOM	2392	CD	LYS	A	147	4.602	-31.594	18.447	1.00	0.00	C
ATOM	2393	HD1	LYS	A	147	5.476	-31.916	18.810	1.00	0.00	
ATOM	2394	HD2	LYS	A	147	3.915	-31.605	19.173	1.00	0.00	
ATOM	2395	CE	LYS	A	147	4.155	-32.546	17.339	1.00	0.00	C
ATOM	2396	HE1	LYS	A	147	3.274	-32.251	16.970	1.00	0.00	
ATOM	2397	HE2	LYS	A	147	4.835	-32.561	16.606	1.00	0.00	
ATOM	2398	NZ	LYS	A	147	4.008	-33.915	17.876	1.00	0.00	N
ATOM	2399	HZ1	LYS	A	147	3.715	-34.530	17.144	1.00	0.00	
ATOM	2400	HZ2	LYS	A	147	4.885	-34.226	18.242	1.00	0.00	
ATOM	2401	HZ3	LYS	A	147	3.324	-33.915	18.605	1.00	0.00	
ATOM	2402	C	LYS	A	147	4.416	-27.902	21.070	1.00	0.00	C
ATOM	2403	O	LYS	A	147	4.138	-26.711	20.950	1.00	0.00	O
ATOM	2404	N	SER	A	148	5.190	-28.340	22.077	1.00	0.00	N
ATOM	2405	HN	SER	A	148	5.408	-29.308	22.201	1.00	0.00	
ATOM	2406	CA	SER	A	148	5.681	-27.328	22.960	1.00	0.00	C
ATOM	2407	HA	SER	A	148	5.056	-26.568	22.782	1.00	0.00	
ATOM	2408	CB	SER	A	148	5.679	-27.697	24.446	1.00	0.00	C
ATOM	2409	HB1	SER	A	148	4.746	-27.762	24.801	1.00	0.00	

```
ATOM   2410  HB2 SER A 148       6.157 -28.561  24.605  1.00  0.00
ATOM   2411  OG  SER A 148       6.358 -26.675  25.162  1.00  0.00           O
ATOM   2412  HG1 SER A 148       6.366 -26.900  26.136  1.00  0.00
ATOM   2413  C   SER A 148       7.100 -27.072  22.621  1.00  0.00           C
ATOM   2414  O   SER A 148       7.848 -27.971  22.250  1.00  0.00           O
ATOM   2415  N   SER A 149       7.505 -25.803  22.734  1.00  0.00           N
ATOM   2416  HN  SER A 149       6.858 -25.074  22.957  1.00  0.00
ATOM   2417  CA  SER A 149       8.884 -25.522  22.527  1.00  0.00           C
ATOM   2418  HA  SER A 149       9.315 -26.388  22.273  1.00  0.00
ATOM   2419  CB  SER A 149       9.192 -24.494  21.430  1.00  0.00           C
ATOM   2420  HB1 SER A 149      10.139 -24.573  21.120  1.00  0.00
ATOM   2421  HB2 SER A 149       8.576 -24.606  20.650  1.00  0.00
ATOM   2422  OG  SER A 149       9.012 -23.185  21.939  1.00  0.00           O
ATOM   2423  HG1 SER A 149       9.213 -22.518  21.222  1.00  0.00
ATOM   2424  C   SER A 149       9.274 -24.899  23.819  1.00  0.00           C
ATOM   2425  O   SER A 149       8.403 -24.555  24.616  1.00  0.00           O
ATOM   2426  N   VAL A 150      10.590 -24.729  24.053  1.00  0.00           N
ATOM   2427  HN  VAL A 150      11.242 -24.995  23.343  1.00  0.00
ATOM   2428  CA  VAL A 150      11.097 -24.179  25.285  1.00  0.00           C
ATOM   2429  HA  VAL A 150      10.817 -24.831  25.990  1.00  0.00
ATOM   2430  CB  VAL A 150      12.601 -24.036  25.273  1.00  0.00           C
ATOM   2431  HB  VAL A 150      12.883 -23.444  24.518  1.00  0.00
ATOM   2432  CG1 VAL A 150      13.069 -23.364  26.576  1.00  0.00           C
ATOM   2433 1HG1 VAL A 150      14.065 -23.271  26.564  1.00  0.00
ATOM   2434 2HG1 VAL A 150      12.651 -22.459  26.653  1.00  0.00
ATOM   2435 3HG1 VAL A 150      12.796 -23.925  27.357  1.00  0.00
ATOM   2436  CG2 VAL A 150      13.228 -25.423  25.051  1.00  0.00           C
ATOM   2437 1HG2 VAL A 150      14.225 -25.341  25.041  1.00  0.00
ATOM   2438 2HG2 VAL A 150      12.951 -26.036  25.791  1.00  0.00
ATOM   2439 3HG2 VAL A 150      12.916 -25.794  24.176  1.00  0.00
ATOM   2440  C   VAL A 150      10.512 -22.811  25.491  1.00  0.00           C
ATOM   2441  O   VAL A 150      10.029 -22.478  26.570  1.00  0.00           O
ATOM   2442  N   SER A 151      10.482 -21.989  24.441  1.00  0.00           N
ATOM   2443  HN  SER A 151      10.810 -22.296  23.547  1.00  0.00
ATOM   2444  CA  SER A 151       9.975 -20.663  24.611  1.00  0.00           C
ATOM   2445  HA  SER A 151       9.503 -20.606  25.491  1.00  0.00
ATOM   2446  CB  SER A 151      11.086 -19.603  24.566  1.00  0.00           C
ATOM   2447  HB1 SER A 151      11.769 -19.776  25.276  1.00  0.00
ATOM   2448  HB2 SER A 151      11.531 -19.595  23.670  1.00  0.00
ATOM   2449  OG  SER A 151      10.556 -18.303  24.792  1.00  0.00           O
ATOM   2450  HG1 SER A 151      11.296 -17.631  24.758  1.00  0.00
ATOM   2451  C   SER A 151       9.109 -20.488  23.424  1.00  0.00           C
ATOM   2452  O   SER A 151       8.117 -21.201  23.281  1.00  0.00           O
ATOM   2453  N   GLY A 152       9.452 -19.520  22.552  1.00  0.00           N
ATOM   2454  HN  GLY A 152      10.183 -18.879  22.786  1.00  0.00
ATOM   2455  CA  GLY A 152       8.765 -19.398  21.268  1.00  0.00           C
ATOM   2456  HA1 GLY A 152       9.468 -19.274  20.568  1.00  0.00
ATOM   2457  HA2 GLY A 152       8.284 -20.260  21.108  1.00  0.00
ATOM   2458  C   GLY A 152       7.766 -18.239  21.194  1.00  0.00           C
ATOM   2459  O   GLY A 152       7.207 -17.984  20.121  1.00  0.00           O
ATOM   2460  N   ILE A 153       7.514 -17.559  22.324  1.00  0.00           N
ATOM   2461  HN  ILE A 153       8.041 -17.780  23.145  1.00  0.00
ATOM   2462  CA  ILE A 153       6.508 -16.516  22.402  1.00  0.00           C
ATOM   2463  HA  ILE A 153       5.795 -16.773  21.750  1.00  0.00
ATOM   2464  CB  ILE A 153       5.875 -16.442  23.800  1.00  0.00           C
ATOM   2465  HB  ILE A 153       6.609 -16.413  24.479  1.00  0.00
ATOM   2466  CG2 ILE A 153       5.064 -15.167  23.955  1.00  0.00           C
ATOM   2467 1HG2 ILE A 153       4.661 -15.137  24.870  1.00  0.00
ATOM   2468 2HG2 ILE A 153       5.661 -14.375  23.828  1.00  0.00
ATOM   2469 3HG2 ILE A 153       4.336 -15.149  23.269  1.00  0.00
ATOM   2470  CG1 ILE A 153       5.032 -17.683  24.099  1.00  0.00           C
ATOM   2471 1HG1 ILE A 153       4.167 -17.599  23.604  1.00  0.00
ATOM   2472 2HG1 ILE A 153       5.529 -18.484  23.767  1.00  0.00
ATOM   2473  CD  ILE A 153       4.715 -17.897  25.583  1.00  0.00           C
ATOM   2474  HD1 ILE A 153       4.164 -18.725  25.690  1.00  0.00
ATOM   2475  HD2 ILE A 153       5.568 -17.996  26.095  1.00  0.00
ATOM   2476  HD3 ILE A 153       4.206 -17.110  25.932  1.00  0.00
ATOM   2477  C   ILE A 153       7.148 -15.182  22.009  1.00  0.00           C
ATOM   2478  O   ILE A 153       8.228 -14.847  22.481  1.00  0.00           O
ATOM   2479  N   ARG A 154       6.438 -14.431  21.156  1.00  0.00           N
ATOM   2480  HN  ARG A 154       5.615 -14.825  20.747  1.00  0.00
```

```
ATOM   2481  CA  ARG A 154       6.801 -13.088  20.800  1.00  0.00           C
ATOM   2482  HA  ARG A 154       7.674 -12.899  21.250  1.00  0.00
ATOM   2483  CB  ARG A 154       7.017 -12.977  19.293  1.00  0.00           C
ATOM   2484  HB1 ARG A 154       7.649 -13.697  19.007  1.00  0.00
ATOM   2485  HB2 ARG A 154       6.138 -13.104  18.834  1.00  0.00
ATOM   2486  CG  ARG A 154       7.593 -11.632  18.871  1.00  0.00           C
ATOM   2487  HG1 ARG A 154       6.910 -10.923  19.047  1.00  0.00
ATOM   2488  HG2 ARG A 154       8.411 -11.450  19.417  1.00  0.00
ATOM   2489  CD  ARG A 154       7.976 -11.566  17.413  1.00  0.00           C
ATOM   2490  HD1 ARG A 154       7.477 -12.273  16.912  1.00  0.00
ATOM   2491  HD2 ARG A 154       7.732 -10.666  17.051  1.00  0.00
ATOM   2492  NE  ARG A 154       9.399 -11.770  17.164  1.00  0.00           N
ATOM   2493  HE  ARG A 154      10.040 -11.599  17.913  1.00  0.00
ATOM   2494  CZ  ARG A 154       9.889 -12.170  15.998  1.00  0.00           C
ATOM   2495  NH1 ARG A 154       9.152 -12.031  14.900  1.00  0.00           N
ATOM   2496 1HH1 ARG A 154       8.238 -11.628  14.959  1.00  0.00
ATOM   2497 2HH1 ARG A 154       9.511 -12.329  14.016  1.00  0.00
ATOM   2498  NH2 ARG A 154      11.088 -12.735  15.950  1.00  0.00           N
ATOM   2499 1HH2 ARG A 154      11.617 -12.857  16.790  1.00  0.00
ATOM   2500 2HH2 ARG A 154      11.463 -13.039  15.074  1.00  0.00
ATOM   2501  C   ARG A 154       5.727 -12.120  21.322  1.00  0.00           C
ATOM   2502  O   ARG A 154       4.531 -12.383  21.222  1.00  0.00           O
ATOM   2503  N   ILE A 155       6.198 -11.011  21.902  1.00  0.00           N
ATOM   2504  HN  ILE A 155       7.179 -10.834  21.827  1.00  0.00
ATOM   2505  CA  ILE A 155       5.395 -10.039  22.636  1.00  0.00           C
ATOM   2506  HA  ILE A 155       4.549 -10.492  22.917  1.00  0.00
ATOM   2507  CB  ILE A 155       6.183  -9.557  23.865  1.00  0.00           C
ATOM   2508  HB  ILE A 155       7.102  -9.304  23.562  1.00  0.00
ATOM   2509  CG2 ILE A 155       5.582  -8.297  24.468  1.00  0.00           C
ATOM   2510 1HG2 ILE A 155       6.122  -8.017  25.262  1.00  0.00
ATOM   2511 2HG2 ILE A 155       5.588  -7.565  23.786  1.00  0.00
ATOM   2512 3HG2 ILE A 155       4.641  -8.480  24.752  1.00  0.00
ATOM   2513  CG1 ILE A 155       6.334 -10.676  24.899  1.00  0.00           C
ATOM   2514 1HG1 ILE A 155       6.723 -11.477  24.444  1.00  0.00
ATOM   2515 2HG1 ILE A 155       6.958 -10.363  25.615  1.00  0.00
ATOM   2516  CD  ILE A 155       5.032 -11.096  25.560  1.00  0.00           C
ATOM   2517  HD1 ILE A 155       5.213 -11.827  26.218  1.00  0.00
ATOM   2518  HD2 ILE A 155       4.631 -10.312  26.034  1.00  0.00
ATOM   2519  HD3 ILE A 155       4.395 -11.426  24.863  1.00  0.00
ATOM   2520  C   ILE A 155       5.083  -8.865  21.717  1.00  0.00           C
ATOM   2521  O   ILE A 155       5.960  -8.414  20.947  1.00  0.00           O
ATOM   2522  N   LYS A 156       3.857  -8.338  21.819  1.00  0.00           N
ATOM   2523  HN  LYS A 156       3.206  -8.721  22.474  1.00  0.00
ATOM   2524  CA  LYS A 156       3.461  -7.204  20.979  1.00  0.00           C
ATOM   2525  HA  LYS A 156       4.314  -6.786  20.665  1.00  0.00
ATOM   2526  CB  LYS A 156       2.644  -7.674  19.770  1.00  0.00           C
ATOM   2527  HB1 LYS A 156       1.693  -7.771  20.064  1.00  0.00
ATOM   2528  HB2 LYS A 156       2.705  -6.966  19.067  1.00  0.00
ATOM   2529  CG  LYS A 156       3.076  -9.001  19.136  1.00  0.00           C
ATOM   2530  HG1 LYS A 156       3.640  -9.515  19.783  1.00  0.00
ATOM   2531  HG2 LYS A 156       2.267  -9.539  18.898  1.00  0.00
ATOM   2532  CD  LYS A 156       3.862  -8.808  17.911  1.00  0.00           C
ATOM   2533  HD1 LYS A 156       3.343  -8.199  17.312  1.00  0.00
ATOM   2534  HD2 LYS A 156       4.722  -8.368  18.170  1.00  0.00
ATOM   2535  CE  LYS A 156       4.174 -10.077  17.160  1.00  0.00           C
ATOM   2536  HE1 LYS A 156       4.733 -10.673  17.737  1.00  0.00
ATOM   2537  HE2 LYS A 156       3.319 -10.542  16.930  1.00  0.00
ATOM   2538  NZ  LYS A 156       4.921  -9.784  15.899  1.00  0.00           N
ATOM   2539  HZ1 LYS A 156       5.116 -10.640  15.420  1.00  0.00
ATOM   2540  HZ2 LYS A 156       4.367  -9.193  15.313  1.00  0.00
ATOM   2541  HZ3 LYS A 156       5.780  -9.323  16.120  1.00  0.00
ATOM   2542  C   LYS A 156       2.637  -6.210  21.806  1.00  0.00           C
ATOM   2543  O   LYS A 156       1.529  -6.549  22.248  1.00  0.00           O
ATOM   2544  N   ILE A 157       3.164  -4.980  21.931  1.00  0.00           N
ATOM   2545  HN  ILE A 157       4.080  -4.820  21.564  1.00  0.00
ATOM   2546  CA  ILE A 157       2.472  -3.859  22.575  1.00  0.00           C
ATOM   2547  HA  ILE A 157       2.129  -4.199  23.451  1.00  0.00
ATOM   2548  CB  ILE A 157       3.448  -2.682  22.816  1.00  0.00           C
ATOM   2549  HB  ILE A 157       3.814  -2.403  21.928  1.00  0.00
ATOM   2550  CG2 ILE A 157       2.734  -1.476  23.398  1.00  0.00           C
ATOM   2551 1HG2 ILE A 157       3.392  -0.736  23.541  1.00  0.00
```

```
ATOM   2552 2HG2 ILE A 157       2.023  -1.172  22.764  1.00  0.00
ATOM   2553 3HG2 ILE A 157       2.317  -1.725  24.272  1.00  0.00
ATOM   2554  CG1 ILE A 157       4.645  -3.087  23.671  1.00  0.00           C
ATOM   2555 1HG1 ILE A 157       5.320  -3.540  23.089  1.00  0.00
ATOM   2556 2HG1 ILE A 157       5.049  -2.265  24.073  1.00  0.00
ATOM   2557  CD  ILE A 157       4.286  -4.023  24.785  1.00  0.00           C
ATOM   2558  HD1 ILE A 157       5.108  -4.251  25.307  1.00  0.00
ATOM   2559  HD2 ILE A 157       3.620  -3.584  25.388  1.00  0.00
ATOM   2560  HD3 ILE A 157       3.891  -4.859  24.404  1.00  0.00
ATOM   2561  C   ILE A 157       1.311  -3.412  21.683  1.00  0.00           C
ATOM   2562  O   ILE A 157       1.486  -3.217  20.495  1.00  0.00           O
ATOM   2563  N   ALA A 158       0.162  -3.144  22.306  1.00  0.00           N
ATOM   2564  HN  ALA A 158       0.138  -3.218  23.303  1.00  0.00
ATOM   2565  CA  ALA A 158      -1.053  -2.751  21.620  1.00  0.00           C
ATOM   2566  HA  ALA A 158      -0.799  -2.426  20.709  1.00  0.00
ATOM   2567  CB  ALA A 158      -1.936  -3.955  21.488  1.00  0.00           C
ATOM   2568  HB1 ALA A 158      -2.780  -3.699  21.016  1.00  0.00
ATOM   2569  HB2 ALA A 158      -1.461  -4.661  20.963  1.00  0.00
ATOM   2570  HB3 ALA A 158      -2.157  -4.308  22.397  1.00  0.00
ATOM   2571  C   ALA A 158      -1.754  -1.626  22.389  1.00  0.00           C
ATOM   2572  O   ALA A 158      -1.359  -1.324  23.489  1.00  0.00           O
ATOM   2573  N   ASP A 159      -2.770  -1.020  21.760  1.00  0.00           N
ATOM   2574  HN  ASP A 159      -2.914  -1.257  20.799  1.00  0.00
ATOM   2575  CA  ASP A 159      -3.702  -0.029  22.355  1.00  0.00           C
ATOM   2576  HA  ASP A 159      -4.414   0.095  21.664  1.00  0.00
ATOM   2577  CB  ASP A 159      -4.369  -0.528  23.638  1.00  0.00           C
ATOM   2578  HB1 ASP A 159      -4.684  -1.465  23.487  1.00  0.00
ATOM   2579  HB2 ASP A 159      -3.690  -0.519  24.372  1.00  0.00
ATOM   2580  CG  ASP A 159      -5.565   0.322  24.074  1.00  0.00           C
ATOM   2581  OD1 ASP A 159      -5.744   1.441  23.481  1.00  0.00           O
ATOM   2582  OD2 ASP A 159      -6.330  -0.136  24.983  1.00  0.00           O
ATOM   2583  C   ASP A 159      -2.984   1.290  22.622  1.00  0.00           C
ATOM   2584  O   ASP A 159      -2.600   1.562  23.738  1.00  0.00           O
ATOM   2585  N   PHE A 160      -2.890   2.134  21.600  1.00  0.00           N
ATOM   2586  HN  PHE A 160      -3.252   1.855  20.710  1.00  0.00
ATOM   2587  CA  PHE A 160      -2.287   3.434  21.727  1.00  0.00           C
ATOM   2588  HA  PHE A 160      -1.725   3.433  22.554  1.00  0.00
ATOM   2589  CB  PHE A 160      -1.373   3.674  20.527  1.00  0.00           C
ATOM   2590  HB1 PHE A 160      -1.878   3.467  19.689  1.00  0.00
ATOM   2591  HB2 PHE A 160      -1.094   4.634  20.519  1.00  0.00
ATOM   2592  CG  PHE A 160      -0.131   2.820  20.555  1.00  0.00           C
ATOM   2593  CD1 PHE A 160      -0.156   1.512  20.133  1.00  0.00           C
ATOM   2594  HD1 PHE A 160      -1.007   1.122  19.782  1.00  0.00
ATOM   2595  CE1 PHE A 160       0.983   0.729  20.189  1.00  0.00           C
ATOM   2596  HE1 PHE A 160       0.945  -0.225  19.892  1.00  0.00
ATOM   2597  CZ  PHE A 160       2.164   1.253  20.649  1.00  0.00           C
ATOM   2598  HZ  PHE A 160       2.991   0.692  20.667  1.00  0.00
ATOM   2599  CD2 PHE A 160       1.049   3.311  21.072  1.00  0.00           C
ATOM   2600  HD2 PHE A 160       1.073   4.238  21.447  1.00  0.00
ATOM   2601  CE2 PHE A 160       2.201   2.543  21.081  1.00  0.00           C
ATOM   2602  HE2 PHE A 160       3.061   2.937  21.405  1.00  0.00
ATOM   2603  C   PHE A 160      -3.374   4.495  21.911  1.00  0.00           C
ATOM   2604  O   PHE A 160      -3.138   5.670  21.620  1.00  0.00           O
ATOM   2605  N   GLY A 161      -4.527   4.082  22.460  1.00  0.00           N
ATOM   2606  HN  GLY A 161      -4.605   3.124  22.734  1.00  0.00
ATOM   2607  CA  GLY A 161      -5.666   4.953  22.676  1.00  0.00           C
ATOM   2608  HA1 GLY A 161      -5.947   5.302  21.782  1.00  0.00
ATOM   2609  HA2 GLY A 161      -6.400   4.393  23.061  1.00  0.00
ATOM   2610  C   GLY A 161      -5.357   6.125  23.619  1.00  0.00           C
ATOM   2611  O   GLY A 161      -5.911   7.212  23.455  1.00  0.00           O
ATOM   2612  N   PHE A 162      -4.474   5.910  24.597  1.00  0.00           N
ATOM   2613  HN  PHE A 162      -3.999   5.030  24.631  1.00  0.00
ATOM   2614  CA  PHE A 162      -4.176   6.902  25.612  1.00  0.00           C
ATOM   2615  HA  PHE A 162      -4.896   7.594  25.550  1.00  0.00
ATOM   2616  CB  PHE A 162      -4.212   6.243  27.000  1.00  0.00           C
ATOM   2617  HB1 PHE A 162      -4.828   5.456  26.961  1.00  0.00
ATOM   2618  HB2 PHE A 162      -3.289   5.933  27.227  1.00  0.00
ATOM   2619  CG  PHE A 162      -4.682   7.134  28.122  1.00  0.00           C
ATOM   2620  CD1 PHE A 162      -6.001   7.572  28.175  1.00  0.00           C
ATOM   2621  HD1 PHE A 162      -6.649   7.276  27.473  1.00  0.00
ATOM   2622  CE1 PHE A 162      -6.428   8.417  29.193  1.00  0.00           C
```

ATOM	2623	HE1	PHE	A	162	-7.376	8.735	29.211	1.00	0.00	
ATOM	2624	CZ	PHE	A	162	-5.549	8.820	30.181	1.00	0.00	C
ATOM	2625	HZ	PHE	A	162	-5.861	9.420	30.918	1.00	0.00	
ATOM	2626	CD2	PHE	A	162	-3.811	7.536	29.133	1.00	0.00	C
ATOM	2627	HD2	PHE	A	162	-2.867	7.207	29.132	1.00	0.00	
ATOM	2628	CE2	PHE	A	162	-4.241	8.391	30.145	1.00	0.00	C
ATOM	2629	HE2	PHE	A	162	-3.598	8.694	30.848	1.00	0.00	
ATOM	2630	C	PHE	A	162	-2.814	7.565	25.327	1.00	0.00	C
ATOM	2631	O	PHE	A	162	-2.358	8.384	26.087	1.00	0.00	O
ATOM	2632	N	ALA	A	163	-2.181	7.206	24.205	1.00	0.00	N
ATOM	2633	HN	ALA	A	163	-2.680	6.662	23.531	1.00	0.00	
ATOM	2634	CA	ALA	A	163	-0.789	7.572	23.915	1.00	0.00	C
ATOM	2635	HA	ALA	A	163	-0.240	7.384	24.729	1.00	0.00	
ATOM	2636	CB	ALA	A	163	-0.276	6.721	22.789	1.00	0.00	C
ATOM	2637	HB1	ALA	A	163	0.672	6.968	22.589	1.00	0.00	
ATOM	2638	HB2	ALA	A	163	-0.321	5.757	23.052	1.00	0.00	
ATOM	2639	HB3	ALA	A	163	-0.838	6.871	21.975	1.00	0.00	
ATOM	2640	C	ALA	A	163	-0.700	9.059	23.580	1.00	0.00	C
ATOM	2641	O	ALA	A	163	-1.696	9.690	23.210	1.00	0.00	O
ATOM	2642	N	ARG	A	164	0.499	9.631	23.718	1.00	0.00	N
ATOM	2643	HN	ARG	A	164	1.262	9.098	24.084	1.00	0.00	
ATOM	2644	CA	ARG	A	164	0.706	11.004	23.347	1.00	0.00	C
ATOM	2645	HA	ARG	A	164	0.145	11.181	22.538	1.00	0.00	
ATOM	2646	CB	ARG	A	164	0.214	11.918	24.472	1.00	0.00	C
ATOM	2647	HB1	ARG	A	164	-0.251	12.697	24.052	1.00	0.00	
ATOM	2648	HB2	ARG	A	164	-0.438	11.399	25.025	1.00	0.00	
ATOM	2649	CG	ARG	A	164	1.273	12.470	25.413	1.00	0.00	C
ATOM	2650	HG1	ARG	A	164	1.665	11.717	25.942	1.00	0.00	
ATOM	2651	HG2	ARG	A	164	1.993	12.909	24.874	1.00	0.00	
ATOM	2652	CD	ARG	A	164	0.645	13.499	26.368	1.00	0.00	C
ATOM	2653	HD1	ARG	A	164	0.253	14.256	25.844	1.00	0.00	
ATOM	2654	HD2	ARG	A	164	-0.072	13.065	26.914	1.00	0.00	
ATOM	2655	NE	ARG	A	164	1.630	14.059	27.291	1.00	0.00	N
ATOM	2656	HE	ARG	A	164	1.611	13.744	28.240	1.00	0.00	
ATOM	2657	CZ	ARG	A	164	2.550	14.958	26.956	1.00	0.00	C
ATOM	2658	NH1	ARG	A	164	2.395	15.689	25.874	1.00	0.00	N
ATOM	2659	1HH1	ARG	A	164	1.584	15.569	25.302	1.00	0.00	
ATOM	2660	2HH1	ARG	A	164	3.088	16.365	25.624	1.00	0.00	
ATOM	2661	NH2	ARG	A	164	3.677	15.044	27.635	1.00	0.00	N
ATOM	2662	1HH2	ARG	A	164	3.844	14.431	28.407	1.00	0.00	
ATOM	2663	2HH2	ARG	A	164	4.365	15.723	27.378	1.00	0.00	
ATOM	2664	C	ARG	A	164	2.168	11.223	22.950	1.00	0.00	C
ATOM	2665	O	ARG	A	164	3.024	10.396	23.225	1.00	0.00	O
ATOM	2666	N	TYR	A	165	2.389	12.293	22.178	1.00	0.00	N
ATOM	2667	HN	TYR	A	165	1.599	12.834	21.888	1.00	0.00	
ATOM	2668	CA	TYR	A	165	3.691	12.713	21.740	1.00	0.00	C
ATOM	2669	HA	TYR	A	165	4.227	11.870	21.689	1.00	0.00	
ATOM	2670	CB	TYR	A	165	3.615	13.394	20.372	1.00	0.00	C
ATOM	2671	HB1	TYR	A	165	2.875	14.067	20.397	1.00	0.00	
ATOM	2672	HB2	TYR	A	165	4.484	13.860	20.205	1.00	0.00	
ATOM	2673	CG	TYR	A	165	3.359	12.491	19.201	1.00	0.00	C
ATOM	2674	CD1	TYR	A	165	4.394	11.808	18.595	1.00	0.00	C
ATOM	2675	HD1	TYR	A	165	5.322	11.906	18.954	1.00	0.00	
ATOM	2676	CE1	TYR	A	165	4.174	10.990	17.500	1.00	0.00	C
ATOM	2677	HE1	TYR	A	165	4.939	10.504	17.077	1.00	0.00	
ATOM	2678	CZ	TYR	A	165	2.900	10.846	16.993	1.00	0.00	C
ATOM	2679	OH	TYR	A	165	2.685	10.040	15.915	1.00	0.00	O
ATOM	2680	HH	TYR	A	165	1.713	10.050	15.680	1.00	0.00	
ATOM	2681	CD2	TYR	A	165	2.093	12.371	18.651	1.00	0.00	C
ATOM	2682	HD2	TYR	A	165	1.337	12.894	19.044	1.00	0.00	
ATOM	2683	CE2	TYR	A	165	1.853	11.540	17.566	1.00	0.00	C
ATOM	2684	HE2	TYR	A	165	0.927	11.444	17.201	1.00	0.00	
ATOM	2685	C	TYR	A	165	4.264	13.717	22.747	1.00	0.00	C
ATOM	2686	O	TYR	A	165	3.546	14.631	23.151	1.00	0.00	O
ATOM	2687	N	LEU	A	166	5.558	13.568	23.066	1.00	0.00	N
ATOM	2688	HN	LEU	A	166	6.028	12.741	22.758	1.00	0.00	
ATOM	2689	CA	LEU	A	166	6.315	14.542	23.835	1.00	0.00	C
ATOM	2690	HA	LEU	A	166	5.736	14.790	24.611	1.00	0.00	
ATOM	2691	CB	LEU	A	166	7.633	13.913	24.289	1.00	0.00	C
ATOM	2692	HB1	LEU	A	166	7.985	13.352	23.540	1.00	0.00	
ATOM	2693	HB2	LEU	A	166	8.279	14.650	24.486	1.00	0.00	

```
ATOM   2694  CG  LEU A 166       7.543  13.035  25.522  1.00  0.00           C
ATOM   2695  HG  LEU A 166       6.941  12.263  25.318  1.00  0.00
ATOM   2696  CD1 LEU A 166       8.909  12.459  25.883  1.00  0.00           C
ATOM   2697 1HD1 LEU A 166       8.824  11.885  26.697  1.00  0.00
ATOM   2698 2HD1 LEU A 166       9.250  11.908  25.121  1.00  0.00
ATOM   2699 3HD1 LEU A 166       9.548  13.206  26.067  1.00  0.00
ATOM   2700  CD2 LEU A 166       6.938  13.824  26.683  1.00  0.00           C
ATOM   2701 1HD2 LEU A 166       6.882  13.238  27.491  1.00  0.00
ATOM   2702 2HD2 LEU A 166       7.515  14.616  26.884  1.00  0.00
ATOM   2703 3HD2 LEU A 166       6.021  14.135  26.433  1.00  0.00
ATOM   2704  C   LEU A 166       6.626  15.771  22.981  1.00  0.00           C
ATOM   2705  O   LEU A 166       7.301  15.647  21.974  1.00  0.00           O
ATOM   2706  N   HIS A 167       6.246  16.948  23.487  1.00  0.00           N
ATOM   2707  HN  HIS A 167       5.810  16.957  24.387  1.00  0.00
ATOM   2708  CA  HIS A 167       6.431  18.213  22.802  1.00  0.00           C
ATOM   2709  HA  HIS A 167       6.363  18.076  21.814  1.00  0.00
ATOM   2710  CB  HIS A 167       5.287  19.175  23.190  1.00  0.00           C
ATOM   2711  HB1 HIS A 167       5.320  19.346  24.175  1.00  0.00
ATOM   2712  HB2 HIS A 167       5.402  20.038  22.698  1.00  0.00
ATOM   2713  ND1 HIS A 167       3.590  18.191  21.588  1.00  0.00           N
ATOM   2714  CG  HIS A 167       3.928  18.640  22.864  1.00  0.00           C
ATOM   2715  CE1 HIS A 167       2.343  17.765  21.583  1.00  0.00           C
ATOM   2716  HE1 HIS A 167       1.851  17.394  20.795  1.00  0.00
ATOM   2717  NE2 HIS A 167       1.851  17.917  22.821  1.00  0.00           N
ATOM   2718  HE2 HIS A 167       0.926  17.674  23.112  1.00  0.00
ATOM   2719  CD2 HIS A 167       2.823  18.461  23.625  1.00  0.00           C
ATOM   2720  HD2 HIS A 167       2.731  18.684  24.595  1.00  0.00
ATOM   2721  C   HIS A 167       7.854  18.705  23.095  1.00  0.00           C
ATOM   2722  O   HIS A 167       8.045  19.552  23.938  1.00  0.00           O
ATOM   2723  N   SER A 168       8.851  18.235  22.330  1.00  0.00           N
ATOM   2724  HN  SER A 168       8.655  17.568  21.611  1.00  0.00
ATOM   2725  CA  SER A 168      10.191  18.683  22.538  1.00  0.00           C
ATOM   2726  HA  SER A 168      10.140  19.553  23.028  1.00  0.00
ATOM   2727  CB  SER A 168      11.044  17.698  23.355  1.00  0.00           C
ATOM   2728  HB1 SER A 168      11.963  18.064  23.504  1.00  0.00
ATOM   2729  HB2 SER A 168      10.615  17.506  24.238  1.00  0.00
ATOM   2730  OG  SER A 168      11.185  16.461  22.673  1.00  0.00           O
ATOM   2731  HG1 SER A 168      11.742  15.839  23.223  1.00  0.00
ATOM   2732  C   SER A 168      10.756  18.773  21.172  1.00  0.00           C
ATOM   2733  O   SER A 168      10.035  19.099  20.229  1.00  0.00           O
ATOM   2734  N   ASN A 169      12.074  18.513  21.028  1.00  0.00           N
ATOM   2735  HN  ASN A 169      12.645  18.373  21.837  1.00  0.00
ATOM   2736  CA  ASN A 169      12.657  18.437  19.719  1.00  0.00           C
ATOM   2737  HA  ASN A 169      12.550  19.330  19.282  1.00  0.00
ATOM   2738  CB  ASN A 169      14.154  18.082  19.764  1.00  0.00           C
ATOM   2739  HB1 ASN A 169      14.508  17.951  18.838  1.00  0.00
ATOM   2740  HB2 ASN A 169      14.670  18.808  20.218  1.00  0.00
ATOM   2741  CG  ASN A 169      14.276  16.785  20.555  1.00  0.00           C
ATOM   2742  OD1 ASN A 169      14.083  16.764  21.772  1.00  0.00           O
ATOM   2743  ND2 ASN A 169      14.532  15.662  19.834  1.00  0.00           N
ATOM   2744 1HD2 ASN A 169      14.629  15.717  18.840  1.00  0.00
ATOM   2745 2HD2 ASN A 169      14.623  14.780  20.297  1.00  0.00
ATOM   2746  C   ASN A 169      11.910  17.326  19.045  1.00  0.00           C
ATOM   2747  O   ASN A 169      11.781  16.225  19.586  1.00  0.00           O
ATOM   2748  N   MET A 170      11.333  17.611  17.865  1.00  0.00           N
ATOM   2749  HN  MET A 170      11.481  18.480  17.394  1.00  0.00
ATOM   2750  CA  MET A 170      10.495  16.587  17.332  1.00  0.00           C
ATOM   2751  HA  MET A 170      10.944  15.747  17.637  1.00  0.00
ATOM   2752  CB  MET A 170       9.047  16.660  17.849  1.00  0.00           C
ATOM   2753  HB1 MET A 170       9.049  16.959  18.803  1.00  0.00
ATOM   2754  HB2 MET A 170       8.529  17.313  17.296  1.00  0.00
ATOM   2755  CG  MET A 170       8.375  15.299  17.761  1.00  0.00           C
ATOM   2756  HG1 MET A 170       8.352  15.123  16.777  1.00  0.00
ATOM   2757  HG2 MET A 170       9.041  14.691  18.193  1.00  0.00
ATOM   2758  SD  MET A 170       6.727  15.127  18.511  1.00  0.00           S
ATOM   2759  CE  MET A 170       7.091  13.405  18.965  1.00  0.00           C
ATOM   2760  HE1 MET A 170       6.301  13.010  19.434  1.00  0.00
ATOM   2761  HE2 MET A 170       7.286  12.877  18.139  1.00  0.00
ATOM   2762  HE3 MET A 170       7.886  13.381  19.572  1.00  0.00
ATOM   2763  C   MET A 170      10.460  16.698  15.843  1.00  0.00           C
ATOM   2764  O   MET A 170      11.378  17.241  15.233  1.00  0.00           O
```

ATOM	2765	N	MET	A	171	9.362	16.189	15.244	1.00	0.00	N
ATOM	2766	HN	MET	A	171	8.615	15.879	15.832	1.00	0.00	
ATOM	2767	CA	MET	A	171	9.189	16.061	13.823	1.00	0.00	C
ATOM	2768	HA	MET	A	171	9.910	15.430	13.535	1.00	0.00	
ATOM	2769	CB	MET	A	171	7.787	15.517	13.479	1.00	0.00	C
ATOM	2770	HB1	MET	A	171	7.106	16.191	13.764	1.00	0.00	
ATOM	2771	HB2	MET	A	171	7.731	15.385	12.489	1.00	0.00	
ATOM	2772	CG	MET	A	171	7.465	14.179	14.167	1.00	0.00	C
ATOM	2773	HG1	MET	A	171	8.197	13.567	13.870	1.00	0.00	
ATOM	2774	HG2	MET	A	171	7.568	14.373	15.143	1.00	0.00	
ATOM	2775	SD	MET	A	171	5.833	13.498	13.791	1.00	0.00	S
ATOM	2776	CE	MET	A	171	5.816	12.390	15.224	1.00	0.00	C
ATOM	2777	HE1	MET	A	171	4.960	11.873	15.235	1.00	0.00	
ATOM	2778	HE2	MET	A	171	6.587	11.756	15.165	1.00	0.00	
ATOM	2779	HE3	MET	A	171	5.891	12.929	16.063	1.00	0.00	
ATOM	2780	C	MET	A	171	9.358	17.405	13.169	1.00	0.00	C
ATOM	2781	O	MET	A	171	10.069	17.521	12.172	1.00	0.00	O
ATOM	2782	N	ALA	A	172	8.741	18.460	13.736	1.00	0.00	N
ATOM	2783	HN	ALA	A	172	8.155	18.314	14.533	1.00	0.00	
ATOM	2784	CA	ALA	A	172	8.909	19.792	13.218	1.00	0.00	C
ATOM	2785	HA	ALA	A	172	9.872	19.844	12.953	1.00	0.00	
ATOM	2786	CB	ALA	A	172	8.011	20.113	12.008	1.00	0.00	C
ATOM	2787	HB1	ALA	A	172	8.182	21.050	11.705	1.00	0.00	
ATOM	2788	HB2	ALA	A	172	8.217	19.479	11.263	1.00	0.00	
ATOM	2789	HB3	ALA	A	172	7.051	20.018	12.271	1.00	0.00	
ATOM	2790	C	ALA	A	172	8.505	20.723	14.315	1.00	0.00	C
ATOM	2791	O	ALA	A	172	7.658	20.377	15.136	1.00	0.00	O
ATOM	2792	N	ALA	A	173	9.118	21.925	14.356	1.00	0.00	N
ATOM	2793	HN	ALA	A	173	9.832	22.135	13.688	1.00	0.00	
ATOM	2794	CA	ALA	A	173	8.765	22.908	15.341	1.00	0.00	C
ATOM	2795	HA	ALA	A	173	7.767	22.856	15.377	1.00	0.00	
ATOM	2796	CB	ALA	A	173	9.371	22.665	16.735	1.00	0.00	C
ATOM	2797	HB1	ALA	A	173	9.077	23.391	17.357	1.00	0.00	
ATOM	2798	HB2	ALA	A	173	9.061	21.781	17.084	1.00	0.00	
ATOM	2799	HB3	ALA	A	173	10.369	22.666	16.669	1.00	0.00	
ATOM	2800	C	ALA	A	173	9.309	24.212	14.878	1.00	0.00	C
ATOM	2801	O	ALA	A	173	10.210	24.266	14.043	1.00	0.00	O
ATOM	2802	N	ASP	A	174	8.743	25.309	15.410	1.00	0.00	N
ATOM	2803	HN	ASP	A	174	7.973	25.215	16.041	1.00	0.00	
ATOM	2804	CA	ASP	A	174	9.235	26.608	15.080	1.00	0.00	C
ATOM	2805	HA	ASP	A	174	9.780	26.480	14.252	1.00	0.00	
ATOM	2806	CB	ASP	A	174	8.132	27.648	14.810	1.00	0.00	C
ATOM	2807	HB1	ASP	A	174	7.471	27.649	15.560	1.00	0.00	
ATOM	2808	HB2	ASP	A	174	8.534	28.559	14.719	1.00	0.00	
ATOM	2809	CG	ASP	A	174	7.432	27.267	13.513	1.00	0.00	C
ATOM	2810	OD1	ASP	A	174	7.757	26.183	12.959	1.00	0.00	O
ATOM	2811	OD2	ASP	A	174	6.564	28.058	13.057	1.00	0.00	O
ATOM	2812	C	ASP	A	174	10.002	27.061	16.278	1.00	0.00	C
ATOM	2813	O	ASP	A	174	9.772	26.589	17.394	1.00	0.00	O
ATOM	2814	N	LEU	A	175	10.956	27.999	16.089	1.00	0.00	N
ATOM	2815	HN	LEU	A	175	11.126	28.369	15.176	1.00	0.00	
ATOM	2816	CA	LEU	A	175	11.727	28.462	17.217	1.00	0.00	C
ATOM	2817	HA	LEU	A	175	12.135	27.630	17.593	1.00	0.00	
ATOM	2818	CB	LEU	A	175	12.838	29.470	16.851	1.00	0.00	C
ATOM	2819	HB1	LEU	A	175	12.420	30.218	16.335	1.00	0.00	
ATOM	2820	HB2	LEU	A	175	13.224	29.827	17.701	1.00	0.00	
ATOM	2821	CG	LEU	A	175	13.994	28.893	16.006	1.00	0.00	C
ATOM	2822	HG	LEU	A	175	13.618	28.594	15.129	1.00	0.00	
ATOM	2823	CD1	LEU	A	175	15.047	29.967	15.692	1.00	0.00	C
ATOM	2824	1HD1	LEU	A	175	15.781	29.564	15.145	1.00	0.00	
ATOM	2825	2HD1	LEU	A	175	14.620	30.712	15.180	1.00	0.00	
ATOM	2826	3HD1	LEU	A	175	15.425	30.323	16.547	1.00	0.00	
ATOM	2827	CD2	LEU	A	175	14.608	27.649	16.667	1.00	0.00	C
ATOM	2828	1HD2	LEU	A	175	15.352	27.301	16.096	1.00	0.00	
ATOM	2829	2HD2	LEU	A	175	14.966	27.893	17.568	1.00	0.00	
ATOM	2830	3HD2	LEU	A	175	13.906	26.944	16.767	1.00	0.00	
ATOM	2831	C	LEU	A	175	10.784	29.148	18.154	1.00	0.00	C
ATOM	2832	O	LEU	A	175	10.241	30.209	17.847	1.00	0.00	O
ATOM	2833	N	CYS	A	176	10.538	28.513	19.314	1.00	0.00	N
ATOM	2834	HN	CYS	A	176	10.996	27.651	19.529	1.00	0.00	
ATOM	2835	CA	CYS	A	176	9.610	29.092	20.235	1.00	0.00	C

ATOM	2836	HA	CYS	A	176	9.530	30.049	19.957	1.00	0.00	
ATOM	2837	CB	CYS	A	176	8.237	28.390	20.197	1.00	0.00	C
ATOM	2838	HB1	CYS	A	176	7.982	28.394	19.230	1.00	0.00	
ATOM	2839	HB2	CYS	A	176	8.428	27.452	20.487	1.00	0.00	
ATOM	2840	SG	CYS	A	176	6.966	29.177	21.238	1.00	0.00	S
ATOM	2841	HG1	CYS	A	176	6.109	28.667	21.161	1.00	0.00	
ATOM	2842	C	CYS	A	176	10.174	28.951	21.607	1.00	0.00	C
ATOM	2843	O	CYS	A	176	10.813	27.953	21.942	1.00	0.00	O
ATOM	2844	N	GLY	A	177	9.943	29.984	22.435	1.00	0.00	N
ATOM	2845	HN	GLY	A	177	9.485	30.793	22.068	1.00	0.00	
ATOM	2846	CA	GLY	A	177	10.321	29.985	23.821	1.00	0.00	C
ATOM	2847	HA1	GLY	A	177	11.031	29.300	23.982	1.00	0.00	
ATOM	2848	HA2	GLY	A	177	10.664	30.888	24.081	1.00	0.00	
ATOM	2849	C	GLY	A	177	9.062	29.648	24.551	1.00	0.00	C
ATOM	2850	O	GLY	A	177	8.315	28.758	24.143	1.00	0.00	O
ATOM	2851	N	SER	A	178	8.779	30.347	25.667	1.00	0.00	N
ATOM	2852	HN	SER	A	178	9.424	31.025	26.018	1.00	0.00	
ATOM	2853	CA	SER	A	178	7.532	30.103	26.347	1.00	0.00	C
ATOM	2854	HA	SER	A	178	7.308	29.160	26.102	1.00	0.00	
ATOM	2855	CB	SER	A	178	7.601	30.257	27.874	1.00	0.00	C
ATOM	2856	HB1	SER	A	178	8.080	29.484	28.289	1.00	0.00	
ATOM	2857	HB2	SER	A	178	8.061	31.108	28.126	1.00	0.00	
ATOM	2858	OG	SER	A	178	6.287	30.296	28.418	1.00	0.00	O
ATOM	2859	HG1	SER	A	178	6.340	30.396	29.412	1.00	0.00	
ATOM	2860	C	SER	A	178	6.555	31.130	25.870	1.00	0.00	C
ATOM	2861	O	SER	A	178	6.912	32.294	25.706	1.00	0.00	O
ATOM	2862	N	PRO	A	179	5.353	30.704	25.556	1.00	0.00	N
ATOM	2863	CD	PRO	A	179	5.156	29.396	24.957	1.00	0.00	C
ATOM	2864	HD1	PRO	A	179	5.328	28.684	25.638	1.00	0.00	
ATOM	2865	HD2	PRO	A	179	5.783	29.279	24.187	1.00	0.00	
ATOM	2866	CA	PRO	A	179	4.337	31.634	25.163	1.00	0.00	C
ATOM	2867	HA	PRO	A	179	4.743	32.410	24.680	1.00	0.00	
ATOM	2868	CB	PRO	A	179	3.396	30.879	24.224	1.00	0.00	C
ATOM	2869	HB1	PRO	A	179	2.442	31.094	24.433	1.00	0.00	
ATOM	2870	HB2	PRO	A	179	3.589	31.110	23.270	1.00	0.00	
ATOM	2871	CG	PRO	A	179	3.696	29.398	24.499	1.00	0.00	C
ATOM	2872	HG1	PRO	A	179	3.099	29.040	25.217	1.00	0.00	
ATOM	2873	HG2	PRO	A	179	3.584	28.851	23.669	1.00	0.00	
ATOM	2874	C	PRO	A	179	3.701	32.247	26.377	1.00	0.00	C
ATOM	2875	O	PRO	A	179	3.287	31.523	27.285	1.00	0.00	O
ATOM	2876	N	MET	A	180	3.533	33.585	26.348	1.00	0.00	N
ATOM	2877	HN	MET	A	180	3.752	34.045	25.488	1.00	0.00	
ATOM	2878	CA	MET	A	180	3.061	34.439	27.449	1.00	0.00	C
ATOM	2879	HA	MET	A	180	3.661	34.230	28.221	1.00	0.00	
ATOM	2880	CB	MET	A	180	3.152	35.920	27.052	1.00	0.00	C
ATOM	2881	HB1	MET	A	180	2.531	36.078	26.284	1.00	0.00	
ATOM	2882	HB2	MET	A	180	2.863	36.473	27.833	1.00	0.00	
ATOM	2883	CG	MET	A	180	4.539	36.389	26.636	1.00	0.00	C
ATOM	2884	HG1	MET	A	180	4.869	36.937	27.405	1.00	0.00	
ATOM	2885	HG2	MET	A	180	5.084	35.554	26.564	1.00	0.00	
ATOM	2886	SD	MET	A	180	4.487	37.331	25.074	1.00	0.00	S
ATOM	2887	CE	MET	A	180	4.341	35.975	23.906	1.00	0.00	C
ATOM	2888	HE1	MET	A	180	4.300	36.339	22.976	1.00	0.00	
ATOM	2889	HE2	MET	A	180	5.135	35.373	23.993	1.00	0.00	
ATOM	2890	HE3	MET	A	180	3.507	35.458	24.099	1.00	0.00	
ATOM	2891	C	MET	A	180	1.592	34.128	27.780	1.00	0.00	C
ATOM	2892	O	MET	A	180	1.142	34.341	28.913	1.00	0.00	O
ATOM	2893	N	TYR	A	181	0.868	33.610	26.782	1.00	0.00	N
ATOM	2894	HN	TYR	A	181	1.351	33.321	25.955	1.00	0.00	
ATOM	2895	CA	TYR	A	181	-0.546	33.443	26.820	1.00	0.00	C
ATOM	2896	HA	TYR	A	181	-0.881	34.156	27.436	1.00	0.00	
ATOM	2897	CB	TYR	A	181	-1.093	33.619	25.416	1.00	0.00	C
ATOM	2898	HB1	TYR	A	181	-0.729	32.888	24.838	1.00	0.00	
ATOM	2899	HB2	TYR	A	181	-2.090	33.551	25.452	1.00	0.00	
ATOM	2900	CG	TYR	A	181	-0.736	34.936	24.786	1.00	0.00	C
ATOM	2901	CD1	TYR	A	181	-1.446	36.075	25.114	1.00	0.00	C
ATOM	2902	HD1	TYR	A	181	-2.166	36.023	25.806	1.00	0.00	
ATOM	2903	CE1	TYR	A	181	-1.177	37.287	24.501	1.00	0.00	C
ATOM	2904	HE1	TYR	A	181	-1.724	38.094	24.723	1.00	0.00	
ATOM	2905	CZ	TYR	A	181	-0.147	37.377	23.579	1.00	0.00	C
ATOM	2906	OH	TYR	A	181	0.119	38.582	22.999	1.00	0.00	O

```
ATOM   2907  HH  TYR A 181       0.884  38.484  22.362  1.00  0.00
ATOM   2908  CD2 TYR A 181       0.280  35.041  23.842  1.00  0.00           C
ATOM   2909  HD2 TYR A 181       0.796  34.224  23.584  1.00  0.00
ATOM   2910  CE2 TYR A 181       0.590  36.251  23.255  1.00  0.00           C
ATOM   2911  HE2 TYR A 181       1.343  36.314  22.600  1.00  0.00
ATOM   2912  C   TYR A 181      -0.922  32.056  27.360  1.00  0.00           C
ATOM   2913  O   TYR A 181      -2.084  31.792  27.646  1.00  0.00           O
ATOM   2914  N   MET A 182       0.072  31.183  27.513  1.00  0.00           N
ATOM   2915  HN  MET A 182       1.014  31.486  27.369  1.00  0.00
ATOM   2916  CA  MET A 182      -0.171  29.844  27.872  1.00  0.00           C
ATOM   2917  HA  MET A 182      -1.070  29.639  27.484  1.00  0.00
ATOM   2918  CB  MET A 182       0.900  28.943  27.269  1.00  0.00           C
ATOM   2919  HB1 MET A 182       1.125  29.281  26.355  1.00  0.00
ATOM   2920  HB2 MET A 182       1.714  28.982  27.849  1.00  0.00
ATOM   2921  CG  MET A 182       0.458  27.493  27.152  1.00  0.00           C
ATOM   2922  HG1 MET A 182       0.396  27.157  28.092  1.00  0.00
ATOM   2923  HG2 MET A 182      -0.456  27.525  26.748  1.00  0.00
ATOM   2924  SD  MET A 182       1.612  26.540  26.159  1.00  0.00           S
ATOM   2925  CE  MET A 182       1.362  27.253  24.529  1.00  0.00           C
ATOM   2926  HE1 MET A 182       1.961  26.798  23.870  1.00  0.00
ATOM   2927  HE2 MET A 182       0.409  27.129  24.254  1.00  0.00
ATOM   2928  HE3 MET A 182       1.577  28.229  24.556  1.00  0.00
ATOM   2929  C   MET A 182      -0.172  29.706  29.401  1.00  0.00           C
ATOM   2930  O   MET A 182       0.637  30.330  30.114  1.00  0.00           O
ATOM   2931  N   ALA A 183      -1.098  28.886  29.903  1.00  0.00           N
ATOM   2932  HN  ALA A 183      -1.660  28.356  29.268  1.00  0.00
ATOM   2933  CA  ALA A 183      -1.326  28.728  31.325  1.00  0.00           C
ATOM   2934  HA  ALA A 183      -1.525  29.634  31.698  1.00  0.00
ATOM   2935  CB  ALA A 183      -2.523  27.844  31.561  1.00  0.00           C
ATOM   2936  HB1 ALA A 183      -2.672  27.741  32.544  1.00  0.00
ATOM   2937  HB2 ALA A 183      -3.331  28.259  31.143  1.00  0.00
ATOM   2938  HB3 ALA A 183      -2.360  26.946  31.153  1.00  0.00
ATOM   2939  C   ALA A 183      -0.073  28.159  31.978  1.00  0.00           C
ATOM   2940  O   ALA A 183       0.548  27.247  31.441  1.00  0.00           O
ATOM   2941  N   PRO A 184       0.333  28.676  33.154  1.00  0.00           N
ATOM   2942  CD  PRO A 184      -0.342  29.750  33.893  1.00  0.00           C
ATOM   2943  HD1 PRO A 184      -1.136  29.393  34.385  1.00  0.00
ATOM   2944  HD2 PRO A 184      -0.634  30.476  33.270  1.00  0.00
ATOM   2945  CA  PRO A 184       1.551  28.219  33.810  1.00  0.00           C
ATOM   2946  HA  PRO A 184       2.327  28.395  33.204  1.00  0.00
ATOM   2947  CB  PRO A 184       1.562  29.008  35.125  1.00  0.00           C
ATOM   2948  HB1 PRO A 184       1.159  28.472  35.867  1.00  0.00
ATOM   2949  HB2 PRO A 184       2.493  29.274  35.374  1.00  0.00
ATOM   2950  CG  PRO A 184       0.721  30.233  34.841  1.00  0.00           C
ATOM   2951  HG1 PRO A 184       0.309  30.584  35.682  1.00  0.00
ATOM   2952  HG2 PRO A 184       1.272  30.951  34.415  1.00  0.00
ATOM   2953  C   PRO A 184       1.567  26.703  34.082  1.00  0.00           C
ATOM   2954  O   PRO A 184       2.624  26.075  33.983  1.00  0.00           O
ATOM   2955  N   GLU A 185       0.411  26.126  34.432  1.00  0.00           N
ATOM   2956  HN  GLU A 185      -0.416  26.682  34.511  1.00  0.00
ATOM   2957  CA  GLU A 185       0.347  24.691  34.699  1.00  0.00           C
ATOM   2958  HA  GLU A 185       0.997  24.499  35.434  1.00  0.00
ATOM   2959  CB  GLU A 185      -1.032  24.287  35.228  1.00  0.00           C
ATOM   2960  HB1 GLU A 185      -1.009  23.311  35.447  1.00  0.00
ATOM   2961  HB2 GLU A 185      -1.213  24.811  36.060  1.00  0.00
ATOM   2962  CG  GLU A 185      -2.188  24.521  34.272  1.00  0.00           C
ATOM   2963  HG1 GLU A 185      -1.808  24.664  33.358  1.00  0.00
ATOM   2964  HG2 GLU A 185      -2.758  23.700  34.271  1.00  0.00
ATOM   2965  CD  GLU A 185      -3.077  25.705  34.597  1.00  0.00           C
ATOM   2966  OE1 GLU A 185      -2.517  26.767  34.974  1.00  0.00           O
ATOM   2967  OE2 GLU A 185      -4.338  25.578  34.448  1.00  0.00           O
ATOM   2968  C   GLU A 185       0.771  23.923  33.430  1.00  0.00           C
ATOM   2969  O   GLU A 185       1.441  22.922  33.510  1.00  0.00           O
ATOM   2970  N   VAL A 186       0.434  24.461  32.253  1.00  0.00           N
ATOM   2971  HN  VAL A 186      -0.041  25.341  32.248  1.00  0.00
ATOM   2972  CA  VAL A 186       0.729  23.819  30.975  1.00  0.00           C
ATOM   2973  HA  VAL A 186       0.496  22.855  31.104  1.00  0.00
ATOM   2974  CB  VAL A 186      -0.141  24.380  29.837  1.00  0.00           C
ATOM   2975  HB  VAL A 186       0.010  25.368  29.797  1.00  0.00
ATOM   2976  CG1 VAL A 186       0.269  23.783  28.492  1.00  0.00           C
ATOM   2977 1HG1 VAL A 186      -0.309  24.162  27.769  1.00  0.00
```

```
ATOM   2978 2HG1 VAL A 186       1.226  24.007  28.306  1.00  0.00
ATOM   2979 3HG1 VAL A 186       0.159  22.789  28.521  1.00  0.00
ATOM   2980  CG2 VAL A 186      -1.630  24.175  30.099  1.00  0.00           C
ATOM   2981 1HG2 VAL A 186      -2.157  24.553  29.338  1.00  0.00
ATOM   2982 2HG2 VAL A 186      -1.822  23.197  30.185  1.00  0.00
ATOM   2983 3HG2 VAL A 186      -1.887  24.641  30.946  1.00  0.00
ATOM   2984  C   VAL A 186       2.221  23.966  30.655  1.00  0.00           C
ATOM   2985  O   VAL A 186       2.854  23.026  30.285  1.00  0.00           O
ATOM   2986  N   ILE A 187       2.756  25.170  30.784  1.00  0.00           N
ATOM   2987  HN  ILE A 187       2.160  25.942  31.005  1.00  0.00
ATOM   2988  CA  ILE A 187       4.202  25.413  30.613  1.00  0.00           C
ATOM   2989  HA  ILE A 187       4.397  25.256  29.645  1.00  0.00
ATOM   2990  CB  ILE A 187       4.577  26.863  30.980  1.00  0.00           C
ATOM   2991  HB  ILE A 187       4.336  27.009  31.939  1.00  0.00
ATOM   2992  CG2 ILE A 187       6.073  27.073  30.840  1.00  0.00           C
ATOM   2993 1HG2 ILE A 187       6.301  28.016  31.081  1.00  0.00
ATOM   2994 2HG2 ILE A 187       6.556  26.447  31.452  1.00  0.00
ATOM   2995 3HG2 ILE A 187       6.348  26.894  29.895  1.00  0.00
ATOM   2996  CG1 ILE A 187       3.785  27.903  30.177  1.00  0.00           C
ATOM   2997 1HG1 ILE A 187       2.812  27.679  30.237  1.00  0.00
ATOM   2998 2HG1 ILE A 187       3.945  28.805  30.578  1.00  0.00
ATOM   2999  CD  ILE A 187       4.155  27.973  28.715  1.00  0.00           C
ATOM   3000  HD1 ILE A 187       3.598  28.670  28.263  1.00  0.00
ATOM   3001  HD2 ILE A 187       5.122  28.211  28.627  1.00  0.00
ATOM   3002  HD3 ILE A 187       3.990  27.085  28.286  1.00  0.00
ATOM   3003  C   ILE A 187       4.988  24.435  31.489  1.00  0.00           C
ATOM   3004  O   ILE A 187       5.882  23.764  31.009  1.00  0.00           O
ATOM   3005  N   MET A 188       4.641  24.397  32.780  1.00  0.00           N
ATOM   3006  HN  MET A 188       3.803  24.870  33.054  1.00  0.00
ATOM   3007  CA  MET A 188       5.407  23.709  33.816  1.00  0.00           C
ATOM   3008  HA  MET A 188       6.354  23.974  33.635  1.00  0.00
ATOM   3009  CB  MET A 188       4.971  24.156  35.221  1.00  0.00           C
ATOM   3010  HB1 MET A 188       3.972  24.127  35.267  1.00  0.00
ATOM   3011  HB2 MET A 188       5.354  23.518  35.889  1.00  0.00
ATOM   3012  CG  MET A 188       5.423  25.564  35.597  1.00  0.00           C
ATOM   3013  HG1 MET A 188       6.401  25.556  35.389  1.00  0.00
ATOM   3014  HG2 MET A 188       4.947  26.144  34.936  1.00  0.00
ATOM   3015  SD  MET A 188       5.074  26.060  37.361  1.00  0.00           S
ATOM   3016  CE  MET A 188       3.283  26.014  37.425  1.00  0.00           C
ATOM   3017  HE1 MET A 188       2.976  26.267  38.342  1.00  0.00
ATOM   3018  HE2 MET A 188       2.909  26.659  36.758  1.00  0.00
ATOM   3019  HE3 MET A 188       2.965  25.090  37.210  1.00  0.00
ATOM   3020  C   MET A 188       5.228  22.187  33.711  1.00  0.00           C
ATOM   3021  O   MET A 188       6.038  21.455  34.224  1.00  0.00           O
ATOM   3022  N   SER A 189       4.146  21.720  33.078  1.00  0.00           N
ATOM   3023  HN  SER A 189       3.550  22.362  32.595  1.00  0.00
ATOM   3024  CA  SER A 189       3.811  20.294  33.073  1.00  0.00           C
ATOM   3025  HA  SER A 189       4.194  19.936  33.925  1.00  0.00
ATOM   3026  CB  SER A 189       2.328  20.088  33.089  1.00  0.00           C
ATOM   3027  HB1 SER A 189       2.111  19.143  33.334  1.00  0.00
ATOM   3028  HB2 SER A 189       1.898  20.710  33.744  1.00  0.00
ATOM   3029  OG  SER A 189       1.780  20.349  31.805  1.00  0.00           O
ATOM   3030  HG1 SER A 189       0.790  20.208  31.830  1.00  0.00
ATOM   3031  C   SER A 189       4.424  19.575  31.861  1.00  0.00           C
ATOM   3032  O   SER A 189       4.175  18.390  31.661  1.00  0.00           O
ATOM   3033  N   GLN A 190       5.179  20.288  31.030  1.00  0.00           N
ATOM   3034  HN  GLN A 190       5.303  21.267  31.194  1.00  0.00
ATOM   3035  CA  GLN A 190       5.828  19.664  29.884  1.00  0.00           C
ATOM   3036  HA  GLN A 190       5.113  19.287  29.296  1.00  0.00
ATOM   3037  CB  GLN A 190       6.594  20.717  29.080  1.00  0.00           C
ATOM   3038  HB1 GLN A 190       6.191  21.614  29.263  1.00  0.00
ATOM   3039  HB2 GLN A 190       7.549  20.714  29.377  1.00  0.00
ATOM   3040  CG  GLN A 190       6.557  20.470  27.577  1.00  0.00           C
ATOM   3041  HG1 GLN A 190       7.170  19.712  27.355  1.00  0.00
ATOM   3042  HG2 GLN A 190       5.624  20.234  27.307  1.00  0.00
ATOM   3043  CD  GLN A 190       6.990  21.682  26.797  1.00  0.00           C
ATOM   3044  OE1 GLN A 190       6.180  22.358  26.168  1.00  0.00           O
ATOM   3045  NE2 GLN A 190       8.281  21.973  26.853  1.00  0.00           N
ATOM   3046 1HE2 GLN A 190       8.900  21.400  27.390  1.00  0.00
ATOM   3047 2HE2 GLN A 190       8.637  22.766  26.358  1.00  0.00
ATOM   3048  C   GLN A 190       6.736  18.528  30.385  1.00  0.00           C
```

ATOM	3049	O	GLN	A	190	7.449	18.712	31.351	1.00	0.00	O
ATOM	3050	N	HIS	A	191	6.647	17.348	29.752	1.00	0.00	N
ATOM	3051	HN	HIS	A	191	5.951	17.249	29.041	1.00	0.00	
ATOM	3052	CA	HIS	A	191	7.517	16.180	30.042	1.00	0.00	C
ATOM	3053	HA	HIS	A	191	7.317	15.529	29.310	1.00	0.00	
ATOM	3054	CB	HIS	A	191	9.001	16.590	30.002	1.00	0.00	C
ATOM	3055	HB1	HIS	A	191	9.119	17.434	30.525	1.00	0.00	
ATOM	3056	HB2	HIS	A	191	9.551	15.863	30.414	1.00	0.00	
ATOM	3057	ND1	HIS	A	191	10.889	16.792	28.350	1.00	0.00	N
ATOM	3058	CG	HIS	A	191	9.533	16.833	28.630	1.00	0.00	C
ATOM	3059	CE1	HIS	A	191	11.082	17.037	27.068	1.00	0.00	C
ATOM	3060	HE1	HIS	A	191	11.967	17.082	26.605	1.00	0.00	
ATOM	3061	NE2	HIS	A	191	9.886	17.214	26.495	1.00	0.00	N
ATOM	3062	HE2	HIS	A	191	9.728	17.405	25.526	1.00	0.00	
ATOM	3063	CD2	HIS	A	191	8.910	17.091	27.455	1.00	0.00	C
ATOM	3064	HD2	HIS	A	191	7.924	17.176	27.313	1.00	0.00	
ATOM	3065	C	HIS	A	191	7.181	15.509	31.391	1.00	0.00	C
ATOM	3066	O	HIS	A	191	7.926	14.637	31.837	1.00	0.00	O
ATOM	3067	N	TYR	A	192	6.064	15.876	32.029	1.00	0.00	N
ATOM	3068	HN	TYR	A	192	5.438	16.516	31.584	1.00	0.00	
ATOM	3069	CA	TYR	A	192	5.728	15.360	33.379	1.00	0.00	C
ATOM	3070	HA	TYR	A	192	6.515	15.582	33.955	1.00	0.00	
ATOM	3071	CB	TYR	A	192	4.498	16.058	33.981	1.00	0.00	C
ATOM	3072	HB1	TYR	A	192	4.546	15.984	34.977	1.00	0.00	
ATOM	3073	HB2	TYR	A	192	4.519	17.022	33.717	1.00	0.00	
ATOM	3074	CG	TYR	A	192	3.171	15.496	33.546	1.00	0.00	C
ATOM	3075	CD1	TYR	A	192	2.621	15.856	32.335	1.00	0.00	C
ATOM	3076	HD1	TYR	A	192	3.102	16.518	31.760	1.00	0.00	
ATOM	3077	CE1	TYR	A	192	1.422	15.327	31.893	1.00	0.00	C
ATOM	3078	HE1	TYR	A	192	1.052	15.598	31.004	1.00	0.00	
ATOM	3079	CZ	TYR	A	192	0.738	14.425	32.681	1.00	0.00	C
ATOM	3080	OH	TYR	A	192	-0.444	13.941	32.200	1.00	0.00	O
ATOM	3081	HH	TYR	A	192	-0.836	13.302	32.861	1.00	0.00	
ATOM	3082	CD2	TYR	A	192	2.486	14.575	34.317	1.00	0.00	C
ATOM	3083	HD2	TYR	A	192	2.870	14.289	35.195	1.00	0.00	
ATOM	3084	CE2	TYR	A	192	1.279	14.040	33.899	1.00	0.00	C
ATOM	3085	HE2	TYR	A	192	0.799	13.378	34.474	1.00	0.00	
ATOM	3086	C	TYR	A	192	5.525	13.837	33.324	1.00	0.00	C
ATOM	3087	O	TYR	A	192	5.868	13.123	34.277	1.00	0.00	O
ATOM	3088	N	ASP	A	193	4.980	13.347	32.201	1.00	0.00	N
ATOM	3089	HN	ASP	A	193	4.814	13.971	31.437	1.00	0.00	
ATOM	3090	CA	ASP	A	193	4.619	11.945	32.046	1.00	0.00	C
ATOM	3091	HA	ASP	A	193	4.654	11.554	32.966	1.00	0.00	
ATOM	3092	CB	ASP	A	193	3.187	11.800	31.516	1.00	0.00	C
ATOM	3093	HB1	ASP	A	193	2.988	10.825	31.421	1.00	0.00	
ATOM	3094	HB2	ASP	A	193	2.565	12.203	32.187	1.00	0.00	
ATOM	3095	CG	ASP	A	193	2.912	12.462	30.173	1.00	0.00	C
ATOM	3096	OD1	ASP	A	193	3.809	13.191	29.658	1.00	0.00	O
ATOM	3097	OD2	ASP	A	193	1.790	12.258	29.651	1.00	0.00	O
ATOM	3098	C	ASP	A	193	5.642	11.226	31.155	1.00	0.00	C
ATOM	3099	O	ASP	A	193	5.375	10.139	30.671	1.00	0.00	O
ATOM	3100	N	ALA	A	194	6.835	11.805	31.006	1.00	0.00	N
ATOM	3101	HN	ALA	A	194	7.036	12.625	31.541	1.00	0.00	
ATOM	3102	CA	ALA	A	194	7.869	11.293	30.091	1.00	0.00	C
ATOM	3103	HA	ALA	A	194	7.446	11.254	29.186	1.00	0.00	
ATOM	3104	CB	ALA	A	194	9.041	12.243	30.041	1.00	0.00	C
ATOM	3105	HB1	ALA	A	194	9.735	11.884	29.417	1.00	0.00	
ATOM	3106	HB2	ALA	A	194	8.732	13.137	29.717	1.00	0.00	
ATOM	3107	HB3	ALA	A	194	9.433	12.338	30.956	1.00	0.00	
ATOM	3108	C	ALA	A	194	8.327	9.886	30.502	1.00	0.00	C
ATOM	3109	O	ALA	A	194	8.771	9.119	29.642	1.00	0.00	O
ATOM	3110	N	LYS	A	195	8.203	9.544	31.792	1.00	0.00	N
ATOM	3111	HN	LYS	A	195	7.761	10.182	32.423	1.00	0.00	
ATOM	3112	CA	LYS	A	195	8.695	8.266	32.303	1.00	0.00	C
ATOM	3113	HA	LYS	A	195	9.205	7.857	31.546	1.00	0.00	
ATOM	3114	CB	LYS	A	195	9.613	8.505	33.499	1.00	0.00	C
ATOM	3115	HB1	LYS	A	195	9.213	9.224	34.068	1.00	0.00	
ATOM	3116	HB2	LYS	A	195	9.674	7.658	34.027	1.00	0.00	
ATOM	3117	CG	LYS	A	195	11.019	8.929	33.119	1.00	0.00	C
ATOM	3118	HG1	LYS	A	195	11.499	8.144	32.728	1.00	0.00	
ATOM	3119	HG2	LYS	A	195	10.963	9.659	32.438	1.00	0.00	

```
ATOM   3120  CD  LYS A 195      11.807   9.434  34.302  1.00  0.00           C
ATOM   3121  HD1 LYS A 195      11.450  10.332  34.560  1.00  0.00
ATOM   3122  HD2 LYS A 195      11.682   8.795  35.061  1.00  0.00
ATOM   3123  CE  LYS A 195      13.280   9.570  34.038  1.00  0.00           C
ATOM   3124  HE1 LYS A 195      13.655   8.686  33.760  1.00  0.00
ATOM   3125  HE2 LYS A 195      13.431  10.240  33.311  1.00  0.00
ATOM   3126  NZ  LYS A 195      13.975  10.023  35.261  1.00  0.00           N
ATOM   3127  HZ1 LYS A 195      14.954  10.110  35.075  1.00  0.00
ATOM   3128  HZ2 LYS A 195      13.609  10.910  35.543  1.00  0.00
ATOM   3129  HZ3 LYS A 195      13.833   9.355  35.992  1.00  0.00
ATOM   3130  C   LYS A 195       7.542   7.332  32.696  1.00  0.00           C
ATOM   3131  O   LYS A 195       7.779   6.323  33.349  1.00  0.00           O
ATOM   3132  N   ALA A 196       6.322   7.649  32.273  1.00  0.00           N
ATOM   3133  HN  ALA A 196       6.211   8.395  31.616  1.00  0.00
ATOM   3134  CA  ALA A 196       5.136   6.931  32.747  1.00  0.00           C
ATOM   3135  HA  ALA A 196       5.107   7.072  33.737  1.00  0.00
ATOM   3136  CB  ALA A 196       3.887   7.502  32.121  1.00  0.00           C
ATOM   3137  HB1 ALA A 196       3.088   7.001  32.455  1.00  0.00
ATOM   3138  HB2 ALA A 196       3.802   8.467  32.367  1.00  0.00
ATOM   3139  HB3 ALA A 196       3.943   7.415  31.126  1.00  0.00
ATOM   3140  C   ALA A 196       5.240   5.440  32.432  1.00  0.00           C
ATOM   3141  O   ALA A 196       4.888   4.601  33.255  1.00  0.00           O
ATOM   3142  N   ASP A 197       5.718   5.124  31.222  1.00  0.00           N
ATOM   3143  HN  ASP A 197       6.077   5.850  30.636  1.00  0.00
ATOM   3144  CA  ASP A 197       5.729   3.766  30.741  1.00  0.00           C
ATOM   3145  HA  ASP A 197       4.793   3.432  30.852  1.00  0.00
ATOM   3146  CB  ASP A 197       6.080   3.708  29.256  1.00  0.00           C
ATOM   3147  HB1 ASP A 197       6.927   4.218  29.106  1.00  0.00
ATOM   3148  HB2 ASP A 197       6.215   2.752  28.995  1.00  0.00
ATOM   3149  CG  ASP A 197       5.011   4.295  28.366  1.00  0.00           C
ATOM   3150  OD1 ASP A 197       3.831   4.313  28.797  1.00  0.00           O
ATOM   3151  OD2 ASP A 197       5.371   4.757  27.251  1.00  0.00           O
ATOM   3152  C   ASP A 197       6.685   2.928  31.591  1.00  0.00           C
ATOM   3153  O   ASP A 197       6.464   1.749  31.769  1.00  0.00           O
ATOM   3154  N   LEU A 198       7.734   3.554  32.127  1.00  0.00           N
ATOM   3155  HN  LEU A 198       7.855   4.532  31.959  1.00  0.00
ATOM   3156  CA  LEU A 198       8.708   2.851  32.951  1.00  0.00           C
ATOM   3157  HA  LEU A 198       9.009   2.047  32.438  1.00  0.00
ATOM   3158  CB  LEU A 198       9.914   3.755  33.197  1.00  0.00           C
ATOM   3159  HB1 LEU A 198       9.575   4.653  33.478  1.00  0.00
ATOM   3160  HB2 LEU A 198      10.454   3.356  33.938  1.00  0.00
ATOM   3161  CG  LEU A 198      10.833   3.949  31.988  1.00  0.00           C
ATOM   3162  HG  LEU A 198      10.250   4.230  31.226  1.00  0.00
ATOM   3163  CD1 LEU A 198      11.868   5.042  32.234  1.00  0.00           C
ATOM   3164 1HD1 LEU A 198      12.448   5.140  31.425  1.00  0.00
ATOM   3165 2HD1 LEU A 198      11.402   5.908  32.416  1.00  0.00
ATOM   3166 3HD1 LEU A 198      12.433   4.795  33.022  1.00  0.00
ATOM   3167  CD2 LEU A 198      11.544   2.652  31.614  1.00  0.00           C
ATOM   3168 1HD2 LEU A 198      12.135   2.812  30.823  1.00  0.00
ATOM   3169 2HD2 LEU A 198      12.098   2.341  32.386  1.00  0.00
ATOM   3170 3HD2 LEU A 198      10.865   1.953  31.387  1.00  0.00
ATOM   3171  C   LEU A 198       8.046   2.391  34.261  1.00  0.00           C
ATOM   3172  O   LEU A 198       8.300   1.290  34.728  1.00  0.00           O
ATOM   3173  N   TRP A 199       7.161   3.210  34.830  1.00  0.00           N
ATOM   3174  HN  TRP A 199       7.024   4.127  34.455  1.00  0.00
ATOM   3175  CA  TRP A 199       6.397   2.788  35.982  1.00  0.00           C
ATOM   3176  HA  TRP A 199       7.079   2.544  36.672  1.00  0.00
ATOM   3177  CB  TRP A 199       5.529   3.922  36.528  1.00  0.00           C
ATOM   3178  HB1 TRP A 199       6.122   4.632  36.908  1.00  0.00
ATOM   3179  HB2 TRP A 199       4.986   4.305  35.781  1.00  0.00
ATOM   3180  CG  TRP A 199       4.589   3.478  37.604  1.00  0.00           C
ATOM   3181  CD1 TRP A 199       3.355   2.931  37.431  1.00  0.00           C
ATOM   3182  HD1 TRP A 199       2.920   2.771  36.545  1.00  0.00
ATOM   3183  NE1 TRP A 199       2.794   2.631  38.645  1.00  0.00           N
ATOM   3184  HE1 TRP A 199       1.884   2.239  38.782  1.00  0.00
ATOM   3185  CE2 TRP A 199       3.679   2.954  39.641  1.00  0.00           C
ATOM   3186  CD2 TRP A 199       4.834   3.481  39.026  1.00  0.00           C
ATOM   3187  CE3 TRP A 199       5.909   3.885  39.827  1.00  0.00           C
ATOM   3188  HE3 TRP A 199       6.739   4.262  39.416  1.00  0.00
ATOM   3189  CZ3 TRP A 199       5.799   3.752  41.192  1.00  0.00           C
ATOM   3190  HZ3 TRP A 199       6.563   4.027  41.776  1.00  0.00
```

ATOM	3191	CZ2	TRP	A	199	3.543	2.863	41.023	1.00	0.00	C
ATOM	3192	HZ2	TRP	A	199	2.696	2.542	41.446	1.00	0.00	
ATOM	3193	CH2	TRP	A	199	4.630	3.236	41.780	1.00	0.00	C
ATOM	3194	HH2	TRP	A	199	4.588	3.138	42.774	1.00	0.00	
ATOM	3195	C	TRP	A	199	5.542	1.575	35.617	1.00	0.00	C
ATOM	3196	O	TRP	A	199	5.508	0.588	36.336	1.00	0.00	O
ATOM	3197	N	SER	A	200	4.832	1.676	34.499	1.00	0.00	N
ATOM	3198	HN	SER	A	200	4.956	2.481	33.919	1.00	0.00	
ATOM	3199	CA	SER	A	200	3.882	0.656	34.090	1.00	0.00	C
ATOM	3200	HA	SER	A	200	3.194	0.600	34.814	1.00	0.00	
ATOM	3201	CB	SER	A	200	3.180	1.055	32.833	1.00	0.00	C
ATOM	3202	HB1	SER	A	200	3.839	1.210	32.097	1.00	0.00	
ATOM	3203	HB2	SER	A	200	2.530	0.348	32.554	1.00	0.00	
ATOM	3204	OG	SER	A	200	2.465	2.251	33.042	1.00	0.00	O
ATOM	3205	HG1	SER	A	200	1.998	2.512	32.197	1.00	0.00	
ATOM	3206	C	SER	A	200	4.595	-0.685	33.927	1.00	0.00	C
ATOM	3207	O	SER	A	200	4.131	-1.698	34.429	1.00	0.00	O
ATOM	3208	N	ILE	A	201	5.721	-0.663	33.208	1.00	0.00	N
ATOM	3209	HN	ILE	A	201	6.031	0.212	32.837	1.00	0.00	
ATOM	3210	CA	ILE	A	201	6.512	-1.842	32.940	1.00	0.00	C
ATOM	3211	HA	ILE	A	201	5.936	-2.482	32.430	1.00	0.00	
ATOM	3212	CB	ILE	A	201	7.723	-1.480	32.050	1.00	0.00	C
ATOM	3213	HB	ILE	A	201	8.173	-0.673	32.434	1.00	0.00	
ATOM	3214	CG2	ILE	A	201	8.773	-2.588	32.046	1.00	0.00	C
ATOM	3215	1HG2	ILE	A	201	9.539	-2.319	31.462	1.00	0.00	
ATOM	3216	2HG2	ILE	A	201	9.102	-2.739	32.978	1.00	0.00	
ATOM	3217	3HG2	ILE	A	201	8.366	-3.432	31.695	1.00	0.00	
ATOM	3218	CG1	ILE	A	201	7.262	-1.126	30.633	1.00	0.00	C
ATOM	3219	1HG1	ILE	A	201	7.010	-1.974	30.166	1.00	0.00	
ATOM	3220	2HG1	ILE	A	201	6.460	-0.534	30.705	1.00	0.00	
ATOM	3221	CD	ILE	A	201	8.305	-0.408	29.779	1.00	0.00	C
ATOM	3222	HD1	ILE	A	201	7.922	-0.216	28.876	1.00	0.00	
ATOM	3223	HD2	ILE	A	201	8.563	0.451	30.221	1.00	0.00	
ATOM	3224	HD3	ILE	A	201	9.113	-0.989	29.682	1.00	0.00	
ATOM	3225	C	ILE	A	201	6.911	-2.473	34.287	1.00	0.00	C
ATOM	3226	O	ILE	A	201	6.906	-3.695	34.446	1.00	0.00	O
ATOM	3227	N	GLY	A	202	7.214	-1.611	35.262	1.00	0.00	N
ATOM	3228	HN	GLY	A	202	7.234	-0.635	35.045	1.00	0.00	
ATOM	3229	CA	GLY	A	202	7.515	-2.023	36.618	1.00	0.00	C
ATOM	3230	HA1	GLY	A	202	8.348	-2.576	36.590	1.00	0.00	
ATOM	3231	HA2	GLY	A	202	7.680	-1.197	37.157	1.00	0.00	
ATOM	3232	C	GLY	A	202	6.387	-2.826	37.242	1.00	0.00	C
ATOM	3233	O	GLY	A	202	6.630	-3.892	37.809	1.00	0.00	O
ATOM	3234	N	THR	A	203	5.161	-2.302	37.162	1.00	0.00	N
ATOM	3235	HN	THR	A	203	5.041	-1.439	36.672	1.00	0.00	
ATOM	3236	CA	THR	A	203	3.968	-2.945	37.769	1.00	0.00	C
ATOM	3237	HA	THR	A	203	4.180	-3.107	38.733	1.00	0.00	
ATOM	3238	CB	THR	A	203	2.746	-2.018	37.727	1.00	0.00	C
ATOM	3239	HB	THR	A	203	1.960	-2.482	38.136	1.00	0.00	
ATOM	3240	OG1	THR	A	203	2.416	-1.743	36.369	1.00	0.00	O
ATOM	3241	HG1	THR	A	203	1.620	-1.139	36.335	1.00	0.00	
ATOM	3242	CG2	THR	A	203	2.967	-0.709	38.463	1.00	0.00	C
ATOM	3243	1HG2	THR	A	203	2.141	-0.149	38.402	1.00	0.00	
ATOM	3244	2HG2	THR	A	203	3.172	-0.897	39.424	1.00	0.00	
ATOM	3245	3HG2	THR	A	203	3.734	-0.219	38.048	1.00	0.00	
ATOM	3246	C	THR	A	203	3.701	-4.289	37.071	1.00	0.00	C
ATOM	3247	O	THR	A	203	3.305	-5.258	37.694	1.00	0.00	O
ATOM	3248	N	VAL	A	204	3.941	-4.338	35.764	1.00	0.00	N
ATOM	3249	HN	VAL	A	204	4.325	-3.534	35.311	1.00	0.00	
ATOM	3250	CA	VAL	A	204	3.663	-5.522	34.978	1.00	0.00	C
ATOM	3251	HA	VAL	A	204	2.741	-5.815	35.231	1.00	0.00	
ATOM	3252	CB	VAL	A	204	3.664	-5.207	33.472	1.00	0.00	C
ATOM	3253	HB	VAL	A	204	4.469	-4.649	33.272	1.00	0.00	
ATOM	3254	CG1	VAL	A	204	3.778	-6.460	32.616	1.00	0.00	C
ATOM	3255	1HG1	VAL	A	204	3.774	-6.205	31.649	1.00	0.00	
ATOM	3256	2HG1	VAL	A	204	4.631	-6.935	32.832	1.00	0.00	
ATOM	3257	3HG1	VAL	A	204	3.003	-7.064	32.804	1.00	0.00	
ATOM	3258	CG2	VAL	A	204	2.422	-4.421	33.103	1.00	0.00	C
ATOM	3259	1HG2	VAL	A	204	2.433	-4.222	32.123	1.00	0.00	
ATOM	3260	2HG2	VAL	A	204	1.609	-4.959	33.325	1.00	0.00	
ATOM	3261	3HG2	VAL	A	204	2.405	-3.564	33.617	1.00	0.00	

```
ATOM   3262  C   VAL A 204       4.672  -6.612  35.345  1.00  0.00           C
ATOM   3263  O   VAL A 204       4.281  -7.745  35.577  1.00  0.00           O
ATOM   3264  N   ILE A 205       5.960  -6.249  35.391  1.00  0.00           N
ATOM   3265  HN  ILE A 205       6.210  -5.318  35.125  1.00  0.00
ATOM   3266  CA  ILE A 205       7.006  -7.163  35.814  1.00  0.00           C
ATOM   3267  HA  ILE A 205       6.991  -7.917  35.157  1.00  0.00
ATOM   3268  CB  ILE A 205       8.392  -6.507  35.775  1.00  0.00           C
ATOM   3269  HB  ILE A 205       8.341  -5.662  36.308  1.00  0.00
ATOM   3270  CG2 ILE A 205       9.433  -7.409  36.411  1.00  0.00           C
ATOM   3271 1HG2 ILE A 205      10.327  -6.963  36.375  1.00  0.00
ATOM   3272 2HG2 ILE A 205       9.186  -7.583  37.364  1.00  0.00
ATOM   3273 3HG2 ILE A 205       9.472  -8.275  35.913  1.00  0.00
ATOM   3274  CG1 ILE A 205       8.808  -6.118  34.363  1.00  0.00           C
ATOM   3275 1HG1 ILE A 205       9.146  -6.930  33.888  1.00  0.00
ATOM   3276 2HG1 ILE A 205       8.013  -5.755  33.878  1.00  0.00
ATOM   3277  CD  ILE A 205       9.891  -5.074  34.351  1.00  0.00           C
ATOM   3278  HD1 ILE A 205      10.130  -4.852  33.406  1.00  0.00
ATOM   3279  HD2 ILE A 205       9.565  -4.250  34.815  1.00  0.00
ATOM   3280  HD3 ILE A 205      10.698  -5.426  34.825  1.00  0.00
ATOM   3281  C   ILE A 205       6.688  -7.636  37.232  1.00  0.00           C
ATOM   3282  O   ILE A 205       6.820  -8.805  37.529  1.00  0.00           O
ATOM   3283  N   TYR A 206       6.311  -6.701  38.108  1.00  0.00           N
ATOM   3284  HN  TYR A 206       6.231  -5.751  37.806  1.00  0.00
ATOM   3285  CA  TYR A 206       6.016  -7.035  39.486  1.00  0.00           C
ATOM   3286  HA  TYR A 206       6.886  -7.366  39.851  1.00  0.00
ATOM   3287  CB  TYR A 206       5.570  -5.818  40.299  1.00  0.00           C
ATOM   3288  HB1 TYR A 206       6.265  -5.104  40.215  1.00  0.00
ATOM   3289  HB2 TYR A 206       4.703  -5.485  39.928  1.00  0.00
ATOM   3290  CG  TYR A 206       5.371  -6.105  41.766  1.00  0.00           C
ATOM   3291  CD1 TYR A 206       4.177  -6.626  42.256  1.00  0.00           C
ATOM   3292  HD1 TYR A 206       3.425  -6.802  41.621  1.00  0.00
ATOM   3293  CE1 TYR A 206       4.006  -6.906  43.602  1.00  0.00           C
ATOM   3294  HE1 TYR A 206       3.136  -7.271  43.934  1.00  0.00
ATOM   3295  CZ  TYR A 206       5.053  -6.675  44.488  1.00  0.00           C
ATOM   3296  OH  TYR A 206       4.968  -6.945  45.828  1.00  0.00           O
ATOM   3297  HH  TYR A 206       5.831  -6.704  46.272  1.00  0.00
ATOM   3298  CD2 TYR A 206       6.407  -5.915  42.658  1.00  0.00           C
ATOM   3299  HD2 TYR A 206       7.284  -5.570  42.323  1.00  0.00
ATOM   3300  CE2 TYR A 206       6.258  -6.192  44.006  1.00  0.00           C
ATOM   3301  HE2 TYR A 206       7.022  -6.043  44.633  1.00  0.00
ATOM   3302  C   TYR A 206       4.928  -8.115  39.532  1.00  0.00           C
ATOM   3303  O   TYR A 206       5.075  -9.099  40.238  1.00  0.00           O
ATOM   3304  N   GLN A 207       3.857  -7.927  38.754  1.00  0.00           N
ATOM   3305  HN  GLN A 207       3.811  -7.124  38.160  1.00  0.00
ATOM   3306  CA  GLN A 207       2.768  -8.871  38.764  1.00  0.00           C
ATOM   3307  HA  GLN A 207       2.498  -8.907  39.726  1.00  0.00
ATOM   3308  CB  GLN A 207       1.584  -8.400  37.933  1.00  0.00           C
ATOM   3309  HB1 GLN A 207       1.202  -7.572  38.344  1.00  0.00
ATOM   3310  HB2 GLN A 207       1.894  -8.199  37.004  1.00  0.00
ATOM   3311  CG  GLN A 207       0.508  -9.474  37.880  1.00  0.00           C
ATOM   3312  HG1 GLN A 207       0.713 -10.096  37.125  1.00  0.00
ATOM   3313  HG2 GLN A 207       0.519  -9.981  38.742  1.00  0.00
ATOM   3314  CD  GLN A 207      -0.875  -8.944  37.676  1.00  0.00           C
ATOM   3315  OE1 GLN A 207      -1.073  -7.771  37.410  1.00  0.00           O
ATOM   3316  NE2 GLN A 207      -1.847  -9.805  37.904  1.00  0.00           N
ATOM   3317 1HE2 GLN A 207      -1.631 -10.738  38.192  1.00  0.00
ATOM   3318 2HE2 GLN A 207      -2.800  -9.525  37.789  1.00  0.00
ATOM   3319  C   GLN A 207       3.245 -10.235  38.246  1.00  0.00           C
ATOM   3320  O   GLN A 207       2.849 -11.259  38.775  1.00  0.00           O
ATOM   3321  N   CYS A 208       4.076 -10.221  37.196  1.00  0.00           N
ATOM   3322  HN  CYS A 208       4.337  -9.338  36.806  1.00  0.00
ATOM   3323  CA  CYS A 208       4.618 -11.435  36.595  1.00  0.00           C
ATOM   3324  HA  CYS A 208       3.819 -11.913  36.231  1.00  0.00
ATOM   3325  CB  CYS A 208       5.603 -11.122  35.472  1.00  0.00           C
ATOM   3326  HB1 CYS A 208       6.234 -10.460  35.878  1.00  0.00
ATOM   3327  HB2 CYS A 208       6.081 -11.987  35.318  1.00  0.00
ATOM   3328  SG  CYS A 208       4.844 -10.489  33.952  1.00  0.00           S
ATOM   3329  HG1 CYS A 208       5.556 -10.311  33.273  1.00  0.00
ATOM   3330  C   CYS A 208       5.353 -12.258  37.654  1.00  0.00           C
ATOM   3331  O   CYS A 208       5.280 -13.488  37.660  1.00  0.00           O
ATOM   3332  N   LEU A 209       6.089 -11.557  38.521  1.00  0.00           N
```

```
ATOM   3333  HN  LEU A 209       5.990 -10.562  38.528  1.00  0.00
ATOM   3334  CA  LEU A 209       7.027 -12.161  39.455  1.00  0.00           C
ATOM   3335  HA  LEU A 209       7.460 -12.934  38.991  1.00  0.00
ATOM   3336  CB  LEU A 209       8.064 -11.103  39.848  1.00  0.00           C
ATOM   3337  HB1 LEU A 209       8.509 -10.784  39.011  1.00  0.00
ATOM   3338  HB2 LEU A 209       7.578 -10.342  40.278  1.00  0.00
ATOM   3339  CG  LEU A 209       9.158 -11.560  40.812  1.00  0.00           C
ATOM   3340  HG  LEU A 209       8.718 -11.928  41.631  1.00  0.00
ATOM   3341  CD1 LEU A 209      10.016 -12.650  40.180  1.00  0.00           C
ATOM   3342 1HD1 LEU A 209      10.724 -12.934  40.827  1.00  0.00
ATOM   3343 2HD1 LEU A 209       9.441 -13.435  39.950  1.00  0.00
ATOM   3344 3HD1 LEU A 209      10.446 -12.296  39.349  1.00  0.00
ATOM   3345  CD2 LEU A 209      10.036 -10.391  41.214  1.00  0.00           C
ATOM   3346 1HD2 LEU A 209      10.745 -10.708  41.844  1.00  0.00
ATOM   3347 2HD2 LEU A 209      10.464 -10.000  40.399  1.00  0.00
ATOM   3348 3HD2 LEU A 209       9.478  -9.694  41.664  1.00  0.00
ATOM   3349  C   LEU A 209       6.280 -12.665  40.691  1.00  0.00           C
ATOM   3350  O   LEU A 209       6.476 -13.798  41.107  1.00  0.00           O
ATOM   3351  N   VAL A 210       5.414 -11.805  41.232  1.00  0.00           N
ATOM   3352  HN  VAL A 210       5.165 -10.996  40.700  1.00  0.00
ATOM   3353  CA  VAL A 210       4.810 -11.968  42.542  1.00  0.00           C
ATOM   3354  HA  VAL A 210       5.412 -12.596  43.034  1.00  0.00
ATOM   3355  CB  VAL A 210       4.746 -10.620  43.279  1.00  0.00           C
ATOM   3356  HB  VAL A 210       4.285  -9.963  42.682  1.00  0.00
ATOM   3357  CG1 VAL A 210       3.929 -10.706  44.552  1.00  0.00           C
ATOM   3358 1HG1 VAL A 210       3.913  -9.811  44.999  1.00  0.00
ATOM   3359 2HG1 VAL A 210       2.995 -10.985  44.330  1.00  0.00
ATOM   3360 3HG1 VAL A 210       4.340 -11.379  45.167  1.00  0.00
ATOM   3361  CG2 VAL A 210       6.127 -10.081  43.557  1.00  0.00           C
ATOM   3362 1HG2 VAL A 210       6.053  -9.206  44.036  1.00  0.00
ATOM   3363 2HG2 VAL A 210       6.630 -10.731  44.126  1.00  0.00
ATOM   3364 3HG2 VAL A 210       6.613  -9.948  42.693  1.00  0.00
ATOM   3365  C   VAL A 210       3.406 -12.578  42.409  1.00  0.00           C
ATOM   3366  O   VAL A 210       3.028 -13.391  43.216  1.00  0.00           O
ATOM   3367  N   GLY A 211       2.629 -12.123  41.423  1.00  0.00           N
ATOM   3368  HN  GLY A 211       2.946 -11.351  40.873  1.00  0.00
ATOM   3369  CA  GLY A 211       1.325 -12.722  41.123  1.00  0.00           C
ATOM   3370  HA1 GLY A 211       1.299 -12.927  40.145  1.00  0.00
ATOM   3371  HA2 GLY A 211       1.251 -13.573  41.644  1.00  0.00
ATOM   3372  C   GLY A 211       0.165 -11.809  41.479  1.00  0.00           C
ATOM   3373  O   GLY A 211      -0.993 -12.204  41.393  1.00  0.00           O
ATOM   3374  N   LYS A 212       0.480 -10.584  41.889  1.00  0.00           N
ATOM   3375  HN  LYS A 212       1.425 -10.388  42.150  1.00  0.00
ATOM   3376  CA  LYS A 212      -0.500  -9.531  41.969  1.00  0.00           C
ATOM   3377  HA  LYS A 212      -1.187  -9.717  41.267  1.00  0.00
ATOM   3378  CB  LYS A 212      -1.193  -9.524  43.335  1.00  0.00           C
ATOM   3379  HB1 LYS A 212      -2.006  -8.946  43.263  1.00  0.00
ATOM   3380  HB2 LYS A 212      -1.468 -10.462  43.544  1.00  0.00
ATOM   3381  CG  LYS A 212      -0.373  -9.016  44.510  1.00  0.00           C
ATOM   3382  HG1 LYS A 212       0.555  -9.381  44.434  1.00  0.00
ATOM   3383  HG2 LYS A 212      -0.342  -8.017  44.471  1.00  0.00
ATOM   3384  CD  LYS A 212      -0.983  -9.453  45.857  1.00  0.00           C
ATOM   3385  HD1 LYS A 212      -1.976  -9.504  45.753  1.00  0.00
ATOM   3386  HD2 LYS A 212      -0.625 -10.357  46.088  1.00  0.00
ATOM   3387  CE  LYS A 212      -0.687  -8.526  47.022  1.00  0.00           C
ATOM   3388  HE1 LYS A 212      -0.976  -7.597  46.791  1.00  0.00
ATOM   3389  HE2 LYS A 212      -1.185  -8.838  47.831  1.00  0.00
ATOM   3390  NZ  LYS A 212       0.759  -8.502  47.341  1.00  0.00           N
ATOM   3391  HZ1 LYS A 212       0.920  -7.884  48.110  1.00  0.00
ATOM   3392  HZ2 LYS A 212       1.062  -9.423  47.584  1.00  0.00
ATOM   3393  HZ3 LYS A 212       1.271  -8.182  46.544  1.00  0.00
ATOM   3394  C   LYS A 212       0.204  -8.216  41.696  1.00  0.00           C
ATOM   3395  O   LYS A 212       1.437  -8.145  41.760  1.00  0.00           O
ATOM   3396  N   PRO A 213      -0.539  -7.143  41.343  1.00  0.00           N
ATOM   3397  CD  PRO A 213      -1.986  -7.129  41.101  1.00  0.00           C
ATOM   3398  HD1 PRO A 213      -2.485  -7.031  41.962  1.00  0.00
ATOM   3399  HD2 PRO A 213      -2.274  -7.970  40.643  1.00  0.00
ATOM   3400  CA  PRO A 213       0.071  -5.829  41.159  1.00  0.00           C
ATOM   3401  HA  PRO A 213       0.852  -5.896  40.539  1.00  0.00
ATOM   3402  CB  PRO A 213      -1.067  -4.941  40.618  1.00  0.00           C
ATOM   3403  HB1 PRO A 213      -1.399  -4.318  41.326  1.00  0.00
```

ATOM	3404	HB2	PRO	A	213	-0.759	-4.412	39.827	1.00	0.00	
ATOM	3405	CG	PRO	A	213	-2.166	-5.919	40.211	1.00	0.00	C
ATOM	3406	HG1	PRO	A	213	-3.069	-5.514	40.356	1.00	0.00	
ATOM	3407	HG2	PRO	A	213	-2.068	-6.178	39.250	1.00	0.00	
ATOM	3408	C	PRO	A	213	0.601	-5.299	42.483	1.00	0.00	C
ATOM	3409	O	PRO	A	213	0.140	-5.692	43.546	1.00	0.00	O
ATOM	3410	N	PRO	A	214	1.590	-4.390	42.457	1.00	0.00	N
ATOM	3411	CD	PRO	A	214	2.204	-3.829	41.237	1.00	0.00	C
ATOM	3412	HD1	PRO	A	214	1.498	-3.529	40.596	1.00	0.00	
ATOM	3413	HD2	PRO	A	214	2.789	-4.509	40.795	1.00	0.00	
ATOM	3414	CA	PRO	A	214	2.154	-3.852	43.694	1.00	0.00	C
ATOM	3415	HA	PRO	A	214	2.383	-4.617	44.296	1.00	0.00	
ATOM	3416	CB	PRO	A	214	3.355	-3.020	43.201	1.00	0.00	C
ATOM	3417	HB1	PRO	A	214	3.466	-2.193	43.752	1.00	0.00	
ATOM	3418	HB2	PRO	A	214	4.198	-3.557	43.232	1.00	0.00	
ATOM	3419	CG	PRO	A	214	3.013	-2.656	41.766	1.00	0.00	C
ATOM	3420	HG1	PRO	A	214	2.472	-1.816	41.737	1.00	0.00	
ATOM	3421	HG2	PRO	A	214	3.847	-2.534	41.227	1.00	0.00	
ATOM	3422	C	PRO	A	214	1.165	-2.971	44.499	1.00	0.00	C
ATOM	3423	O	PRO	A	214	1.291	-2.857	45.719	1.00	0.00	O
ATOM	3424	N	PHE	A	215	0.202	-2.348	43.814	1.00	0.00	N
ATOM	3425	HN	PHE	A	215	0.081	-2.571	42.847	1.00	0.00	
ATOM	3426	CA	PHE	A	215	-0.676	-1.361	44.415	1.00	0.00	C
ATOM	3427	HA	PHE	A	215	-0.680	-1.537	45.399	1.00	0.00	
ATOM	3428	CB	PHE	A	215	-0.130	0.049	44.161	1.00	0.00	C
ATOM	3429	HB1	PHE	A	215	-0.176	0.236	43.180	1.00	0.00	
ATOM	3430	HB2	PHE	A	215	-0.703	0.705	44.652	1.00	0.00	
ATOM	3431	CG	PHE	A	215	1.297	0.260	44.604	1.00	0.00	C
ATOM	3432	CD1	PHE	A	215	1.638	0.258	45.950	1.00	0.00	C
ATOM	3433	HD1	PHE	A	215	0.926	0.115	46.638	1.00	0.00	
ATOM	3434	CE1	PHE	A	215	2.951	0.452	46.356	1.00	0.00	C
ATOM	3435	HE1	PHE	A	215	3.182	0.425	47.328	1.00	0.00	
ATOM	3436	CZ	PHE	A	215	3.934	0.681	45.422	1.00	0.00	C
ATOM	3437	HZ	PHE	A	215	4.876	0.847	45.714	1.00	0.00	
ATOM	3438	CD2	PHE	A	215	2.299	0.486	43.673	1.00	0.00	C
ATOM	3439	HD2	PHE	A	215	2.073	0.509	42.699	1.00	0.00	
ATOM	3440	CE2	PHE	A	215	3.613	0.681	44.078	1.00	0.00	C
ATOM	3441	HE2	PHE	A	215	4.329	0.822	43.395	1.00	0.00	
ATOM	3442	C	PHE	A	215	-2.095	-1.532	43.864	1.00	0.00	C
ATOM	3443	O	PHE	A	215	-2.345	-1.268	42.695	1.00	0.00	O
ATOM	3444	N	GLN	A	216	-3.006	-1.987	44.727	1.00	0.00	N
ATOM	3445	HN	GLN	A	216	-2.705	-2.198	45.657	1.00	0.00	
ATOM	3446	CA	GLN	A	216	-4.420	-2.197	44.400	1.00	0.00	C
ATOM	3447	HA	GLN	A	216	-4.512	-2.106	43.408	1.00	0.00	
ATOM	3448	CB	GLN	A	216	-4.862	-3.600	44.825	1.00	0.00	C
ATOM	3449	HB1	GLN	A	216	-4.960	-3.615	45.820	1.00	0.00	
ATOM	3450	HB2	GLN	A	216	-5.745	-3.798	44.400	1.00	0.00	
ATOM	3451	CG	GLN	A	216	-3.886	-4.695	44.426	1.00	0.00	C
ATOM	3452	HG1	GLN	A	216	-3.652	-4.582	43.460	1.00	0.00	
ATOM	3453	HG2	GLN	A	216	-3.059	-4.608	44.982	1.00	0.00	
ATOM	3454	CD	GLN	A	216	-4.452	-6.081	44.622	1.00	0.00	C
ATOM	3455	OE1	GLN	A	216	-5.497	-6.444	44.074	1.00	0.00	O
ATOM	3456	NE2	GLN	A	216	-3.731	-6.886	45.386	1.00	0.00	N
ATOM	3457	1HE2	GLN	A	216	-2.875	-6.560	45.788	1.00	0.00	
ATOM	3458	2HE2	GLN	A	216	-4.042	-7.820	45.562	1.00	0.00	
ATOM	3459	C	GLN	A	216	-5.255	-1.139	45.125	1.00	0.00	C
ATOM	3460	O	GLN	A	216	-4.816	-0.571	46.134	1.00	0.00	O
ATOM	3461	N	ALA	A	217	-6.469	-0.898	44.626	1.00	0.00	N
ATOM	3462	HN	ALA	A	217	-6.742	-1.339	43.771	1.00	0.00	
ATOM	3463	CA	ALA	A	217	-7.398	-0.014	45.294	1.00	0.00	C
ATOM	3464	HA	ALA	A	217	-7.258	-0.140	46.276	1.00	0.00	
ATOM	3465	CB	ALA	A	217	-7.091	1.414	44.942	1.00	0.00	C
ATOM	3466	HB1	ALA	A	217	-7.737	2.018	45.408	1.00	0.00	
ATOM	3467	HB2	ALA	A	217	-6.160	1.636	45.231	1.00	0.00	
ATOM	3468	HB3	ALA	A	217	-7.172	1.538	43.953	1.00	0.00	
ATOM	3469	C	ALA	A	217	-8.832	-0.380	44.921	1.00	0.00	C
ATOM	3470	O	ALA	A	217	-9.063	-1.120	43.964	1.00	0.00	O
ATOM	3471	N	ASN	A	218	-9.778	0.186	45.686	1.00	0.00	N
ATOM	3472	HN	ASN	A	218	-9.502	0.897	46.333	1.00	0.00	
ATOM	3473	CA	ASN	A	218	-11.197	-0.184	45.623	1.00	0.00	C
ATOM	3474	HA	ASN	A	218	-11.203	-1.176	45.495	1.00	0.00	

```
ATOM   3475  CB  ASN A 218     -11.933   0.144  46.928  1.00  0.00           C
ATOM   3476  HB1 ASN A 218     -11.959   1.138  47.039  1.00  0.00
ATOM   3477  HB2 ASN A 218     -12.867  -0.209  46.865  1.00  0.00
ATOM   3478  CG  ASN A 218     -11.286  -0.452  48.164  1.00  0.00           C
ATOM   3479  OD1 ASN A 218     -10.584  -1.461  48.089  1.00  0.00           O
ATOM   3480  ND2 ASN A 218     -11.519   0.168  49.310  1.00  0.00           N
ATOM   3481 1HD2 ASN A 218     -12.096   0.985  49.328  1.00  0.00
ATOM   3482 2HD2 ASN A 218     -11.118  -0.180  50.157  1.00  0.00
ATOM   3483  C   ASN A 218     -11.871   0.527  44.437  1.00  0.00           C
ATOM   3484  O   ASN A 218     -12.927   0.113  43.990  1.00  0.00           O
ATOM   3485  N   SER A 219     -11.249   1.603  43.940  1.00  0.00           N
ATOM   3486  HN  SER A 219     -10.364   1.863  44.326  1.00  0.00
ATOM   3487  CA  SER A 219     -11.801   2.412  42.865  1.00  0.00           C
ATOM   3488  HA  SER A 219     -12.172   1.775  42.189  1.00  0.00
ATOM   3489  CB  SER A 219     -12.933   3.265  43.375  1.00  0.00           C
ATOM   3490  HB1 SER A 219     -13.471   3.621  42.611  1.00  0.00
ATOM   3491  HB2 SER A 219     -13.525   2.731  43.979  1.00  0.00
ATOM   3492  OG  SER A 219     -12.444   4.374  44.116  1.00  0.00           O
ATOM   3493  HG1 SER A 219     -13.214   4.922  44.442  1.00  0.00
ATOM   3494  C   SER A 219     -10.700   3.264  42.230  1.00  0.00           C
ATOM   3495  O   SER A 219      -9.584   3.341  42.754  1.00  0.00           O
ATOM   3496  N   PRO A 220     -10.974   3.907  41.065  1.00  0.00           N
ATOM   3497  CD  PRO A 220     -12.132   3.638  40.191  1.00  0.00           C
ATOM   3498  HD1 PRO A 220     -12.949   4.098  40.538  1.00  0.00
ATOM   3499  HD2 PRO A 220     -12.304   2.655  40.128  1.00  0.00
ATOM   3500  CA  PRO A 220     -10.050   4.886  40.482  1.00  0.00           C
ATOM   3501  HA  PRO A 220      -9.176   4.427  40.323  1.00  0.00
ATOM   3502  CB  PRO A 220     -10.791   5.370  39.226  1.00  0.00           C
ATOM   3503  HB1 PRO A 220     -11.330   6.189  39.421  1.00  0.00
ATOM   3504  HB2 PRO A 220     -10.150   5.562  38.483  1.00  0.00
ATOM   3505  CG  PRO A 220     -11.698   4.210  38.860  1.00  0.00           C
ATOM   3506  HG1 PRO A 220     -12.489   4.529  38.338  1.00  0.00
ATOM   3507  HG2 PRO A 220     -11.201   3.525  38.327  1.00  0.00
ATOM   3508  C   PRO A 220      -9.715   6.080  41.397  1.00  0.00           C
ATOM   3509  O   PRO A 220      -8.565   6.508  41.437  1.00  0.00           O
ATOM   3510  N   GLN A 221     -10.716   6.608  42.118  1.00  0.00           N
ATOM   3511  HN  GLN A 221     -11.613   6.166  42.095  1.00  0.00
ATOM   3512  CA  GLN A 221     -10.539   7.815  42.941  1.00  0.00           C
ATOM   3513  HA  GLN A 221      -9.973   8.438  42.402  1.00  0.00
ATOM   3514  CB  GLN A 221     -11.882   8.502  43.201  1.00  0.00           C
ATOM   3515  HB1 GLN A 221     -12.594   7.802  43.255  1.00  0.00
ATOM   3516  HB2 GLN A 221     -11.828   8.990  44.072  1.00  0.00
ATOM   3517  CG  GLN A 221     -12.262   9.500  42.105  1.00  0.00           C
ATOM   3518  HG1 GLN A 221     -13.085   9.991  42.390  1.00  0.00
ATOM   3519  HG2 GLN A 221     -11.511  10.149  41.988  1.00  0.00
ATOM   3520  CD  GLN A 221     -12.538   8.847  40.767  1.00  0.00           C
ATOM   3521  OE1 GLN A 221     -13.025   7.720  40.696  1.00  0.00           O
ATOM   3522  NE2 GLN A 221     -12.236   9.558  39.688  1.00  0.00           N
ATOM   3523 1HE2 GLN A 221     -11.848  10.474  39.786  1.00  0.00
ATOM   3524 2HE2 GLN A 221     -12.397   9.177  38.777  1.00  0.00
ATOM   3525  C   GLN A 221      -9.799   7.476  44.247  1.00  0.00           C
ATOM   3526  O   GLN A 221      -9.163   8.359  44.842  1.00  0.00           O
ATOM   3527  N   ASP A 222      -9.871   6.206  44.673  1.00  0.00           N
ATOM   3528  HN  ASP A 222     -10.433   5.564  44.152  1.00  0.00
ATOM   3529  CA  ASP A 222      -9.161   5.701  45.878  1.00  0.00           C
ATOM   3530  HA  ASP A 222      -9.351   6.360  46.605  1.00  0.00
ATOM   3531  CB  ASP A 222      -9.693   4.328  46.321  1.00  0.00           C
ATOM   3532  HB1 ASP A 222     -10.088   3.875  45.522  1.00  0.00
ATOM   3533  HB2 ASP A 222      -8.922   3.791  46.662  1.00  0.00
ATOM   3534  CG  ASP A 222     -10.758   4.358  47.413  1.00  0.00           C
ATOM   3535  OD1 ASP A 222     -11.281   5.449  47.703  1.00  0.00           O
ATOM   3536  OD2 ASP A 222     -11.048   3.285  47.973  1.00  0.00           O
ATOM   3537  C   ASP A 222      -7.649   5.634  45.609  1.00  0.00           C
ATOM   3538  O   ASP A 222      -6.839   5.972  46.489  1.00  0.00           O
ATOM   3539  N   LEU A 223      -7.282   5.189  44.397  1.00  0.00           N
ATOM   3540  HN  LEU A 223      -7.996   4.930  43.746  1.00  0.00
ATOM   3541  CA  LEU A 223      -5.872   5.065  43.983  1.00  0.00           C
ATOM   3542  HA  LEU A 223      -5.372   4.644  44.740  1.00  0.00
ATOM   3543  CB  LEU A 223      -5.792   4.162  42.746  1.00  0.00           C
ATOM   3544  HB1 LEU A 223      -6.236   3.294  42.969  1.00  0.00
ATOM   3545  HB2 LEU A 223      -6.291   4.610  42.004  1.00  0.00
```

ATOM	3546	CG	LEU	A	223	-4.378	3.853	42.243	1.00	0.00	C
ATOM	3547	HG	LEU	A	223	-3.998	4.710	41.897	1.00	0.00	
ATOM	3548	CD1	LEU	A	223	-3.460	3.354	43.358	1.00	0.00	C
ATOM	3549	1HD1	LEU	A	223	-2.552	3.166	42.983	1.00	0.00	
ATOM	3550	2HD1	LEU	A	223	-3.389	4.053	44.069	1.00	0.00	
ATOM	3551	3HD1	LEU	A	223	-3.838	2.516	43.751	1.00	0.00	
ATOM	3552	CD2	LEU	A	223	-4.418	2.835	41.113	1.00	0.00	C
ATOM	3553	1HD2	LEU	A	223	-3.487	2.648	40.800	1.00	0.00	
ATOM	3554	2HD2	LEU	A	223	-4.836	1.988	41.442	1.00	0.00	
ATOM	3555	3HD2	LEU	A	223	-4.958	3.200	40.354	1.00	0.00	
ATOM	3556	C	LEU	A	223	-5.271	6.458	43.701	1.00	0.00	C
ATOM	3557	O	LEU	A	223	-4.068	6.668	43.885	1.00	0.00	O
ATOM	3558	N	ARG	A	224	-6.116	7.391	43.239	1.00	0.00	N
ATOM	3559	HN	ARG	A	224	-7.063	7.124	43.062	1.00	0.00	
ATOM	3560	CA	ARG	A	224	-5.726	8.785	42.979	1.00	0.00	C
ATOM	3561	HA	ARG	A	224	-4.988	8.753	42.305	1.00	0.00	
ATOM	3562	CB	ARG	A	224	-6.906	9.575	42.398	1.00	0.00	C
ATOM	3563	HB1	ARG	A	224	-7.253	9.081	41.601	1.00	0.00	
ATOM	3564	HB2	ARG	A	224	-7.621	9.629	43.095	1.00	0.00	
ATOM	3565	CG	ARG	A	224	-6.568	10.994	41.960	1.00	0.00	C
ATOM	3566	HG1	ARG	A	224	-5.757	11.301	42.458	1.00	0.00	
ATOM	3567	HG2	ARG	A	224	-6.376	10.992	40.979	1.00	0.00	
ATOM	3568	CD	ARG	A	224	-7.707	11.965	42.234	1.00	0.00	C
ATOM	3569	HD1	ARG	A	224	-8.584	11.486	42.199	1.00	0.00	
ATOM	3570	HD2	ARG	A	224	-7.593	12.385	43.134	1.00	0.00	
ATOM	3571	NE	ARG	A	224	-7.728	13.023	41.240	1.00	0.00	N
ATOM	3572	HE	ARG	A	224	-8.533	13.092	40.650	1.00	0.00	
ATOM	3573	CZ	ARG	A	224	-6.750	13.906	41.060	1.00	0.00	C
ATOM	3574	NH1	ARG	A	224	-5.726	13.933	41.895	1.00	0.00	N
ATOM	3575	1HH1	ARG	A	224	-5.688	13.290	42.660	1.00	0.00	
ATOM	3576	2HH1	ARG	A	224	-4.989	14.596	41.762	1.00	0.00	
ATOM	3577	NH2	ARG	A	224	-6.786	14.740	40.031	1.00	0.00	N
ATOM	3578	1HH2	ARG	A	224	-7.550	14.707	39.387	1.00	0.00	
ATOM	3579	2HH2	ARG	A	224	-6.049	15.403	39.899	1.00	0.00	
ATOM	3580	C	ARG	A	224	-5.220	9.435	44.272	1.00	0.00	C
ATOM	3581	O	ARG	A	224	-4.134	10.007	44.285	1.00	0.00	O
ATOM	3582	N	MET	A	225	-6.007	9.318	45.350	1.00	0.00	N
ATOM	3583	HN	MET	A	225	-6.822	8.742	45.284	1.00	0.00	
ATOM	3584	CA	MET	A	225	-5.730	9.998	46.632	1.00	0.00	C
ATOM	3585	HA	MET	A	225	-5.418	10.916	46.386	1.00	0.00	
ATOM	3586	CB	MET	A	225	-7.002	10.113	47.481	1.00	0.00	C
ATOM	3587	HB1	MET	A	225	-7.393	9.200	47.601	1.00	0.00	
ATOM	3588	HB2	MET	A	225	-6.760	10.492	48.374	1.00	0.00	
ATOM	3589	CG	MET	A	225	-8.055	11.016	46.834	1.00	0.00	C
ATOM	3590	HG1	MET	A	225	-7.602	11.903	46.744	1.00	0.00	
ATOM	3591	HG2	MET	A	225	-8.199	10.621	45.926	1.00	0.00	
ATOM	3592	SD	MET	A	225	-9.599	11.137	47.771	1.00	0.00	S
ATOM	3593	CE	MET	A	225	-10.649	11.991	46.589	1.00	0.00	C
ATOM	3594	HE1	MET	A	225	-11.556	12.127	46.987	1.00	0.00	
ATOM	3595	HE2	MET	A	225	-10.246	12.879	46.366	1.00	0.00	
ATOM	3596	HE3	MET	A	225	-10.729	11.442	45.757	1.00	0.00	
ATOM	3597	C	MET	A	225	-4.623	9.262	47.413	1.00	0.00	C
ATOM	3598	O	MET	A	225	-3.890	9.894	48.187	1.00	0.00	O
ATOM	3599	N	PHE	A	226	-4.489	7.944	47.190	1.00	0.00	N
ATOM	3600	HN	PHE	A	226	-5.191	7.473	46.655	1.00	0.00	
ATOM	3601	CA	PHE	A	226	-3.346	7.163	47.704	1.00	0.00	C
ATOM	3602	HA	PHE	A	226	-3.350	7.224	48.702	1.00	0.00	
ATOM	3603	CB	PHE	A	226	-3.511	5.689	47.324	1.00	0.00	C
ATOM	3604	HB1	PHE	A	226	-4.396	5.379	47.672	1.00	0.00	
ATOM	3605	HB2	PHE	A	226	-3.505	5.626	46.326	1.00	0.00	
ATOM	3606	CG	PHE	A	226	-2.466	4.729	47.839	1.00	0.00	C
ATOM	3607	CD1	PHE	A	226	-2.611	4.103	49.070	1.00	0.00	C
ATOM	3608	HD1	PHE	A	226	-3.395	4.327	49.649	1.00	0.00	
ATOM	3609	CE1	PHE	A	226	-1.679	3.168	49.506	1.00	0.00	C
ATOM	3610	HE1	PHE	A	226	-1.794	2.730	50.398	1.00	0.00	
ATOM	3611	CZ	PHE	A	226	-0.596	2.840	48.715	1.00	0.00	C
ATOM	3612	HZ	PHE	A	226	0.076	2.171	49.033	1.00	0.00	
ATOM	3613	CD2	PHE	A	226	-1.380	4.378	47.052	1.00	0.00	C
ATOM	3614	HD2	PHE	A	226	-1.265	4.807	46.156	1.00	0.00	
ATOM	3615	CE2	PHE	A	226	-0.448	3.441	47.485	1.00	0.00	C
ATOM	3616	HE2	PHE	A	226	0.330	3.204	46.903	1.00	0.00	

```
ATOM   3617  C   PHE A 226      -2.033   7.769  47.175  1.00  0.00           C
ATOM   3618  O   PHE A 226      -1.147   8.138  47.947  1.00  0.00           O
ATOM   3619  N   TYR A 227      -1.928   7.892  45.849  1.00  0.00           N
ATOM   3620  HN  TYR A 227      -2.704   7.620  45.280  1.00  0.00
ATOM   3621  CA  TYR A 227      -0.720   8.410  45.189  1.00  0.00           C
ATOM   3622  HA  TYR A 227       0.046   7.887  45.563  1.00  0.00
ATOM   3623  CB  TYR A 227      -0.794   8.189  43.673  1.00  0.00           C
ATOM   3624  HB1 TYR A 227      -1.705   8.461  43.363  1.00  0.00
ATOM   3625  HB2 TYR A 227      -0.107   8.772  43.240  1.00  0.00
ATOM   3626  CG  TYR A 227      -0.554   6.770  43.199  1.00  0.00           C
ATOM   3627  CD1 TYR A 227       0.373   5.930  43.806  1.00  0.00           C
ATOM   3628  HD1 TYR A 227       0.886   6.261  44.598  1.00  0.00
ATOM   3629  CE1 TYR A 227       0.605   4.643  43.340  1.00  0.00           C
ATOM   3630  HE1 TYR A 227       1.267   4.054  43.803  1.00  0.00
ATOM   3631  CZ  TYR A 227      -0.082   4.176  42.233  1.00  0.00           C
ATOM   3632  OH  TYR A 227       0.120   2.922  41.736  1.00  0.00           O
ATOM   3633  HH  TYR A 227      -0.469   2.780  40.940  1.00  0.00
ATOM   3634  CD2 TYR A 227      -1.209   6.288  42.078  1.00  0.00           C
ATOM   3635  HD2 TYR A 227      -1.853   6.882  41.597  1.00  0.00
ATOM   3636  CE2 TYR A 227      -0.994   5.003  41.606  1.00  0.00           C
ATOM   3637  HE2 TYR A 227      -1.500   4.673  40.809  1.00  0.00
ATOM   3638  C   TYR A 227      -0.535   9.898  45.510  1.00  0.00           C
ATOM   3639  O   TYR A 227       0.576  10.363  45.674  1.00  0.00           O
ATOM   3640  N   GLU A 228      -1.645  10.632  45.556  1.00  0.00           N
ATOM   3641  HN  GLU A 228      -2.520  10.163  45.435  1.00  0.00
ATOM   3642  CA  GLU A 228      -1.670  12.095  45.774  1.00  0.00           C
ATOM   3643  HA  GLU A 228      -1.114  12.524  45.062  1.00  0.00
ATOM   3644  CB  GLU A 228      -3.112  12.591  45.665  1.00  0.00           C
ATOM   3645  HB1 GLU A 228      -3.450  12.362  44.752  1.00  0.00
ATOM   3646  HB2 GLU A 228      -3.657  12.111  46.353  1.00  0.00
ATOM   3647  CG  GLU A 228      -3.319  14.078  45.871  1.00  0.00           C
ATOM   3648  HG1 GLU A 228      -2.787  14.364  46.668  1.00  0.00
ATOM   3649  HG2 GLU A 228      -2.988  14.559  45.059  1.00  0.00
ATOM   3650  CD  GLU A 228      -4.779  14.453  46.099  1.00  0.00           C
ATOM   3651  OE1 GLU A 228      -5.668  13.624  45.741  1.00  0.00           O
ATOM   3652  OE2 GLU A 228      -5.036  15.562  46.660  1.00  0.00           O
ATOM   3653  C   GLU A 228      -1.058  12.440  47.133  1.00  0.00           C
ATOM   3654  O   GLU A 228      -0.339  13.438  47.239  1.00  0.00           O
ATOM   3655  N   LYS A 229      -1.303  11.583  48.142  1.00  0.00           N
ATOM   3656  HN  LYS A 229      -1.841  10.762  47.953  1.00  0.00
ATOM   3657  CA  LYS A 229      -0.809  11.806  49.521  1.00  0.00           C
ATOM   3658  HA  LYS A 229      -0.607  12.785  49.520  1.00  0.00
ATOM   3659  CB  LYS A 229      -1.884  11.452  50.556  1.00  0.00           C
ATOM   3660  HB1 LYS A 229      -1.854  10.463  50.699  1.00  0.00
ATOM   3661  HB2 LYS A 229      -1.650  11.919  51.409  1.00  0.00
ATOM   3662  CG  LYS A 229      -3.324  11.821  50.212  1.00  0.00           C
ATOM   3663  HG1 LYS A 229      -3.316  12.332  49.353  1.00  0.00
ATOM   3664  HG2 LYS A 229      -3.840  10.973  50.091  1.00  0.00
ATOM   3665  CD  LYS A 229      -4.043  12.667  51.259  1.00  0.00           C
ATOM   3666  HD1 LYS A 229      -3.759  12.367  52.170  1.00  0.00
ATOM   3667  HD2 LYS A 229      -3.792  13.626  51.130  1.00  0.00
ATOM   3668  CE  LYS A 229      -5.547  12.544  51.157  1.00  0.00           C
ATOM   3669  HE1 LYS A 229      -5.807  12.428  50.198  1.00  0.00
ATOM   3670  HE2 LYS A 229      -5.852  11.750  51.683  1.00  0.00
ATOM   3671  NZ  LYS A 229      -6.227  13.749  51.686  1.00  0.00           N
ATOM   3672  HZ1 LYS A 229      -7.217  13.636  51.604  1.00  0.00
ATOM   3673  HZ2 LYS A 229      -5.984  13.874  52.648  1.00  0.00
ATOM   3674  HZ3 LYS A 229      -5.939  14.551  51.163  1.00  0.00
ATOM   3675  C   LYS A 229       0.475  11.004  49.867  1.00  0.00           C
ATOM   3676  O   LYS A 229       0.833  10.923  51.023  1.00  0.00           O
ATOM   3677  N   ASN A 230       1.173  10.469  48.861  1.00  0.00           N
ATOM   3678  HN  ASN A 230       0.860  10.671  47.933  1.00  0.00
ATOM   3679  CA  ASN A 230       2.328   9.633  48.989  1.00  0.00           C
ATOM   3680  HA  ASN A 230       2.389   9.176  48.102  1.00  0.00
ATOM   3681  CB  ASN A 230       3.587  10.459  49.222  1.00  0.00           C
ATOM   3682  HB1 ASN A 230       3.431  11.388  48.885  1.00  0.00
ATOM   3683  HB2 ASN A 230       3.777  10.488  50.203  1.00  0.00
ATOM   3684  CG  ASN A 230       4.796   9.876  48.506  1.00  0.00           C
ATOM   3685  OD1 ASN A 230       4.912   8.659  48.317  1.00  0.00           O
ATOM   3686  ND2 ASN A 230       5.694  10.737  48.068  1.00  0.00           N
ATOM   3687 1HD2 ASN A 230       5.560  11.717  48.214  1.00  0.00
```

ATOM	3688	2HD2	ASN	A	230	6.509	10.410	47.590	1.00	0.00	
ATOM	3689	C	ASN	A	230	2.141	8.592	50.090	1.00	0.00	C
ATOM	3690	O	ASN	A	230	3.113	8.193	50.738	1.00	0.00	O
ATOM	3691	N	ARG	A	231	0.903	8.121	50.259	1.00	0.00	N
ATOM	3692	HN	ARG	A	231	0.139	8.603	49.831	1.00	0.00	
ATOM	3693	CA	ARG	A	231	0.629	6.919	51.053	1.00	0.00	C
ATOM	3694	HA	ARG	A	231	1.140	6.992	51.910	1.00	0.00	
ATOM	3695	CB	ARG	A	231	-0.861	6.831	51.410	1.00	0.00	C
ATOM	3696	HB1	ARG	A	231	-1.384	6.747	50.562	1.00	0.00	
ATOM	3697	HB2	ARG	A	231	-1.003	6.016	51.971	1.00	0.00	
ATOM	3698	CG	ARG	A	231	-1.395	8.034	52.179	1.00	0.00	C
ATOM	3699	HG1	ARG	A	231	-0.954	8.064	53.076	1.00	0.00	
ATOM	3700	HG2	ARG	A	231	-1.173	8.866	51.670	1.00	0.00	
ATOM	3701	CD	ARG	A	231	-2.888	7.991	52.387	1.00	0.00	C
ATOM	3702	HD1	ARG	A	231	-3.357	7.988	51.504	1.00	0.00	
ATOM	3703	HD2	ARG	A	231	-3.139	7.170	52.900	1.00	0.00	
ATOM	3704	NE	ARG	A	231	-3.324	9.159	53.140	1.00	0.00	N
ATOM	3705	HE	ARG	A	231	-2.619	9.765	53.509	1.00	0.00	
ATOM	3706	CZ	ARG	A	231	-4.594	9.476	53.371	1.00	0.00	C
ATOM	3707	NH1	ARG	A	231	-5.566	8.663	52.984	1.00	0.00	N
ATOM	3708	1HH1	ARG	A	231	-5.345	7.807	52.516	1.00	0.00	
ATOM	3709	2HH1	ARG	A	231	-6.520	8.905	53.160	1.00	0.00	
ATOM	3710	NH2	ARG	A	231	-4.891	10.613	53.976	1.00	0.00	N
ATOM	3711	1HH2	ARG	A	231	-4.160	11.234	54.260	1.00	0.00	
ATOM	3712	2HH2	ARG	A	231	-5.846	10.852	54.150	1.00	0.00	
ATOM	3713	C	ARG	A	231	1.107	5.700	50.252	1.00	0.00	C
ATOM	3714	O	ARG	A	231	0.442	4.664	50.222	1.00	0.00	O
ATOM	3715	N	SER	A	232	2.278	5.829	49.611	1.00	0.00	N
ATOM	3716	HN	SER	A	232	2.836	6.635	49.810	1.00	0.00	
ATOM	3717	CA	SER	A	232	2.791	4.860	48.636	1.00	0.00	C
ATOM	3718	HA	SER	A	232	2.279	4.007	48.733	1.00	0.00	
ATOM	3719	CB	SER	A	232	2.545	5.369	47.234	1.00	0.00	C
ATOM	3720	HB1	SER	A	232	1.564	5.371	47.042	1.00	0.00	
ATOM	3721	HB2	SER	A	232	2.904	6.298	47.144	1.00	0.00	
ATOM	3722	OG	SER	A	232	3.171	4.574	46.227	1.00	0.00	O
ATOM	3723	HG1	SER	A	232	2.972	4.960	45.326	1.00	0.00	
ATOM	3724	C	SER	A	232	4.278	4.594	48.925	1.00	0.00	C
ATOM	3725	O	SER	A	232	5.114	5.493	48.832	1.00	0.00	O
ATOM	3726	N	LEU	A	233	4.561	3.352	49.335	1.00	0.00	N
ATOM	3727	HN	LEU	A	233	3.804	2.702	49.407	1.00	0.00	
ATOM	3728	CA	LEU	A	233	5.896	2.880	49.684	1.00	0.00	C
ATOM	3729	HA	LEU	A	233	6.512	3.666	49.646	1.00	0.00	
ATOM	3730	CB	LEU	A	233	5.872	2.287	51.101	1.00	0.00	C
ATOM	3731	HB1	LEU	A	233	5.104	1.649	51.159	1.00	0.00	
ATOM	3732	HB2	LEU	A	233	6.729	1.792	51.247	1.00	0.00	
ATOM	3733	CG	LEU	A	233	5.722	3.299	52.229	1.00	0.00	C
ATOM	3734	HG	LEU	A	233	5.004	3.947	51.976	1.00	0.00	
ATOM	3735	CD1	LEU	A	233	5.300	2.600	53.508	1.00	0.00	C
ATOM	3736	1HD1	LEU	A	233	5.205	3.274	54.240	1.00	0.00	
ATOM	3737	2HD1	LEU	A	233	4.424	2.140	53.363	1.00	0.00	
ATOM	3738	3HD1	LEU	A	233	5.993	1.926	53.763	1.00	0.00	
ATOM	3739	CD2	LEU	A	233	7.014	4.090	52.437	1.00	0.00	C
ATOM	3740	1HD2	LEU	A	233	6.887	4.745	53.182	1.00	0.00	
ATOM	3741	2HD2	LEU	A	233	7.756	3.461	52.669	1.00	0.00	
ATOM	3742	3HD2	LEU	A	233	7.243	4.581	51.596	1.00	0.00	
ATOM	3743	C	LEU	A	233	6.323	1.812	48.674	1.00	0.00	C
ATOM	3744	O	LEU	A	233	5.500	1.024	48.211	1.00	0.00	O
ATOM	3745	N	MET	A	234	7.623	1.745	48.393	1.00	0.00	N
ATOM	3746	HN	MET	A	234	8.201	2.533	48.607	1.00	0.00	
ATOM	3747	CA	MET	A	234	8.238	0.574	47.788	1.00	0.00	C
ATOM	3748	HA	MET	A	234	8.185	0.710	46.799	1.00	0.00	
ATOM	3749	CB	MET	A	234	9.703	0.444	48.221	1.00	0.00	C
ATOM	3750	HB1	MET	A	234	10.015	1.332	48.559	1.00	0.00	
ATOM	3751	HB2	MET	A	234	9.759	-0.231	48.957	1.00	0.00	
ATOM	3752	CG	MET	A	234	10.626	0.001	47.075	1.00	0.00	C
ATOM	3753	HG1	MET	A	234	11.491	-0.222	47.525	1.00	0.00	
ATOM	3754	HG2	MET	A	234	10.206	-0.837	46.727	1.00	0.00	
ATOM	3755	SD	MET	A	234	10.827	1.281	45.764	1.00	0.00	S
ATOM	3756	CE	MET	A	234	12.037	2.357	46.540	1.00	0.00	C
ATOM	3757	HE1	MET	A	234	12.248	3.118	45.927	1.00	0.00	
ATOM	3758	HE2	MET	A	234	11.665	2.713	47.397	1.00	0.00	

```
ATOM   3759  HE3 MET A 234      12.871   1.839  46.728  1.00  0.00
ATOM   3760  C   MET A 234       7.466  -0.669  48.217  1.00  0.00           C
ATOM   3761  O   MET A 234       7.297  -0.917  49.402  1.00  0.00           O
ATOM   3762  N   PRO A 235       6.953  -1.486  47.270  1.00  0.00           N
ATOM   3763  CD  PRO A 235       7.048  -1.282  45.811  1.00  0.00           C
ATOM   3764  HD1 PRO A 235       8.003  -1.177  45.535  1.00  0.00
ATOM   3765  HD2 PRO A 235       6.531  -0.471  45.538  1.00  0.00
ATOM   3766  CA  PRO A 235       6.245  -2.717  47.621  1.00  0.00           C
ATOM   3767  HA  PRO A 235       5.679  -2.577  48.433  1.00  0.00
ATOM   3768  CB  PRO A 235       5.488  -3.019  46.320  1.00  0.00           C
ATOM   3769  HB1 PRO A 235       5.307  -3.998  46.229  1.00  0.00
ATOM   3770  HB2 PRO A 235       4.625  -2.515  46.282  1.00  0.00
ATOM   3771  CG  PRO A 235       6.442  -2.542  45.231  1.00  0.00           C
ATOM   3772  HG1 PRO A 235       7.150  -3.225  45.050  1.00  0.00
ATOM   3773  HG2 PRO A 235       5.948  -2.341  44.385  1.00  0.00
ATOM   3774  C   PRO A 235       7.201  -3.870  47.972  1.00  0.00           C
ATOM   3775  O   PRO A 235       8.340  -3.848  47.568  1.00  0.00           O
ATOM   3776  N   SER A 236       6.643  -4.930  48.544  1.00  0.00           N
ATOM   3777  HN  SER A 236       5.661  -4.891  48.728  1.00  0.00
ATOM   3778  CA  SER A 236       7.343  -6.132  48.920  1.00  0.00           C
ATOM   3779  HA  SER A 236       8.073  -5.816  49.526  1.00  0.00
ATOM   3780  CB  SER A 236       6.418  -7.044  49.665  1.00  0.00           C
ATOM   3781  HB1 SER A 236       5.554  -7.144  49.172  1.00  0.00
ATOM   3782  HB2 SER A 236       6.838  -7.943  49.787  1.00  0.00
ATOM   3783  OG  SER A 236       6.140  -6.501  50.950  1.00  0.00           O
ATOM   3784  HG1 SER A 236       5.522  -7.113  51.444  1.00  0.00
ATOM   3785  C   SER A 236       7.957  -6.859  47.712  1.00  0.00           C
ATOM   3786  O   SER A 236       7.245  -7.499  46.921  1.00  0.00           O
ATOM   3787  N   ILE A 237       9.295  -6.819  47.643  1.00  0.00           N
ATOM   3788  HN  ILE A 237       9.775  -6.168  48.231  1.00  0.00
ATOM   3789  CA  ILE A 237      10.099  -7.668  46.762  1.00  0.00           C
ATOM   3790  HA  ILE A 237       9.542  -7.829  45.947  1.00  0.00
ATOM   3791  CB  ILE A 237      11.413  -6.962  46.356  1.00  0.00           C
ATOM   3792  HB  ILE A 237      12.001  -6.919  47.163  1.00  0.00
ATOM   3793  CG2 ILE A 237      12.162  -7.774  45.285  1.00  0.00           C
ATOM   3794 1HG2 ILE A 237      13.007  -7.300  45.038  1.00  0.00
ATOM   3795 2HG2 ILE A 237      12.382  -8.680  45.647  1.00  0.00
ATOM   3796 3HG2 ILE A 237      11.584  -7.870  44.475  1.00  0.00
ATOM   3797  CG1 ILE A 237      11.193  -5.502  45.944  1.00  0.00           C
ATOM   3798 1HG1 ILE A 237      10.596  -5.060  46.614  1.00  0.00
ATOM   3799 2HG1 ILE A 237      12.076  -5.032  45.923  1.00  0.00
ATOM   3800  CD  ILE A 237      10.564  -5.345  44.604  1.00  0.00           C
ATOM   3801  HD1 ILE A 237      10.451  -4.372  44.401  1.00  0.00
ATOM   3802  HD2 ILE A 237      11.149  -5.765  43.910  1.00  0.00
ATOM   3803  HD3 ILE A 237       9.670  -5.792  44.601  1.00  0.00
ATOM   3804  C   ILE A 237      10.406  -8.987  47.481  1.00  0.00           C
ATOM   3805  O   ILE A 237      11.157  -9.006  48.453  1.00  0.00           O
ATOM   3806  N   PRO A 238       9.899 -10.137  46.992  1.00  0.00           N
ATOM   3807  CD  PRO A 238       8.978 -10.257  45.862  1.00  0.00           C
ATOM   3808  HD1 PRO A 238       9.437  -9.994  45.013  1.00  0.00
ATOM   3809  HD2 PRO A 238       8.180  -9.672  46.004  1.00  0.00
ATOM   3810  CA  PRO A 238      10.276 -11.438  47.530  1.00  0.00           C
ATOM   3811  HA  PRO A 238       9.884 -11.520  48.446  1.00  0.00
ATOM   3812  CB  PRO A 238       9.746 -12.445  46.492  1.00  0.00           C
ATOM   3813  HB1 PRO A 238      10.443 -12.665  45.809  1.00  0.00
ATOM   3814  HB2 PRO A 238       9.431 -13.287  46.930  1.00  0.00
ATOM   3815  CG  PRO A 238       8.595 -11.723  45.853  1.00  0.00           C
ATOM   3816  HG1 PRO A 238       8.461 -12.046  44.916  1.00  0.00
ATOM   3817  HG2 PRO A 238       7.758 -11.871  46.380  1.00  0.00
ATOM   3818  C   PRO A 238      11.798 -11.603  47.669  1.00  0.00           C
ATOM   3819  O   PRO A 238      12.568 -11.160  46.812  1.00  0.00           O
ATOM   3820  N   ARG A 239      12.192 -12.306  48.732  1.00  0.00           N
ATOM   3821  HN  ARG A 239      11.492 -12.737  49.302  1.00  0.00
ATOM   3822  CA  ARG A 239      13.577 -12.476  49.102  1.00  0.00           C
ATOM   3823  HA  ARG A 239      14.002 -11.574  49.027  1.00  0.00
ATOM   3824  CB  ARG A 239      13.665 -12.957  50.551  1.00  0.00           C
ATOM   3825  HB1 ARG A 239      13.107 -13.782  50.645  1.00  0.00
ATOM   3826  HB2 ARG A 239      14.620 -13.175  50.754  1.00  0.00
ATOM   3827  CG  ARG A 239      13.182 -11.938  51.573  1.00  0.00           C
ATOM   3828  HG1 ARG A 239      12.270 -11.626  51.308  1.00  0.00
ATOM   3829  HG2 ARG A 239      13.136 -12.380  52.469  1.00  0.00
```

```
ATOM   3830  CD  ARG A 239      14.095 -10.730  51.677  1.00  0.00           C
ATOM   3831  HD1 ARG A 239      14.846 -10.935  52.304  1.00  0.00
ATOM   3832  HD2 ARG A 239      14.465 -10.515  50.773  1.00  0.00
ATOM   3833  NE  ARG A 239      13.431  -9.535  52.170  1.00  0.00           N
ATOM   3834  HE  ARG A 239      12.549  -9.647  52.627  1.00  0.00
ATOM   3835  CZ  ARG A 239      13.918  -8.300  52.054  1.00  0.00           C
ATOM   3836  NH1 ARG A 239      15.013  -8.074  51.344  1.00  0.00           N
ATOM   3837 1HH1 ARG A 239      15.480  -8.833  50.890  1.00  0.00
ATOM   3838 2HH1 ARG A 239      15.372  -7.144  51.262  1.00  0.00
ATOM   3839  NH2 ARG A 239      13.306  -7.293  52.649  1.00  0.00           N
ATOM   3840 1HH2 ARG A 239      12.478  -7.458  53.186  1.00  0.00
ATOM   3841 2HH2 ARG A 239      13.670  -6.366  52.563  1.00  0.00
ATOM   3842  C   ARG A 239      14.275 -13.436  48.129  1.00  0.00           C
ATOM   3843  O   ARG A 239      15.489 -13.478  48.110  1.00  0.00           O
ATOM   3844  N   GLU A 240      13.504 -14.170  47.307  1.00  0.00           N
ATOM   3845  HN  GLU A 240      12.512 -14.099  47.416  1.00  0.00
ATOM   3846  CA  GLU A 240      14.018 -15.082  46.246  1.00  0.00           C
ATOM   3847  HA  GLU A 240      14.684 -15.676  46.697  1.00  0.00
ATOM   3848  CB  GLU A 240      12.878 -15.919  45.651  1.00  0.00           C
ATOM   3849  HB1 GLU A 240      12.159 -15.299  45.337  1.00  0.00
ATOM   3850  HB2 GLU A 240      13.236 -16.436  44.873  1.00  0.00
ATOM   3851  CG  GLU A 240      12.257 -16.905  46.627  1.00  0.00           C
ATOM   3852  HG1 GLU A 240      11.767 -17.595  46.094  1.00  0.00
ATOM   3853  HG2 GLU A 240      13.000 -17.341  47.135  1.00  0.00
ATOM   3854  CD  GLU A 240      11.281 -16.311  47.638  1.00  0.00           C
ATOM   3855  OE1 GLU A 240      10.647 -15.297  47.300  1.00  0.00           O
ATOM   3856  OE2 GLU A 240      11.176 -16.856  48.773  1.00  0.00           O
ATOM   3857  C   GLU A 240      14.685 -14.277  45.112  1.00  0.00           C
ATOM   3858  O   GLU A 240      15.484 -14.794  44.357  1.00  0.00           O
ATOM   3859  N   THR A 241      14.282 -13.012  44.991  1.00  0.00           N
ATOM   3860  HN  THR A 241      13.815 -12.591  45.769  1.00  0.00
ATOM   3861  CA  THR A 241      14.482 -12.200  43.787  1.00  0.00           C
ATOM   3862  HA  THR A 241      14.130 -12.737  43.020  1.00  0.00
ATOM   3863  CB  THR A 241      13.687 -10.892  43.896  1.00  0.00           C
ATOM   3864  HB  THR A 241      14.007 -10.347  44.671  1.00  0.00
ATOM   3865  OG1 THR A 241      12.306 -11.206  44.146  1.00  0.00           O
ATOM   3866  HG1 THR A 241      11.779 -10.359  44.219  1.00  0.00
ATOM   3867  CG2 THR A 241      13.817 -10.047  42.650  1.00  0.00           C
ATOM   3868 1HG2 THR A 241      13.286  -9.207  42.760  1.00  0.00
ATOM   3869 2HG2 THR A 241      14.779  -9.817  42.502  1.00  0.00
ATOM   3870 3HG2 THR A 241      13.472 -10.558  41.863  1.00  0.00
ATOM   3871  C   THR A 241      15.977 -11.933  43.564  1.00  0.00           C
ATOM   3872  O   THR A 241      16.664 -11.502  44.456  1.00  0.00           O
ATOM   3873  N   SER A 242      16.447 -12.157  42.339  1.00  0.00           N
ATOM   3874  HN  SER A 242      15.816 -12.497  41.642  1.00  0.00
ATOM   3875  CA  SER A 242      17.857 -11.927  41.958  1.00  0.00           C
ATOM   3876  HA  SER A 242      18.411 -12.491  42.570  1.00  0.00
ATOM   3877  CB  SER A 242      18.147 -12.385  40.559  1.00  0.00           C
ATOM   3878  HB1 SER A 242      19.132 -12.497  40.430  1.00  0.00
ATOM   3879  HB2 SER A 242      17.687 -13.254  40.379  1.00  0.00
ATOM   3880  OG  SER A 242      17.683 -11.435  39.603  1.00  0.00           O
ATOM   3881  HG1 SER A 242      17.888 -11.763  38.681  1.00  0.00
ATOM   3882  C   SER A 242      18.203 -10.452  42.121  1.00  0.00           C
ATOM   3883  O   SER A 242      17.342  -9.579  41.988  1.00  0.00           O
ATOM   3884  N   PRO A 243      19.478 -10.126  42.424  1.00  0.00           N
ATOM   3885  CD  PRO A 243      20.602 -11.065  42.579  1.00  0.00           C
ATOM   3886  HD1 PRO A 243      20.975 -11.320  41.687  1.00  0.00
ATOM   3887  HD2 PRO A 243      20.310 -11.888  43.066  1.00  0.00
ATOM   3888  CA  PRO A 243      19.870  -8.744  42.694  1.00  0.00           C
ATOM   3889  HA  PRO A 243      19.360  -8.407  43.486  1.00  0.00
ATOM   3890  CB  PRO A 243      21.389  -8.840  42.903  1.00  0.00           C
ATOM   3891  HB1 PRO A 243      21.882  -8.688  42.046  1.00  0.00
ATOM   3892  HB2 PRO A 243      21.702  -8.183  43.589  1.00  0.00
ATOM   3893  CG  PRO A 243      21.594 -10.256  43.391  1.00  0.00           C
ATOM   3894  HG1 PRO A 243      22.531 -10.560  43.217  1.00  0.00
ATOM   3895  HG2 PRO A 243      21.397 -10.327  44.369  1.00  0.00
ATOM   3896  C   PRO A 243      19.517  -7.777  41.553  1.00  0.00           C
ATOM   3897  O   PRO A 243      19.065  -6.656  41.803  1.00  0.00           O
ATOM   3898  N   TYR A 244      19.704  -8.229  40.307  1.00  0.00           N
ATOM   3899  HN  TYR A 244      20.000  -9.175  40.174  1.00  0.00
ATOM   3900  CA  TYR A 244      19.491  -7.389  39.142  1.00  0.00           C
```

```
ATOM   3901  HA  TYR A 244      19.962  -6.525  39.321  1.00  0.00
ATOM   3902  CB  TYR A 244      20.124  -8.047  37.919  1.00  0.00           C
ATOM   3903  HB1 TYR A 244      19.893  -9.020  37.933  1.00  0.00
ATOM   3904  HB2 TYR A 244      19.735  -7.624  37.101  1.00  0.00
ATOM   3905  CG  TYR A 244      21.626  -7.934  37.823  1.00  0.00           C
ATOM   3906  CD1 TYR A 244      22.319  -6.861  38.370  1.00  0.00           C
ATOM   3907  HD1 TYR A 244      21.812  -6.139  38.841  1.00  0.00
ATOM   3908  CE1 TYR A 244      23.703  -6.770  38.276  1.00  0.00           C
ATOM   3909  HE1 TYR A 244      24.182  -5.994  38.686  1.00  0.00
ATOM   3910  CZ  TYR A 244      24.422  -7.759  37.611  1.00  0.00           C
ATOM   3911  OH  TYR A 244      25.775  -7.643  37.466  1.00  0.00           O
ATOM   3912  HH  TYR A 244      26.126  -8.436  36.968  1.00  0.00
ATOM   3913  CD2 TYR A 244      22.363  -8.909  37.162  1.00  0.00           C
ATOM   3914  HD2 TYR A 244      21.885  -9.687  36.754  1.00  0.00
ATOM   3915  CE2 TYR A 244      23.747  -8.832  37.052  1.00  0.00           C
ATOM   3916  HE2 TYR A 244      24.253  -9.548  36.571  1.00  0.00
ATOM   3917  C   TYR A 244      17.992  -7.089  38.952  1.00  0.00           C
ATOM   3918  O   TYR A 244      17.623  -5.951  38.699  1.00  0.00           O
ATOM   3919  N   LEU A 245      17.131  -8.102  39.075  1.00  0.00           N
ATOM   3920  HN  LEU A 245      17.476  -9.016  39.287  1.00  0.00
ATOM   3921  CA  LEU A 245      15.691  -7.899  38.905  1.00  0.00           C
ATOM   3922  HA  LEU A 245      15.564  -7.481  38.005  1.00  0.00
ATOM   3923  CB  LEU A 245      14.952  -9.243  38.948  1.00  0.00           C
ATOM   3924  HB1 LEU A 245      15.367  -9.848  38.268  1.00  0.00
ATOM   3925  HB2 LEU A 245      15.072  -9.635  39.860  1.00  0.00
ATOM   3926  CG  LEU A 245      13.456  -9.176  38.662  1.00  0.00           C
ATOM   3927  HG  LEU A 245      13.011  -8.705  39.424  1.00  0.00
ATOM   3928  CD1 LEU A 245      13.180  -8.376  37.403  1.00  0.00           C
ATOM   3929 1HD1 LEU A 245      12.194  -8.346  37.236  1.00  0.00
ATOM   3930 2HD1 LEU A 245      13.527  -7.445  37.517  1.00  0.00
ATOM   3931 3HD1 LEU A 245      13.636  -8.809  36.626  1.00  0.00
ATOM   3932  CD2 LEU A 245      12.866 -10.575  38.557  1.00  0.00           C
ATOM   3933 1HD2 LEU A 245      11.886 -10.510  38.370  1.00  0.00
ATOM   3934 2HD2 LEU A 245      13.315 -11.071  37.814  1.00  0.00
ATOM   3935 3HD2 LEU A 245      13.009 -11.063  39.418  1.00  0.00
ATOM   3936  C   LEU A 245      15.175  -6.960  40.002  1.00  0.00           C
ATOM   3937  O   LEU A 245      14.313  -6.107  39.749  1.00  0.00           O
ATOM   3938  N   ALA A 246      15.704  -7.124  41.218  1.00  0.00           N
ATOM   3939  HN  ALA A 246      16.410  -7.820  41.351  1.00  0.00
ATOM   3940  CA  ALA A 246      15.282  -6.320  42.352  1.00  0.00           C
ATOM   3941  HA  ALA A 246      14.286  -6.383  42.409  1.00  0.00
ATOM   3942  CB  ALA A 246      15.848  -6.880  43.633  1.00  0.00           C
ATOM   3943  HB1 ALA A 246      15.549  -6.317  44.404  1.00  0.00
ATOM   3944  HB2 ALA A 246      15.523  -7.817  43.759  1.00  0.00
ATOM   3945  HB3 ALA A 246      16.847  -6.877  43.585  1.00  0.00
ATOM   3946  C   ALA A 246      15.701  -4.867  42.125  1.00  0.00           C
ATOM   3947  O   ALA A 246      14.931  -3.972  42.350  1.00  0.00           O
ATOM   3948  N   ASN A 247      16.933  -4.658  41.656  1.00  0.00           N
ATOM   3949  HN  ASN A 247      17.530  -5.444  41.498  1.00  0.00
ATOM   3950  CA  ASN A 247      17.440  -3.309  41.364  1.00  0.00           C
ATOM   3951  HA  ASN A 247      17.414  -2.803  42.226  1.00  0.00
ATOM   3952  CB  ASN A 247      18.901  -3.327  40.897  1.00  0.00           C
ATOM   3953  HB1 ASN A 247      19.473  -3.644  41.653  1.00  0.00
ATOM   3954  HB2 ASN A 247      18.982  -3.962  40.129  1.00  0.00
ATOM   3955  CG  ASN A 247      19.414  -1.962  40.447  1.00  0.00           C
ATOM   3956  OD1 ASN A 247      19.199  -1.562  39.306  1.00  0.00           O
ATOM   3957  ND2 ASN A 247      20.102  -1.236  41.324  1.00  0.00           N
ATOM   3958 1HD2 ASN A 247      20.268  -1.589  42.245  1.00  0.00
ATOM   3959 2HD2 ASN A 247      20.453  -0.337  41.063  1.00  0.00
ATOM   3960  C   ASN A 247      16.522  -2.656  40.323  1.00  0.00           C
ATOM   3961  O   ASN A 247      16.150  -1.502  40.454  1.00  0.00           O
ATOM   3962  N   LEU A 248      16.152  -3.434  39.297  1.00  0.00           N
ATOM   3963  HN  LEU A 248      16.469  -4.382  39.279  1.00  0.00
ATOM   3964  CA  LEU A 248      15.319  -2.968  38.220  1.00  0.00           C
ATOM   3965  HA  LEU A 248      15.791  -2.177  37.832  1.00  0.00
ATOM   3966  CB  LEU A 248      15.189  -4.081  37.182  1.00  0.00           C
ATOM   3967  HB1 LEU A 248      16.106  -4.307  36.852  1.00  0.00
ATOM   3968  HB2 LEU A 248      14.790  -4.880  37.632  1.00  0.00
ATOM   3969  CG  LEU A 248      14.324  -3.739  35.974  1.00  0.00           C
ATOM   3970  HG  LEU A 248      13.376  -3.618  36.268  1.00  0.00
ATOM   3971  CD1 LEU A 248      14.783  -2.428  35.324  1.00  0.00           C
```

ATOM	3972	1HD1	LEU	A	248	14.202	-2.225	34.536	1.00	0.00	
ATOM	3973	2HD1	LEU	A	248	14.714	-1.684	35.989	1.00	0.00	
ATOM	3974	3HD1	LEU	A	248	15.732	-2.520	35.023	1.00	0.00	
ATOM	3975	CD2	LEU	A	248	14.368	-4.882	34.992	1.00	0.00	C
ATOM	3976	1HD2	LEU	A	248	13.802	-4.661	34.198	1.00	0.00	
ATOM	3977	2HD2	LEU	A	248	15.312	-5.032	34.697	1.00	0.00	
ATOM	3978	3HD2	LEU	A	248	14.020	-5.711	35.430	1.00	0.00	
ATOM	3979	C	LEU	A	248	13.938	-2.525	38.738	1.00	0.00	C
ATOM	3980	O	LEU	A	248	13.484	-1.441	38.421	1.00	0.00	O
ATOM	3981	N	LEU	A	249	13.279	-3.383	39.522	1.00	0.00	N
ATOM	3982	HN	LEU	A	249	13.711	-4.255	39.752	1.00	0.00	
ATOM	3983	CA	LEU	A	249	11.955	-3.094	40.053	1.00	0.00	C
ATOM	3984	HA	LEU	A	249	11.359	-2.922	39.269	1.00	0.00	
ATOM	3985	CB	LEU	A	249	11.441	-4.302	40.839	1.00	0.00	C
ATOM	3986	HB1	LEU	A	249	12.172	-4.612	41.447	1.00	0.00	
ATOM	3987	HB2	LEU	A	249	10.656	-4.007	41.385	1.00	0.00	
ATOM	3988	CG	LEU	A	249	11.003	-5.488	39.985	1.00	0.00	C
ATOM	3989	HG	LEU	A	249	11.679	-5.618	39.259	1.00	0.00	
ATOM	3990	CD1	LEU	A	249	10.961	-6.769	40.805	1.00	0.00	C
ATOM	3991	1HD1	LEU	A	249	10.672	-7.528	40.222	1.00	0.00	
ATOM	3992	2HD1	LEU	A	249	11.871	-6.959	41.173	1.00	0.00	
ATOM	3993	3HD1	LEU	A	249	10.313	-6.661	41.559	1.00	0.00	
ATOM	3994	CD2	LEU	A	249	9.647	-5.209	39.342	1.00	0.00	C
ATOM	3995	1HD2	LEU	A	249	9.374	-5.994	38.786	1.00	0.00	
ATOM	3996	2HD2	LEU	A	249	8.965	-5.053	40.057	1.00	0.00	
ATOM	3997	3HD2	LEU	A	249	9.714	-4.397	38.762	1.00	0.00	
ATOM	3998	C	LEU	A	249	12.010	-1.846	40.936	1.00	0.00	C
ATOM	3999	O	LEU	A	249	11.089	-1.038	40.886	1.00	0.00	O
ATOM	4000	N	LEU	A	250	13.072	-1.698	41.742	1.00	0.00	N
ATOM	4001	HN	LEU	A	250	13.819	-2.360	41.687	1.00	0.00	
ATOM	4002	CA	LEU	A	250	13.170	-0.588	42.706	1.00	0.00	C
ATOM	4003	HA	LEU	A	250	12.334	-0.622	43.254	1.00	0.00	
ATOM	4004	CB	LEU	A	250	14.381	-0.750	43.634	1.00	0.00	C
ATOM	4005	HB1	LEU	A	250	15.171	-0.981	43.066	1.00	0.00	
ATOM	4006	HB2	LEU	A	250	14.539	0.129	44.084	1.00	0.00	
ATOM	4007	CG	LEU	A	250	14.272	-1.814	44.726	1.00	0.00	C
ATOM	4008	HG	LEU	A	250	14.036	-2.681	44.286	1.00	0.00	
ATOM	4009	CD1	LEU	A	250	15.609	-1.991	45.464	1.00	0.00	C
ATOM	4010	1HD1	LEU	A	250	15.509	-2.691	46.171	1.00	0.00	
ATOM	4011	2HD1	LEU	A	250	16.314	-2.272	44.813	1.00	0.00	
ATOM	4012	3HD1	LEU	A	250	15.873	-1.125	45.888	1.00	0.00	
ATOM	4013	CD2	LEU	A	250	13.159	-1.476	45.702	1.00	0.00	C
ATOM	4014	1HD2	LEU	A	250	13.106	-2.184	46.406	1.00	0.00	
ATOM	4015	2HD2	LEU	A	250	13.349	-0.593	46.131	1.00	0.00	
ATOM	4016	3HD2	LEU	A	250	12.289	-1.428	45.212	1.00	0.00	
ATOM	4017	C	LEU	A	250	13.279	0.733	41.941	1.00	0.00	C
ATOM	4018	O	LEU	A	250	12.757	1.747	42.386	1.00	0.00	O
ATOM	4019	N	GLY	A	251	13.983	0.700	40.805	1.00	0.00	N
ATOM	4020	HN	GLY	A	251	14.326	-0.182	40.482	1.00	0.00	
ATOM	4021	CA	GLY	A	251	14.273	1.886	40.019	1.00	0.00	C
ATOM	4022	HA1	GLY	A	251	14.457	2.635	40.655	1.00	0.00	
ATOM	4023	HA2	GLY	A	251	15.093	1.699	39.477	1.00	0.00	
ATOM	4024	C	GLY	A	251	13.128	2.268	39.098	1.00	0.00	C
ATOM	4025	O	GLY	A	251	13.019	3.426	38.705	1.00	0.00	O
ATOM	4026	N	LEU	A	252	12.310	1.280	38.714	1.00	0.00	N
ATOM	4027	HN	LEU	A	252	12.524	0.343	38.991	1.00	0.00	
ATOM	4028	CA	LEU	A	252	11.120	1.516	37.908	1.00	0.00	C
ATOM	4029	HA	LEU	A	252	11.354	2.223	37.241	1.00	0.00	
ATOM	4030	CB	LEU	A	252	10.731	0.232	37.179	1.00	0.00	C
ATOM	4031	HB1	LEU	A	252	10.700	-0.507	37.852	1.00	0.00	
ATOM	4032	HB2	LEU	A	252	9.821	0.364	36.787	1.00	0.00	
ATOM	4033	CG	LEU	A	252	11.664	-0.200	36.055	1.00	0.00	C
ATOM	4034	HG	LEU	A	252	12.593	-0.242	36.422	1.00	0.00	
ATOM	4035	CD1	LEU	A	252	11.270	-1.573	35.551	1.00	0.00	C
ATOM	4036	1HD1	LEU	A	252	11.888	-1.847	34.814	1.00	0.00	
ATOM	4037	2HD1	LEU	A	252	11.330	-2.233	36.300	1.00	0.00	
ATOM	4038	3HD1	LEU	A	252	10.332	-1.545	35.206	1.00	0.00	
ATOM	4039	CD2	LEU	A	252	11.671	0.800	34.914	1.00	0.00	C
ATOM	4040	1HD2	LEU	A	252	12.293	0.484	34.197	1.00	0.00	
ATOM	4041	2HD2	LEU	A	252	10.747	0.886	34.540	1.00	0.00	
ATOM	4042	3HD2	LEU	A	252	11.978	1.690	35.252	1.00	0.00	

```
ATOM   4043  C   LEU A 252       9.976   1.999  38.810  1.00  0.00           C
ATOM   4044  O   LEU A 252       9.239   2.904  38.432  1.00  0.00           O
ATOM   4045  N   LEU A 253       9.844   1.395  39.993  1.00  0.00           N
ATOM   4046  HN  LEU A 253      10.547   0.748  40.289  1.00  0.00
ATOM   4047  CA  LEU A 253       8.692   1.654  40.876  1.00  0.00           C
ATOM   4048  HA  LEU A 253       7.931   1.944  40.296  1.00  0.00
ATOM   4049  CB  LEU A 253       8.308   0.369  41.616  1.00  0.00           C
ATOM   4050  HB1 LEU A 253       9.131   0.000  42.048  1.00  0.00
ATOM   4051  HB2 LEU A 253       7.639   0.604  42.321  1.00  0.00
ATOM   4052  CG  LEU A 253       7.706  -0.727  40.749  1.00  0.00           C
ATOM   4053  HG  LEU A 253       8.364  -0.954  40.031  1.00  0.00
ATOM   4054  CD1 LEU A 253       7.450  -1.989  41.567  1.00  0.00           C
ATOM   4055 1HD1 LEU A 253       7.055  -2.694  40.978  1.00  0.00
ATOM   4056 2HD1 LEU A 253       8.313  -2.319  41.949  1.00  0.00
ATOM   4057 3HD1 LEU A 253       6.814  -1.781  42.310  1.00  0.00
ATOM   4058  CD2 LEU A 253       6.418  -0.236  40.092  1.00  0.00           C
ATOM   4059 1HD2 LEU A 253       6.034  -0.965  39.526  1.00  0.00
ATOM   4060 2HD2 LEU A 253       5.759   0.019  40.800  1.00  0.00
ATOM   4061 3HD2 LEU A 253       6.618   0.560  39.520  1.00  0.00
ATOM   4062  C   LEU A 253       9.032   2.776  41.863  1.00  0.00           C
ATOM   4063  O   LEU A 253       8.900   2.604  43.069  1.00  0.00           O
ATOM   4064  N   GLN A 254       9.433   3.931  41.311  1.00  0.00           N
ATOM   4065  HN  GLN A 254       9.604   3.937  40.326  1.00  0.00
ATOM   4066  CA  GLN A 254       9.637   5.172  42.035  1.00  0.00           C
ATOM   4067  HA  GLN A 254       9.701   4.931  43.003  1.00  0.00
ATOM   4068  CB  GLN A 254      10.927   5.842  41.571  1.00  0.00           C
ATOM   4069  HB1 GLN A 254      10.895   5.940  40.576  1.00  0.00
ATOM   4070  HB2 GLN A 254      10.985   6.747  41.993  1.00  0.00
ATOM   4071  CG  GLN A 254      12.180   5.063  41.935  1.00  0.00           C
ATOM   4072  HG1 GLN A 254      12.038   4.101  41.702  1.00  0.00
ATOM   4073  HG2 GLN A 254      12.947   5.424  41.404  1.00  0.00
ATOM   4074  CD  GLN A 254      12.529   5.154  43.404  1.00  0.00           C
ATOM   4075  OE1 GLN A 254      12.230   6.138  44.068  1.00  0.00           O
ATOM   4076  NE2 GLN A 254      13.169   4.119  43.918  1.00  0.00           N
ATOM   4077 1HE2 GLN A 254      13.395   3.334  43.341  1.00  0.00
ATOM   4078 2HE2 GLN A 254      13.427   4.120  44.884  1.00  0.00
ATOM   4079  C   GLN A 254       8.455   6.103  41.777  1.00  0.00           C
ATOM   4080  O   GLN A 254       8.200   6.472  40.629  1.00  0.00           O
ATOM   4081  N   ARG A 255       7.772   6.502  42.856  1.00  0.00           N
ATOM   4082  HN  ARG A 255       8.110   6.228  43.756  1.00  0.00
ATOM   4083  CA  ARG A 255       6.561   7.315  42.795  1.00  0.00           C
ATOM   4084  HA  ARG A 255       5.893   6.802  42.256  1.00  0.00
ATOM   4085  CB  ARG A 255       5.986   7.529  44.201  1.00  0.00           C
ATOM   4086  HB1 ARG A 255       5.910   6.638  44.648  1.00  0.00
ATOM   4087  HB2 ARG A 255       6.620   8.107  44.715  1.00  0.00
ATOM   4088  CG  ARG A 255       4.611   8.192  44.230  1.00  0.00           C
ATOM   4089  HG1 ARG A 255       4.086   7.870  43.442  1.00  0.00
ATOM   4090  HG2 ARG A 255       4.146   7.923  45.073  1.00  0.00
ATOM   4091  CD  ARG A 255       4.671   9.712  44.178  1.00  0.00           C
ATOM   4092  HD1 ARG A 255       5.577  10.016  44.473  1.00  0.00
ATOM   4093  HD2 ARG A 255       4.507  10.016  43.240  1.00  0.00
ATOM   4094  NE  ARG A 255       3.694  10.382  45.026  1.00  0.00           N
ATOM   4095  HE  ARG A 255       2.966   9.824  45.425  1.00  0.00
ATOM   4096  CZ  ARG A 255       3.700  11.688  45.309  1.00  0.00           C
ATOM   4097  NH1 ARG A 255       4.685  12.466  44.888  1.00  0.00           N
ATOM   4098 1HH1 ARG A 255       5.435  12.078  44.352  1.00  0.00
ATOM   4099 2HH1 ARG A 255       4.679  13.442  45.106  1.00  0.00
ATOM   4100  NH2 ARG A 255       2.710  12.214  46.011  1.00  0.00           N
ATOM   4101 1HH2 ARG A 255       1.959  11.635  46.329  1.00  0.00
ATOM   4102 2HH2 ARG A 255       2.712  13.191  46.224  1.00  0.00
ATOM   4103  C   ARG A 255       6.867   8.638  42.088  1.00  0.00           C
ATOM   4104  O   ARG A 255       6.031   9.160  41.376  1.00  0.00           O
ATOM   4105  N   ASN A 256       8.071   9.166  42.283  1.00  0.00           N
ATOM   4106  HN  ASN A 256       8.732   8.670  42.846  1.00  0.00
ATOM   4107  CA  ASN A 256       8.458  10.444  41.702  1.00  0.00           C
ATOM   4108  HA  ASN A 256       7.612  10.903  41.430  1.00  0.00
ATOM   4109  CB  ASN A 256       9.151  11.326  42.740  1.00  0.00           C
ATOM   4110  HB1 ASN A 256       8.638  11.272  43.596  1.00  0.00
ATOM   4111  HB2 ASN A 256      10.078  10.979  42.884  1.00  0.00
ATOM   4112  CG  ASN A 256       9.252  12.783  42.344  1.00  0.00           C
ATOM   4113  OD1 ASN A 256       8.947  13.161  41.211  1.00  0.00           O
```

```
ATOM   4114  ND2 ASN A 256       9.680  13.610  43.279  1.00  0.00           N
ATOM   4115 1HD2 ASN A 256       9.915  13.261  44.186  1.00  0.00
ATOM   4116 2HD2 ASN A 256       9.769  14.586  43.081  1.00  0.00
ATOM   4117  C   ASN A 256       9.329  10.201  40.461  1.00  0.00           C
ATOM   4118  O   ASN A 256      10.352   9.512  40.526  1.00  0.00           O
ATOM   4119  N   GLN A 257       8.899  10.789  39.335  1.00  0.00           N
ATOM   4120  HN  GLN A 257       8.088  11.371  39.388  1.00  0.00
ATOM   4121  CA  GLN A 257       9.551  10.628  38.027  1.00  0.00           C
ATOM   4122  HA  GLN A 257       9.604   9.648  37.834  1.00  0.00
ATOM   4123  CB  GLN A 257       8.702  11.276  36.920  1.00  0.00           C
ATOM   4124  HB1 GLN A 257       9.194  11.177  36.055  1.00  0.00
ATOM   4125  HB2 GLN A 257       7.834  10.782  36.868  1.00  0.00
ATOM   4126  CG  GLN A 257       8.385  12.763  37.111  1.00  0.00           C
ATOM   4127  HG1 GLN A 257       9.066  13.155  37.729  1.00  0.00
ATOM   4128  HG2 GLN A 257       8.443  13.216  36.221  1.00  0.00
ATOM   4129  CD  GLN A 257       7.003  13.033  37.693  1.00  0.00           C
ATOM   4130  OE1 GLN A 257       6.613  12.476  38.719  1.00  0.00           O
ATOM   4131  NE2 GLN A 257       6.244  13.917  37.054  1.00  0.00           N
ATOM   4132 1HE2 GLN A 257       6.588  14.370  36.231  1.00  0.00
ATOM   4133 2HE2 GLN A 257       5.329  14.130  37.396  1.00  0.00
ATOM   4134  C   GLN A 257      10.978  11.194  38.097  1.00  0.00           C
ATOM   4135  O   GLN A 257      11.902  10.715  37.431  1.00  0.00           O
ATOM   4136  N   LYS A 258      11.138  12.215  38.935  1.00  0.00           N
ATOM   4137  HN  LYS A 258      10.316  12.617  39.337  1.00  0.00
ATOM   4138  CA  LYS A 258      12.421  12.789  39.309  1.00  0.00           C
ATOM   4139  HA  LYS A 258      12.819  13.239  38.510  1.00  0.00
ATOM   4140  CB  LYS A 258      12.150  13.833  40.403  1.00  0.00           C
ATOM   4141  HB1 LYS A 258      11.522  14.516  40.030  1.00  0.00
ATOM   4142  HB2 LYS A 258      11.716  13.367  41.174  1.00  0.00
ATOM   4143  CG  LYS A 258      13.364  14.574  40.941  1.00  0.00           C
ATOM   4144  HG1 LYS A 258      13.883  13.948  41.524  1.00  0.00
ATOM   4145  HG2 LYS A 258      13.931  14.855  40.167  1.00  0.00
ATOM   4146  CD  LYS A 258      13.004  15.814  41.757  1.00  0.00           C
ATOM   4147  HD1 LYS A 258      12.695  16.531  41.132  1.00  0.00
ATOM   4148  HD2 LYS A 258      12.264  15.580  42.387  1.00  0.00
ATOM   4149  CE  LYS A 258      14.164  16.355  42.566  1.00  0.00           C
ATOM   4150  HE1 LYS A 258      14.996  16.321  42.012  1.00  0.00
ATOM   4151  HE2 LYS A 258      13.973  17.302  42.822  1.00  0.00
ATOM   4152  NZ  LYS A 258      14.396  15.573  43.806  1.00  0.00           N
ATOM   4153  HZ1 LYS A 258      15.168  15.964  44.307  1.00  0.00
ATOM   4154  HZ2 LYS A 258      13.577  15.605  44.378  1.00  0.00
ATOM   4155  HZ3 LYS A 258      14.600  14.623  43.568  1.00  0.00
ATOM   4156  C   LYS A 258      13.407  11.676  39.730  1.00  0.00           C
ATOM   4157  O   LYS A 258      14.569  11.710  39.349  1.00  0.00           O
ATOM   4158  N   ASP A 259      12.930  10.677  40.482  1.00  0.00           N
ATOM   4159  HN  ASP A 259      11.942  10.622  40.625  1.00  0.00
ATOM   4160  CA  ASP A 259      13.796   9.639  41.120  1.00  0.00           C
ATOM   4161  HA  ASP A 259      14.711  10.042  41.109  1.00  0.00
ATOM   4162  CB  ASP A 259      13.336   9.350  42.548  1.00  0.00           C
ATOM   4163  HB1 ASP A 259      12.366   9.107  42.521  1.00  0.00
ATOM   4164  HB2 ASP A 259      13.864   8.576  42.896  1.00  0.00
ATOM   4165  CG  ASP A 259      13.504  10.508  43.518  1.00  0.00           C
ATOM   4166  OD1 ASP A 259      14.545  11.215  43.448  1.00  0.00           O
ATOM   4167  OD2 ASP A 259      12.617  10.668  44.368  1.00  0.00           O
ATOM   4168  C   ASP A 259      13.786   8.302  40.346  1.00  0.00           C
ATOM   4169  O   ASP A 259      14.480   7.373  40.712  1.00  0.00           O
ATOM   4170  N   ARG A 260      12.978   8.206  39.289  1.00  0.00           N
ATOM   4171  HN  ARG A 260      12.502   9.028  38.976  1.00  0.00
ATOM   4172  CA  ARG A 260      12.758   6.965  38.574  1.00  0.00           C
ATOM   4173  HA  ARG A 260      12.787   6.229  39.250  1.00  0.00
ATOM   4174  CB  ARG A 260      11.381   7.005  37.908  1.00  0.00           C
ATOM   4175  HB1 ARG A 260      10.713   7.304  38.589  1.00  0.00
ATOM   4176  HB2 ARG A 260      11.414   7.668  37.160  1.00  0.00
ATOM   4177  CG  ARG A 260      10.908   5.676  37.338  1.00  0.00           C
ATOM   4178  HG1 ARG A 260      11.539   5.385  36.618  1.00  0.00
ATOM   4179  HG2 ARG A 260      10.896   4.992  38.068  1.00  0.00
ATOM   4180  CD  ARG A 260       9.509   5.805  36.753  1.00  0.00           C
ATOM   4181  HD1 ARG A 260       9.528   6.426  35.969  1.00  0.00
ATOM   4182  HD2 ARG A 260       9.184   4.906  36.458  1.00  0.00
ATOM   4183  NE  ARG A 260       8.546   6.330  37.707  1.00  0.00           N
ATOM   4184  HE  ARG A 260       8.683   6.105  38.672  1.00  0.00
```

```
ATOM   4185  CZ  ARG A 260       7.496   7.086  37.397  1.00  0.00           C
ATOM   4186  NH1 ARG A 260       7.202   7.364  36.138  1.00  0.00           N
ATOM   4187 1HH1 ARG A 260       7.773   7.004  35.400  1.00  0.00
ATOM   4188 2HH1 ARG A 260       6.409   7.934  35.924  1.00  0.00
ATOM   4189  NH2 ARG A 260       6.725   7.550  38.358  1.00  0.00           N
ATOM   4190 1HH2 ARG A 260       6.931   7.334  39.312  1.00  0.00
ATOM   4191 2HH2 ARG A 260       5.934   8.119  38.134  1.00  0.00
ATOM   4192  C   ARG A 260      13.873   6.756  37.540  1.00  0.00           C
ATOM   4193  O   ARG A 260      14.279   7.666  36.852  1.00  0.00           O
ATOM   4194  N   MET A 261      14.349   5.521  37.449  1.00  0.00           N
ATOM   4195  HN  MET A 261      14.046   4.849  38.125  1.00  0.00
ATOM   4196  CA  MET A 261      15.285   5.086  36.426  1.00  0.00           C
ATOM   4197  HA  MET A 261      16.194   5.406  36.692  1.00  0.00
ATOM   4198  CB  MET A 261      15.244   3.562  36.327  1.00  0.00           C
ATOM   4199  HB1 MET A 261      15.457   3.181  37.227  1.00  0.00
ATOM   4200  HB2 MET A 261      14.320   3.288  36.059  1.00  0.00
ATOM   4201  CG  MET A 261      16.216   2.971  35.327  1.00  0.00           C
ATOM   4202  HG1 MET A 261      16.017   3.423  34.457  1.00  0.00
ATOM   4203  HG2 MET A 261      17.126   3.242  35.642  1.00  0.00
ATOM   4204  SD  MET A 261      16.017   1.189  35.241  1.00  0.00           S
ATOM   4205  CE  MET A 261      16.356   0.743  36.941  1.00  0.00           C
ATOM   4206  HE1 MET A 261      16.280  -0.248  37.047  1.00  0.00
ATOM   4207  HE2 MET A 261      17.281   1.034  37.184  1.00  0.00
ATOM   4208  HE3 MET A 261      15.696   1.194  37.542  1.00  0.00
ATOM   4209  C   MET A 261      14.865   5.683  35.093  1.00  0.00           C
ATOM   4210  O   MET A 261      13.687   5.640  34.767  1.00  0.00           O
ATOM   4211  N   ASP A 262      15.813   6.243  34.337  1.00  0.00           N
ATOM   4212  HN  ASP A 262      16.750   6.294  34.683  1.00  0.00
ATOM   4213  CA  ASP A 262      15.503   6.792  32.991  1.00  0.00           C
ATOM   4214  HA  ASP A 262      14.529   7.020  32.977  1.00  0.00
ATOM   4215  CB  ASP A 262      16.250   8.104  32.733  1.00  0.00           C
ATOM   4216  HB1 ASP A 262      16.008   8.434  31.821  1.00  0.00
ATOM   4217  HB2 ASP A 262      15.962   8.774  33.417  1.00  0.00
ATOM   4218  CG  ASP A 262      17.761   7.975  32.804  1.00  0.00           C
ATOM   4219  OD1 ASP A 262      18.274   6.931  32.332  1.00  0.00           O
ATOM   4220  OD2 ASP A 262      18.416   8.925  33.341  1.00  0.00           O
ATOM   4221  C   ASP A 262      15.769   5.719  31.922  1.00  0.00           C
ATOM   4222  O   ASP A 262      16.310   4.655  32.208  1.00  0.00           O
ATOM   4223  N   PHE A 263      15.354   6.014  30.691  1.00  0.00           N
ATOM   4224  HN  PHE A 263      15.135   6.965  30.475  1.00  0.00
ATOM   4225  CA  PHE A 263      15.206   5.010  29.646  1.00  0.00           C
ATOM   4226  HA  PHE A 263      14.570   4.332  30.013  1.00  0.00
ATOM   4227  CB  PHE A 263      14.600   5.626  28.389  1.00  0.00           C
ATOM   4228  HB1 PHE A 263      15.031   6.514  28.229  1.00  0.00
ATOM   4229  HB2 PHE A 263      14.786   5.020  27.615  1.00  0.00
ATOM   4230  CG  PHE A 263      13.111   5.838  28.469  1.00  0.00           C
ATOM   4231  CD1 PHE A 263      12.230   4.790  28.237  1.00  0.00           C
ATOM   4232  HD1 PHE A 263      12.588   3.882  28.019  1.00  0.00
ATOM   4233  CE1 PHE A 263      10.855   4.989  28.305  1.00  0.00           C
ATOM   4234  HE1 PHE A 263      10.232   4.228  28.124  1.00  0.00
ATOM   4235  CZ  PHE A 263      10.348   6.230  28.621  1.00  0.00           C
ATOM   4236  HZ  PHE A 263       9.359   6.371  28.674  1.00  0.00
ATOM   4237  CD2 PHE A 263      12.584   7.082  28.799  1.00  0.00           C
ATOM   4238  HD2 PHE A 263      13.201   7.845  28.992  1.00  0.00
ATOM   4239  CE2 PHE A 263      11.211   7.275  28.863  1.00  0.00           C
ATOM   4240  HE2 PHE A 263      10.846   8.179  29.086  1.00  0.00
ATOM   4241  C   PHE A 263      16.541   4.325  29.352  1.00  0.00           C
ATOM   4242  O   PHE A 263      16.600   3.117  29.200  1.00  0.00           O
ATOM   4243  N   GLU A 264      17.615   5.113  29.285  1.00  0.00           N
ATOM   4244  HN  GLU A 264      17.532   6.075  29.545  1.00  0.00
ATOM   4245  CA  GLU A 264      18.912   4.603  28.840  1.00  0.00           C
ATOM   4246  HA  GLU A 264      18.782   4.105  27.983  1.00  0.00
ATOM   4247  CB  GLU A 264      19.844   5.782  28.544  1.00  0.00           C
ATOM   4248  HB1 GLU A 264      19.936   6.335  29.372  1.00  0.00
ATOM   4249  HB2 GLU A 264      20.740   5.425  28.281  1.00  0.00
ATOM   4250  CG  GLU A 264      19.320   6.673  27.415  1.00  0.00           C
ATOM   4251  HG1 GLU A 264      18.417   7.007  27.684  1.00  0.00
ATOM   4252  HG2 GLU A 264      19.947   7.446  27.321  1.00  0.00
ATOM   4253  CD  GLU A 264      19.186   6.003  26.040  1.00  0.00           C
ATOM   4254  OE1 GLU A 264      19.805   4.918  25.834  1.00  0.00           O
ATOM   4255  OE2 GLU A 264      18.467   6.566  25.158  1.00  0.00           O
```

```
ATOM   4256  C   GLU A 264      19.457   3.601  29.874  1.00  0.00           C
ATOM   4257  O   GLU A 264      20.071   2.601  29.504  1.00  0.00           O
ATOM   4258  N   ALA A 265      19.190   3.852  31.159  1.00  0.00           N
ATOM   4259  HN  ALA A 265      18.738   4.712  31.397  1.00  0.00
ATOM   4260  CA  ALA A 265      19.531   2.923  32.226  1.00  0.00           C
ATOM   4261  HA  ALA A 265      20.514   2.752  32.161  1.00  0.00
ATOM   4262  CB  ALA A 265      19.235   3.547  33.567  1.00  0.00           C
ATOM   4263  HB1 ALA A 265      19.472   2.903  34.294  1.00  0.00
ATOM   4264  HB2 ALA A 265      19.775   4.382  33.673  1.00  0.00
ATOM   4265  HB3 ALA A 265      18.262   3.770  33.623  1.00  0.00
ATOM   4266  C   ALA A 265      18.751   1.616  32.045  1.00  0.00           C
ATOM   4267  O   ALA A 265      19.303   0.536  32.223  1.00  0.00           O
ATOM   4268  N   PHE A 266      17.460   1.746  31.700  1.00  0.00           N
ATOM   4269  HN  PHE A 266      17.109   2.668  31.537  1.00  0.00
ATOM   4270  CA  PHE A 266      16.532   0.622  31.547  1.00  0.00           C
ATOM   4271  HA  PHE A 266      16.622   0.076  32.380  1.00  0.00
ATOM   4272  CB  PHE A 266      15.087   1.127  31.442  1.00  0.00           C
ATOM   4273  HB1 PHE A 266      14.781   1.406  32.352  1.00  0.00
ATOM   4274  HB2 PHE A 266      15.070   1.916  30.828  1.00  0.00
ATOM   4275  CG  PHE A 266      14.101   0.113  30.916  1.00  0.00           C
ATOM   4276  CD1 PHE A 266      13.781  -1.024  31.641  1.00  0.00           C
ATOM   4277  HD1 PHE A 266      14.175  -1.154  32.551  1.00  0.00
ATOM   4278  CE1 PHE A 266      12.925  -1.984  31.126  1.00  0.00           C
ATOM   4279  HE1 PHE A 266      12.745  -2.822  31.641  1.00  0.00
ATOM   4280  CZ  PHE A 266      12.328  -1.783  29.914  1.00  0.00           C
ATOM   4281  HZ  PHE A 266      11.671  -2.449  29.561  1.00  0.00
ATOM   4282  CD2 PHE A 266      13.526   0.275  29.675  1.00  0.00           C
ATOM   4283  HD2 PHE A 266      13.756   1.077  29.124  1.00  0.00
ATOM   4284  CE2 PHE A 266      12.634  -0.661  29.183  1.00  0.00           C
ATOM   4285  HE2 PHE A 266      12.209  -0.520  28.289  1.00  0.00
ATOM   4286  C   PHE A 266      16.924  -0.245  30.337  1.00  0.00           C
ATOM   4287  O   PHE A 266      17.027  -1.457  30.473  1.00  0.00           O
ATOM   4288  N   PHE A 267      17.131   0.374  29.167  1.00  0.00           N
ATOM   4289  HN  PHE A 267      17.075   1.372  29.137  1.00  0.00
ATOM   4290  CA  PHE A 267      17.442  -0.363  27.906  1.00  0.00           C
ATOM   4291  HA  PHE A 267      16.644  -0.947  27.755  1.00  0.00
ATOM   4292  CB  PHE A 267      17.624   0.568  26.700  1.00  0.00           C
ATOM   4293  HB1 PHE A 267      18.346   1.226  26.913  1.00  0.00
ATOM   4294  HB2 PHE A 267      17.897   0.017  25.912  1.00  0.00
ATOM   4295  CG  PHE A 267      16.403   1.349  26.304  1.00  0.00           C
ATOM   4296  CD1 PHE A 267      15.140   0.774  26.346  1.00  0.00           C
ATOM   4297  HD1 PHE A 267      15.041  -0.191  26.591  1.00  0.00
ATOM   4298  CE1 PHE A 267      14.020   1.526  26.052  1.00  0.00           C
ATOM   4299  HE1 PHE A 267      13.112   1.113  26.117  1.00  0.00
ATOM   4300  CZ  PHE A 267      14.148   2.840  25.671  1.00  0.00           C
ATOM   4301  HZ  PHE A 267      13.332   3.378  25.458  1.00  0.00
ATOM   4302  CD2 PHE A 267      16.512   2.681  25.924  1.00  0.00           C
ATOM   4303  HD2 PHE A 267      17.413   3.114  25.897  1.00  0.00
ATOM   4304  CE2 PHE A 267      15.388   3.418  25.581  1.00  0.00           C
ATOM   4305  HE2 PHE A 267      15.481   4.364  25.270  1.00  0.00
ATOM   4306  C   PHE A 267      18.715  -1.194  28.067  1.00  0.00           C
ATOM   4307  O   PHE A 267      18.836  -2.257  27.479  1.00  0.00           O
ATOM   4308  N   SER A 268      19.655  -0.675  28.855  1.00  0.00           N
ATOM   4309  HN  SER A 268      19.437   0.146  29.383  1.00  0.00
ATOM   4310  CA  SER A 268      20.976  -1.246  28.980  1.00  0.00           C
ATOM   4311  HA  SER A 268      21.044  -1.952  28.275  1.00  0.00
ATOM   4312  CB  SER A 268      22.024  -0.190  28.717  1.00  0.00           C
ATOM   4313  HB1 SER A 268      22.925  -0.611  28.609  1.00  0.00
ATOM   4314  HB2 SER A 268      21.798   0.330  27.893  1.00  0.00
ATOM   4315  OG  SER A 268      22.093   0.722  29.807  1.00  0.00           O
ATOM   4316  HG1 SER A 268      22.790   1.414  29.619  1.00  0.00
ATOM   4317  C   SER A 268      21.153  -1.872  30.368  1.00  0.00           C
ATOM   4318  O   SER A 268      22.283  -2.040  30.834  1.00  0.00           O
ATOM   4319  N   HIS A 269      20.036  -2.226  31.019  1.00  0.00           N
ATOM   4320  HN  HIS A 269      19.159  -2.173  30.541  1.00  0.00
ATOM   4321  CA  HIS A 269      20.061  -2.688  32.409  1.00  0.00           C
ATOM   4322  HA  HIS A 269      20.638  -2.038  32.903  1.00  0.00
ATOM   4323  CB  HIS A 269      18.666  -2.671  33.058  1.00  0.00           C
ATOM   4324  HB1 HIS A 269      18.224  -1.794  32.870  1.00  0.00
ATOM   4325  HB2 HIS A 269      18.114  -3.411  32.675  1.00  0.00
ATOM   4326  ND1 HIS A 269      18.899  -4.097  35.125  1.00  0.00           N
```

```
ATOM   4327  CG  HIS A 269      18.716  -2.853  34.538  1.00  0.00            C
ATOM   4328  CE1 HIS A 269      18.947  -3.951  36.434  1.00  0.00            C
ATOM   4329  HE1 HIS A 269      19.071  -4.687  37.100  1.00  0.00
ATOM   4330  NE2 HIS A 269      18.802  -2.648  36.723  1.00  0.00            N
ATOM   4331  HE2 HIS A 269      18.792  -2.251  37.641  1.00  0.00
ATOM   4332  CD2 HIS A 269      18.670  -1.956  35.554  1.00  0.00            C
ATOM   4333  HD2 HIS A 269      18.559  -0.966  35.463  1.00  0.00
ATOM   4334  C   HIS A 269      20.680  -4.070  32.464  1.00  0.00            C
ATOM   4335  O   HIS A 269      20.353  -4.925  31.650  1.00  0.00            O
ATOM   4336  N   PRO A 270      21.595  -4.318  33.426  1.00  0.00            N
ATOM   4337  CD  PRO A 270      22.114  -3.317  34.372  1.00  0.00            C
ATOM   4338  HD1 PRO A 270      21.373  -2.968  34.946  1.00  0.00
ATOM   4339  HD2 PRO A 270      22.536  -2.559  33.875  1.00  0.00
ATOM   4340  CA  PRO A 270      22.231  -5.623  33.586  1.00  0.00            C
ATOM   4341  HA  PRO A 270      22.875  -5.742  32.831  1.00  0.00
ATOM   4342  CB  PRO A 270      22.803  -5.519  34.998  1.00  0.00            C
ATOM   4343  HB1 PRO A 270      22.128  -5.812  35.675  1.00  0.00
ATOM   4344  HB2 PRO A 270      23.628  -6.077  35.087  1.00  0.00
ATOM   4345  CG  PRO A 270      23.138  -4.067  35.197  1.00  0.00            C
ATOM   4346  HG1 PRO A 270      23.064  -3.820  36.163  1.00  0.00
ATOM   4347  HG2 PRO A 270      24.064  -3.874  34.873  1.00  0.00
ATOM   4348  C   PRO A 270      21.301  -6.848  33.499  1.00  0.00            C
ATOM   4349  O   PRO A 270      21.692  -7.880  32.928  1.00  0.00            O
ATOM   4350  N   PHE A 271      20.084  -6.727  34.047  1.00  0.00            N
ATOM   4351  HN  PHE A 271      19.818  -5.847  34.440  1.00  0.00
ATOM   4352  CA  PHE A 271      19.133  -7.834  34.091  1.00  0.00            C
ATOM   4353  HA  PHE A 271      19.611  -8.546  34.605  1.00  0.00
ATOM   4354  CB  PHE A 271      17.851  -7.413  34.807  1.00  0.00            C
ATOM   4355  HB1 PHE A 271      18.067  -7.198  35.759  1.00  0.00
ATOM   4356  HB2 PHE A 271      17.480  -6.600  34.358  1.00  0.00
ATOM   4357  CG  PHE A 271      16.793  -8.477  34.796  1.00  0.00            C
ATOM   4358  CD1 PHE A 271      16.944  -9.636  35.550  1.00  0.00            C
ATOM   4359  HD1 PHE A 271      17.755  -9.748  36.124  1.00  0.00
ATOM   4360  CE1 PHE A 271      15.988 -10.636  35.517  1.00  0.00            C
ATOM   4361  HE1 PHE A 271      16.105 -11.461  36.070  1.00  0.00
ATOM   4362  CZ  PHE A 271      14.881 -10.492  34.724  1.00  0.00            C
ATOM   4363  HZ  PHE A 271      14.180 -11.205  34.707  1.00  0.00
ATOM   4364  CD2 PHE A 271      15.693  -8.364  33.970  1.00  0.00            C
ATOM   4365  HD2 PHE A 271      15.589  -7.559  33.386  1.00  0.00
ATOM   4366  CE2 PHE A 271      14.734  -9.359  33.947  1.00  0.00            C
ATOM   4367  HE2 PHE A 271      13.928  -9.258  33.364  1.00  0.00
ATOM   4368  C   PHE A 271      18.775  -8.336  32.678  1.00  0.00            C
ATOM   4369  O   PHE A 271      18.373  -9.480  32.525  1.00  0.00            O
ATOM   4370  N   LEU A 272      18.898  -7.466  31.668  1.00  0.00            N
ATOM   4371  HN  LEU A 272      19.361  -6.596  31.836  1.00  0.00
ATOM   4372  CA  LEU A 272      18.381  -7.741  30.329  1.00  0.00            C
ATOM   4373  HA  LEU A 272      17.591  -8.340  30.463  1.00  0.00
ATOM   4374  CB  LEU A 272      17.983  -6.422  29.666  1.00  0.00            C
ATOM   4375  HB1 LEU A 272      18.682  -5.743  29.890  1.00  0.00
ATOM   4376  HB2 LEU A 272      17.966  -6.568  28.677  1.00  0.00
ATOM   4377  CG  LEU A 272      16.635  -5.846  30.069  1.00  0.00            C
ATOM   4378  HG  LEU A 272      16.618  -5.711  31.060  1.00  0.00
ATOM   4379  CD1 LEU A 272      16.428  -4.504  29.400  1.00  0.00            C
ATOM   4380 1HD1 LEU A 272      15.540  -4.131  29.669  1.00  0.00
ATOM   4381 2HD1 LEU A 272      17.153  -3.876  29.682  1.00  0.00
ATOM   4382 3HD1 LEU A 272      16.454  -4.619  28.407  1.00  0.00
ATOM   4383  CD2 LEU A 272      15.519  -6.806  29.716  1.00  0.00            C
ATOM   4384 1HD2 LEU A 272      14.641  -6.411  29.988  1.00  0.00
ATOM   4385 2HD2 LEU A 272      15.520  -6.970  28.729  1.00  0.00
ATOM   4386 3HD2 LEU A 272      15.659  -7.671  30.198  1.00  0.00
ATOM   4387  C   LEU A 272      19.426  -8.452  29.456  1.00  0.00            C
ATOM   4388  O   LEU A 272      19.105  -8.808  28.317  1.00  0.00            O
ATOM   4389  N   GLU A 273      20.666  -8.622  29.946  1.00  0.00            N
ATOM   4390  HN  GLU A 273      20.831  -8.464  30.919  1.00  0.00
ATOM   4391  CA  GLU A 273      21.785  -9.035  29.087  1.00  0.00            C
ATOM   4392  HA  GLU A 273      21.366  -9.467  28.288  1.00  0.00
ATOM   4393  CB  GLU A 273      22.610  -7.818  28.649  1.00  0.00            C
ATOM   4394  HB1 GLU A 273      23.410  -8.139  28.141  1.00  0.00
ATOM   4395  HB2 GLU A 273      22.045  -7.245  28.054  1.00  0.00
ATOM   4396  CG  GLU A 273      23.095  -6.965  29.805  1.00  0.00            C
ATOM   4397  HG1 GLU A 273      22.319  -6.791  30.411  1.00  0.00
```

```
ATOM   4398  HG2 GLU A 273      23.795  -7.480  30.299  1.00  0.00
ATOM   4399  CD  GLU A 273      23.688  -5.627  29.397  1.00  0.00           C
ATOM   4400  OE1 GLU A 273      23.451  -5.197  28.244  1.00  0.00           O
ATOM   4401  OE2 GLU A 273      24.408  -5.030  30.221  1.00  0.00           O
ATOM   4402  C   GLU A 273      22.676 -10.049  29.804  1.00  0.00           C
ATOM   4403  O   GLU A 273      23.847  -9.755  30.071  1.00  0.00           O
ATOM   4404  N   GLN A 274      22.161 -11.258  30.057  1.00  0.00           N
ATOM   4405  HN  GLN A 274      21.225 -11.470  29.776  1.00  0.00
ATOM   4406  CA  GLN A 274      22.975 -12.283  30.752  1.00  0.00           C
ATOM   4407  HA  GLN A 274      23.907 -12.204  30.399  1.00  0.00
ATOM   4408  CB  GLN A 274      22.973 -12.001  32.254  1.00  0.00           C
ATOM   4409  HB1 GLN A 274      23.414 -12.767  32.721  1.00  0.00
ATOM   4410  HB2 GLN A 274      23.495 -11.164  32.420  1.00  0.00
ATOM   4411  CG  GLN A 274      21.583 -11.820  32.844  1.00  0.00           C
ATOM   4412  HG1 GLN A 274      21.081 -11.146  32.303  1.00  0.00
ATOM   4413  HG2 GLN A 274      21.099 -12.694  32.820  1.00  0.00
ATOM   4414  CD  GLN A 274      21.657 -11.342  34.272  1.00  0.00           C
ATOM   4415  OE1 GLN A 274      20.967 -11.841  35.159  1.00  0.00           O
ATOM   4416  NE2 GLN A 274      22.523 -10.373  34.508  1.00  0.00           N
ATOM   4417 1HE2 GLN A 274      23.077 -10.005  33.761  1.00  0.00
ATOM   4418 2HE2 GLN A 274      22.624 -10.007  35.433  1.00  0.00
ATOM   4419  C   GLN A 274      22.480 -13.701  30.441  1.00  0.00           C
ATOM   4420  O   GLN A 274      21.413 -13.883  29.860  1.00  0.00           O
ATOM   4421  N   GLY A 275      23.288 -14.696  30.845  1.00  0.00           N
ATOM   4422  HN  GLY A 275      24.130 -14.442  31.321  1.00  0.00
ATOM   4423  CA  GLY A 275      23.027 -16.147  30.639  1.00  0.00           C
ATOM   4424  HA1 GLY A 275      23.663 -16.481  29.944  1.00  0.00
ATOM   4425  HA2 GLY A 275      23.201 -16.619  31.503  1.00  0.00
ATOM   4426  C   GLY A 275      21.601 -16.440  30.190  1.00  0.00           C
ATOM   4427  OT1 GLY A 275      20.671 -16.461  31.001  1.00  0.00           O
ATOM   4428  OT2 GLY A 275      21.279 -17.641  29.989  1.00  0.00           O
ATOM   4429  HT2 GLY A 275      20.322 -17.686  29.701  1.00  0.00
TER
ATOM      1  C   LIG     1      10.409  -8.574   0.191  1.00  0.00      LIG
ATOM      2  C1  LIG     1       9.283  -8.184  -0.519  1.00  0.00      LIG
ATOM      3  C2  LIG     1       9.197  -6.879  -1.014  1.00  0.00      LIG
ATOM      4  C3  LIG     1      10.230  -5.950  -0.790  1.00  0.00      LIG
ATOM      5  C4  LIG     1      11.357  -6.374  -0.065  1.00  0.00      LIG
ATOM      6  C5  LIG     1      11.448  -7.683   0.420  1.00  0.00      LIG
ATOM      7  C6  LIG     1      10.138  -4.569  -1.305  1.00  0.00      LIG
ATOM      8  C7  LIG     1       8.979  -3.901  -1.452  1.00  0.00      LIG
ATOM      9  N   LIG     1       8.955  -2.589  -1.909  1.00  0.00      LIG
ATOM     10  N1  LIG     1       7.859  -1.812  -2.097  1.00  0.00      LIG
ATOM     11  C8  LIG     1       8.355  -0.640  -2.496  1.00  0.00      LIG
ATOM     12  C9  LIG     1       9.760  -0.637  -2.583  1.00  0.00      LIG
ATOM     13  C10 LIG     1      10.139  -1.912  -2.215  1.00  0.00      LIG
ATOM     14  N2  LIG     1      11.358  -2.527  -2.110  1.00  0.00      LIG
ATOM     15  C11 LIG     1      11.343  -3.759  -1.696  1.00  0.00      LIG
ATOM     16  C12 LIG     1      10.589   0.512  -2.960  1.00  0.00      LIG
ATOM     17  C13 LIG     1      10.108   1.756  -3.315  1.00  0.00      LIG
ATOM     18  C14 LIG     1      12.576   1.722  -3.237  1.00  0.00      LIG
ATOM     19  C15 LIG     1      12.018   0.486  -2.974  1.00  0.00      LIG
ATOM     20  F   LIG     1      10.494  -9.824   0.661  1.00  0.00      LIG
ATOM     21  S   LIG     1      11.362   2.877  -3.615  1.00  0.00      LIG
ATOM     22  C16 LIG     1      13.982   2.088  -3.222  1.00  0.00      LIG
ATOM     23  O   LIG     1      14.904   1.284  -3.403  1.00  0.00      LIG
ATOM     24  N3  LIG     1      14.231   3.406  -2.925  1.00  0.00      LIG
ATOM     25  C17 LIG     1      15.586   3.914  -2.679  1.00  0.00      LIG
ATOM     26  C18 LIG     1      15.552   5.392  -3.029  1.00  0.00      LIG
ATOM     27  C19 LIG     1      16.789   6.257  -3.030  1.00  0.00      LIG
ATOM     28  C20 LIG     1      16.154   5.874  -4.333  1.00  0.00      LIG
ATOM     29  C21 LIG     1      16.133   3.609  -1.252  1.00  0.00      LIG
ATOM     30  F1  LIG     1      15.240   2.920  -0.501  1.00  0.00      LIG
ATOM     31  F2  LIG     1      17.270   2.875  -1.306  1.00  0.00      LIG
ATOM     32  F3  LIG     1      16.424   4.738  -0.560  1.00  0.00      LIG
ATOM     33  H   LIG     1       8.480  -8.894  -0.687  1.00  0.00      LIG
ATOM     34  H1  LIG     1       8.309  -6.606  -1.581  1.00  0.00      LIG
ATOM     35  H2  LIG     1      12.179  -5.695   0.151  1.00  0.00      LIG
ATOM     36  H3  LIG     1      12.320  -8.008   0.978  1.00  0.00      LIG
ATOM     37  H4  LIG     1       8.006  -4.309  -1.216  1.00  0.00      LIG
ATOM     38  H5  LIG     1       7.660   0.165  -2.699  1.00  0.00      LIG
```

```
ATOM     39  H6  LIG     1      12.313  -4.278  -1.631  1.00  0.00      LIG
ATOM     40  H7  LIG     1       9.081   2.079  -3.403  1.00  0.00      LIG
ATOM     41  H8  LIG     1      12.628  -0.381  -2.740  1.00  0.00      LIG
ATOM     42  H9  LIG     1      13.446   3.925  -2.538  1.00  0.00      LIG
ATOM     43  H10 LIG     1      16.248   3.397  -3.386  1.00  0.00      LIG
ATOM     44  H11 LIG     1      14.638   5.876  -2.696  1.00  0.00      LIG
ATOM     45  H12 LIG     1      17.745   5.813  -2.777  1.00  0.00      LIG
ATOM     46  H13 LIG     1      16.682   7.286  -2.702  1.00  0.00      LIG
ATOM     47  H14 LIG     1      16.711   5.189  -4.964  1.00  0.00      LIG
ATOM     48  H15 LIG     1      15.626   6.643  -4.888  1.00  0.00      LIG
ENDMDL
```
